# Enantioselective three-component aminomethylation of α-diazo ketones with alcohols and 1,3,5-triazines

**DOI:** 10.1038/s41467-020-15345-2

**Published:** 2020-03-23

**Authors:** Jiuwei Che, Li Niu, Shikun Jia, Dong Xing, Wenhao Hu

**Affiliations:** 1 Shanghai Engineering Research Center of Molecular Therapeutics and New Drug Development, School of Chemistry and Molecular Engineering, East China Normal University, Shanghai, 200062 China; 2 Guangdong Key Laboratory of Chiral Molecule and Drug Discovery, School of Pharmaceutical Sciences, Sun Yat-sen University, Guangzhou, 510006 China

**Keywords:** Asymmetric catalysis, Homogeneous catalysis, Synthetic chemistry methodology

## Abstract

Enantioselective *α*-aminomethylation of carbonyl compounds constitutes a powerful protocol for introducing aminomethyl groups to simple organic molecules. However, current strategies rely on nucleophile-based enantioselective activation with inherently activated substrates only, and enantioselective protocol based on the activation of in situ-generated unstable formaldimines remains elusive, probably owing to their unstable nature and the lack of steric environment for efficient stereocontrols. Here, based on a rhodium/chiral phosphoric acid cooperative catalysis, we achieved an enantioselective three-component reaction of *α*-diazo ketones with alcohols and 1,3,5-triazines. A dual hydrogen bonding between the chiral phosphoric acid catalyst and two distinct active intermediates was proposed to be crucial for the efficient electrophile-based enantiocontrol. A series of chiral *β*-amino-*α*-hydroxy ketones including those derived from simple aliphatic alcohols, allylic alcohol, propargyl alcohol, complicated natural alcohols and water could all be prepared in high efficiency and enantioselectivity.

## Abstract (web summary)

Strategies for enantioselective *α*-aminomethylation of carbonyl compounds rely on the chiral activation of stable ketones substrate. Here, the authors report a rhodium/chiral phosphoric acid cooperative catalysis for the three-component reaction of *α*-diazo ketones with alcohols and 1,3,5-triazines via imine chiral activation.

## Introduction

**Fig. 1:**
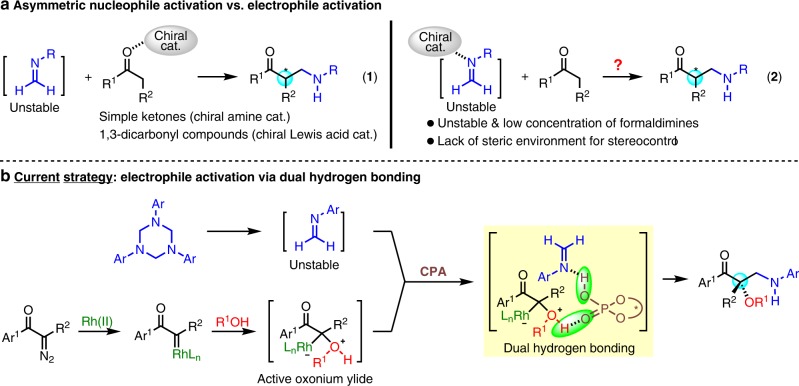
Reaction design for aminomethylation with formaldimines. **a** Previous aminomethylation work of nucleophile activation mode. **b** Design of CPA dual hydrogen bonding directed electrophile activation mode multicomponent aminomethylation.

As an important branch of Mannich reaction, the *α*-aminomethylation of carbonyl compounds constitutes a powerful protocol for introducing aminomethyl groups to simple organic molecules^1–8^. The resulting *β*-amino carbonyl compounds are versatile synthetic building blocks for a wide variety of natural products and biologically active compounds^9^. Different types of formaldehyde-derived imines or iminium salts, which are generally unstable and have to be in situ generated from formaldehyde with aromatic amines^10,11^, *α*-aminomethyl ethers^12–17^, *N*,*O*-acetals^18–20^, or 1,3,5-triaryl-1,3,5-triazines^21–24^, have been successfully applied. Within this context, enantioselective version of this transformation has also been achieved by catalytic asymmetric activation of the nucleophilic carbonyl compounds with either amine catalysts^10,11,14,16^ or chiral Lewis acid^23,24^. While this nucleophile-based activating strategy was feasible and showed good enantiocontrol, the nucleophiles were limited to inherently activated substrates such as unmodified ketones or 1,3-dicarbonyl compounds (Fig. 1a, Eq 1). On the other hand, while the activation of stable imines by chiral Brønsted acid catalysts has been extensively investigated in a variety of enantioselective Mannich reactions^25–36^, this electrophile-based activating protocol has not been applied to aminomethylation reactions with the in situ-generated formaldimines, probably owing to their generally unstable nature and the lack of steric environment on the carbon atom for efficient stereocontrols (Fig. 1a, Eq 2).

In recent years, metal carbene-involved multicomponent reactions (MCRs) based on electrophilic trapping of active onium ylides have emerged as a powerful strategy to enable previously inefficient or even impossible chemical transformations^37–44^. Among them, the Mannich-type trapping of oxonium ylides with aryl imines has offered a rapid way for the synthesis of *β*-amino alcohols^45–48^. By utilizing rhodium/chiral phosphoric acid (CPA) cooperative catalysis, efficient stereocontrol via a crucial dual hydrogen bonding activation^34,49,50^ from CPA toward both the oxonium ylide and the imine substrate has been achieved^45,46,48^.

Encouraged by these discoveries, we design a three-component reaction between *α*-diazo ketones, alcohols and 1,3,5-triaryl-1,3,5-triazines, anticipating that a similar dual hydrogen bonding activation would be operational (Fig. 1b), thus allowing an efficient electrophile-based asymmetric activation of formaldimines and offering an enantioselective aminomethylation reaction to give chiral *β*-amino-*α*-hydroxy ketones, which are widely existed structural scaffolds in synthetic and medicinal chemistry^51–54^. The challenges for the design of this three-component reaction are three-fold: (1) the in situ-generated formaldimine from 1,3,5-triazine has a low concentration in the reaction system, thus the electrophilic trapping of the active oxonium ylide species would be much less efficient and the undesired O–H insertion product^55^ might be predominant; (2) 1,3,5-triaryl-1,3,5-triazines may undergo [4 + 1] cycloaddition with diazo compounds under metal catalysis^56^, which would lead to low reactivity of the desired three-component reaction; (3) it is not trivial whether the proposed dual hydrogen bonding between CPA and the two reactive intermediates could be effectively formed, which is owing to the unstable nature, low concentration and the lack of substituents on the carbon atom of the in situ-generated formaldimines.

## Results

### Reaction optimization

By choosing 1,3,5-triphenyl-1,3,5-triazine **3a** as the formaldimine precursor, initial screening of different diazo compounds was conducted with benzylic alcohol **1a** under Rh(II)/phosphoric acid co-catalyzed conditions (For the initial screening of different diazo compounds, see Supplementary Fig. 1 in the Supplementary Information). It was found that diazoacetophenone **2a** reacted with **1a** and **3a** smoothly to afford three-component product **4a** in 43% yield when Rh_2_(OAc)_4_ and racemic phosphoric acid *rac-***6a** were employed as the co-catalysts (Table 1, entry 1). In comparison, in the absence of CPA, the desired three-component product was not observed, instead, the [4 + 1] cyclization product **8** from **2a** and **3a** was obtained as the major product^56^. A series of CPAs were screened (entries 2–10), and the one possessing a bulky 3,3’-bis(2,4,6-triisopropylphenyl)-BINOL backbone [(*R*)-**6j**] showed most promising result, giving **4a** in 61% yield with 74% ee (entry 10). When Rh_2_(OAc)_4_ was replaced with Rh_2_(esp)_2_, both the yield and enantioselectivity were further improved (entry 11) (for a screening of different rhodium catalyst, see Supplementary Table 1 in the Supplementary Information). When 1-phenyl-diazoacetophenone **2b** was used as the diazo source, the desired transformation occurred smoothly, affording **5a** possessing a quaternary carbon center in 81% yield with 80% ee (entry 12). Introducing an *ortho*-trifluoromethyl substituent to the aryl ring of benzylic alcohol, as well as lowering the reaction temperature to −10 °C, allowed the corresponding three-component product **5b** to be obtained in 81% yield with 94% ee (entries 13–14). Control experiments between **1b** and **2b** indicated that both rhodium and CPA catalysts were indispensable for this transformation (entries 15–16).

**Table 1:** Condition optimization^a^.


Entry	1	2	Rh(II)	6	4 or 5	Yield (%)^b^	Ee (%)^c^
1	**1a**	**2a**	Rh_2_(OAc)_4_	*rac*-**6a**	**4a**	43	–
2	**1a**	**2a**	Rh_2_(OAc)_4_	(*R*)-**6b**	**4a**	37	24
3	**1a**	**2a**	Rh_2_(OAc)_4_	(*R*)-**6c**	**4a**	31	12
4	**1a**	**2a**	Rh_2_(OAc)_4_	(*R*)-**6d**	**4a**	47	26
5	**1a**	**2a**	Rh_2_(OAc)_4_	(*R*)-**6e**	**4a**	53	43
6	**1a**	**2a**	Rh_2_(OAc)_4_	(*R*)-**6f**	**4a**	39	8
7	**1a**	**2a**	Rh_2_(OAc)_4_	(*R*)-**6g**	**4a**	45	16
8	**1a**	**2a**	Rh_2_(OAc)_4_	(*R*)-**6h**	**4a**	43	24
9	**1a**	**2a**	Rh_2_(OAc)_4_	(*R*)-**6i**	**4a**	<5	–
10	**1a**	**2a**	Rh_2_(OAc)_4_	(*R*)-**6j**	**4a**	61	72
11	**1a**	**2a**	Rh_2_(esp)_2_	(*R*)-**6j**	**4a**	80	72
12	**1a**	**2b**	Rh_2_(esp)_2_	(*R*)-**6j**	**5a**	81	78
13	**1b**	**2b**	Rh_2_(esp)_2_	(*R*)-**6j**	**5b**	84	90
14^d^	**1b**	**2b**	Rh_2_(esp)_2_	(*R*)-**6j**	**5b**	81	94
15	**1b**	**2b**	**–**	(*R*)-**6j**	**5b**	<5	–
16	**1b**	**2b**	Rh_2_(esp)_2_	(*R*)-**6j**	**5b**	<5	–

^a^All reactions were run in 0.1 mmol scale of **1**, **1**:**2**:**3a** = 1/1/0.33.

^b^Isolated yield.

^c^Determined by chiral HPLC analysis.

^d^At −10 °C.

### Substrate scope

**Fig. 2:**
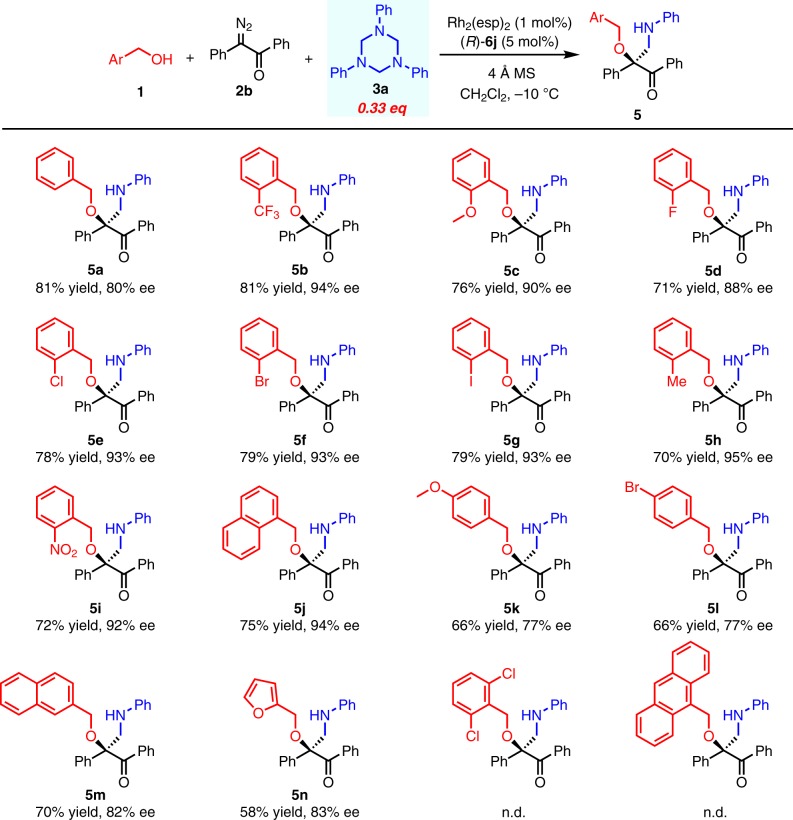
Substrate scope of benzylic alcohols. All reactions were run in 0.3 mmol scale of **1**, **1**:**2b**:**3a** = 1/1/0.33. All yields shown were based on isolated products. Ee values were determined by chiral HPLC analysis.

With the optimized reaction conditions in hand, the scope of this transformation was first investigated with different substituted benzylic alcohols (Fig. 2). Different *ortho*-substituents on the aryl ring of benzyl alcohols, including CF_3_, NO_2_, Cl, Br, I, MeO, Me, could be well tolerated, yielding the corresponding products in high yields with excellent ee (**5b-5c**, **5e-5i**). 1-Naphthylmethyl alcohol also worked well and showed excellent enantioselectivity (**5j**). Non-substituted benzylic alcohol or the one bearing fluoro substituent at *ortho*-position gave the corresponding products with decreased ee (**5a**, **5d**, **5k-5n**). These results indicated that the steric effects of the benzylic alcohol substrate, rather than its electronic properties, were more crucial for the enantiocontrol. Further increased bulkiness on the alcohol component completely inhibited the reactivity, indicating a strict balance between the steric effect and the reactivity of this transformation.

**Fig. 3:**
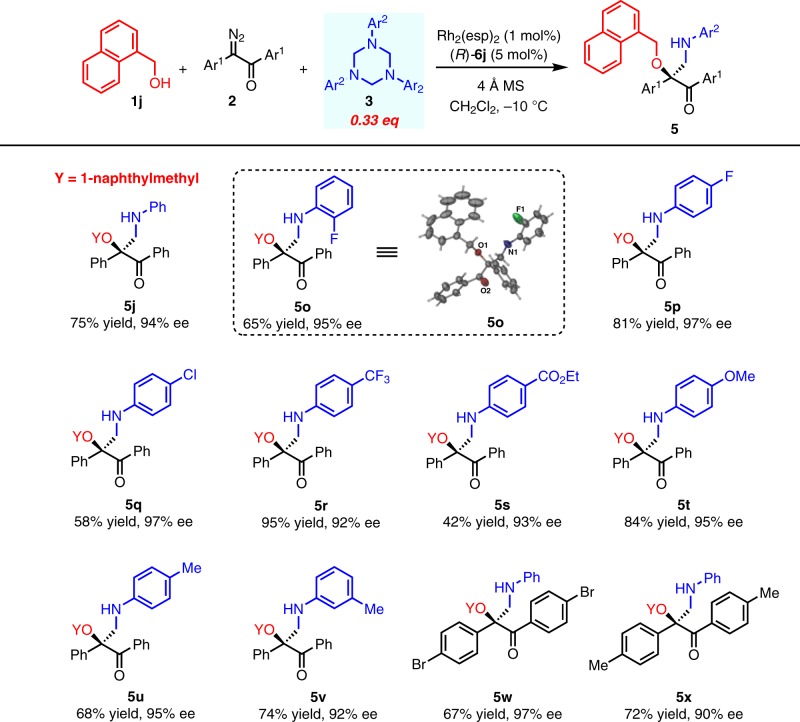
Substrates Scope of 1,3,5-Triaryl-1,3,5-triazines and diazo ketones. All reactions were run in 0.3 mmol scale of **1j**, **1j**:**2**:**3** = 1/1/0.33. All yields shown were based on isolated products. Ee values were determined by chiral HPLC analysis.

The scope of 1,3,5-triaryl-1,3,5- triazines was then investigated by choosing 1-naphthylmethyl alcohol **1j** as the alcohol (Fig. 3). In general, different substituents on the aryl ring of 1,3,5-triaryl-1,3,5-triazine, including Me, MeO, F, Cl, CF_3_, CO_2_Et at different positions, could all be well tolerated, yielding the corresponding products in moderate to good yields with excellent ee (**5o**-**5v**). Different substituted diazoacetophenones also worked well to give the desired products in good yields with excellent ee (**5w** and **5x**). The absolute configuration of **5o** was unambiguously determined as *S* by single crystal X-ray analysis.

**Fig. 4:**
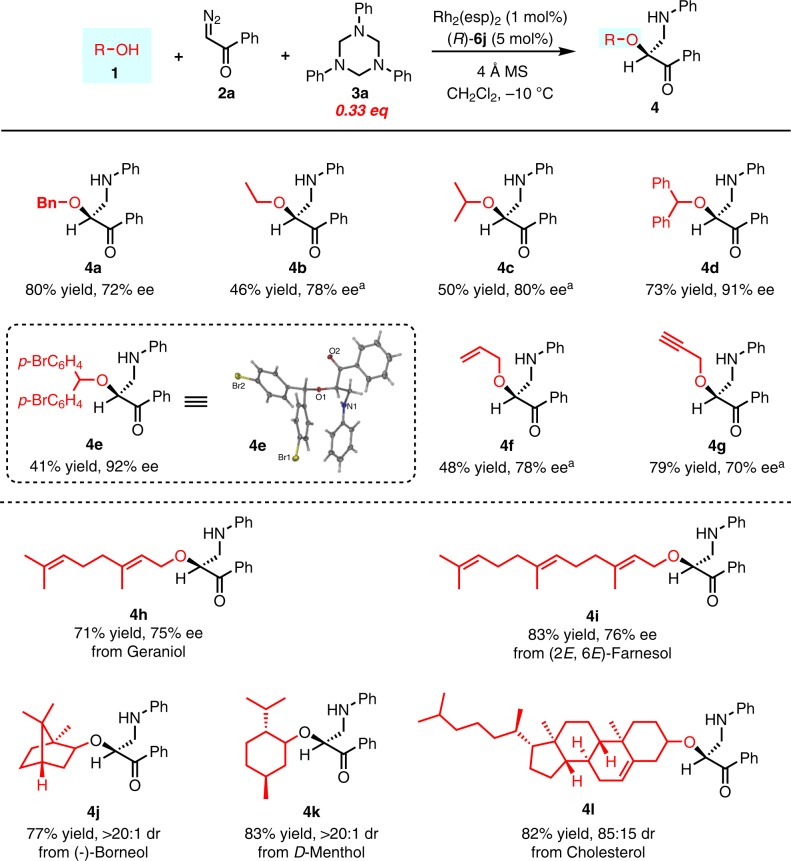
Substrates scope of different alcohols. Unless otherwise noted, the reactions were run in 0.3 mmol scale of **2a**, **1**:**2a**:**3a** = 1/1/0.33. All yields shown were based on isolated products. Ee values were determined by chiral HPLC analysis. Dr values was determined by ^1^H NMR of the crude reaction mixture. ^a^The reactions were run in 0.3 mmol scale of **2a**, **1**:**2a**:**3** = 3/1/0.33.

In view of the great synthetic potential of the resulted chiral *β*-amino-*α*-hydroxy ketones, further efforts were made to expand the scope of alcohols (Fig. 4). Gratifyingly, by choosing diazoacetophenone **2a** as the diazo source (For the screening of different diazo compounds, see Supplementary Fig. 2 in the Supplementary Information), aliphatic alcohols such as ethanol and 2-propanol reacted smoothly under standard conditions to give the corresponding products in moderate yields with high ee (**4b** and **4c**). Diarylmethanols also worked well to give the desired products with excellent ee (**4d** and **4e**) and the absolute configuration of the products derived from **2a** was confirmed to be *S* by single crystal X-ray analysis of **4e**. Allyl alcohol and propargyl alcohol were good substrates, affording the corresponding products in moderate to good yields with slightly decreased ee (**4f** and **4g**). The introduced allyl and propargyl groups offered a convenient way for further synthetic elaborations of the resulted *β*-amino ketones.

This method also offers an efficient protocol for the late-stage functionalization of complicated natural alcohols to introduce chiral *β*-amino-*α*-hydroxy ketone side chains. For example, geraniol and (2*E*, 6*E*)-farnesol were converted to corresponding three-component products in good yields with high ee (**4h** and **4i**). (−)-Borneol and *D*-menthol also worked well to give desired product **4j** and **4k** in good yields with excellent dr. Cholesterol underwent this transformation to give the corresponding product **4l** in 82% yield with 85:15 dr. To investigate the match/mismatch effect of this transformation, (*R*)-**6j** and (*S*)-**6j** were separately used as the catalyst for the three-component reactions of several chiral alcohols including (−)-borneol, *D*-menthol or *L*-menthol, reversed diastereoselectivities were generally observed (for detailed results, see Supplementary Table 2 in the Supplementary Information), indicating that the stereoselectivity of this transformation was mostly controlled by the chiral catalyst rather than the chiral substrate.

**Fig. 5:**
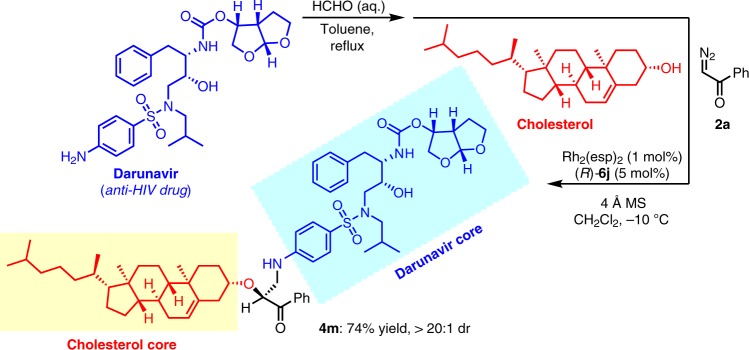
Drug linkage of Darunavir and cholesterol. Using Our protocol as a linkage tool reaction to connect Darunavir and cholesterol.

The modification of commercial drugs and connection of two pharmaceutical fragments are useful for identifying potentially new drug molecules^57–60^. With the current strategy, an anti-HIV drug Darunavir^61^ was successfully connected with cholesterol via a triazine formation/three-component reaction sequence, providing new compound **4m** in high yield and dr (Fig. 5).

**Fig. 6:**
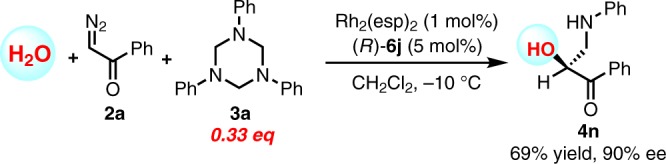
H_2_O derived three-component aminomethylation. Using water as nucleophile to directly afford *O*-unprotected *β*-amino-*α*-hydroxy ketone.

More strikingly, water could be used as nucleophile to undergo current transformation to deliver the three-component product **4n** in good yield and excellent ee (Fig. 6), thus offering a straightforward way to chiral *β*-amino-*α*-hydroxy ketones.

### Mechanistic studies

**Fig. 7:**
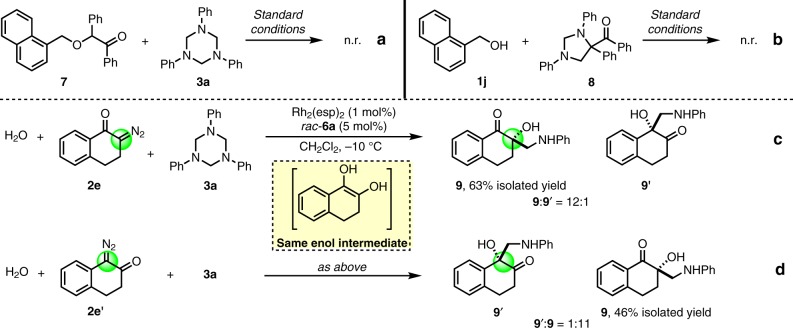
Mechanistic studies. **a** Control reaction to exclude the formation of product **5j** from insertion product **7**. **b** Control reaction to exclude the formation of product **5j** from cycloaddition product **8**. **c**, **d** Control reactions to verify the involvement of enol intermediate with diazo ketone **2e** and **2e’**.

To gain some insights into the pathway of this transformation, two control experiments were conducted. First, the O–H insertion product **7** derived from **1j** and **2b** was allowed to react with **3a** under the optimal conditions, and the Mannich-type product **5j** was not detected (Fig. 7a). This result indicated that a stepwise O–H insertion/Mannich reaction pathway is unlikely involved in current transformation. On the other hand, the [4 + 1] cycloaddition product **8** derived from **2b** and **3a**, which has been observed as the major product when Rh(II) alone was used as the catalyst, failed to react with **1j** under the optimal reaction conditions (Fig. 7b), thus extruding the involvement of **8** as the intermediate for this transformation.

In previously reported O–H insertion and rearrangement transformations between *α*-diazo ketones and alcohols, an enol intermediate has been proposed and characterized as the key intermediate^62,63^. Recent related DFT studies also supported the involvement of an enol intermediate in these processes^64–66^. To verify whether an enol intermediate was also involved for the current transformation, parallel experiments starting from 2-diazo-1-tetralone (**2e**) or 1-diazo-2-tetralone (**2e’**) were conducted. For both reactions, the same three-component product **9** whose tertiary carbon was located at the 2-position were obtained as the major product (Fig. 7c, d), indicating that an enol intermediate, rather than an oxonium ylide, might be involved for this transformation.

## Discussion

In summary, a highly enantioselective three-component reaction of *α*-diazo ketones with 1,3,5-triazines and alcohols was developed under Rh(II)/chiral phosphoric acid cooperative catalysis. This reaction offers an efficient electrophile-based asymmetric activation mode of formaldimines for aminomethylation. A very broad scope of alcohols, including simple aliphatic alcohols, complicated natural alcohols and water, could all be applied, leading to a series of chiral *β*-amino-*α*-hydroxy ketones in good efficiency and high enantiocontrol. Efforts on understanding the stereocontrol of this transformation as well as applying this protocol to the trapping of other types of active intermediates are ongoing.

## Methods

### General methods

See Supplementary Methods for further details.

### Typical procedure for the aminomethylation reaction

Under a nitrogen atmosphere, a suspension of Rh_2_(esp)_2_ (1.0 mol%), chiral phosphoric acid (*R*)-**6j** (5 mol%), 4 Å molecular sieve (300 mg) was stirred in 2.0 mL of CH_2_Cl_2_ at −10 °C and then the mixture of alcohol **1b** (0.3 mmol), diazoacetophenones **2b** (0.3 mmol) and 1,3,5-triaryl-1,3,5-triazinanes **3a** (0.1 mmol) in 2 mL of CH_2_Cl_2_ was introduced to the suspension over 2 h via a syringe pump. After completion of the addition, the reaction mixture was stirred for another 6 h until the diazo compound was completely consumed. The reaction mixture was then filtered through a short pad of Celite® and the filtrate was concentrated to give a residue which was subjected to HPLC for the ee values. Purification of the crude products by flash chromatography on silica gel (eluent: EtOAc/light petroleum ether = 1/80~1/40) afforded pure products (*S*)-**5b**. White solid, 81% yield, 94% ee, ^1^H NMR (400 MHz, CDCl_3_) δ 7.91 (s, 2 H), 7.69–6.99 (m, 14 H), 6.70–6.47 (m, 3 H), 4.88 (d, *J* = 10.1 Hz, 1 H), 4.38 (d, *J* = 10.1 Hz, 1 H), 4.08 (d, *J* = 12.5 Hz, 1 H), 3.87 (d, *J* = 12.1 Hz, 1 H), 3.81 (s, 1 H). ^13^C NMR (101 MHz, CDCl_3_) δ 199.80, 147.88, 138.96, 135.40, 134.59, 133.07, 131.82, 130.26, 130.11, 129.08, 128.92, 128.20, 128.13, 127.86, 125.83, 125.13, 117.36, 99.99, 87.31, 63.32, 47.80. ^19^F NMR (376 MHz, CDCl_3_) δ -59.68. HRMS: Calcd. for C_29_H_25_F_3_NO_2_ (M + H)^+^: 476.1837, found: 476.1804, HPLC (Chiral IA, λ = 254 nm, hexane/2-propanol = 20/1, Flow rate = 1.0 mL/min), *t*_major_ = 5.81 min, *t*_minor_ = 6.68 min.

## Supplementary information

Supplementary Information

## Data Availability

Additional data supporting the findings described in this manuscript are available in the Supplementary Information. For full characterization data of new compounds and experimental details, see Supplementary Methods. For the ^1^H, ^13^C and ^19^F NMR spectra of new compounds, see Supplementary Figs. 5–92. The supplementary crystallographic data for **5o** (Supplementary Fig. 3), **4e** (Supplementary Fig. 4) are available free of charge from the Cambridge Crystallographic Data Centre under reference numbers CCDC1887265 (**5o**), CCDC1887266 (**4e**) via https://www.ccdc.cam.ac.uk/. All other data are available from the authors upon reasonable request.

## References

[1] Tramontini M, Angiolini L (1990). Further advances in the chemistry of Mannich bases. Tetrahedron.

[2] Córdova A (2004). The direct catalytic asymmetric Mannich reaction. Acc. Chem. Res..

[3] Notz W, Tanaka F, Barbas CF (2004). Enamine-based organocatalysis with proline and diamines: the development of direct catalytic asymmetric Aldol, Mannich, Michael, and Diels−Alder reactions. Acc. Chem. Res..

[4] Ting A, Schaus SE (2007). Organocatalytic asymmetric Mannich reactions: new methodology, catalyst design, and synthetic applications. Eur. J. Org. Chem..

[5] Verkade JMM, Hemert LJCV, Quaedflieg PJLM, Rutjes FPJT (2008). Organocatalysed asymmetric Mannich reactions. Chem. Soc. Rev..

[6] Weiner B, Szymanski W, Janssen DB, Minnaard AJ, Feringa BL (2010). Recent advances in the catalytic asymmetric synthesis of β-amino acids. Chem. Soc. Rev..

[7] Karimi B, Enders D, Jafari E (2013). Recent advances in metal-catalyzed asymmetric Mannich reactions. Synthesis.

[8] Roselll MS, Pozo C, Fustero S (2016). A decade of advance in the asymmetric vinylogous Mannich reaction. Synthesis.

[9] Roman G (2015). Mannich bases in medicinal chemistry and drug design. Eur. J. Med. Chem..

[10] Ibrahem I, Casas J, Córdova A (2004). Direct catalytic enantioselective α-aminomethylation of ketones. Angew. Chem. Int. Ed..

[11] Sundén H, Ibrahem I, Eriksson L, Córdova A (2005). Direct catalytic enantioselective Aza-Diels-Alder reactions. Angew. Chem. Int. Ed..

[12] Enders D, Ward D, Adam J, Raabe G (1996). Efficient Regio- and enantioselective Mannich reactions. Angew. Chem. Int. Ed..

[13] Enders D, Adam J, Oberbörsch S, Ward D (2002). Asymmetric Mannich reactions by α-silyl controlled aminomethylation of ketones. Synthesis.

[14] Ibrahem I, Dziedzic P, Córdova A (2006). Organocatalytic asymmetric α-aminomethylation of cyclohexanones. Synthesis.

[15] Chi Y, Gellman SH (2006). Enantioselective organocatalytic aminomethylation of aldehydes: a role for ionic interactions and efficient access to β^2^-amino acids. J. Am. Chem. Soc..

[16] Ibrahem I, Zhao G-L, Córdova A (2007). Direct catalytic enantioselective α-aminomethylation of aldehydes. Chem. Eur. J..

[17] Xu J (2015). Aminomethylation of enals through carbene and acid cooperative catalysis: concise access to β^2^-amino acids. Angew. Chem. Int. Ed..

[18] Kim C (2009). Formal alkyne Aza-Prins cyclization: gold(I)-catalyzed cycloisomerization of mixed N,O-acetals generated from homopropargylic amines to highly substituted piperidines. J. Am. Chem. Soc..

[19] Boaz NC, Bair NC, Le TT, Peelen TJ (2010). Activation of Fmoc-protected N,O-acetals using trimethylsilyl halides: mechanistic and synthetic studies. Org. Lett..

[20] You Y, Zhang L, Cui L, Mi X, Luo S (2017). Catalytic asymmetric Mannich reaction with N-carbamoyl imine surrogates of formaldehyde and glyoxylate. Angew. Chem. Int. Ed..

[21] Oda S, Sam B, Krische MJ (2015). Hydroaminomethylation beyond carbonylation: allene-imine reductive coupling by ruthenium-catalyzed transfer hydrogenation. Angew. Chem. Int. Ed..

[22] Oda S, Franke J, Krische MJ (2016). Diene hydroaminomethylation via ruthenium-catalyzed C–C bond forming transfer hydrogenation: beyond carbonylation. Chem. Sci..

[23] Lian X (2017). A new approach to the asymmetric Mannich reaction catalyzed by chiral N,N‘-dioxide-metal complexes. Chem. Sci..

[24] Gong J, Li S-W, Qurban S, Kang Q (2017). Enantioselective Mannich reaction employing 1,3,5-triaryl-1,3,5-triazinanes catalyzed by chiral-at-metal rhodium complexes. Eur. J. Org. Chem..

[25] Uraguchi D, Terada M (2004). Chiral Brønsted acid-catalyzed direct Mannich reactions via electrophilic activation. J. Am. Chem. Soc..

[26] Akiyama T, Itoh J, Yokota K, Fuchibe K (2004). Enantioselective Mannich-type reaction catalyzed by a chiral Brønsted acid. Angew. Chem. Int. Ed..

[27] Zhang D, Zhou J, Xia F, Kang Z, Hu W (2015). Bond cleavage, fragment modification and reassembly in enantioselective three-component reactions. Nat. Commun..

[28] Wang Y-B, Zheng S-C, Hu Y-M, Tan B (2017). Brønsted acid-catalysed enantioselective construction of axially chiral arylquinazolinones. Nat. Commun..

[29] Zhang L (2019). Phosphoric acid-catalyzed atroposelective construction of axially chiral arylpyrroles. Nat. Commun..

[30] Gong W (2019). Permanent porous hydrogen-bonded frameworks with two types of Brønsted acid sites for heterogeneous asymmetric catalysis. Nat. Commun..

[31] Zhang J (2018). Asymmetric phosphoric acid–catalyzed four-component Ugi reaction. Science.

[32] Connon SJ (2006). Chiral phosphoric acids: powerful organocatalysts for asymmetric addition reactions to imines. Angew. Chem. Int. Ed..

[33] Yu J, Shi F, Gong L-Z (2011). Brønsted-acid-catalyzed asymmetric multicomponent reactions for the facile synthesis of highly enantioenriched structurally diverse nitrogenous heterocycles. Acc. Chem. Res..

[34] Reid JP, Simón L, Goodman JM (2016). A practical guide for predicting the stereochemistry of bifunctional phosphoric acid catalyzed reactions of imines. Acc. Chem. Res..

[35] Wang Y-B, Tan B (2018). Construction of axially chiral compounds via asymmetric organocatalysis. Acc. Chem. Res..

[36] Wang Q, Wang D-X, Wang M-X, Zhu J (2018). Still unconquered: enantioselective Passerini and Ugi multicomponent reactions. Acc. Chem. Res..

[37] Guo X, Hu W (2013). Novel multicomponent reactions via trapping of protic onium ylides with electrophiles. Acc. Chem. Res..

[38] Zhang D, Hu W (2017). Asymmetric multicomponent reactions based on trapping of active intermediates. Chem. Rec..

[39] Xia Y, Qiu D, Wang J (2017). Transition-metal-catalyzed cross-couplings through carbene migratory insertion. Chem. Rev..

[40] Qiu H (2012). Highly enantioselective trapping of zwitterionic intermediates by imines. Nat. Chem..

[41] Zhou C-Y (2012). Dirhodium carboxylates catalyzed enantioselective coupling reactions of α-diazophosphonates, anilines, and electron-deficient aldehydes. Angew. Chem. Int. Ed..

[42] Jia S, Xing D, Zhang D, Hu W (2014). Catalytic asymmetric functionalization of aromatic C-H bonds by electrophilic trapping of metal-carbene-induced zwitterionic intermediates. Angew. Chem. Int. Ed..

[43] Nicolle SM, Lewis W, Hayes CJ, Moody CJ (2016). Stereoselective synthesis of functionalized pyrrolidines by the diverted N−H insertion reaction of metallocarbenes with β-aminoketone derivatives. Angew. Chem. Int. Ed..

[44] Yuan W, Eriksson L, Szabý KJ (2016). Rhodium-catalyzed geminal oxyfluorination and oxytrifluoro-methylation of diazocarbonyl compounds. Angew. Chem. Int. Ed..

[45] Huang H, Guo X, Hu W (2007). Efficient trapping of oxonium ylides with imines: A highly diastereoselective three-component reaction for the synthesis of β-amino-α-hydroxyesters with quaternary stereocenters. Angew. Chem. Int. Ed..

[46] Hu W (2008). Cooperative catalysis with chiral Brønsted acid-Rh_2_(OAc)_4_: highly enantioselective three-component reactions of diazo compounds with alcohols and imines. J. Am. Chem. Soc..

[47] Guan X-Y, Yang L-P, Hu W (2010). Cooperative catalysis in multicomponent reactions: highly enantioselective synthesis of γ-hydroxyketones with a quaternary carbon stereocenter. Angew. Chem. Int. Ed..

[48] Wei H (2018). Enantioselective oxidative cyclization/Mannich addition enabled by gold(I)/chiral phosphoric acid cooperative catalysis. Angew. Chem. Int. Ed..

[49] Akiyama T (2007). Stronger Brønsted acids. Chem. Rev..

[50] Parmar D, Sugiono E, Raja S, Rueping M (2014). Complete field guide to asymmetric BINOL-phosphate derived Brønsted acid and metal catalysis: history and classification by mode of activation, Brønsted acidity, hydrogen bonding, ion pairing, and metal phosphates. Chem. Rev..

[51] Ogata M (1987). Synthesis and oral antifungal activity of novel azolylpropanolones and related compounds. J. Med. Chem..

[52] Takasugi M, Monde K, Katsui N, Shirata A (1987). Spirobrassinin, a novel sulfur-containing Phytoalexin from the Daikon Rhaphanus sativus L. var. hortensis (Cruciferae). Chem. Lett..

[53] Monde K, Sasaki K, Shirata A, Takasugi M (1991). Brassicanal C and two dioxindoles from cabbage. Phytochemistry.

[54] Liu Y (2018). Catalytic enantioselective radical coupling of activated ketones with N-aryl glycines. Chem. Sci..

[55] Doyle, M. P., McKervey, M. A., Ye, T. *Modern Catalytic Methods for Organic Synthesis with Diazo Compounds (From Cyclopropanes to Ylides)* (Wiley-Interscience Press, New York, 1998).

[56] Zhu C, Xu G, Sun J (2016). Gold-catalyzed formal [4+1]/[4+3] cycloadditions of diazo esters with triazines. Angew. Chem. Int. Ed..

[57] Thirumurugan P, Matosiuk D, Jozwiak K (2013). Click chemistry for drug development and diverse chemical–biology applications. Chem. Rev..

[58] Abendroth F, Seitz O (2014). Double-clicking peptides onto phosphorothioate oligonucleotides: combining two proapoptotic agents in one molecule. Angew. Chem. Int. Ed..

[59] Kacprzak K, Skiera I, Piasecka M, Paryzek Z (2016). Alkaloids and isoprenoids modification by copper(I)-catalyzed Huisgen 1,3-dipolar cycloaddition (click chemistry): toward new functions and molecular architectures. Chem. Rev..

[60] Simonetti M, Cannas DM, Just-Baringo X, Vitorica-Yrezabal IJ, Larrosa I (2018). Cyclometallated ruthenium catalyst enables late-stage directed Arylation of pharmaceuticals. Nat. Chem..

[61] Kanters S (2017). Comparative efficacy and safety of second-line antiretroviral therapy for treatment of HIV/AIDS: a systematic review and network meta-analysis. Lancet HIV.

[62] Wood JL, Moniz GA (1999). Rhodium carbenoid-initiated Claisen rearrangement: scope and mechanistic observations. Org. Lett..

[63] Moniz GA, Wood JL (2001). Catalyst-based control of [2,3]- and [3,3]-rearrangement in α-diazoketone-derived propargyloxy enols. J. Am. Chem. Soc..

[64] Li Z (2012). Scope and mechanistic analysis of the enantioselective synthesis of allenes by rhodium-catalyzed tandem ylide formation/[2,3]-sigmatropic rearrangement between donor/acceptor carbenoids and propargylic alcohols. J. Am. Chem. Soc..

[65] Xu B (2014). Highly enantioselective S–H bond insertion cooperatively catalyzed by dirhodium complexes and chiral spiro phosphoric acids. Chem. Sci..

[66] Ren Y-Y, Zhu S-F, Zhou Q-L (2018). Chiral proton-transfer shuttle catalysts for carbene insertion reactions. Org. Biomol. Chem..

