# Supplementary material for: Enantioselective three-component aminomethylation of α-diazo ketones with alcohols and 1,3,5-triazines
Source: Nat Commun. 2020 Mar 23;11:1511. doi: 10.1038/s41467-020-15345-2 (PMC7089982; doi:10.1038/s41467-020-15345-2)
Supplement: Supplementary file 1 — Supplementary Information [file 41467_2020_15345_MOESM1_ESM.pdf]

Supplementary Information

Enantioselective Three-Component Aminomethylation of α- Diazo Ketones with Alcohols and 1,3,5-Triazines

Jiuwei Che,[†] Li Niu,[†] Shikun Jia,[‡] Dong Xing[*,†] and Wenhao Hu[*,‡]

[†]Shanghai Engineering Research Center of Molecular Therapeutics and New Drug Development, School of Chemistry and Molecular Engineering, East China Normal University, Shanghai 200062, China

[‡]School of Pharmaceutical Sciences, Sun Yat-sen University, Guangzhou 510006, China

E-mail: dxing@sat.ecnu.edu.cn; huwh9@mail.sysu.edu.cn.

Supplementary Methods.

1. General Information and Materials

General: All ^{1}H NMR (400 MHz) and ^{13}C NMR (100 MHz) and ^{19}F NMR (376 MHz) spectra were recorded on Brucker spectrometers in $CDCl_3$. Tetramethylsilane (TMS) served as an internal standard ($\delta = 0$) for ^{1}H NMR, and $CDCl_3$ was used as internal standard ($\delta = 77.0$) for ^{13}C NMR. Chemical shifts are reported in parts per million as follows: chemical shift, multiplicity (s =singlet, d = doublet, t = triplet, q = quartet, m = multiplet, br = broad). High-resolution mass spectrometry (HRMS) was performed on IonSpec FT-ICR or Waters Micromass Q-TOF micro Synapt High Definition Mass Spectrometer. HPLC analysis was performed on Dalian Elite (UV230+ UV/Vis Detector and P230P High Pressure Pump). Chiralpak IA column was purchased from Daicel Chemical Industries, LTD. Melting points were uncorrected. Single crystal X-ray diffraction data (**5o** and **4e**) were recorded on Bruker-AXS SMART APEX II single crystal X-ray diffractometer. The racemic standards used in HPLC studies were prepared according to the general procedure by using racemic BINOL derived phosphoric acid catalysts.

All reactions and manipulations were carried out under an argon atmosphere in a flame-dried or oven-dried flask containing magnetic stir bar. Bis[rhodium(α,α,α′,α′-tetramethyl-1,3-benzenedipropionic acid)] [$Rh_2(esp)_2$] and alcohols **1** were used directly after purchased from Sigma-Aldrich®. Diazoacetophenone **2** were prepared according to the literature method.[1-2] 1,3,5-Triaryl-1,3,5-triazinanes **3** were prepared from condensation of the corresponding aromatic amines with formalin according to the literature method.[3] Chiral phosphoric acids (PPAs) **6a-j** were prepared according to the literature procedure.[4-7] Dichloromethane (DCM) was distilled over calcium hydride. 4 Å molecular sieves was dried in a Muffle furnace at 250 °C over 5 hrs. Solvents for the column chromatography were distilled before use.

2. General Procedure I for Optimization of Three-Component Reactions (Table 1):

Under a nitrogen atmosphere, a suspension of Rh(II) catalyst (1.0 mol%), racemic or chiral phosphoric acid **6** (5 mol%), 4 Å molecular sieve (0.1 g) was stirred in 1.0 mL of CH_2Cl_2 at 0℃ and then the mixture of alcohol **1** (0.1 mmol), diazoacetophenone **2** (0.1 mmol) and 1,3,5-triphenyl-1,3,5-triazinane **3a** (0.033 mmol) in 1mL of CH_2Cl_2 was introduced to the suspension over 1 h via a syringe pump. After completion of the addition, the reaction mixture was stirred for another 2 h, then filtered and the filtrate was concentrated to give

a residue which was subjected to HPLC for the ee values. Purification of the crude products by flash chromatography on silica gel (eluent: EtOAc/light petroleum ether = 1/80~1/40) afforded pure products.

3. General Procedure II for Enantioselective Three-Component Reactions (Table 2, Scheme 3, Scheme 4, Scheme 5):

Under a nitrogen atmosphere, a suspension of $Rh_2(esp)_2$ (1.0 mol%), chiral phosphoric acid (*R*)-**6j** (5 mol%), 4 Å molecular sieve (0.3 g) was stirred in 2.0 mL of CH_2Cl_2 at -10 ^oC and then the mixture of alcohol **1** (0.3 mmol), diazoacetophenones **2** (0.3 mmol) and 1,3,5-triaryl-1,3,5-triazinanes **3** (0.1 mmol) in 2 mL of CH_2Cl_2 was introduced to the suspension over 2 h via a syringe pump. After completion of the addition, the reaction mixture was stirred for another 6 h until the diazo completely consumed, then filtered and the filtrate was concentrated to give a residue which was subjected to HPLC for the ee values. Purification of the crude products by flash chromatography on silica gel (eluent: EtOAc/light petroleum ether = 1/80~1/40) afforded pure products.

4. General Procedure III for the Enantioselective Three-Component Reactions of Water (Figure 3, Figure 4, Eq 5 and 6):

Under a nitrogen atmosphere, a suspension of $Rh_2(esp)_2$ (1.0 mol%), chiral phosphoric acid (*R*)-**6j** (5 mol%) and H_2O (3 mmol) was stirred in 2.0 mL of CH_2Cl_2 at -10 ^oC and then the mixture of diazoacetophenones **2** (0.3 mmol) and 1,3,5-triaryl-1,3,5-triazinanes **3** (0.1 mmol) in 2 mL of CH_2Cl_2 was introduced to the suspension over 2 h via a syringe pump. After completion of the addition, the reaction mixture was stirred for another 6 h until the diazo completely consumed, then filtered and the filtrate was concentrated to give a residue which was subjected to HPLC for the ee values. Purification of the crude products by flash chromatography on silica gel (eluent: EtOAc/light petroleum ether = 1/10~1/20) afforded the pure products. For the reactions starting from **2e** and **2e’** (Figure 4, Eq 5 and 6), racemic phosphoric acid *rac*-**6a** was used instead of (*R*)-**6j**, the ratio between the products **9** and **9’** was determined by HPLC analysis of the crude reaction mixture.

5. Screening of Different Diazo Compounds with Triazine and Benzyl Alcohol:

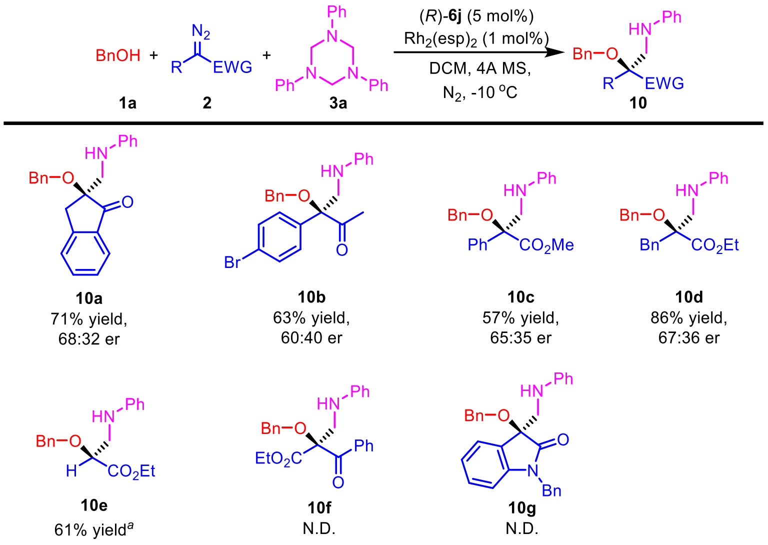

Supplementary Figure 1 | **Screening of Different Diazo Compound with Triazine and Benzyl Alcohol**. All reactions were conducted in 0.3 mmol scale of **1a**, **1a**:**2**:**3a**= 1/1/0.33. Yields of isolated products after column chromatography were reported and ee values were determined by HPLC analysis using a chiral stationary phase. [a]*rac*-**6a** was used as the catalyst and the use of (*R*)-**6j** as the catalyst led no product formation.

6. Screening of Different Diazo Compounds with Triazine and EtOH (or H_2O):

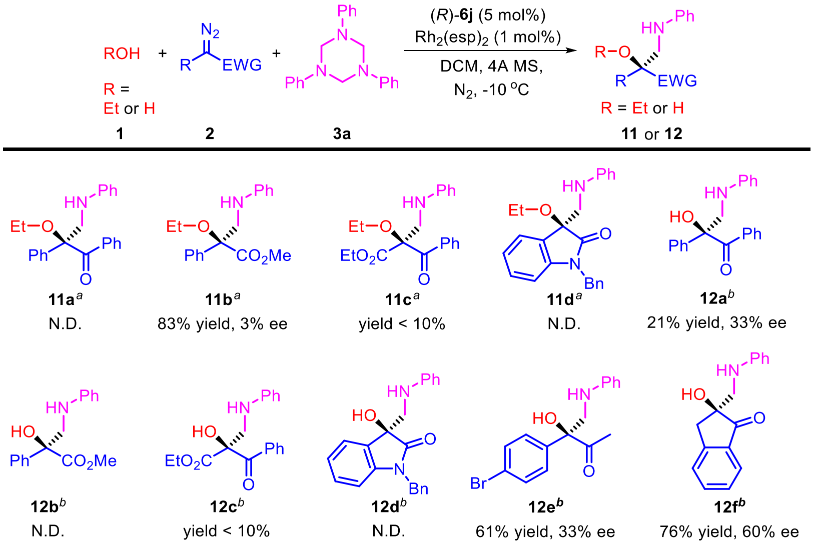

Supplementary Figure 2 | **Screening of Different Diazo Compounds with Triazine and EtOH (or H_2O)**. [a]All reactions were conducted in 0.3 mmol scale of **3a**, **1**:**2**:**3a**= 3/1/0.33. [b]All reactions were conducted in 0.1 mmol scale of **3a**, **1**:**2**:**3a**= 10/1/0.33. No 4 Å MS added.

7. Screening of Rhodium (II) Catalysts.

Supplementary Table 1. Screening of Rhodium (II) Catalysts.[a]

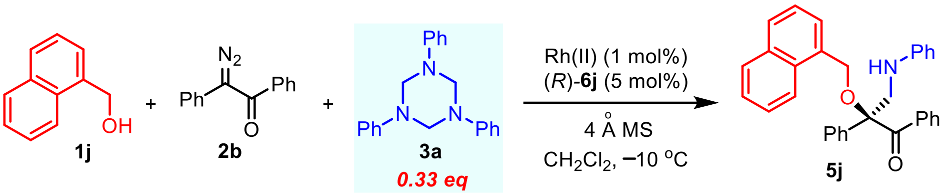

Entry	Rh(II) cat.	Yield (%)	Ee (%)
1	$Rh_2(esp)_2$	75	94
2	$Rh_2(OAc)_4$	54	93
3	$Rh_2(oct)_4$	51	83
4	$Rh_2(Ph_3CCO_2)_4$	81	89
5	$Rh_2(TFA)_4$	37	90
6	Rh_2(*S*-nttl)$_4$	63	96
7	$Rh_2(cap)_4$	n.r.	/

[a]All reactions were run in 0.1 mmol scale of **1**, **1j**:**2b**:**3a** = 1/1/0.33. All yields shown were based on isolated products. Ee values were determined by chiral HPLC analysis.

8. Parallel Experiments with both (*R*)-6j and (*S*)-6j.

Supplementary Table 2. Control Experiments on Match/Mismatch Effect.[a]

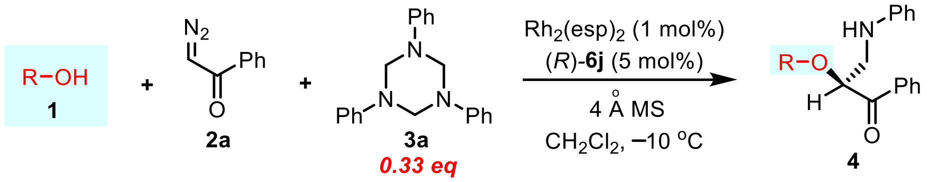

Entry	ROH	CPA*	Yield (%)	dr
1[b]	(-)-borneol	(*R*)-**6j**	72	85:15
2[b]		(*S*)-**6j**	69	10:90
3	*D*-menthol	(*R*)-**6j**	83	>20:1
4		(*S*)-**6j**	85	21:79
5	*L*-menthol	(*R*)-**6j**	64	>20:1
6		(*S*)-**6j**	66	<1:20

[a]Unless otherwise noted, all reactions were run in 0.3 mmol scale of **1**, **1**:**2a**:**3a** = 1/1/0.33. All yields shown were based on isolated products. Dr values was determined by HPLC. [b]Reaction was conducted at 0 °C.

9. Characterization Data of Compounds

(*S*)-2-(benzyloxy)-1,2-diphenyl-3-(phenylamino)propan-1-one (5a):

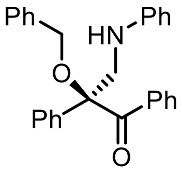

5a

(*S*)-**5a**: White solid, 81% yield, 78% ee; ^{1}H NMR (400 MHz, $CDCl_3$) δ 8.00 (d, 2H), 7.60 (d, *J* = 7.7 Hz, 2H), 7.44 (t, *J* = 7.4 Hz, 1H), 7.38 – 7.22 (m, 8H), 7.16 – 7.07 (m, 4H), 6.66 (t, *J* = 7.3 Hz, 1H), 6.58 (d, *J* = 8.0 Hz, 2H), 4.61 (d, *J* = 10.2 Hz, 1H), 4.28 (d, *J* = 10.2 Hz, 1H), 4.12 (d, *J* = 12.5 Hz, 1H), 3.82 (d, *J* = 12.8 Hz, 1H), 3.75 (s, 1H). ^{13}C NMR (101 MHz, $CDCl_3$) δ 199.77, 148.07, 139.21, 137.17, 134.67, 133.10, 130.11, 129.13, 128.86, 128.36,

128.17, 128.10, 128.06, 127.90, 125.16, 117.39, 112.93, 99.99, 86.78, 66.82, 47.78. HRMS (ESI): Calcd. for $C_{28}H_{26}NO_2$ $(M+H)^+$: 408.1964; found: 408.1979; HPLC (Chiral IC, λ= 254 nm, hexane/2-propanol = 80/1, Flow rate = 1.0 mL/min), t_{major} = 6.44 min, t_{minor} = 7.18 min.

(*S*)-1,2-diphenyl-3-(phenylamino)-2-((2-(trifluoromethyl)benzyl)oxy)propan-1-one (5b):

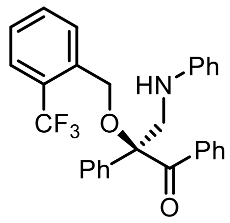

5b

(*S*)-**5b**: White solid, 81% yield, 94% ee; ^1H NMR (400 MHz, $CDCl_3$) δ 7.91 (s, 2H), 7.69 – 6.99 (m, 14H), 6.70 – 6.47 (m, 3H), 4.88 (d, *J* = 10.1 Hz, 1H), 4.38 (d, *J* = 10.1 Hz, 1H), 4.08 (d, *J* = 12.5 Hz, 1H), 3.87 (d, *J* = 12.1 Hz, 1H), 3.81 (s, 1H). ^{13}C NMR (101 MHz, $CDCl_3$) δ 199.80, 147.88, 138.96, 133.83 (q, *J* = 201.0 Hz), 130.26, 130.11, 129.08, 128.92, 128.20, 128.13, 127.86, 125.86 (q, *J* = 8.0 Hz), 125.13, 117.36, 99.99, 87.31, 63.32, 47.80. ^{19}F NMR (376 MHz, $CDCl_3$) δ -59.68. HRMS: Calcd. for $C_{29}H_{25}F_3NO_2$ $(M+H)^+$: 476.1837; found: 476.1804; HPLC (Chiral IA, λ= 254 nm, hexane/2-propanol = 20/1, Flow rate = 1.0 mL/min), t_{major} = 5.81 min, t_{minor} = 6.68 min.

(*S*)-2-((2-methoxybenzyl)oxy)-1,2-diphenyl-3-(phenylamino)propan-1-one (5c):

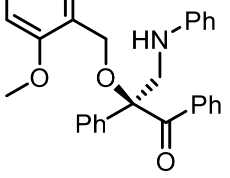

5c

(*S*)-**5c**: White solid, 76% yield, 90% ee; ^1H NMR (400 MHz, $CDCl_3$) δ 8.03 (d, *J* = 7.7 Hz, 2H), 7.59 (d, *J* = 7.6 Hz, 2H), 7.42 (t, *J* = 7.1 Hz, 1H), 7.35 – 7.19 (m, 6H), 7.17 – 7.06 (m, 3H), 6.90 (t, *J* = 7.3 Hz, 1H), 6.82 (d, *J* = 8.0 Hz, 1H), 6.63 (t, *J* = 7.1 Hz, 1H), 6.57 (d, *J* = 7.7 Hz, 2H), 4.76 (d, *J* = 10.4 Hz, 1H), 4.21 (d, *J* = 10.4 Hz, 1H), 4.14 (d, *J* = 12.4 Hz, 1H), 4.00 (s, 1H), 3.84 (dd, *J* = 12.2, 5.4 Hz, 1H), 3.69 (s, 3H). ^{13}C NMR (101 MHz, $CDCl_3$) δ 199.82, 157.25, 148.32, 139.61, 134.71, 132.92, 130.14, 129.60, 129.19, 129.03, 128.64, 128.03, 127.87, 125.60, 125.20, 120.39, 117.07, 112.90, 110.29, 86.69, 61.70, 55.32, 47.49. HRMS: calcd for $C_{29}H_{28}NO_3$ $(M+H)^+$: 438.2069; found: 438.2092; HPLC (Chiral IA, λ= 254 nm, hexane/2-propanol = 40/1, Flow rate = 1.0 mL/min), t_{major} = 9.94 min, t_{minor} = 10.84 min.

(*S*)-2-((2-fluorobenzyl)oxy)-1,2-diphenyl-3-(phenylamino)propan-1-one (5d):

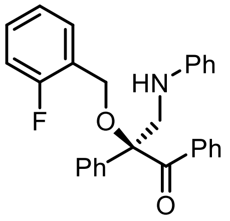

5d

(*S*)-**5d**: White solid, 71% yield, 88% ee; ^1H NMR (400 MHz, $CDCl_3$) δ 7.99 (d, *J* = 7.6 Hz, 2H), 7.58 (d, *J* = 7.6 Hz, 2H), 7.43 (t, *J* = 7.4 Hz, 1H), 7.34 (t, *J* = 7.5 Hz, 2H), 7.30 – 7.22 (m, 4H), 7.15 – 6.96 (m, 5H), 6.65 (t, *J* = 7.3 Hz, 1H), 6.59 (d, *J* = 7.9 Hz, 2H), 4.75 (d, *J* = 10.4 Hz, 1H), 4.26 (d, *J* = 10.4 Hz, 1H), 4.12 (d, *J* = 10.3 Hz, 1H), 3.86 (d, *J* =

11.8 Hz, 2H). ^{13}C NMR (101 MHz, $CDCl_3$) δ 199.56, 160.88 (d, J = 247.9 Hz), 148.07, 139.12, 134.55, 133.12, 130.41 (d, J = 4.1 Hz), 130.09, 129.85 (d, J = 8.2 Hz), 129.11, 128.83, 128.14, 128.11, 125.11, 124.28 (d, J = 14.7 Hz), 124.05 (d, J = 3.6 Hz), 117.36, 115.45, 115.24, 112.93, 98.26, 86.84, 60.79, 47.70. ^{19}F NMR (376 MHz, $CDCl_3$) δ -118.16. HRMS: Calcd for $C_{28}H_{25}FNO_2$ $(M+H)^+$: 426.1869; found: 426.1830; HPLC (Chiral IA, λ= 254 nm, hexane/2-propanol = 40/1, Flow rate = 1.0 mL/min), t_{major} = 8.60 min, t_{minor} = 10.36 min.

(*S*)-2-((2-chlorobenzyl)oxy)-1,2-diphenyl-3-(phenylamino)propan-1-one (5e):

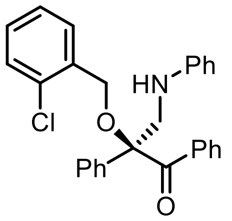

5e

(*S*)-**5e**: White solid, 78% yield, 93% ee; ^{1}H NMR (400 MHz, $CDCl_3$) δ 7.97 (d, J = 7.5 Hz, 2H), 7.61 (d, J = 7.3 Hz, 2H), 7.45 – 7.07 (m, 12H), 6.64 (t, J = 6.9 Hz, 1H), 6.57 (d, J = 7.6 Hz, 2H), 4.80 (d, J = 10.9 Hz, 1H), 4.28 (d, J = 10.8 Hz, 1H), 4.11 (d, J = 11.0 Hz, 1H), 3.87 (d, J = 13.3 Hz, 2H). ^{13}C NMR (101 MHz, $CDCl_3$) δ 199.71, 147.97, 139.12, 134.80, 134.56, 133.49, 133.10, 130.12, 129.95, 129.40, 129.20, 129.11, 128.86, 128.14, 126.76, 125.11, 117.32, 112.87, 86.97, 64.21, 47.74. HRMS: Calcd. for $C_{28}H_{25}ClNO_2$ $(M+H)^+$: 442.1574; found: 442.1600; HPLC (Chiral IA, λ= 254 nm, hexane/2-propanol = 40/1, Flow rate = 1.0 mL/min), t_{major} = 8.16 min, t_{minor} = 10.12 min.

(*S*)-2-((2-bromobenzyl)oxy)-1,2-diphenyl-3-(phenylamino)propan-1-one (5f):

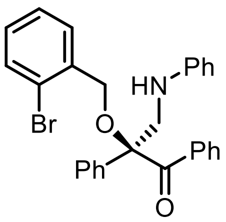

5f

(*S*)-**5f**: White solid, 79% yield, 93% ee; ^{1}H NMR (400 MHz, $CDCl_3$) δ 7.96 (d, J = 7.7 Hz, 2H), 7.61 (d, J = 7.5 Hz, 2H), 7.50 (d, J = 7.8 Hz, 1H), 7.42 (t, J = 7.3 Hz, 1H), 7.35 (t, J = 7.5 Hz, 2H), 7.29 – 7.03 (m, 8H), 6.64 (t, J = 7.2 Hz, 1H), 6.57 (d, J = 7.9 Hz, 2H), 4.78 (d, J = 11.0 Hz, 1H), 4.28 (d, J = 10.9 Hz, 1H), 4.10 (d, J = 9.8 Hz, 1H), 3.95 – 3.84 (m, 2H). ^{13}C NMR (101 MHz, $CDCl_3$) δ 199.76, 147.95, 139.11, 136.47, 134.62, 133.06, 132.66, 130.14, 130.10, 129.41, 129.09, 128.85, 128.13, 127.35, 125.15, 123.40, 117.31, 112.87, 87.07, 66.51, 47.80. HRMS: Calcd. for $C_{28}H_{25}BrNO_2$ $(M+H)^+$: 486.1069; found: 486.1078; HPLC (Chiral IA, λ= 254 nm, hexane/2-propanol = 40/1, Flow rate = 1.0 mL/min), t_{major} = 7.91 min, t_{minor} = 9.38 min.

(*S*)-2-((2-iodobenzyl)oxy)-1,2-diphenyl-3-(phenylamino)propan-1-one (5g):

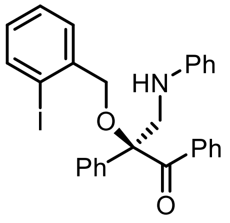

5g

(*S*)-**5g**: White solid, 77% yield, 95% ee; ^{1}H NMR (400 MHz, $CDCl_3$) δ 7.95 (d, *J* = 7.7 Hz, 2H), 7.79 (d, *J* = 7.8 Hz, 1H), 7.62 (d, *J* = 7.6 Hz, 2H), 7.42 (t, *J* = 7.3 Hz, 1H), 7.36 (t, *J* = 7.5 Hz, 2H), 7.29 – 7.22 (m, 4H), 7.19 (d, *J* = 14.9 Hz, 1H), 7.11 (t, *J* = 7.7 Hz, 2H), 6.93 (dd, *J* = 18.5, 7.6 Hz, 2H), 6.64 (t, *J* = 7.2 Hz, 1H), 6.58 (d, *J* = 8.0 Hz, 2H), 4.70 (d, *J* = 10.8 Hz, 1H), 4.25 (d, *J* = 10.8 Hz, 1H), 4.08 (d, *J* = 12.4 Hz, 1H), 3.97 (s, 1H), 3.89 (dd, *J* = 12.5, 5.6 Hz, 1H). ^{13}C NMR (101 MHz, $CDCl_3$) δ 199.90, 147.89, 139.54, 139.31, 139.08, 134.57, 133.09, 130.22, 129.79, 129.61, 129.09, 128.86, 128.15, 125.16, 117.27, 112.88, 98.58, 87.14, 70.88, 47.81. HRMS: Calcd. for $C_{28}H_{25}INO_2$ $(M+H)^+$: 534.0930; found: 534.0953; HPLC (Chiral IA, λ= 254 nm, hexane/2-propanol = 40/1, Flow rate = 1.0 mL/min), t_{major} = 8.52 min, t_{minor} = 9.92 min.

(*S*)-2-((2-methylbenzyl)oxy)-1,2-diphenyl-3-(phenylamino)propan-1-one (5h):

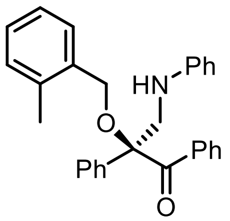

5h

(*S*)-**5h**: White solid, 70% yield, 95% ee; ^{1}H NMR (400 MHz, CDCl3) δ 7.98 (d, *J* = 7.8 Hz, 2H), 7.60 (d, *J* = 7.6 Hz, 2H), 7.43 (t, *J* = 7.2 Hz, 1H), 7.35 (t, *J* = 7.3 Hz, 2H), 7.29 – 7.23 (m, 4H), 7.20 – 7.05 (m, 5H), 6.96 (d, *J* = 7.4 Hz, 1H), 6.65 (t, *J* = 7.2 Hz, 1H), 6.57 (d, *J* = 7.7 Hz, 2H), 4.68 (d, *J* = 10.3 Hz, 1H), 4.22 (s, 1H), 4.16 (dd, *J* = 12.3, 1.7 Hz, 1H), 3.83 (dd, *J* = 13.0, 2.9 Hz, 1H), 3.74 (s, 1H), 2.19 (s, 3H). ^{13}C NMR (101 MHz, $CDCl_3$) δ 199.96, 147.99, 139.31, 136.60, 135.26, 134.60, 133.11, 130.18, 130.15, 129.15, 128.93, 128.89, 128.14, 128.11, 128.03, 125.84, 125.09, 117.33, 112.81, 86.71, 64.83, 47.71, 18.80. HRMS: Calcd. for $C_{29}H_{28}NO_2$ $(M+H)^+$: 422.2120; found: 422.2141; HPLC (Chiral IA, λ= 254 nm, hexane/2-propanol = 80/1, Flow rate = 1.0 mL/min), t_{major} = 8.19 min, t_{minor} = 9.72 min.

(*S*)-2-((2-nitrobenzyl)oxy)-1,2-diphenyl-3-(phenylamino)propan-1-one (5i):

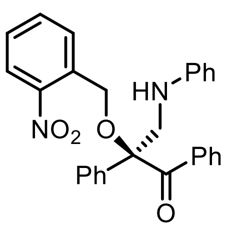

5i

(*S*)-**5i**: White solid, 72% yield, 92% ee; ^{1}H NMR (400 MHz, $CDCl_3$) δ 7.98 (d, *J* = 7.8 Hz, 1H), 7.88 (d, *J* = 7.4 Hz, 2H), 7.59 (d, *J* = 7.2 Hz, 2H), 7.55 – 7.46 (m, 2H), 7.41 – 7.17 (m, 7H), 7.09 (t, *J* = 7.0 Hz, 2H), 6.65 – 6.55 (m, 3H), 5.12 (d, *J* = 13.1 Hz, 1H), 4.57 (d, *J* = 13.0 Hz, 1H), 4.09 (d, *J* = 11.9 Hz, 1H), 3.87 (d, *J* = 15.8 Hz, 2H). ^{13}C NMR (101 MHz, $CDCl_3$) δ 199.55, 147.83, 147.50, 138.76, 134.57, 133.39, 133.24, 133.04, 129.91, 129.36, 129.08, 129.03, 128.42, 128.33, 128.15, 125.11, 124.76, 117.48, 113.01, 87.47, 64.00, 48.07. HRMS: Calcd. for $C_{28}H_{25}N_2O_4$ $(M+H)^+$: 453.1814; found: 453.1803; HPLC (Chiral IA, λ= 254 nm, hexane/2-propanol = 40/1, Flow rate = 1.0 mL/min), t_{major}

= 15.69 min, t_{minor} = 18.41 min.

(*S*)-2-(naphthalen-1-ylmethoxy)-1,2-diphenyl-3-(phenylamino)propan-1-one (5j):

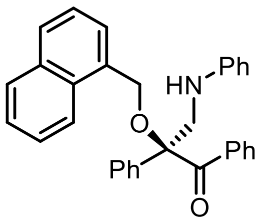

5j

(*S*)-**5j**: White solid, 75% yield, 94% ee; ^{1}H NMR (400 MHz, $CDCl_3$) δ 8.00 (d, *J* = 7.7 Hz, 2H), 7.91 (d, *J* = 8.3 Hz, 1H), 7.84 (d, *J* = 8.1 Hz, 1H), 7.77 (d, *J* = 8.2 Hz, 1H), 7.57 (d, *J* = 7.3 Hz, 2H), 7.50 – 7.39 (m, 3H), 7.35 – 7.21 (m, 6H), 7.15 (t, *J* = 7.8 Hz, 2H), 7.07 (d, *J* = 6.9 Hz, 1H), 6.68 (t, *J* = 7.3 Hz, 1H), 6.61 (d, *J* = 7.8 Hz, 2H), 5.14 (d, *J* = 10.3 Hz, 1H), 4.62 (d, *J* = 10.3 Hz, 1H), 4.29 (d, *J* = 10.7 Hz, 1H), 3.87 (d, *J* = 12.7 Hz, 1H), 3.76 (s, 1H). ^{13}C NMR (101 MHz, $CDCl_3$) δ 199.95, 147.96, 139.26, 134.67, 133.64, 133.12, 132.93, 131.81, 130.24, 129.20, 128.92, 128.83, 128.52, 128.18, 128.13, 126.98, 126.37, 125.78, 125.23, 125.12, 123.96, 117.48, 112.93, 86.94, 65.02, 47.78. HRMS: Calcd. for $C_{32}H_{28}NO_2$ $(M+H)^+$: 458.2120; found: 458.2108; HPLC (Chiral IA, λ= 254 nm, hexane/2-propanol = 40/1, Flow rate = 1.0 mL/min), t_{major} = 7.46 min, t_{minor} = 8.86 min.

(*S*)-2-((4-methoxybenzyl)oxy)-1,2-diphenyl-3-(phenylamino)propan-1-one (5k):

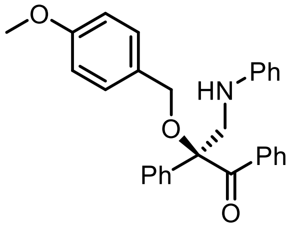

5k

(*S*)-**5k**: Colorless oil, 66% yield, 77% ee; ^{1}H NMR (400 MHz, $CDCl_3$) δ 7.98 – 7.91 (m, 2H), 7.51 (dd, *J* = 7.1, 1.1 Hz, 2H), 7.38 (t, *J* = 6.7 Hz, 1H), 7.30 – 7.15 (m, 5H), 7.06 (t, *J* = 7.5 Hz, 2H), 6.93 – 6.88 (m, 2H), 6.71 (dd, *J* = 4.7, 3.8 Hz, 2H), 6.63 – 6.49 (m, 3H), 4.44 (d, *J* = 9.8 Hz, 1H), 4.08 (dd, *J* = 34.7, 11.2 Hz, 2H), 3.77 – 3.68 (m, 5H). ^{13}C NMR (101 MHz, $CDCl_3$) δ 199.90, 159.40, 148.13, 139.26, 134.66, 133.14, 130.17, 129.82, 129.26, 129.17, 128.85, 128.19, 128.09, 125.14, 117.35, 113.77, 112.91, 86.61, 66.53, 55.27, 47.74. HRMS: Calcd. for $C_{29}H_{28}NO_3$ (M+H)+: 438.2069; found: 438.2081; HPLC (Chiral IA, λ= 254 nm, hexane/2-propanol = 40/1, Flow rate = 1.0 mL/min), t_{major} = 11.18 min, t_{minor} = 12.72 min.

(*S*)-2-((4-bromobenzyl)oxy)-1,2-diphenyl-3-(phenylamino)propan-1-one (5l):

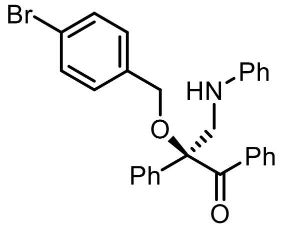

5l

(*S*)-**5l**: Colorless oil, 69% yield, 79% ee; ^{1}H NMR (400 MHz, $CDCl_3$) δ 7.89 (d, *J* = 8.1 Hz, 2H), 7.51 (d, *J* = 8.3 Hz, 2H), 7.39 – 7.15 (m, 8H), 7.06 (t, *J* = 7.4 Hz, 2H), 6.86 (d, *J* = 8.1 Hz, 2H), 6.60 (t, *J* = 7.0 Hz, 1H), 6.51 (d, *J* = 8.3 Hz, 2H), 4.49 (d, *J* = 10.3 Hz, 1H), 4.08 (dd, *J* = 36.1, 11.5 Hz, 2H), 3.73 (d, *J* = 12.8 Hz, 1H), 3.64 (s, 1H). ^{13}C NMR (101 MHz, $CDCl_3$) δ 199.68, 147.98, 138.95, 136.09, 134.57,

133.23, 131.51, 130.09, 129.78, 129.22, 129.00, 128.27, 128.24, 125.09, 121.94, 117.56, 112.97, 86.81, 66.21, 47.86. HRMS: Calcd. for $C_{28}H_{25}BrNO_2$ (M+H)+: 486.1069; found: 486.1078; HPLC (Chiral IA, λ= 254 nm, hexane/2-propanol = 40/1, Flow rate = 1.0 mL/min), t_{major} = 9.96 min, t_{minor} = 12.64 min.

(*S*)-2-(naphthalen-2-ylmethoxy)-1,2-diphenyl-3-(phenylamino)propan-1-one (5m):

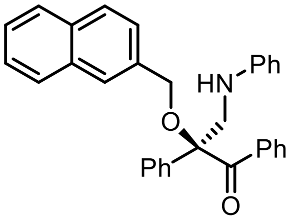

5m

(*S*)-**5m**: Colorless oil, 70% yield, 82% ee; ^{1}H NMR (400 MHz, $CDCl_3$) δ 7.96 (d, *J* = 7.9 Hz, 1H), 7.73 (dd, *J* = 5.7, 3.1 Hz, 1H), 7.70 – 7.61 (m, 1H), 7.55 (d, *J* = 7.0 Hz, 1H), 7.46 – 7.36 (m, 2H), 7.32 – 7.16 (m, 3H), 7.14 – 7.00 (m, 2H), 6.64 – 6.49 (m, 2H), 4.69 (d, *J* = 10.1 Hz, 1H), 4.35 (d, *J* = 10.1 Hz, 1H), 4.11 (d, *J* = 12.7 Hz, 1H), 3.78 (d, *J* = 12.8 Hz, 1H). ^{13}C NMR (101 MHz, $CDCl_3$) δ 199.89, 148.10, 139.16, 134.72, 134.51, 133.19, 133.00, 130.21, 129.20, 128.93, 128.24, 128.17, 128.09, 127.95, 127.68, 127.22, 126.16, 126.12, 125.16, 117.45, 112.98, 86.86, 67.08, 47.86. HRMS: Calcd. for $C_{32}H_{28}NO_2$ (M+H)+: 458.2120; found: 458.2131; HPLC (Chiral IA, λ= 254 nm, hexane/2-propanol = 40/1, Flow rate = 1.0 mL/min), t_{major} = 9.56 min, t_{minor} = 12.32 min.

(*S*)-2-(furan-2-ylmethoxy)-1,2-diphenyl-3-(phenylamino)propan-1-one (5n):

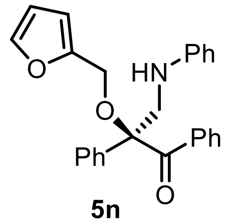

5n

(*S*)-**5n**: Colorless oil, 58% yield, 83% ee; ^{1}H NMR (400 MHz, $CDCl_3$) δ 7.97 – 7.91 (m, 2H), 7.50 (dd, *J* = 7.2, 1.2 Hz, 2H), 7.37 (td, *J* = 7.2, 1.1 Hz, 1H), 7.29 – 7.14 (m, 6H), 7.06 (dd, *J* = 9.7, 4.3 Hz, 2H), 6.58 (ddd, *J* = 22.6, 14.6, 4.2 Hz, 3H), 6.22 – 6.16 (m, 1H), 5.97 (d, *J* = 3.0 Hz, 1H), 4.52 (d, *J* = 11.8 Hz, 1H), 4.22 (d, *J* = 11.8 Hz, 1H), 4.00 (d, *J* = 12.3 Hz, 1H), 3.76 (d, *J* = 14.1 Hz, 2H). ^{13}C NMR (101 MHz, $CDCl_3$) δ 199.32, 150.67, 148.10, 142.92, 138.95, 134.46, 133.09, 130.23, 129.12, 128.81, 128.14, 125.17, 117.39, 112.98, 110.37, 109.64, 86.86, 59.24, 47.88. HRMS: Calcd. for $C_{26}H_{24}NO_3$ (M+H)+: 398.1765; found: 398.1773; HPLC (Chiral IA, λ= 254 nm, hexane/2-propanol = 40/1, Flow rate = 1.0 mL/min), t_{major} = 8.72 min, t_{minor} = 9.47 min.

(*S*)-3-((2-fluorophenyl)amino)-2-(naphthalen-1-ylmethoxy)-1,2-diphenylpropan-1-one (5o):

(*S*)-**5o**: White solid, 65% yield, 95% ee; ^{1}H NMR (400 MHz, $CDCl_3$) δ 8.00 (d, *J* = 7.8 Hz, 2H), 7.96 (d, *J* = 8.1 Hz, 1H), 7.84 (d, *J* = 8.0 Hz, 1H), 7.77 (d, *J* = 8.3 Hz, 1H), 7.57 (d, *J*

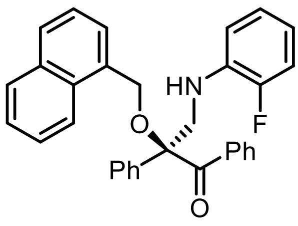

5o

= 8.3 Hz, 2H), 7.51 – 7.40 (m, 3H), 7.35 – 7.21 (m, 6H), 7.03 – 6.88 (m, 3H), 6.77 (t, *J* = 8.4 Hz, 1H), 6.58 (dd, *J* = 12.7, 7.5 Hz, 1H), 5.16 (d, *J* = 10.2 Hz, 1H), 4.59 (d, *J* = 10.2 Hz, 1H), 4.29 (dd, *J* = 12.8, 5.2 Hz, 1H), 4.12 (s, 1H), 3.89 (dd, *J* = 12.7, 4.5 Hz, 1H). ^{13}C NMR (101 MHz, $CDCl_3$) δ 200.00, 151.64 (d, *J* = 238.8 Hz), 139.11, 136.50 (d, *J* = 11.4 Hz), 134.49, 133.63, 133.21, 132.71, 131.88, 130.32, 128.95, 128.87, 128.47, 128.19, 127.01, 126.39, 125.82, 125.18, 125.01, 124.59 (d, *J* = 3.5 Hz), 123.98, 116.66 (d, *J* = 7.0 Hz), 114.29 (d, *J* = 18.2 Hz), 112.11 (d, *J* = 3.2 Hz), 86.83, 65.25, 47.45. ^{19}F NMR (376 MHz, $CDCl_3$) δ -136.86. HRMS: Calcd. for $C_{32}H_{27}FNO_2$ $(M+H)^+$: 476.2026; found: 476.2044; HPLC (Chiral IA, λ= 254 nm, hexane/2-propanol = 80/1, Flow rate = 1.0 mL/min), t_{major} = 8.43 min, t_{minor} = 10.32 min.

(*S*)-3-((4-fluorophenyl)amino)-2-(naphthalen-1-ylmethoxy)-1,2-diphenylpropan-1-one (5p):

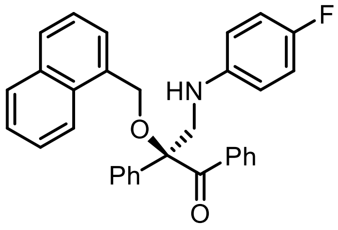

5p

(*S*)-**5p**: White solid, 81% yield, 97% ee; ^{1}H NMR (400 MHz, $CDCl_3$) δ 8.01 (d, *J* = 7.8 Hz, 2H), 7.93 (d, *J* = 8.4 Hz, 1H), 7.85 (d, *J* = 8.1 Hz, 1H), 7.77 (d, *J* = 8.3 Hz, 1H), 7.59 – 7.54 (m, 2H), 7.46 (td, *J* = 15.1, 7.5 Hz, 3H), 7.36 – 7.22 (m, 6H), 7.09 (d, *J* = 6.9 Hz, 1H), 6.83 (t, *J* = 8.7 Hz, 2H), 6.47 (dd, *J* = 8.8, 4.3 Hz, 2H), 5.10 (d, *J* = 10.5 Hz, 1H), 4.65 (d, *J* = 10.5 Hz, 1H), 4.20 (d, *J* = 12.6 Hz, 1H), 3.83 (d, *J* = 12.6 Hz, 1H), 3.67 (s, 1H). ^{13}C NMR (101 MHz, $CDCl_3$) δ 199.85, 156.07 (d, *J* = 190.8 Hz), 144.33 (d, *J* = 1.8 Hz), 139.21, 134.63, 133.66, 133.16, 132.92, 131.76, 130.22, 128.92, 128.87, 128.58, 128.20, 126.88, 126.37, 125.81, 125.23, 125.10, 123.88, 115.52 (d, *J* = 22.3 Hz), 113.67 (d, *J* = 7.3 Hz), 87.04, 65.16, 48.59. ^{19}F NMR (376 MHz, $CDCl_3$) δ -128.11. HRMS: Calcd. for $C_{32}H_{27}FNO_2$ $(M+H)^+$: 476.2026; found: 476.2044; HPLC (Chiral IA, λ= 254 nm, hexane/2-propanol = 80/1, Flow rate = 1.0 mL/min), t_{major} = 11.00 min, t_{minor} = 13.22 min.

(*S*)-3-((4-chlorophenyl)amino)-2-(naphthalen-1-ylmethoxy)-1,2-diphenylpropan-1-one (5q):

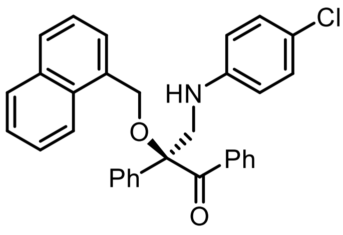

5q

(*S*)-**5q**: White solid, 58% yield, 97% ee; ^{1}H NMR (400 MHz, $CDCl_3$) δ 8.01 (d, *J* = 8.0 Hz, 2H), 7.90 (d, *J* = 8.4 Hz, 1H), 7.84 (d, *J* = 8.1 Hz, 1H), 7.77 (d, *J* = 8.3 Hz, 1H), 7.55 (d, *J* = 7.7 Hz, 2H), 7.45 (ddd, *J* = 15.1, 13.2, 7.4 Hz, 3H), 7.35 – 7.23 (m, 6H), 7.11 – 7.01 (m, 3H), 6.45 (d, *J* = 8.6 Hz, 2H),

5.07 (d, J = 10.5 Hz, 1H), 4.65 (d, J = 10.5 Hz, 1H), 4.20 (dd, J = 12.7, 5.2 Hz, 1H), 3.83 (dd, J = 12.7, 5.0 Hz, 1H), 3.72 (s, 1H). ^{13}C NMR (101 MHz, $CDCl_3$) δ 199.72, 146.51, 139.11, 134.55, 133.66, 133.23, 132.83, 131.73, 130.23, 128.96, 128.94, 128.61, 128.23, 126.92, 126.41, 125.84, 125.24, 125.06, 123.80, 122.01, 113.95, 86.93, 65.18, 48.04. HRMS: Calcd. for $C_{32}H_{27}ClNO_2$ $(M+H)^+$: 492.1730; found: 492.1721; HPLC (Chiral IA, λ= 254 nm, hexane/2-propanol = 40/1, Flow rate = 1.0 mL/min), t_{major} = 10.83 min, t_{minor} = 13.61 min.

(*S*)-2-(naphthalen-1-ylmethoxy)-1,2-diphenyl-3-((4-(trifluoromethyl)phenyl)amino) propan-1-one (5r):

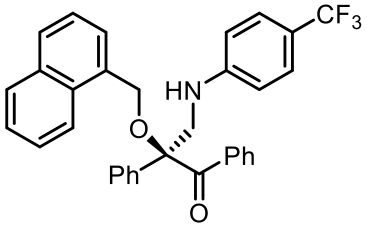

5r

(*S*)-**5r**: White solid, 95% yield, 92% ee; ^{1}H NMR (400 MHz, $CDCl_3$) δ 8.06 – 8.00 (m, 2H), 7.87 (dd, J = 16.1, 8.2 Hz, 2H), 7.77 (d, J = 8.2 Hz, 1H), 7.56 (d, J = 8.0 Hz, 2H), 7.46 (td, J = 16.1, 7.4 Hz, 3H), 7.36 – 7.24 (m, 8H), 7.10 (d, J = 6.9 Hz, 1H), 6.51 (d, J = 8.3 Hz, 2H), 5.06 (d, J = 10.6 Hz, 1H), 4.69 (d, J = 10.6 Hz, 1H), 4.26 (d, J = 12.7 Hz, 1H), 4.03 (s, 1H), 3.89 (d, J = 12.7 Hz, 1H). ^{13}C NMR (101 MHz, $CDCl_3$) δ 199.51, 150.29, 138.97, 134.43, 133.68, 133.35, 132.70, 131.68, 130.25, 129.04, 129.00, 128.68, 128.33, 128.29, 126.91, 126.55, 126.51, 126.47, 126.44, 125.87, 125.24, 125.01, 123.67, 119.12, 118.79, 111.98, 86.83, 65.28, 47.50. ^{19}F NMR (376 MHz, CDCl3) δ -61.01. HRMS: Calcd. for $C_{33}H_{27}F_3NO_2$ $(M+H)^+$: 526.1994; found: 526.2015; HPLC (Chiral IA, λ= 254 nm, hexane/2-propanol = 40/1, Flow rate = 1.0 mL/min), t_{major} = 10.38 min, t_{minor} = 12.63 min.

Ethyl (*S*)-4-((2-(naphthalen-1-ylmethoxy)-3-oxo-2,3-diphenylpropyl)amino)benzoate (5s):

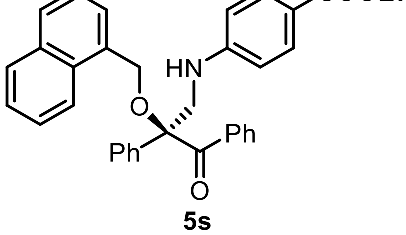

5s

(*S*)-**5s**: White solid, 42% yield, 93% ee; ^{1}H NMR (400 MHz, $CDCl_3$) δ 8.02 (d, J = 8.0 Hz, 2H), 7.90 – 7.75 (m, 5H), 7.56 (d, J = 8.2 Hz, 2H), 7.45 (qd, J = 15.4, 7.4 Hz, 3H), 7.36 – 7.23 (m, 6H), 7.09 (d, J = 6.9 Hz, 1H), 6.48 (d, J = 8.6 Hz, 2H), 5.06 (d, J = 10.5 Hz, 1H), 4.68 (d, J = 10.5 Hz, 1H), 4.30 (dd, J = 13.5, 6.3 Hz, 3H), 4.14 (s, 1H), 3.92 (dd, J = 12.9, 4.7 Hz, 1H), 1.35 (t, J = 7.1 Hz, 3H). ^{13}C NMR (101 MHz, $CDCl_3$) δ 199.50, 166.81, 151.50, 138.92, 134.42, 133.67, 133.35, 132.67, 131.70, 131.41, 130.25, 129.04, 129.01, 128.65, 128.33, 128.28, 126.98, 126.48, 125.87, 125.24, 125.02, 123.68, 118.97, 111.63, 99.99, 86.77, 65.27, 60.19, 47.35, 14.46. HRMS: Calcd. for $C_{35}H_{32}NO_4$ $(M+H)^+$: 530.2331;

found: 530.2340; HPLC (Chiral IA, λ= 254 nm, hexane/2-propanol = 10/1, Flow rate = 1.0 mL/min), t_{major} = 14.96 min, t_{minor} = 17.45 min.

(*S*)-3-((4-methoxyphenyl)amino)-2-(naphthalen-1-ylmethoxy)-1,2-diphenylpropan-1-one (5t):

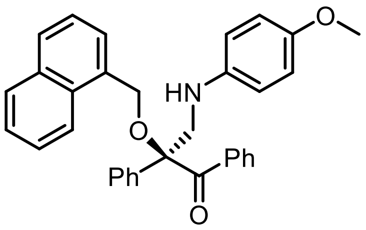

5t

(*S*)-**5t**: White solid, 84% yield, 95% ee; ^{1}H NMR (400 MHz, $CDCl_3$) δ 8.00 (d, *J* = 7.6 Hz, 2H), 7.94 (d, *J* = 8.3 Hz, 1H), 7.84 (d, *J* = 8.1 Hz, 1H), 7.77 (d, *J* = 8.2 Hz, 1H), 7.57 (d, *J* = 8.0 Hz, 2H), 7.45 (dq, *J* = 14.3, 7.1 Hz, 3H), 7.35 – 7.22 (m, 6H), 7.08 (d, *J* = 6.9 Hz, 1H), 6.75 (d, *J* = 8.7 Hz, 2H), 6.55 (d, *J* = 8.7 Hz, 2H), 5.14 (d, *J* = 10.4 Hz, 1H), 4.61 (d, *J* = 10.3 Hz, 1H), 4.23 (d, *J* = 12.6 Hz, 1H), 3.84 (d, *J* = 12.7 Hz, 1H), 3.72 (s, 3H). ^{13}C NMR (101 MHz, $CDCl_3$) δ 200.09, 152.16, 142.31, 139.32, 134.74, 133.63, 133.07, 133.02, 131.81, 130.22, 128.88, 128.79, 128.50, 128.16, 128.09, 126.91, 126.34, 125.77, 125.22, 125.15, 124.03, 114.86, 114.16, 87.08, 65.03, 55.86, 48.79. HRMS: Calcd. for $C_{33}H_{30}NO_3$ $(M+H)^+$: 488.2226; found: 488.2224; HPLC (Chiral IA, λ= 254 nm, hexane/2-propanol = 40/1, Flow rate = 1.0 mL/min), t_{major} = 13.30 min, t_{minor} = 20.30 min.

(*S*)-2-(naphthalen-1-ylmethoxy)-1,2-diphenyl-3-(*p*-tolylamino)propan-1-one (5n):

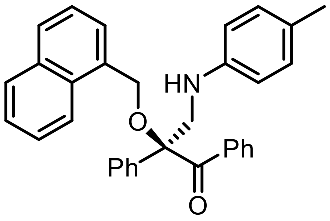

5u

(*S*)-**5u**: White solid, 68% yield, 95% ee; ^{1}H NMR (400 MHz, $CDCl_3$) δ 7.99 (d, *J* = 8.0 Hz, 2H), 7.92 (d, *J* = 8.2 Hz, 1H), 7.84 (d, *J* = 8.1 Hz, 1H), 7.77 (d, *J* = 8.2 Hz, 1H), 7.57 (dd, *J* = 7.2, 1.1 Hz, 2H), 7.50 – 7.39 (m, 3H), 7.28 (ddd, *J* = 13.4, 10.3, 5.1 Hz, 6H), 7.06 (d, *J* = 6.9 Hz, 1H), 6.99 – 6.95 (m, 2H), 6.58 – 6.53 (m, 2H), 5.15 (d, *J* = 10.3 Hz, 1H), 4.60 (d, *J* = 10.3 Hz, 1H), 4.27 (d, *J* = 12.5 Hz, 1H), 3.85 (d, *J* = 12.7 Hz, 1H), 3.64 (s, 1H), 2.21 (s, 3H). ^{13}C NMR (101 MHz, $CDCl_3$) δ 200.08, 145.76, 139.30, 134.73, 133.62, 133.08, 132.99, 131.84, 130.24, 129.69, 128.90, 128.80, 128.49, 128.16, 128.10, 126.99, 126.67, 126.34, 125.77, 125.23, 125.14, 124.04, 113.09, 86.98, 64.97, 48.14, 20.37. HRMS: Calcd. for $C_{33}H_{30}NO_2$ $(M+H)^+$: 472.2277; found: 472.2238; HPLC (Chiral IA, λ= 254 nm, hexane/2-propanol = 40/1, Flow rate = 1.0 mL/min), t_{major} = 9.26 min, t_{minor} = 12.48 min.

(*S*)-2-(naphthalen-1-ylmethoxy)-1,2-diphenyl-3-(*m*-tolylamino)propan-1-one (5v):

(*S*)-**5v**: White solid, 74% yield, 92% ee; ^{1}H NMR (400 MHz, $CDCl_3$) δ 7.97 – 7.89 (m, 2H), 7.84 (d, *J* = 8.1 Hz, 1H), 7.77 (d, *J* = 8.2 Hz, 1H), 7.51 – 7.38 (m, 1H), 7.32 (t, *J* =

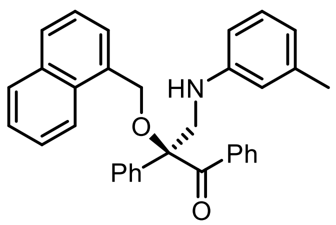

5v

7.6 Hz, 2H), 7.13 (dd, *J* = 16.0, 7.7 Hz, 3H), 7.06 (d, *J* = 7.9 Hz, 6H), 6.67 (d, *J* = 7.2 Hz, 2H), 6.60 (d, *J* = 7.9 Hz, 1H), 5.12 (d, *J* = 10.4 Hz, 2H), 4.63 (d, *J* = 10.4 Hz, 1H), 4.26 (dd, *J* = 12.4, 4.3 Hz, 1H), 3.82 (d, *J* = 12.7 Hz, 1H), 3.76 (s, 1H), 2.32 (s, 1H), 2.27 (s, 3H). ^{13}C NMR (101 MHz, $CDCl_3$) δ 200.03, 147.97, 139.26, 139.02, 134.65, 133.62, 133.14, 132.93, 131.81, 130.25, 129.08, 128.92, 128.82, 128.51, 128.18, 128.13, 127.00, 126.37, 125.79, 125.24, 125.09, 124.00, 118.39, 113.64, 110.15, 86.89, 64.98, 47.77, 21.61. HRMS: Calcd. for $C_{33}H_{30}NO_2$ $(M+H)^+$: 472.2277; found: 472.2238; HPLC (Chiral IA, λ= 254 nm, hexane/2-propanol = 80/1, Flow rate = 1.0 mL/min), t_{major} = 8.20 min, t_{minor} = 11.14 min.

(*R*)-1,2-bis(4-bromophenyl)-2-(naphthalen-1-ylmethoxy)-3-(phenylamino)propan-1-one (5w):

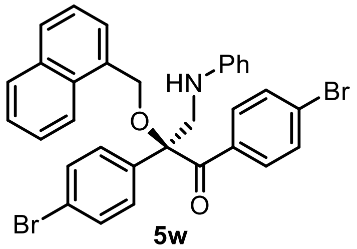

5w

(*S*)-**5w**: White solid, 67% yield, 97% ee; ^{1}H NMR (400 MHz, $CDCl_3$) δ 7.92 – 7.78 (m, 5H), 7.53 – 7.31 (m, 9H), 7.19 – 7.08 (m, 3H), 6.70 (t, *J* = 7.3 Hz, 1H), 6.59 (d, *J* = 7.9 Hz, 2H), 5.16 (d, *J* = 10.5 Hz, 1H), 4.63 (d, *J* = 10.5 Hz, 1H), 4.25 (d, *J* = 12.7 Hz, 1H), 3.82 (d, *J* = 12.8 Hz, 1H), 3.67 (s, 1H). ^{13}C NMR (101 MHz, $CDCl_3$) δ 198.38, 162.12, 147.61, 138.19, 133.68, 132.79, 132.41, 132.18, 131.71, 131.62, 129.29, 129.20, 128.77, 128.69, 127.13, 126.80, 126.53, 125.97, 125.24, 123.75, 122.53, 117.86, 112.99, 86.66, 65.39, 47.59. HRMS: Calcd. for $C_{32}H_{26}Br_2NO_2$ $(M+H)^+$: 614.0330; found: 614.0365; HPLC (Chiral IA, λ= 254 nm, hexane/2-propanol = 40/1, Flow rate = 1.0 mL/min), t_{major} = 11.90 min, t_{minor} = 16.33 min.

(*R*)-2-(naphthalen-1-ylmethoxy)-3-(phenylamino)-1,2-di-p-tolylpropan-1-one (5x):

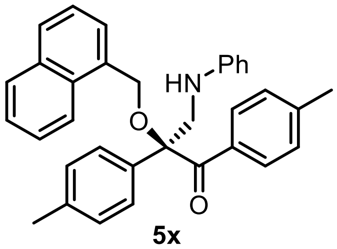

5x

(*S*)-**5x**: White solid, 72% yield, 90% ee; ^{1}H NMR (400 MHz, $CDCl_3$) δ 7.97 – 7.89 (m, 3H), 7.84 (d, *J* = 8.1 Hz, 1H), 7.77 (d, *J* = 8.2 Hz, 1H), 7.51 – 7.38 (m, 4H), 7.32 (t, *J* = 7.6 Hz, 1H), 7.13 (dd, *J* = 16.0, 7.7 Hz, 5H), 7.06 (d, *J* = 7.9 Hz, 2H), 6.67 (d, *J* = 7.2 Hz, 1H), 6.60 (d, *J* = 7.9 Hz, 1H), 5.12 (d, *J* = 10.4 Hz, 1H), 4.63 (d, *J* = 10.4 Hz, 1H), 4.26 (dd, *J* = 12.4, 4.3 Hz, 1H), 3.82 (d, *J* = 12.7 Hz, 1H), 3.76 (s, 1H), 2.32 (s, 3H), 2.27 (s, 3H). ^{13}C NMR (101 MHz, $CDCl_3$) δ 199.42, 148.07, 143.93, 137.73, 136.49, 133.61, 133.13, 132.06, 131.77, 130.41, 129.58, 129.16, 128.86, 128.72, 128.49, 126.88, 126.27, 125.74, 125.24, 124.96, 124.01, 117.34, 112.91, 86.82, 64.86, 47.71, 21.67, 21.07. HRMS: Calcd. for $C_{34}H_{32}NO_2$ $(M+H)^+$: 486.2433;

found: 486.2454; HPLC (Chiral IA, λ= 254 nm, hexane/2-propanol = 40/1, Flow rate = 1.0 mL/min), t_{major} = 9.68 min, t_{minor} = 24.72 min.

(*S*)-2-(benzyloxy)-1-phenyl-3-(phenylamino)propan-1-one (4a):

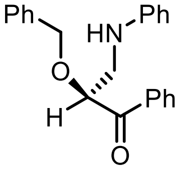

4a

(*S*)-**4a**: White solid, 80% yield, 72% ee; ^{1}H NMR (400 MHz, $CDCl_3$) δ 8.00 (d, *J* = 7.7 Hz, 2H), 7.60 (t, *J* = 7.3 Hz, 1H), 7.47 (t, *J* = 7.6 Hz, 2H), 7.36 – 7.29 (m, 5H), 7.16 (t, *J* = 7.7 Hz, 2H), 6.73 (t, *J* = 7.2 Hz, 1H), 6.59 (d, *J* = 7.9 Hz, 2H), 4.97 (dd, *J* = 7.2, 4.1 Hz, 1H), 4.72 (d, *J* = 11.5 Hz, 1H), 4.44 (d, *J* = 11.5 Hz, 1H), 4.11 (s, 1H), 3.64 (dd, *J* = 13.2, 3.2 Hz, 1H), 3.48 (dd, *J* = 13.3, 7.6 Hz, 1H). ^{13}C NMR (101 MHz, $CDCl_3$) δ 199.14, 147.36, 137.17, 135.36, 133.72, 129.31, 128.79, 128.63, 128.55, 128.20, 128.13, 118.19, 113.57, 80.19, 72.23, 46.35. HRMS: Calcd. for $C_{22}H_{22}NO_2$ $(M+H)^+$: 332.1651; found: 332.1666; HPLC (Chiral IC, λ= 254 nm, hexane/2-propanol = 3/1, Flow rate = 1.0 mL/min), t_{major} = 8.39 min, t_{minor} = 14.57 min.

(*S*)-2-ethoxy-1-phenyl-3-(phenylamino)propan-1-one (4b):

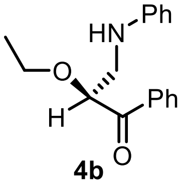

4b

(*S*)-**4b**: Colorless oil, 46% yield, 78% ee; ^{1}H NMR (400 MHz, $CDCl_3$) δ 8.04 (s, 2H), 7.58 (d, *J* = 7.4 Hz, 1H), 7.47 (t, *J* = 7.6 Hz, 2H), 7.18 (t, *J* = 7.8 Hz, 2H), 6.74 (t, *J* = 7.3 Hz, 1H), 6.64 (d, *J* = 7.8 Hz, 2H), 4.85 (dd, *J* = 7.6, 4.2 Hz, 1H), 4.19 (s, 1H), 3.63 (dt, *J* = 12.7, 5.6 Hz, 2H), 3.46 (ddd, *J* = 21.0, 11.0, 7.4 Hz, 2H), 1.22 (t, *J* = 7.0 Hz, 3H). ^{13}C NMR (101 MHz, $CDCl_3$) δ 199.54, 135.33, 133.67, 129.34, 128.76, 128.68, 118.19, 113.59, 81.21, 65.98, 46.27, 15.36. HRMS: Calcd. for $C_{17}H_{20}NO_2$ $(M+H)^+$: 270.1494; found: 270.1505; HPLC (Chiral IC, λ= 254 nm, hexane/2-propanol = 10/1, Flow rate = 1.0 mL/min), t_{major} = 11.08 min, t_{minor} = 17.75 min.

(*S*)-2-isopropoxy-1-phenyl-3-(phenylamino)propan-1-one (4c):

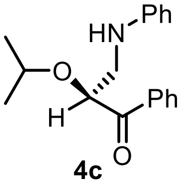

4c

(*S*)-**4c**: Colorless oil, 50% yield, 80% ee; ^{1}H NMR (400 MHz, $CDCl_3$) δ 8.07 (d, *J* = 7.6 Hz, 2H), 7.59 (t, *J* = 7.3 Hz, 1H), 7.48 (t, *J* = 7.6 Hz, 2H), 7.18 (t, *J* = 7.7 Hz, 2H), 6.74 (t, *J* = 7.3 Hz, 1H), 6.65 (d, *J* = 7.8 Hz, 2H), 4.89 (dd, *J* = 8.0, 3.8 Hz, 1H), 4.16 (s, 1H), 3.62 (ddd, *J* = 14.4, 10.4, 4.8 Hz, 2H), 3.38 (dd, *J* = 13.3, 8.2 Hz, 1H), 1.15 (d, *J* = 5.9 Hz, 6H). ^{13}C NMR (101 MHz, $CDCl_3$) δ 200.34, 147.41, 135.33, 133.57, 129.34, 128.82, 128.68, 118.25, 113.75, 79.46, 72.30, 46.88, 22.97, 21.74. HRMS: Calcd. for $C_{18}H_{22}NO_2$ $(M+H)^+$: 284.1651; found: 284.1666; HPLC (Chiral IC, λ= 254 nm, hexane/2-propanol = 10/1, Flow rate = 1.0

mL/min), t_{major} = 10.20 min, t_{minor} = 12.55 min.

(*S*)-2-(benzhydryloxy)-1-phenyl-3-(phenylamino)propan-1-one (4d):

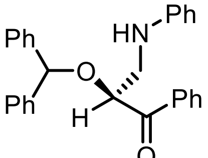

4d

(*S*)-**4f**: Colorless oil, 73% yield, 91% ee; ^{1}H NMR (400 MHz, $CDCl_3$) δ 7.87 – 7.84 (m, 2H), 7.59 – 7.53 (m, 1H), 7.40 (dd, *J* = 10.7, 4.8 Hz, 2H), 7.36 – 7.19 (m, 10H), 7.16 – 7.10 (m, 2H), 6.74 – 6.68 (m, 1H), 6.52 (dd, *J* = 8.5, 0.9 Hz, 2H), 5.49 (s, 1H), 5.00 (dd, *J* = 7.1, 4.2 Hz, 1H), 4.08 (s, 1H), 3.61 (dd, *J* = 13.4, 4.1 Hz, 1H), 3.51 (dd, *J* = 13.4, 7.2 Hz, 1H). ^{13}C NMR (101 MHz, $CDCl_3$) δ 199.21, 147.33, 141.53, 140.57, 135.61, 133.58, 129.26, 128.72, 128.51, 128.24, 128.11, 127.65, 127.61, 127.23, 118.04, 113.38, 82.83, 78.06, 46.62. HRMS: Calcd. for $C_{28}H_{26}NO_2$ $(M+H)^+$: 408.1964; found: 408.1977; HPLC (Chiral IA, λ= 254 nm, hexane/2-propanol = 20/1, Flow rate = 1.0 mL/min), t_{major} = 11.56 min, t_{minor} = 14.10 min.

(*S*)-2-(bis(4-bromophenyl)methoxy)-1-phenyl-3-(phenylamino)propan-1-one (4e):

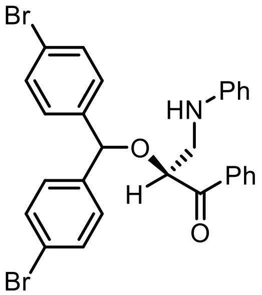

4e

(*S*)-**4e**: White solid, 41% yield, 92% ee; ^{1}H NMR (400 MHz, $CDCl_3$) δ 7.84 (d, *J* = 7.6 Hz, 2H), 7.59 (t, *J* = 7.3 Hz, 1H), 7.47 – 7.08 (m, 12H), 6.74 (t, *J* = 7.3 Hz, 1H), 6.53 (d, *J* = 7.9 Hz, 2H), 5.37 (s, 1H), 4.98 (dd, *J* = 7.0, 3.9 Hz, 1H), 4.01 (s, 1H), 3.62 (dd, *J* = 13.6, 3.5 Hz, 1H), 3.50 (dd, *J* = 13.7, 7.3 Hz, 1H). ^{13}C NMR (101 MHz, $CDCl_3$) δ 198.82, 147.05, 140.05, 139.05, 135.41, 133.83, 131.97, 131.44, 129.35, 129.20, 128.87, 128.38, 122.37, 121.90, 118.30, 113.39, 81.45, 78.03, 46.61. HRMS: Calcd. for $C_{28}H_{24}Br_2NO_2$ $(M+H)^+$: 564.0174; found: 564.0187; HPLC (Chiral IC, λ= 254 nm, hexane/2-propanol = 5/1, Flow rate = 1.0 mL/min), t_{minor} = 10.52 min, t_{major} = 13.40 min.

(*S*)-2-(allyloxy)-1-phenyl-3-(phenylamino)propan-1-one (4f):

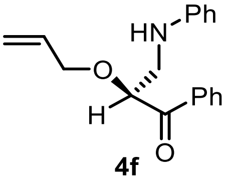

4f

(*S*)-**4f**: Colorless oil, 48% yield, 78% ee; ^{1}H NMR (400 MHz, $CDCl_3$) δ 8.05 – 8.00 (m, 2H), 7.60 (t, *J* = 7.4 Hz, 1H), 7.48 (t, *J* = 7.7 Hz, 2H), 7.18 (dd, *J* = 8.4, 7.4 Hz, 2H), 6.74 (t, *J* = 7.3 Hz, 1H), 6.63 (d, *J* = 7.7 Hz, 2H), 5.89 (ddt, *J* = 16.4, 10.4, 5.8 Hz, 1H), 5.26 (dd, *J* = 17.2, 1.5 Hz, 1H), 5.18 (dd, *J* = 10.3, 1.2 Hz, 1H), 4.95 (dd, *J* = 7.5, 4.1 Hz, 1H), 4.17 (dd, *J* = 12.5, 5.5 Hz, 2H), 3.97 (dd, *J* = 12.5, 6.1 Hz, 1H), 3.62 (dd, *J* = 13.4, 4.1 Hz, 1H), 3.45 (dd, *J* = 13.4, 7.5 Hz, 1H). ^{13}C NMR (101 MHz, $CDCl_3$) δ 199.18, 147.41, 135.36, 133.94, 133.71, 129.33, 128.80, 128.62, 118.24, 118.21, 113.57, 80.17, 71.33, 46.32. HRMS: Calcd. for

$C_{18}H_{20}NO_2$ (M+H)$^+$: 282.1494; found: 282.1487; HPLC (Chiral IA, λ= 254 nm, hexane/2-propanol = 10/1, Flow rate = 1.0 mL/min), t_{major} = 9.87 min, t_{minor} = 11.07 min.

(*S*)-1-phenyl-3-(phenylamino)-2-(prop-2-yn-1-yloxy)propan-1-one (4g):

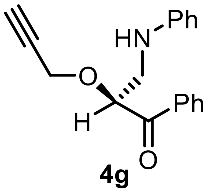

4g

(*S*)-**4g**: Colorless oil, 79% yield, 70% ee; ^{1}H NMR (400 MHz, $CDCl_3$) δ 8.03 – 7.98 (m, 2H), 7.64 – 7.58 (m, 1H), 7.52 – 7.46 (m, 2H), 7.20 – 7.14 (m, 2H), 6.76 – 6.71 (m, 1H), 6.63 (dd, *J* = 8.6, 0.9 Hz, 2H), 5.25 (dd, *J* = 7.3, 3.9 Hz, 1H), 4.40 (dd, *J* = 16.1, 2.4 Hz, 1H), 4.19 (dd, *J* = 16.1, 2.4 Hz, 2H), 3.65 (dd, *J* = 13.5, 3.9 Hz, 1H), 3.44 (dd, *J* = 13.5, 7.3 Hz, 1H), 2.43 (t, *J* = 2.4 Hz, 1H). ^{13}C NMR (101 MHz, $CDCl_3$) δ 198.19, 147.35, 135.29, 133.83, 129.30, 128.87, 128.60, 118.27, 113.65, 78.87, 78.81, 75.92, 57.41, 46.25. HRMS: Calcd. for $C_{18}H_{18}NO_2$ (M+H)$^+$: 280.1338; found: 280.1346; HPLC (Chiral IA, λ= 254 nm, hexane/2-propanol = 10/1, Flow rate = 1.0 mL/min), t_{major} = 13.58 min, t_{minor} = 17.99 min.

(*R,E*)-2-((3,7-dimethylocta-2,6-dien-1-yl)oxy)-1-phenyl-3-(phenylamino)propan-1-one (4h):

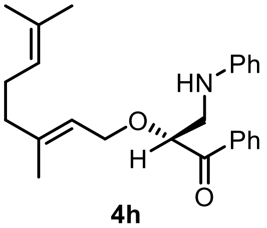

4h

(*S*)-**4h**: Colorless oil, 71% yield, 75% ee; ^{1}H NMR (400 MHz, $CDCl_3$) δ 8.06 – 8.01 (m, 2H), 7.59 (ddd, *J* = 6.9, 2.4, 1.2 Hz, 1H), 7.47 (dd, *J* = 10.6, 4.7 Hz, 2H), 7.21 – 7.14 (m, 2H), 6.73 (t, *J* = 7.3 Hz, 1H), 6.63 (dd, *J* = 8.5, 0.9 Hz, 2H), 5.32 (td, *J* = 7.1, 1.2 Hz, 1H), 5.09 – 5.03 (m, 1H), 4.91 (dd, *J* = 7.7, 4.1 Hz, 1H), 4.15 (dd, *J* = 11.6, 6.8 Hz, 2H), 4.01 (dd, *J* = 11.6, 7.3 Hz, 1H), 3.60 (dd, *J* = 13.3, 4.1 Hz, 1H), 3.42 (dd, *J* = 13.3, 7.7 Hz, 1H), 2.08 – 1.94 (m, 4H), 1.67 (s, 3H), 1.58 (s, 3H), 1.55 (s, 3H). ^{13}C NMR (101 MHz, $CDCl_3$) δ 199.59, 147.48, 141.96, 135.43, 133.60, 131.78, 129.30, 128.73, 128.64, 123.83, 119.92, 118.15, 113.59, 79.84, 66.61, 46.33, 39.53, 26.24, 25.68, 17.70, 16.46. HRMS: Calcd. for $C_{25}H_{32}NO_2$ (M+H)$^+$: 378.2433; found: 378.2456; HPLC (Chiral IA, λ= 254 nm, hexane/2-propanol = 10/1, Flow rate = 1.0 mL/min), t_{mior} = 6.59 min, t_{major} = 7.66 min.

(*S*)-1-phenyl-3-(phenylamino)-2-(((*2E,6E*)-3,7,11-trimethyldodeca-2,6,10-trien-1-yl)oxy)propan-1-one (4i):

(*S*)-**4i**: Colorless oil, 83% yield, 76% ee; ^{1}H NMR (400 MHz, $CDCl_3$) δ 8.07 – 7.99 (m, 2H), 7.62 – 7.56 (m, 1H), 7.47 (t, *J* = 7.7 Hz, 2H), 7.21 – 7.14 (m, 2H), 6.73 (t, *J* = 7.3 Hz, 1H), 6.63 (d, *J* = 7.7 Hz, 2H), 5.33 (t, *J* = 6.6 Hz, 1H), 5.11 – 5.04 (m, 2H), 4.91 (dd, *J* = 7.7, 4.1 Hz, 1H), 4.15 (dd, *J* = 11.6, 6.8 Hz, 2H), 4.01 (dd, *J* = 11.5, 7.3 Hz, 1H), 3.60 (dd,

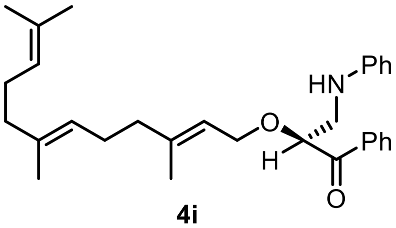

4i

J = 13.3, 4.1 Hz, 1H), 3.42 (dd, J = 13.3, 7.8 Hz, 1H), 2.10 – 1.93 (m, 8H), 1.67 (s, 3H), 1.59 (s, 3H), 1.58 (s, 3H), 1.55 (s, 3H). ^{13}C NMR (101 MHz, $CDCl_3$) δ 199.59, 147.47, 142.02, 135.40, 133.61, 131.35, 129.31, 128.74, 128.65, 124.32, 123.73, 119.92, 118.15, 113.60, 79.85, 66.61, 46.34, 39.71, 39.56, 26.73, 26.19, 25.72, 17.71, 16.49, 16.03. HRMS: Calcd. for $C_{30}H_{40}NO_2$ $(M+H)^+$: 446.3059; found: 446.3074; HPLC (Chiral IA, λ= 254 nm, hexane/2-propanol = 20/1, Flow rate = 1.0 mL/min), t_{minor} = 7.47 min, t_{major} = 8.52 min.

(*S*)-1-phenyl-3-(phenylamino)-2-(((*1S,2R,4S*)-1,7,7-trimethylbicyclo[2.2.1]heptan-2-yl)oxy)propan-1-one (4j):

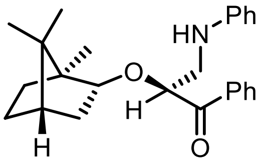

4j

4j: White Solid, 77% yield, >20:1 d.r.; ^{1}H NMR (400 MHz, $CDCl_3$) δ 7.99 (d, J = 7.1 Hz, 2H), 7.51 (t, J = 7.3 Hz, 1H), 7.40 (t, J = 7.6 Hz, 2H), 7.12 (dd, J = 13.2, 6.2 Hz, 2H), 6.68 (t, J = 6.8 Hz, 1H), 6.58 (t, J = 9.0 Hz, 2H), 4.72 (dd, J = 8.4, 3.8 Hz, 1H), 4.07 (s, 1H), 3.62 – 3.49 (m, 2H), 3.42 – 3.32 (m, 1H), 1.98 (dtd, J = 20.1, 14.8, 10.8 Hz, 2H), 1.69 – 1.52 (m, 2H), 1.17 (dd, J = 18.4, 8.7 Hz, 3H), 0.81 – 0.61 (m, 9H). ^{13}C NMR (101 MHz, $CDCl_3$) δ 200.37, 147.38, 135.38, 133.57, 129.39, 129.09, 128.93, 128.64, 118.25, 113.74, 86.82, 84.01, 82.81, 80.17, 49.73, 49.49, 47.85, 46.89, 44.91, 36.35, 35.76, 28.32, 26.90, 26.58, 19.68, 18.82, 14.16, 13.60. HRMS: Calcd. for $C_{25}H_{32}NO_2$ $(M+H)^+$: 378.2433; found: 378.2451.

(S)-2-(((1S,2R,5S)-2-isopropyl-5-methylcyclohexyl)oxy)-1-phenyl-3-(phenylamino) propan-1-one (4k)

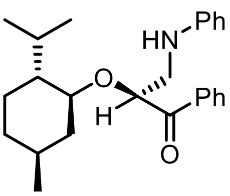

4k

4k: White Solid, 83% yield, >20:1 d.r.; ^{1}H NMR (400 MHz, $CDCl_3$) δ 8.10 – 8.05 (m, 1H), 7.60 (t, J = 7.4 Hz, 1H), 7.48 (t, J = 7.7 Hz, 1H), 7.18 (dd, J = 8.5, 7.4 Hz, 1H), 6.73 (t, J = 7.3 Hz, 1H), 6.59 (d, J = 7.7 Hz, 1H), 4.85 (dd, J = 7.9, 3.9 Hz, 1H), 4.15 (s, 1H), 3.57 (dd, J = 13.4, 3.8 Hz, 1H), 3.39 (dd, J = 13.4, 7.9 Hz, 1H), 3.21 (td, J = 10.6, 4.2 Hz, 1H), 2.40 (dtd, J = 14.0, 7.0, 2.6 Hz, 1H), 1.77 (ddd, J = 4.8, 2.9, 0.8 Hz, 1H), 1.61 (ddd, J = 11.8, 6.1, 2.6 Hz, 1H), 1.45 – 1.36 (m, 1H), 1.34 (s, 1H), 1.27 (d, J = 11.6 Hz, 1H), 0.95 (d, J = 7.0 Hz, 1H), 0.81 (d, J = 7.0 Hz, 2H), 0.77 (d, J = 6.6 Hz, 1H). ^{13}C NMR (101 MHz, $CDCl_3$) δ 200.43, 147.26, 135.23, 133.57, 129.35, 128.92, 128.72, 118.03, 113.30, 81.72, 80.92, 48.40, 46.87, 41.78, 34.19, 31.59, 25.46, 22.83, 22.14, 21.13, 15.98. HRMS: Calcd. for $C_{25}H_{34}NO_2$ $(M+H)^+$: 380.5520; found: 380.5529.

(*S*)-2-(((*3S,8S,9S,10R,13R,14S,17R*)-10,13-dimethyl-17-((*R*)-6-methylheptan-2-yl)-2,3,4,7,8,9,10,11,12,13,14,15,16,17-tetradecahydro-1H-cyclopenta[a]phenanthren-3-yl)oxy)-1-phenyl-3-(phenylamino)propan-1-one (4l):

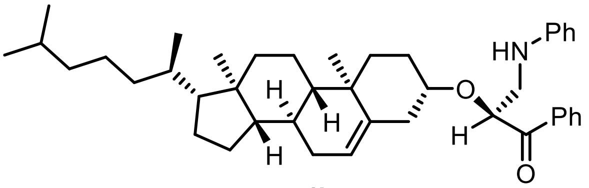

4l

4k: Colorless oil, 82% yield, 85:15 d.r.; ^{1}H NMR (400 MHz, $CDCl_3$) δ 8.05 (d, *J* = 7.7 Hz, 2H), 7.60 (t, *J* = 7.3 Hz, 1H), 7.48 (t, *J* = 7.5 Hz, 2H), 7.19 (t, *J* = 7.7 Hz, 2H), 6.75 (t, *J* = 7.3 Hz, 1H), 6.65 (d, *J* = 7.9 Hz, 2H), 5.26 (dd, *J* = 21.9, 3.9 Hz, 1H), 4.96 (dd, *J* = 7.5, 3.4 Hz, 1H), 4.14 (s, 1H), 3.60 (dd, *J* = 13.1, 3.1 Hz, 1H), 3.38 (dd, *J* = 13.2, 8.1 Hz, 1H), 3.21 (ddd, *J* = 14.6, 10.2, 4.3 Hz, 1H), 2.34 – 2.21 (m, 2H), 2.03 – 1.74 (m, 5H), 1.59 – 0.82 (m, 33H), 0.66 (s, 3H). ^{13}C NMR (101 MHz, $CDCl_3$) δ 200.01, 147.41, 140.32, 135.28, 133.60, 129.34, 128.81, 128.70, 122.06, 118.24, 113.72, 79.72, 79.22, 56.71, 56.12, 50.08, 46.82, 42.30, 39.74, 39.52, 37.02, 36.69, 36.18, 35.77, 31.82, 29.22, 28.21, 28.02, 24.26, 23.81, 22.82, 22.56, 21.04, 19.36, 18.71, 11.85. HRMS: Calcd. for $C_{42}H_{60}NO_2$ $(M+H)^+$: 610.4624; found: 610.4639.

(3R,3aS,6aR)-hexahydrofuro[2,3-b]furan-3-yl ((2S,3R)-4-((4-(((S)-2-(((3S,8S,9S,10R,13R,14S,17R)-10,13-dimethyl-17-((R)-6-methylheptan-2-yl)-2,3,4,7,8,9,10,11,12,13,14,15,16,17-tetradecahydro-1H-cyclopenta[a]phenanthren-3-yl)oxy)-3-oxo-3-phenylpropyl)amino)-N-isobutylphenyl)sulfonamido)-3-hydroxy-1-phenylbutan-2-yl)carbamate (4m):

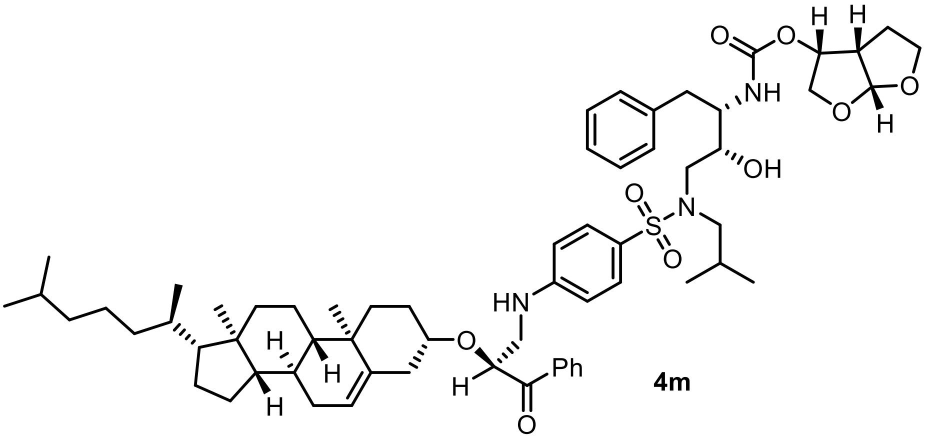

4l: Colorless crystal, ^{1}H NMR (400 MHz, $CDCl_3$) δ 8.09 – 8.03 (m, 2H), 7.65 – 7.46 (m, 5H), 7.25 (dt, *J* = 20.2, 7.3 Hz, 5H), 6.62 (d, *J* = 8.7 Hz, 2H), 5.63 (d, *J* = 5.2 Hz, 1H), 5.27 (dd, *J* = 19.5, 4.9 Hz, 1H), 5.05 – 4.91 (m, 3H), 4.68 (s, 1H), 3.97 – 3.81 (m, 4H), 3.73 – 3.60 (m, 3H), 3.53 – 3.45 (m, 1H), 3.31 – 3.04 (m, 3H), 2.84 (dddd, *J* = 19.7, 17.1, 13.3, 7.4 Hz, 5H), 2.39 – 2.20 (m, 2H), 2.07 – 1.73 (m, 7H), 1.69 – 1.20 (m, 20H), 0.95 – 0.82 (m, 20H), 0.66 (s, 3H).

^{13}C NMR (101 MHz, $CDCl_3$) δ 199.42, 155.45, 151.16, 140.03, 137.72, 134.97, 133.92, 129.48, 129.41, 128.84, 128.52, 126.54, 125.34, 122.40, 122.30, 112.42, 109.31, 79.81, 78.86, 73.38, 72.88, 70.80, 69.63, 58.92, 56.69, 56.10, 55.11, 53.78, 50.07, 45.79, 45.36, 42.29, 39.69, 39.51, 38.90, 37.10, 36.72, 36.17, 35.77, 35.63, 31.81, 31.60, 31.45, 30.19, 29.72, 29.16, 28.22, 28.02, 27.33, 25.83, 24.27, 23.81, 22.84, 22.58, 21.02, 20.21, 19.94, 19.36, 18.71, 14.16, 11.86. HRMS: Calcd. for $C_{63}H_{89}N_3O_9SNa$ $(M+Na)^+$: 1086.6217; found: 1086.6245.

(*S*)-2-hydroxy-1-phenyl-3-(phenylamino)propan-1-one (4n):

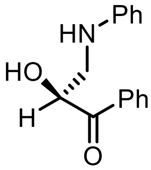

4n

(*S*)-**4m**: Pale yellow oil, 69% yield, 90% ee; ^1H NMR (400 MHz, $CDCl_3$) δ 7.94 (d, J = 7.6 Hz, 2H), 7.65 (t, J = 7.4 Hz, 1H), 7.52 (t, J = 7.7 Hz, 2H), 7.15 (t, J = 7.8 Hz, 2H), 6.73 (t, J = 7.3 Hz, 1H), 6.58 (d, J = 7.9 Hz, 2H), 5.30 (s, 1H), 4.08 (s, 1H), 3.93 (s, 1H), 3.64 (dd, J = 12.9, 3.4 Hz, 1H), 3.26 (dd, J = 12.9, 6.6 Hz, 1H). ^{13}C NMR (101 MHz, $CDCl_3$) δ 200.31, 147.57, 134.35, 133.54, 129.27, 129.05, 128.60, 118.39, 113.75, 72.47, 48.73. HRMS: Calcd. for $C_{15}H_{16}NO_2$ $(M+H)^+$: 242.1181; found: 242.1200; HPLC (Chiral IC, λ= 254 nm, hexane/2-propanol = 3/1, Flow rate = 1.0 mL/min), t_{minor} = 8.47 min, t_{major} = 10.66 min.

2-(naphthalen-1-ylmethoxy)-1,2-diphenylethan-1-one (7):

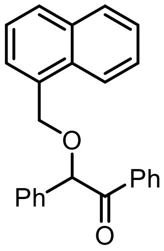

7

7: Colorless oil, 89% yield; ^1H NMR (400 MHz, $CDCl_3$) δ 8.18 – 8.08 (m, 1H), 7.92 – 7.87 (m, 2H), 7.86 – 7.78 (m, 2H), 7.49 – 7.37 (m, 7H), 7.34 – 7.24 (m, 5H), 5.70 (s, 1H), 5.08 (s, 2H). ^{13}C NMR (101 MHz, $CDCl_3$) δ 197.53, 136.32, 135.07, 133.80, 133.20, 132.80, 131.91, 129.21, 129.05, 128.85, 128.53, 128.50, 128.42, 127.68, 127.19, 126.38, 125.93, 125.21, 124.28, 83.81, 70.02. Calcd. for $C_{25}H_{21}O_2$ $(M+H)^+$: 353.4410; found: 353.4414.

phenyl(1,3,4-triphenylimidazolidin-4-yl)methanone (8):

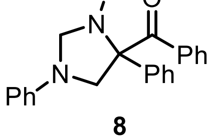

8

8: Pale yellow oil, 71% yield; ^1H NMR (400 MHz, $CDCl_3$) δ 8.03 – 7.95 (m, 4H), 7.66 – 7.57 (m, 3H), 7.52 – 7.42 (m, 4H), 7.38 (t, J = 7.7 Hz, 2H), 7.34 – 7.27 (m, 2H), 7.22 (dd, J = 10.2, 5.3 Hz, 2H), 6.78 (td, J = 7.4, 0.6 Hz, 1H), 6.54 (d, J = 8.5 Hz, 2H), 5.16 (d, J = 1.9 Hz, 1H), 5.03 (d, J = 1.8 Hz, 1H), 4.74 (dd, J = 8.9, 0.9 Hz, 1H), 3.32 (d, J = 8.8 Hz, 1H). ^{13}C NMR (101 MHz, $CDCl_3$) δ 198.03, 194.61, 145.30, 139.01, 134.93, 134.31, 133.05, 132.98, 130.52, 129.95, 129.34, 129.07, 129.00, 128.28, 128.07, 124.63, 118.42, 113.24, 92.14,

82.31, 56.13. Calcd. for $C_{28}H_{25}N_2O$ $(M+H)^+$: 405.5210; found: 405.5218.

2-hydroxy-2-((phenylamino)methyl)-3,4-dihydronaphthalen-1(2H)-one (9):

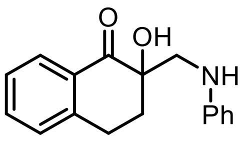

9

9: White solid, 63% yield; ^1H NMR (400 MHz, $CDCl_3$) δ 8.01 (d, *J* = 7.8 Hz, 1H), 7.55 (t, *J* = 7.5 Hz, 1H), 7.36 (t, *J* = 7.5 Hz, 1H), 7.28 (d, *J* = 7.8 Hz, 1H), 7.13 (t, *J* = 7.6 Hz, 2H), 6.70 (t, *J* = 7.3 Hz, 1H), 6.58 (d, *J* = 8.5 Hz, 2H), 4.09 (s, 1H), 4.04 (s, 1H), 3.45 (d, *J* = 12.4 Hz, 1H), 3.26 (d, *J* = 12.5 Hz, 1H), 3.18 – 3.00 (m, 2H), 2.54 (ddd, *J* = 13.5, 4.8, 2.6 Hz, 1H), 2.18 (td, *J* = 12.6, 6.1 Hz, 1H). ^{13}C NMR (101 MHz, $CDCl_3$) δ 200.19, 148.07, 143.38, 134.41, 130.12, 129.24, 129.15, 128.18, 127.09, 118.08, 113.48, 75.91, 48.36, 32.40, 26.34. Calcd. for $C_{17}H_{18}NO_2$ $(M+H)^+$: 268.3360; found: 268.3363.

10. X-ray Diffraction Parameters and Data of 5o and 4e

Supplementary Figure 3. X-ray Diffraction Parameters and Data of 5o (CCDC: 1887265)

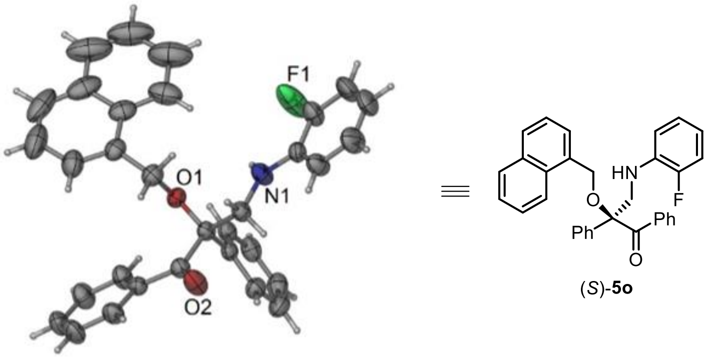

Bond precision:	C-C = 0.0046 A		Wavelength=1.54184
Cell:	a=11.6904(1)	b=14.5130(2)	c=15.0004(2)
	alpha=90	beta=90	gamma=90
Temperature:	293 K		

	Calculated	Reported
Volume	2545.01(5)	2545.01(5)
Space group	P 21 21 21	P 21 21 21
Hall group	P 2ac 2ab	P 2ac 2ab
Moiety formula	C32 H26 F N O2	C32 H26 F N O2
Sum formula	C32 H26 F N O2	C32 H26 F N O2
Mr	475.54	475.54
Dx,g cm-3	1.241	1.241
Z	4	4
Mu (mm-1)	0.656	0.656
F000	1000.0	1000.0
F000'	1002.99	
h,k,lmax	13,17,17	13,17,17
Nref	4536[2568]	4518

Tmin,Tmax	0.754,0.894	0.809,1.000
Tmin'	0.754	

Correction method= # Reported T Limits: Tmin=0.809 Tmax=1.000 AbsCorr = MULTI-SCAN

Data completeness= 1.76/1.00 Theta(max)= 67.050

R(reflections)= 0.0389(4269) wR2(reflections)= 0.1015(4518)

S = 1.029 Npar= 326

Supplementary Figure 4. X-ray Diffraction Parameters and Data of 4e (CCDC: 1887266)

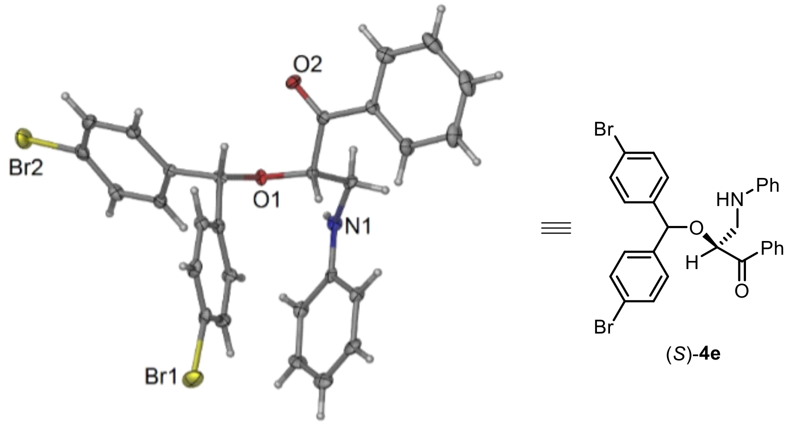

Bond precision: C-C = 0.0085 A Wavelength=1.54184

Cell:	a=12.0517(1)	b=12.6179(1)	c=16.4441(1)
	alpha=90	beta=103.239(1)	gamma=90
Temperature:	100 K		

	Calculated	Reported
Volume	2434.15(3)	2434.15(3)
Space group	P 21	P 1 21 1
Hall group	P 2yb	P 2yb
Moiety formula	C28 H23 Br2 N O2	2(C28 H23 Br2 N O2)
Sum formula	C28 H23 Br2 N O2	C56 H46 Br4 N2 O4
Mr	565.27	1130.59
Dx,g cm-3	1.543	1.543
Z	4	2
Mu (mm-1)	4.423	4.423
F000	1136.0	1136.0
F000'	1133.02	
h,k,lmax	14,15,19	14,15,19
Nref	8715[4574]	8682
Tmin,Tmax	0.166,0.243	0.272,1.000
Tmin'	0.105	

Correction method= # Reported T Limits: Tmin=0.272 Tmax=1.000 AbsCorr = MULTI-SCAN

Data completeness= 1.90/1.00 Theta(max)= 67.061

R(reflections)= 0.0405(8476) wR2(reflections)= 0.1051(8682)

S = 1.031 Npar= 595

11. NMR Spectra of Compounds

Supplementary Figure 5. ^{1}H NMR spectrum of (*S*)-**5a** (400 MHz, $CDCl_3$)

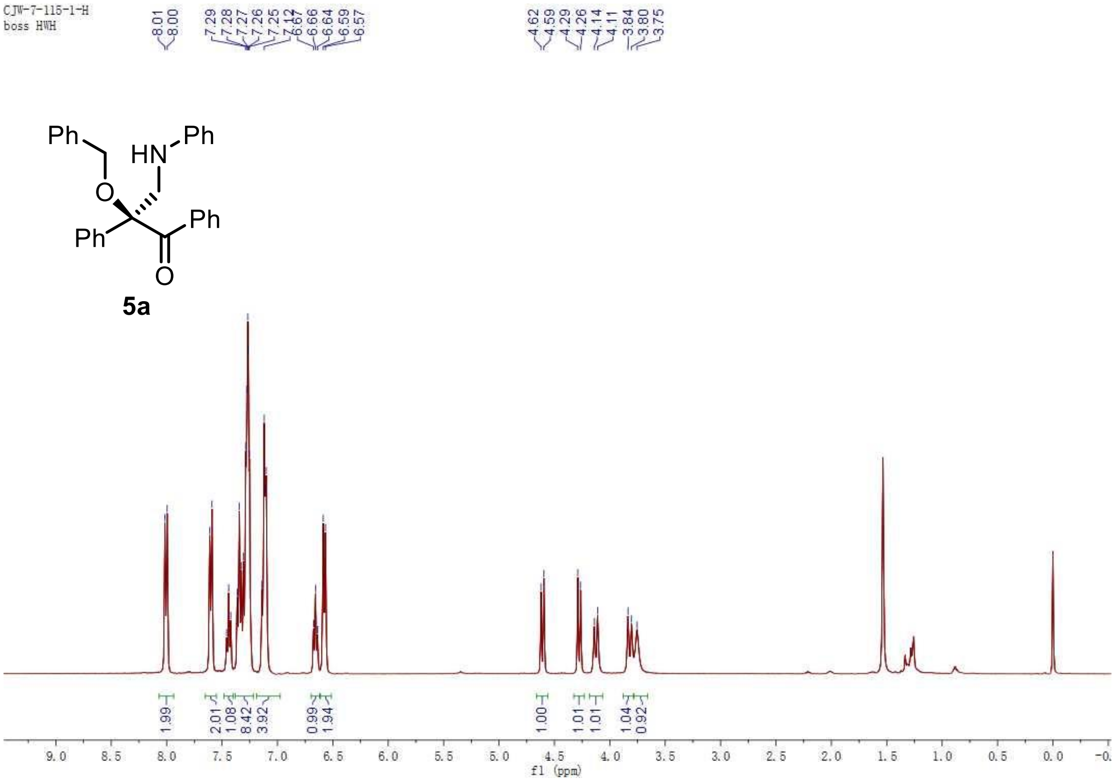

Supplementary Figure 6. ^{13}C NMR spectrum of (*S*)-**5a** (100 MHz, $CDCl_3$)

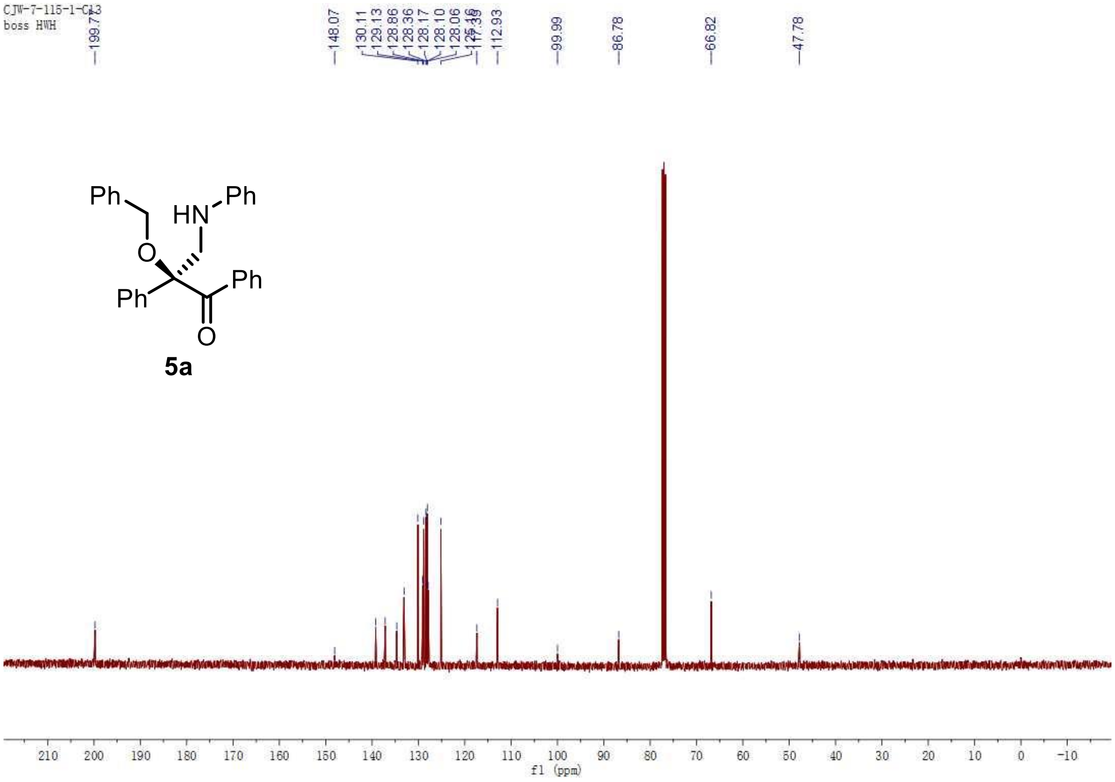

Supplementary Figure 7. ^{1}H NMR spectrum of (*S*)-**5b** (400 MHz, $CDCl_3$)

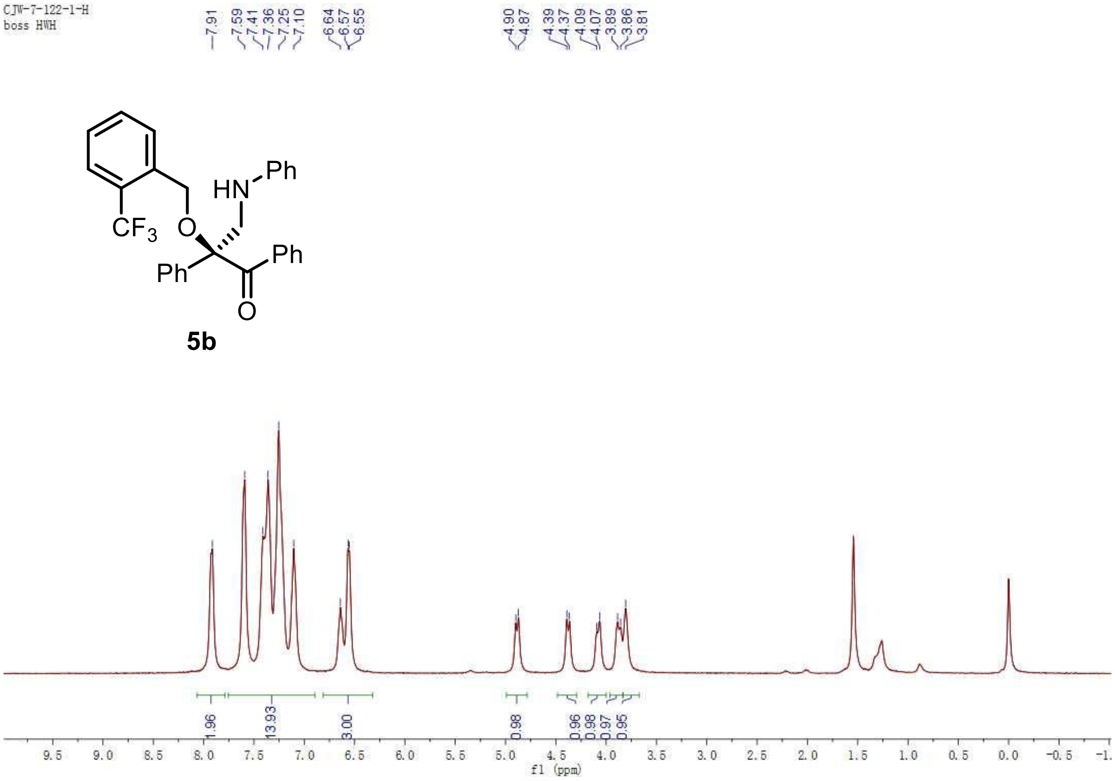

Supplementary Figure 8. ^{19}F NMR spectrum of (*S*)-**5b** (376 MHz, $CDCl_3$)

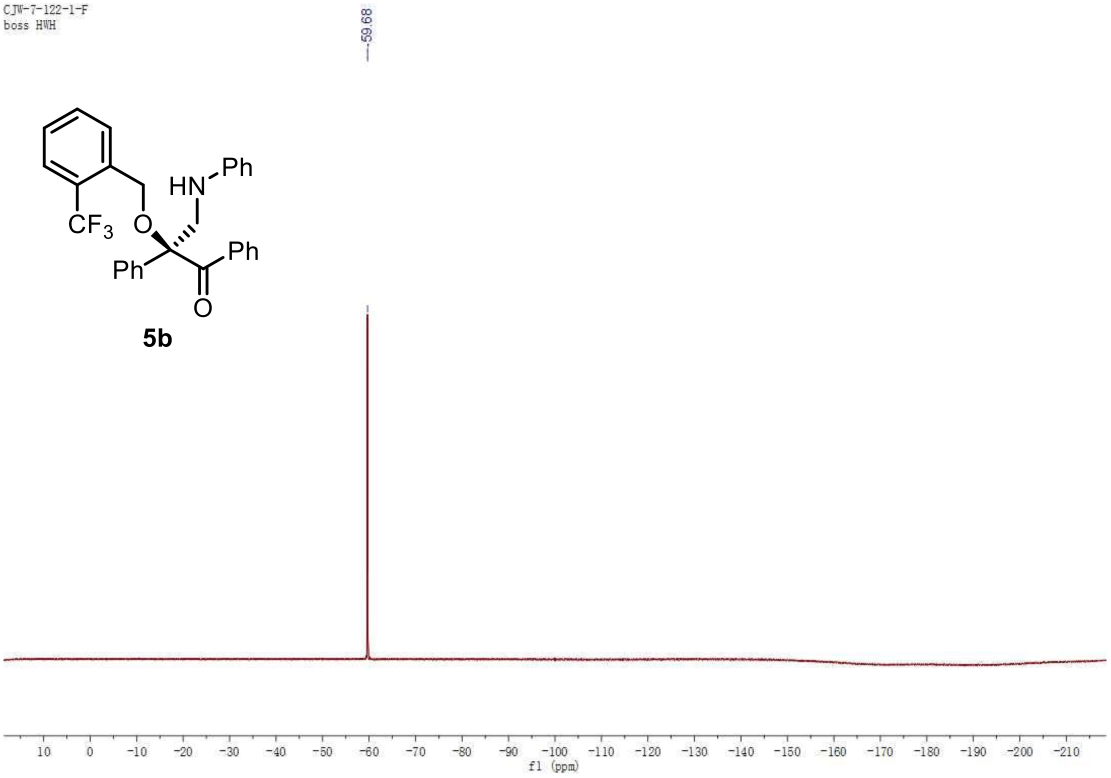

Supplementary Figure 9. ^{13}C NMR spectrum of (*S*)-**5b** (100 MHz, $CDCl_3$)

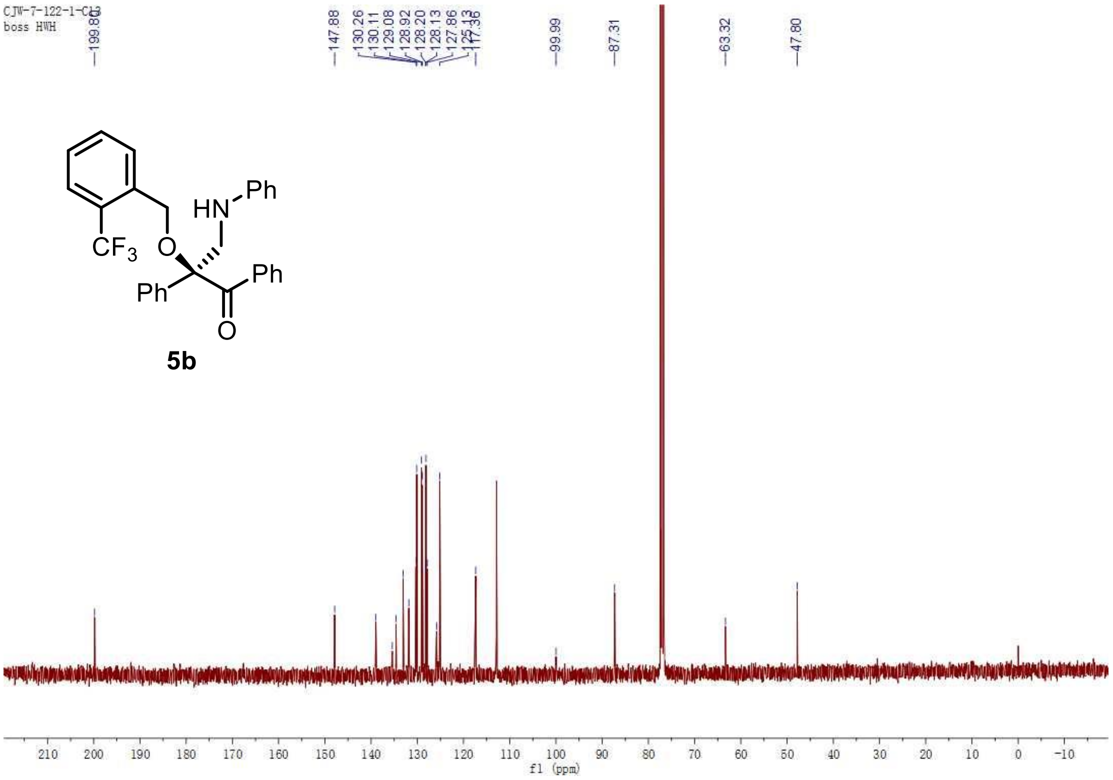

Supplementary Figure 10. ^{1}H NMR spectrum of (*S*)-**5c** (400 MHz, $CDCl_3$)

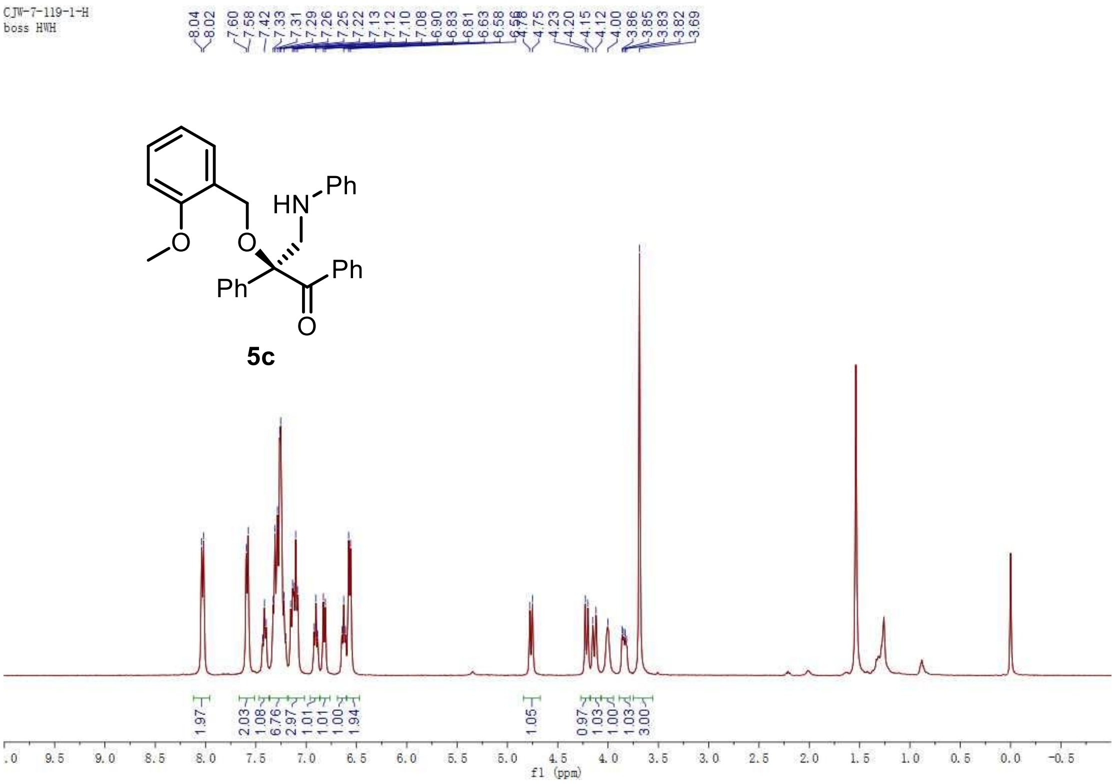

Supplementary Figure 11. ^{13}C NMR spectrum of (*S*)-**5c** (100 MHz, $CDCl_3$)

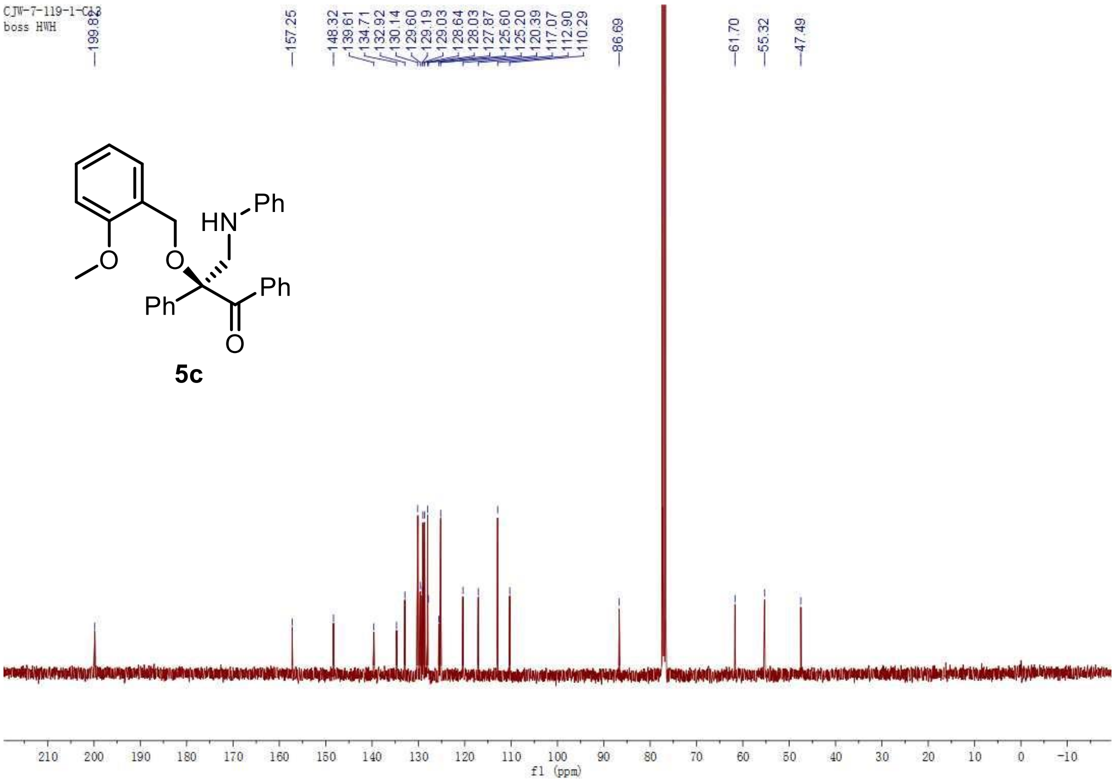

Supplementary Figure 12. ^{1}H NMR spectrum of (*S*)-**5d** (400 MHz, $CDCl_3$)

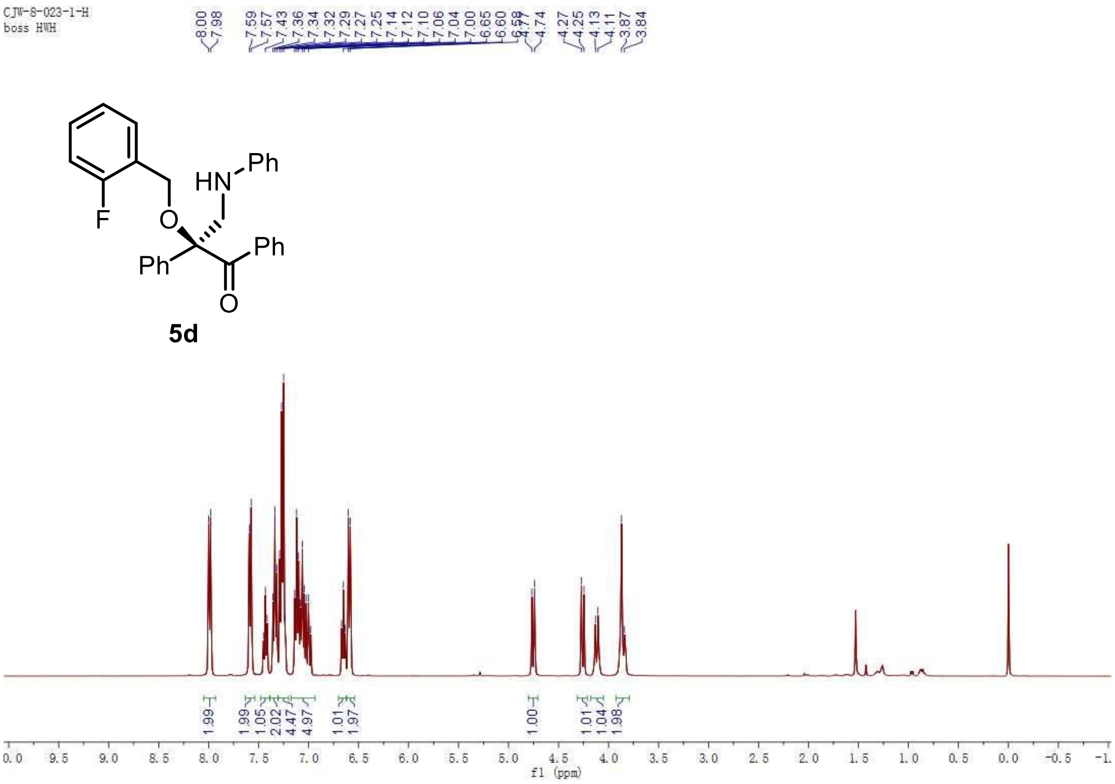

Supplementary Figure 13. ^{19}F NMR spectrum of (*S*)-**5d** (376 MHz, $CDCl_3$)

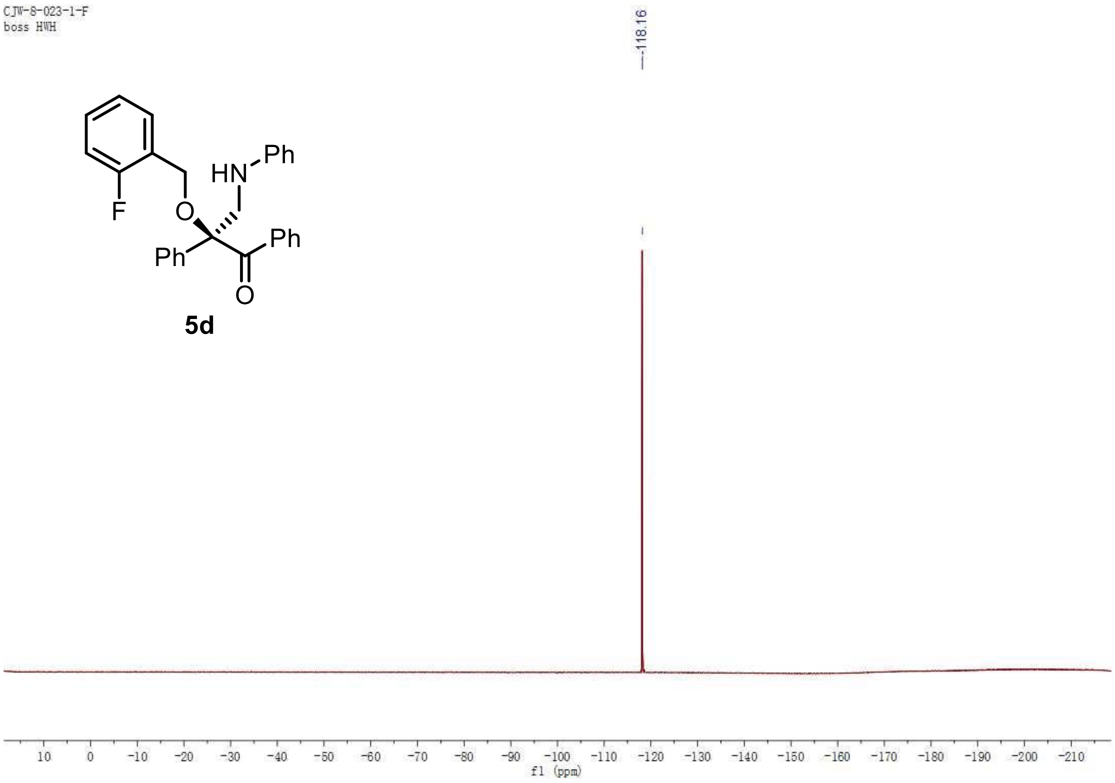

Supplementary Figure 14. ^{13}C NMR spectrum of (*S*)-**5d** (100 MHz, $CDCl_3$)

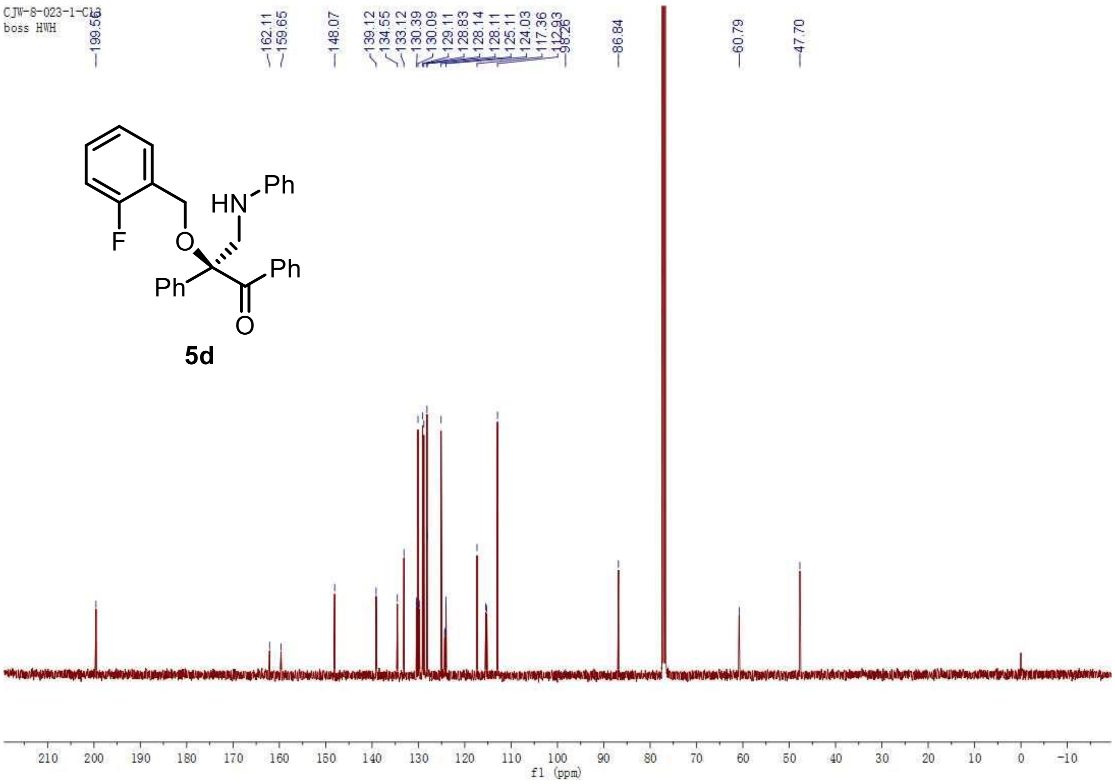

Supplementary Figure 15. ^{1}H NMR spectrum of (*S*)-**5e** (400 MHz, $CDCl_3$)

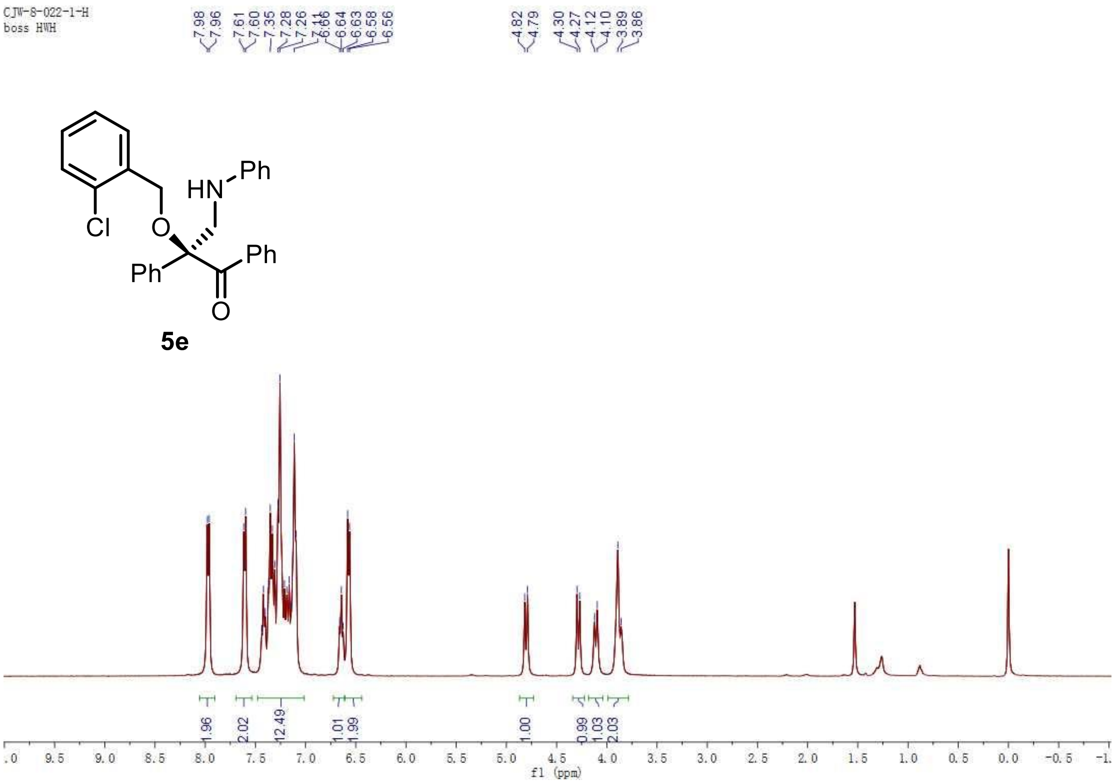

Supplementary Figure 16. ^{13}C NMR spectrum of (*S*)-**5e** (100 MHz, $CDCl_3$)

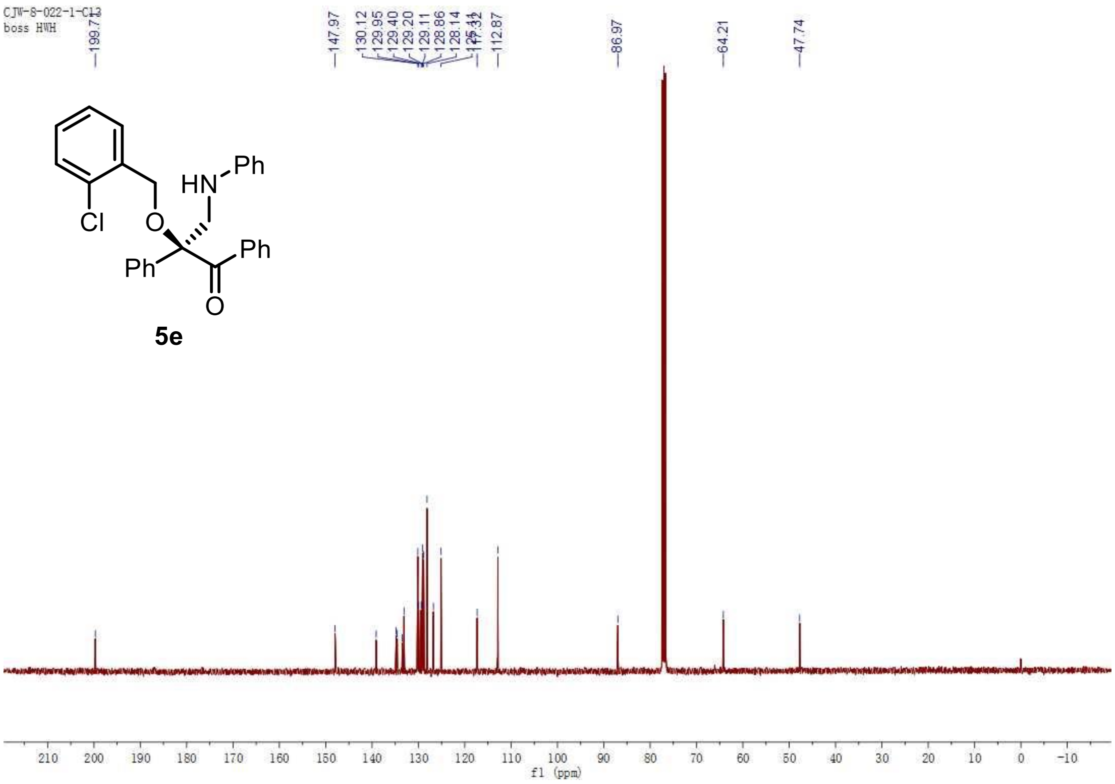

Supplementary Figure 17. ^{1}H NMR spectrum of (*S*)-**5f** (400 MHz, $CDCl_3$)

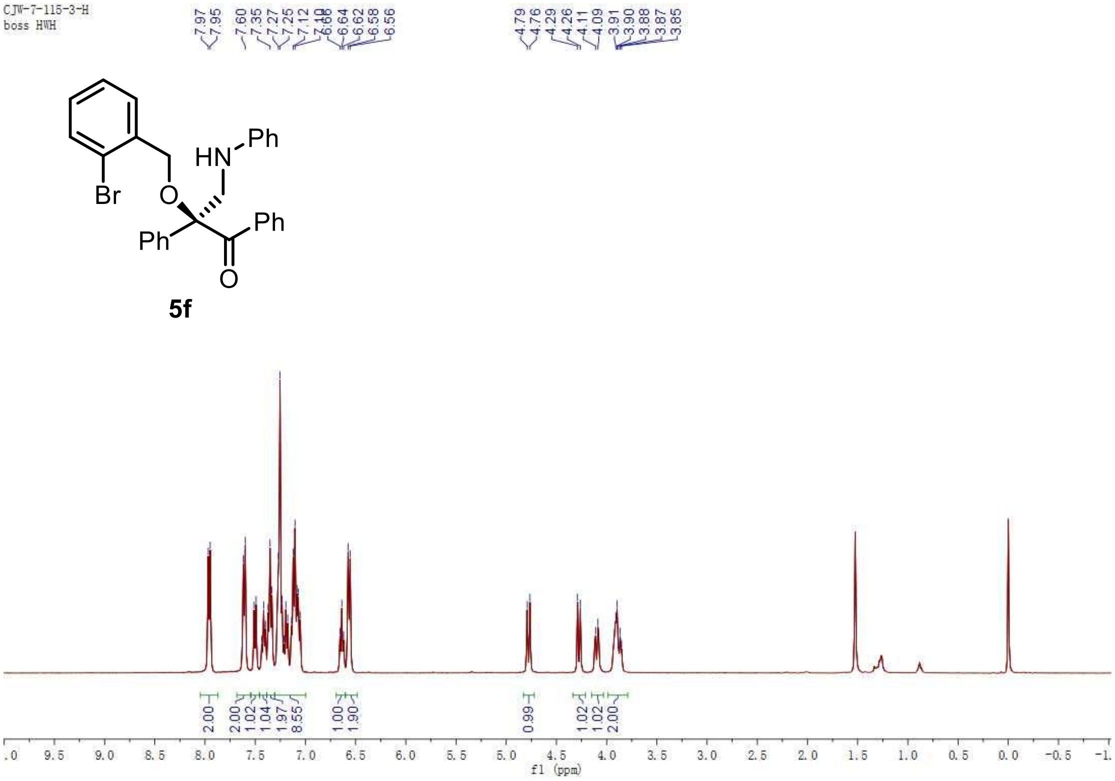

Supplementary Figure 18. ^{13}C NMR spectrum of (*S*)-**5f** (100 MHz, $CDCl_3$)

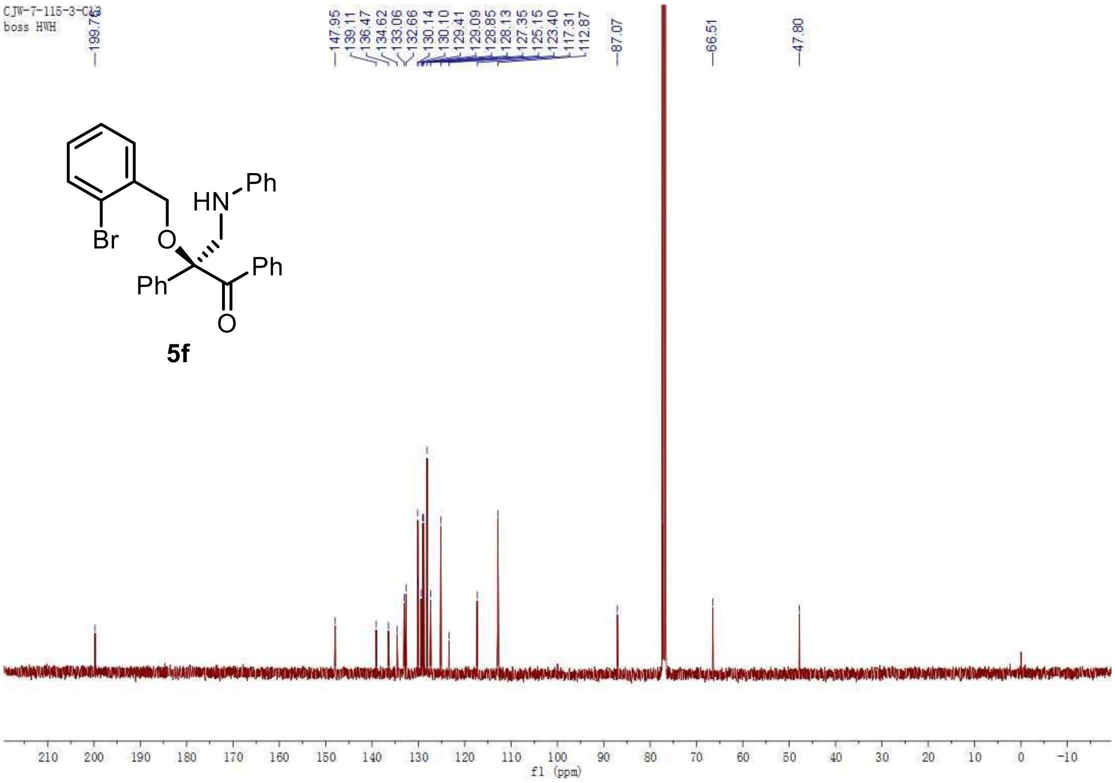

Supplementary Figure 19. ^{1}H NMR spectrum of (*S*)-**5g** (400 MHz, $CDCl_3$)

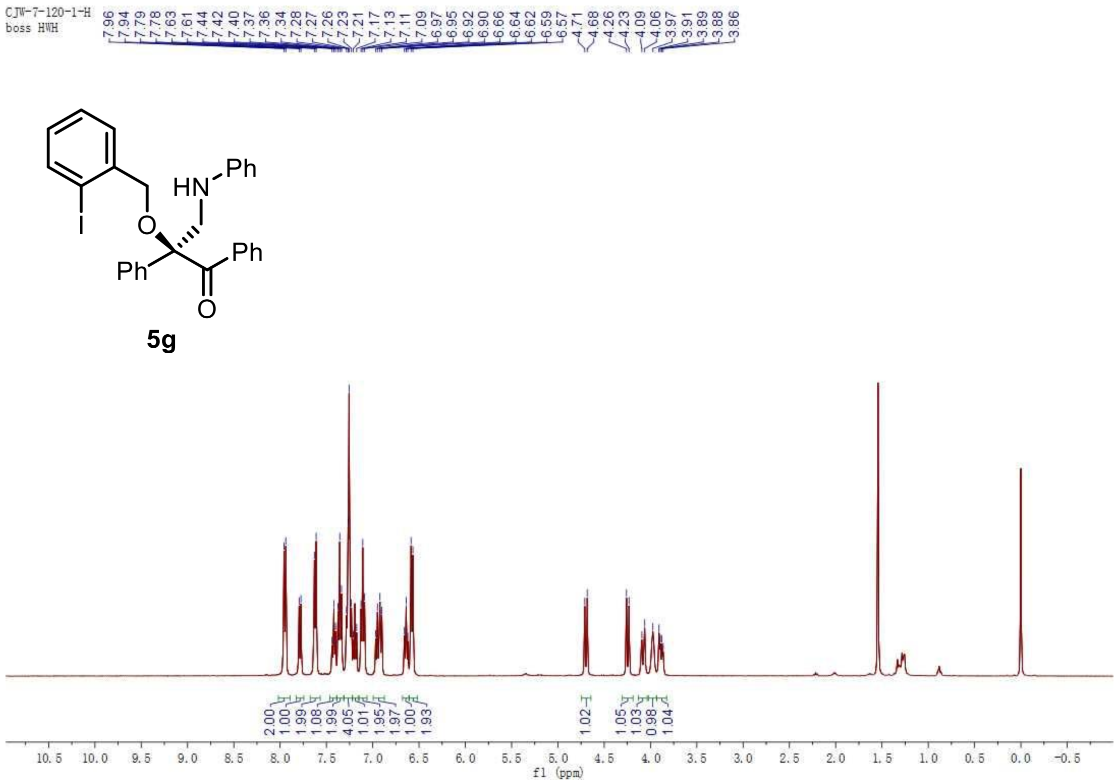

Supplementary Figure 20. ^{13}C NMR spectrum of (*S*)-**5g** (100 MHz, $CDCl_3$)

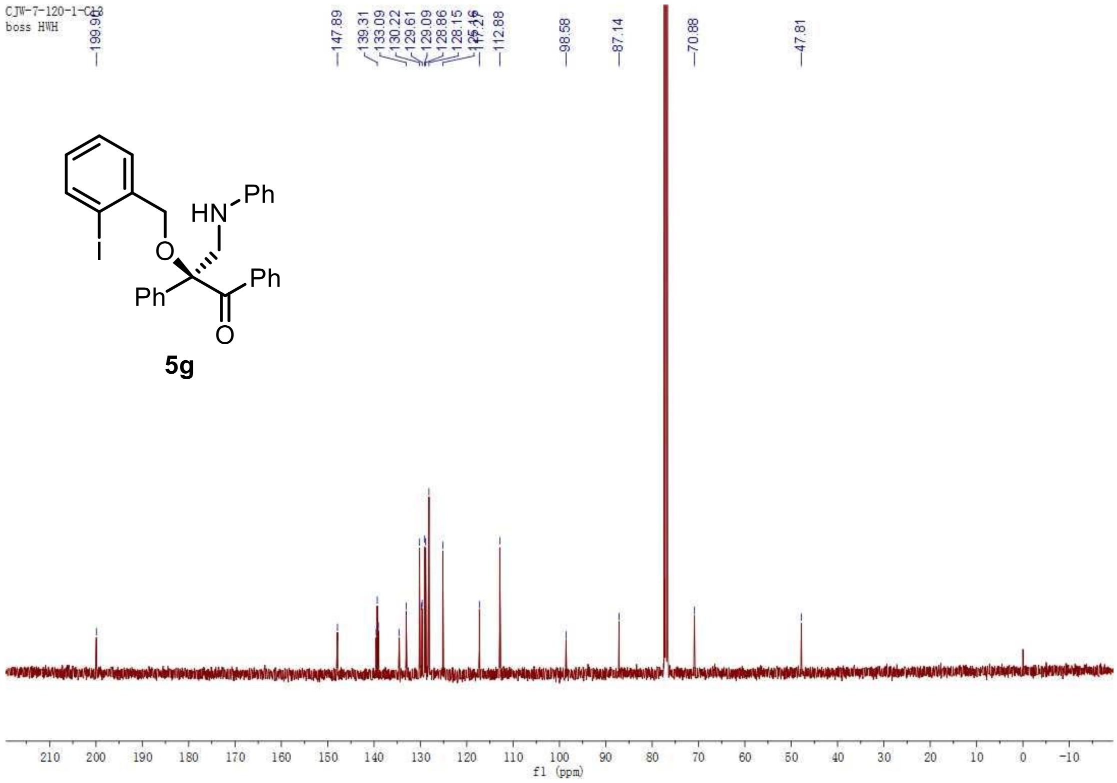

Supplementary Figure 21. ^{1}H NMR spectrum of (*S*)-**5h** (400 MHz, $CDCl_3$)

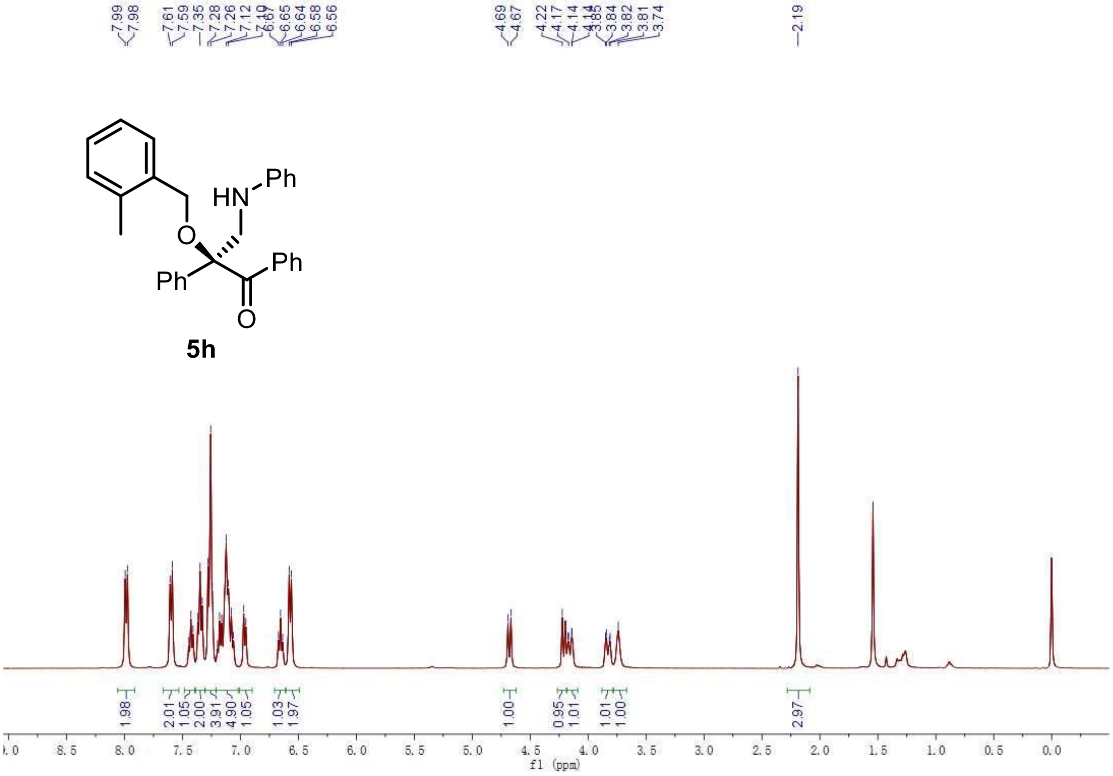

Supplementary Figure 22. ^{13}C NMR spectrum of (*S*)-**5h** (100 MHz, $CDCl_3$)

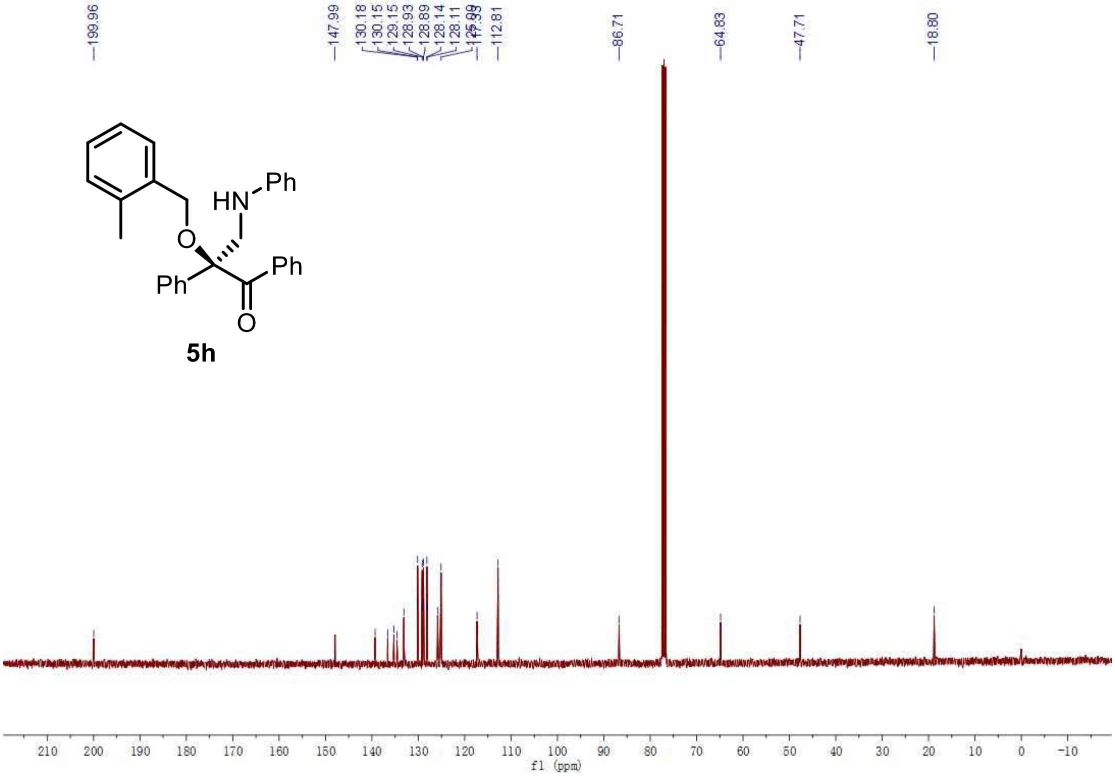

Supplementary Figure 23. ^{1}H NMR spectrum of (*S*)-**5i** (400 MHz, $CDCl_3$)

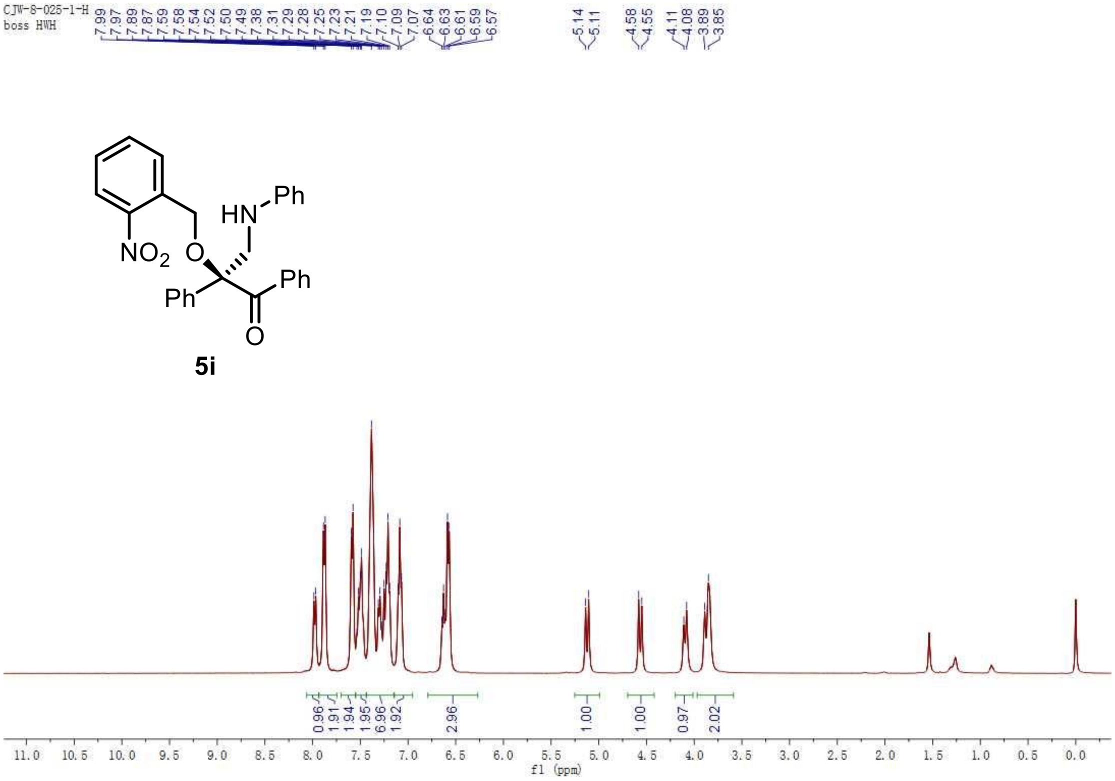

Supplementary Figure 24. ^{13}C NMR spectrum of (*S*)-**5i** (100 MHz, $CDCl_3$)

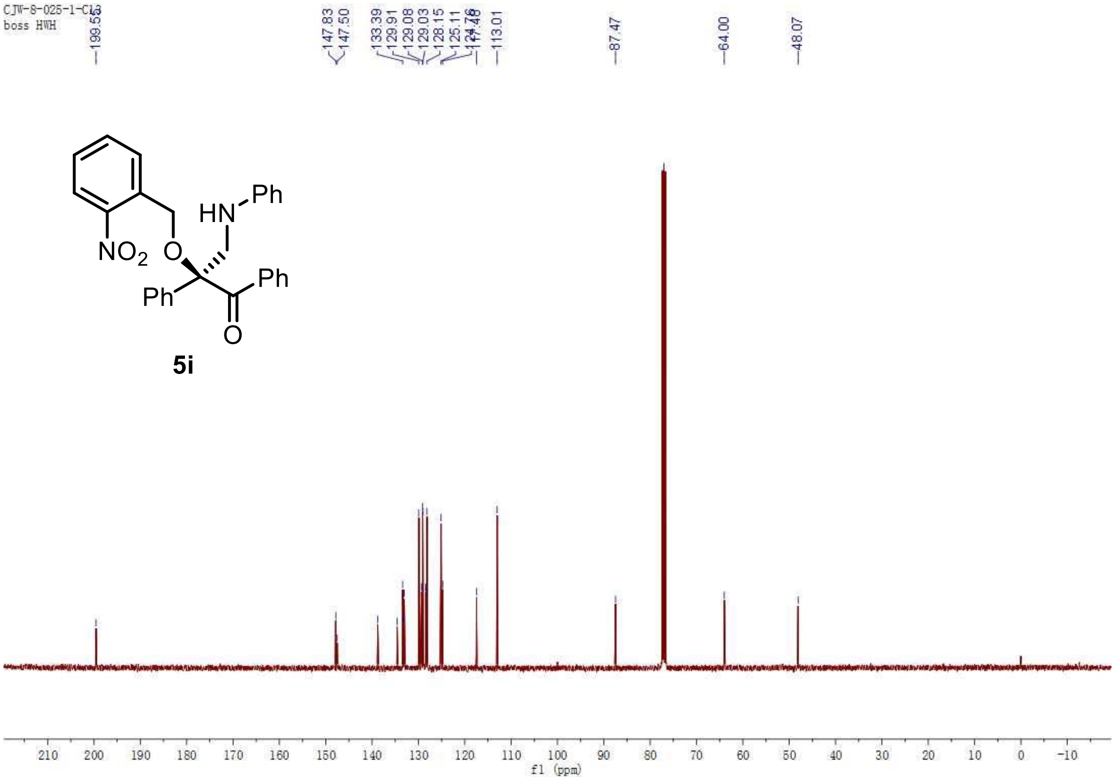

Supplementary Figure 25. ^{1}H NMR spectrum of (*S*)-**5j** (400 MHz, $CDCl_3$)

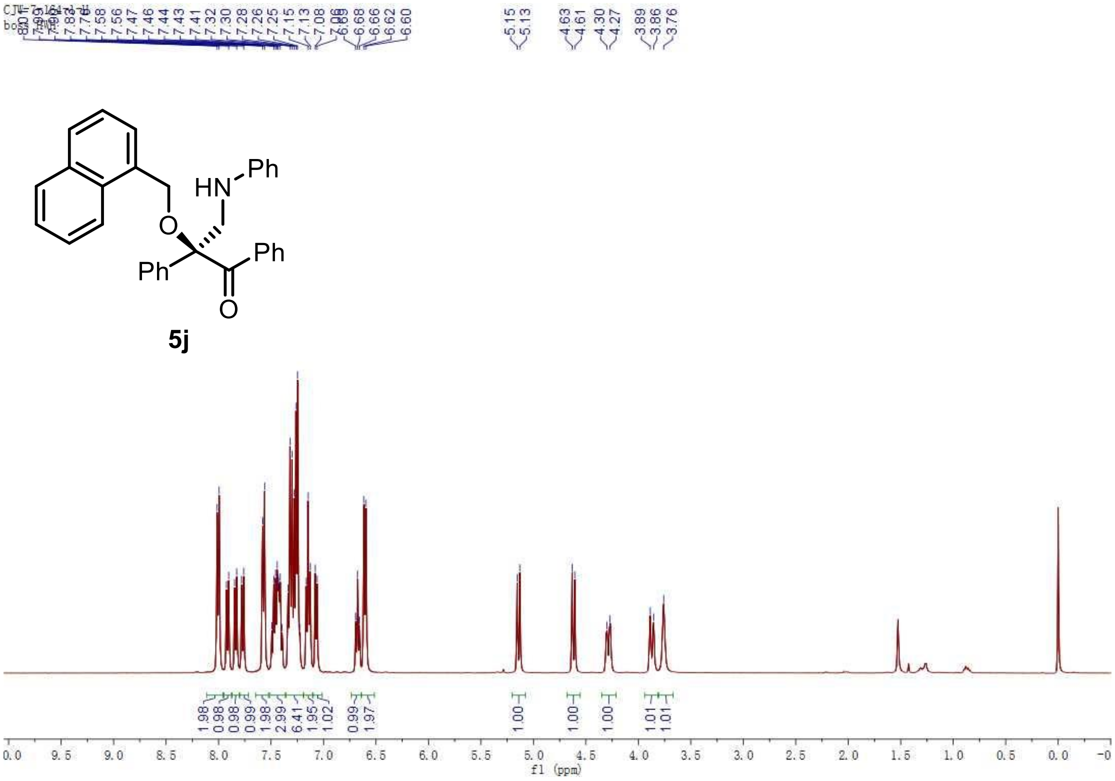

Supplementary Figure 26. ^{13}C NMR spectrum of (*S*)-**5j** (100 MHz, $CDCl_3$)

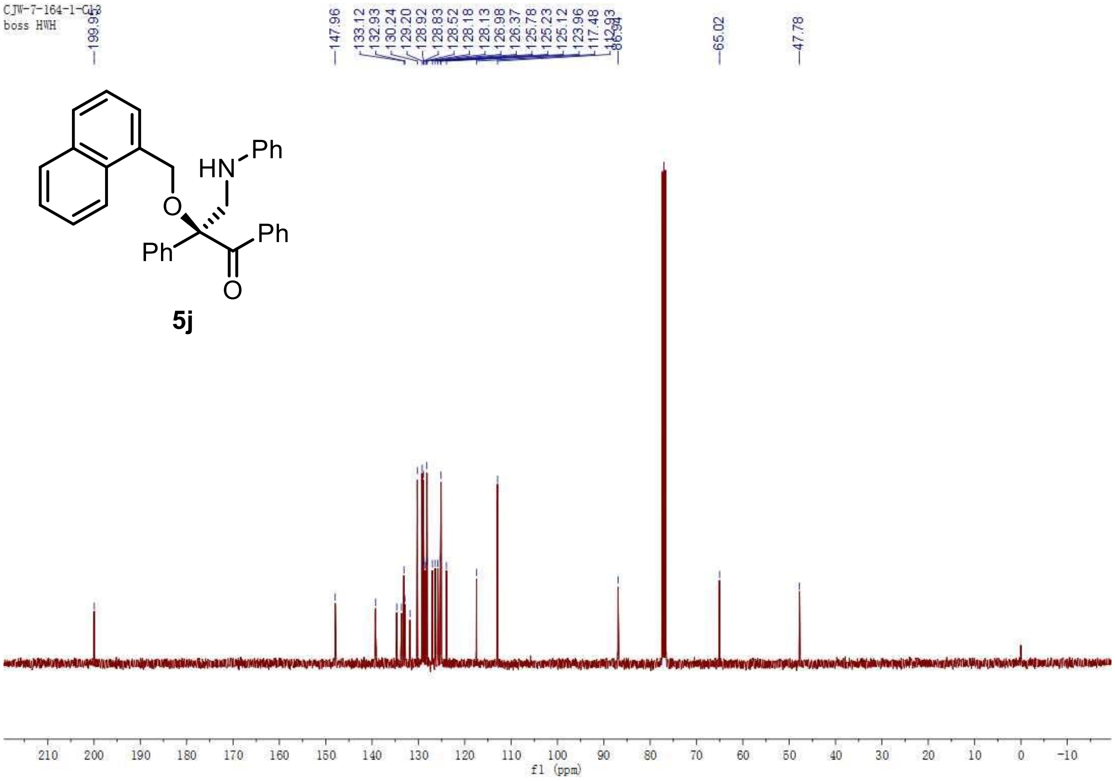

Supplementary Figure 27. ^{1}H NMR spectrum of (S)-**5k** (400 MHz, $CDCl_3$)

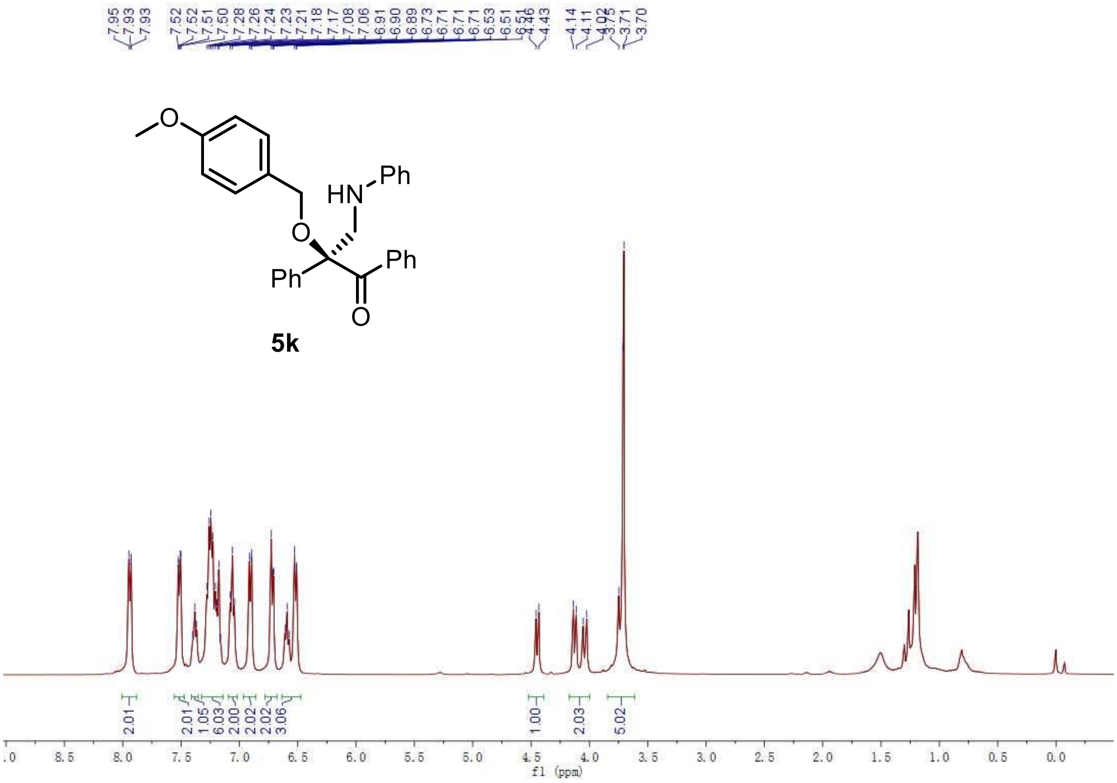

Supplementary Figure 28. ^{13}C NMR spectrum of (S)-**5k** (100 MHz, $CDCl_3$)

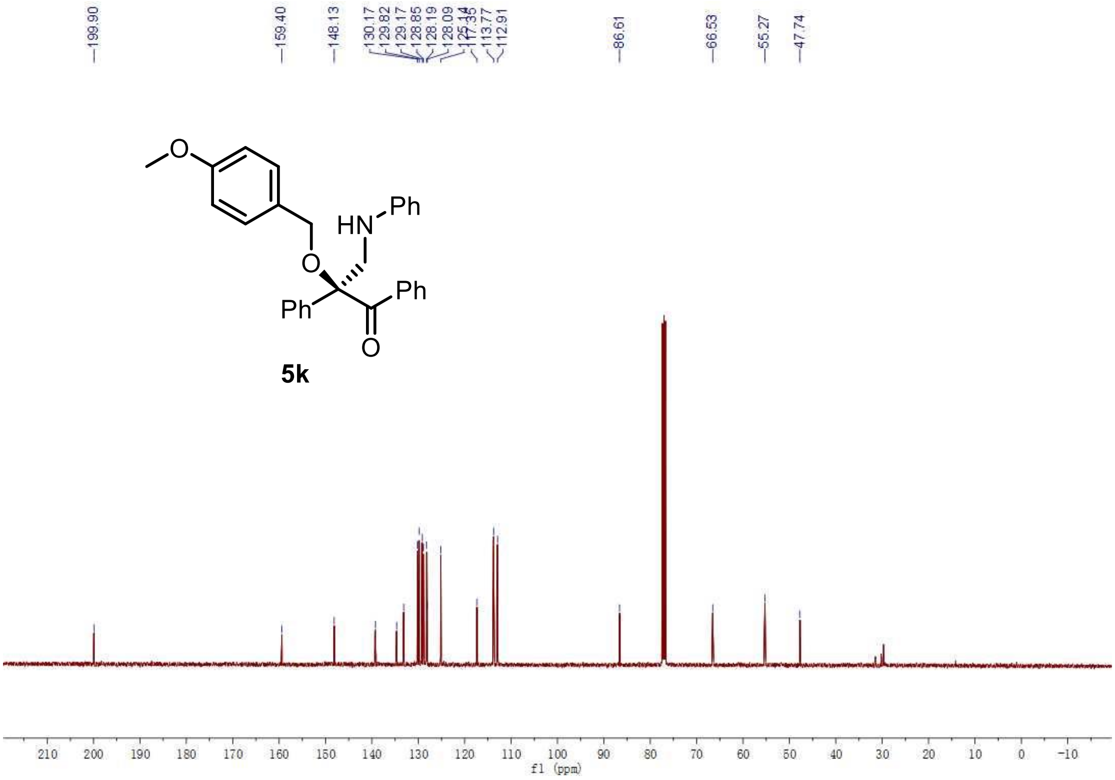

Supplementary Figure 29. ^{1}H NMR spectrum of (*S*)-**5l** (400 MHz, $CDCl_3$)

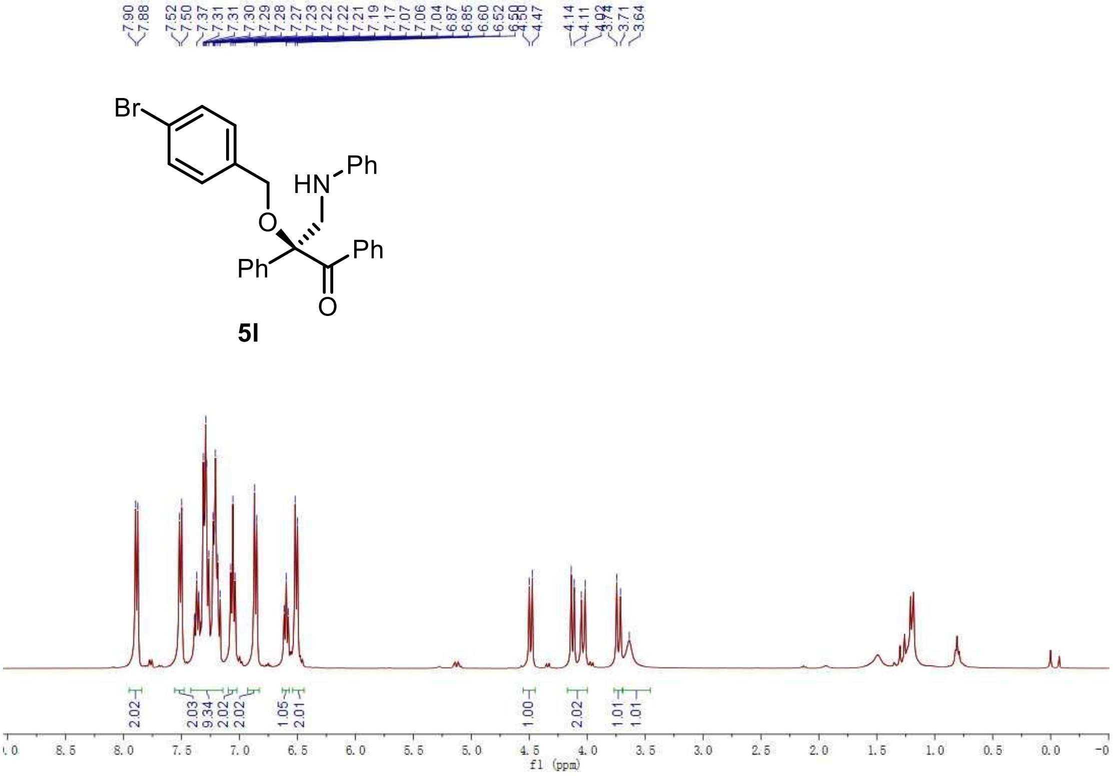

Supplementary Figure 30. ^{13}C NMR spectrum of (*S*)-**5l** (100 MHz, $CDCl_3$)

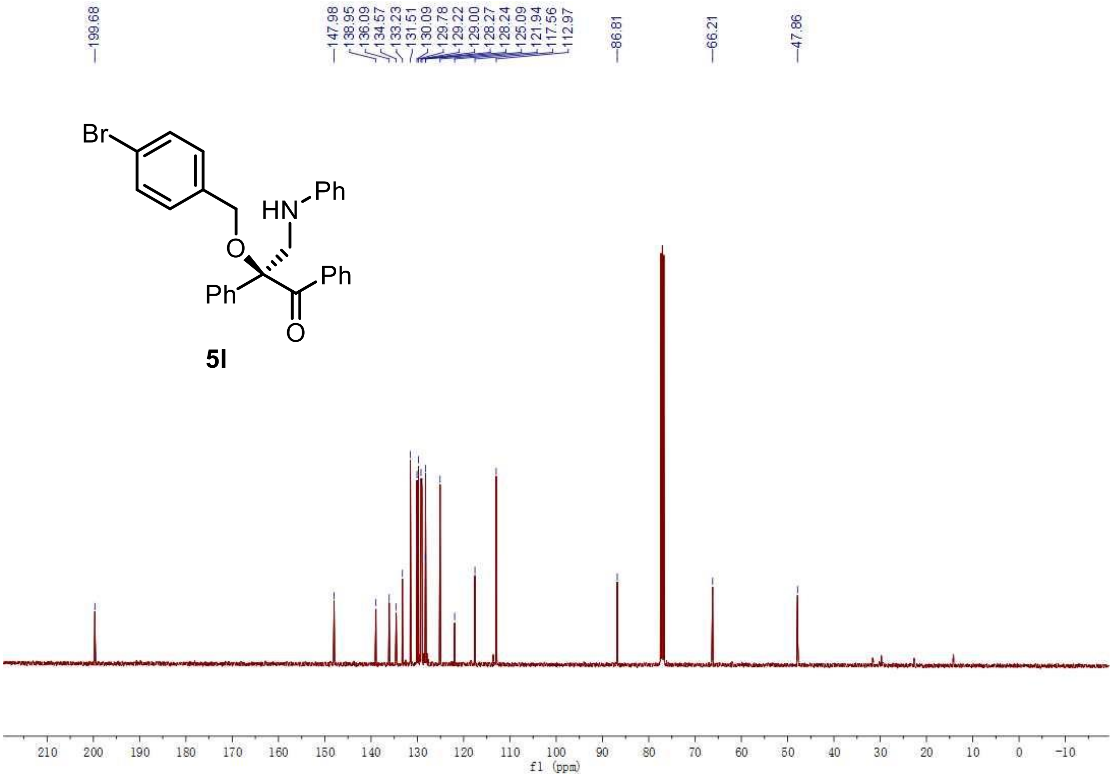

Supplementary Figure 31. ^{1}H NMR spectrum of (*S*)-**5m** (400 MHz, $CDCl_3$)

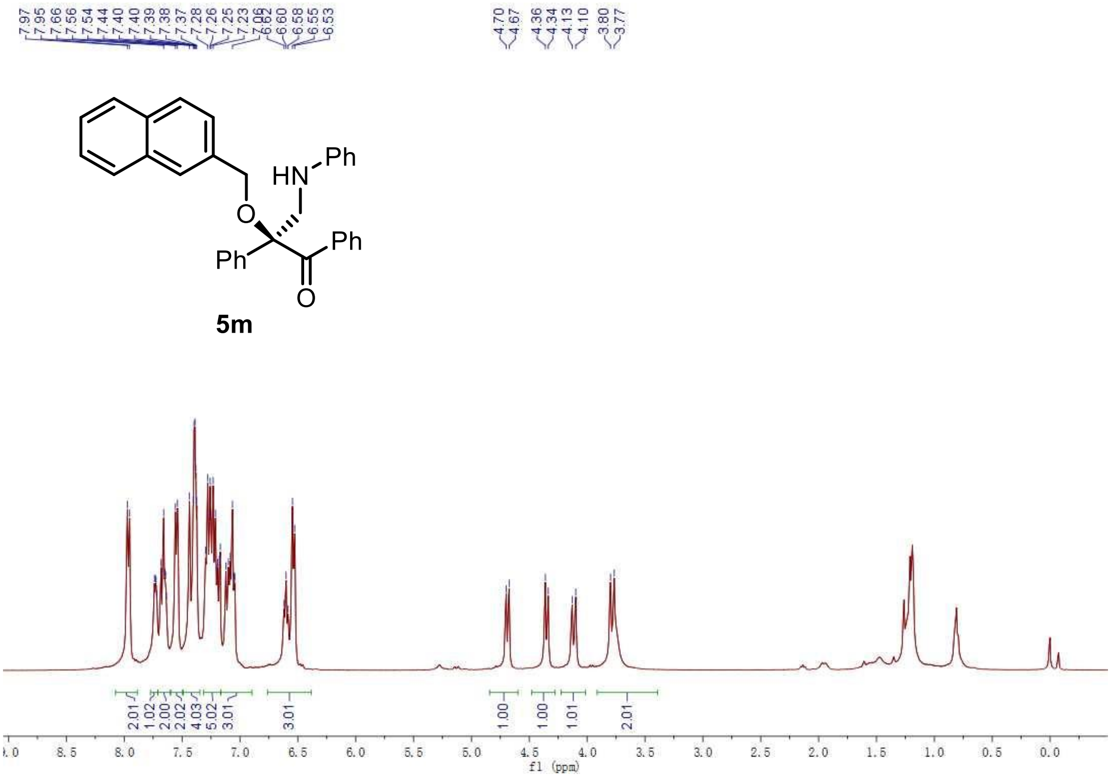

Supplementary Figure 32. ^{13}C NMR spectrum of (*S*)-**5m** (100 MHz, $CDCl_3$)

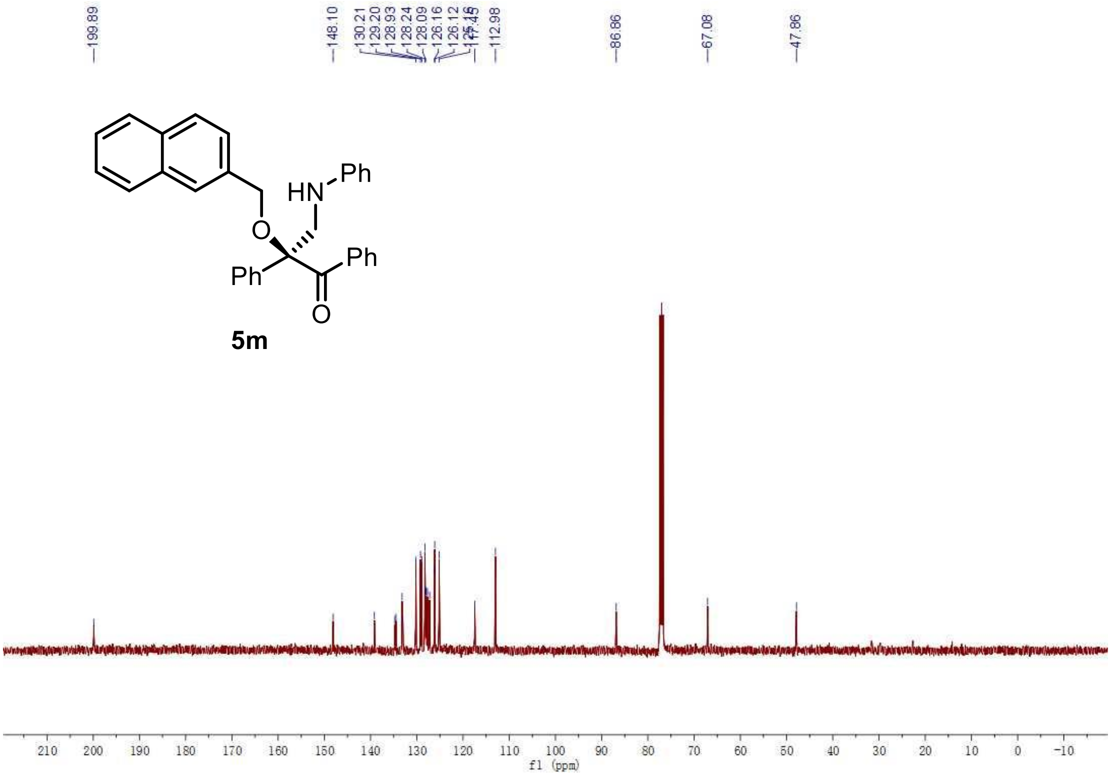

Supplementary Figure 33. ^{1}H NMR spectrum of (*S*)-**5n** (400 MHz, $CDCl_3$)

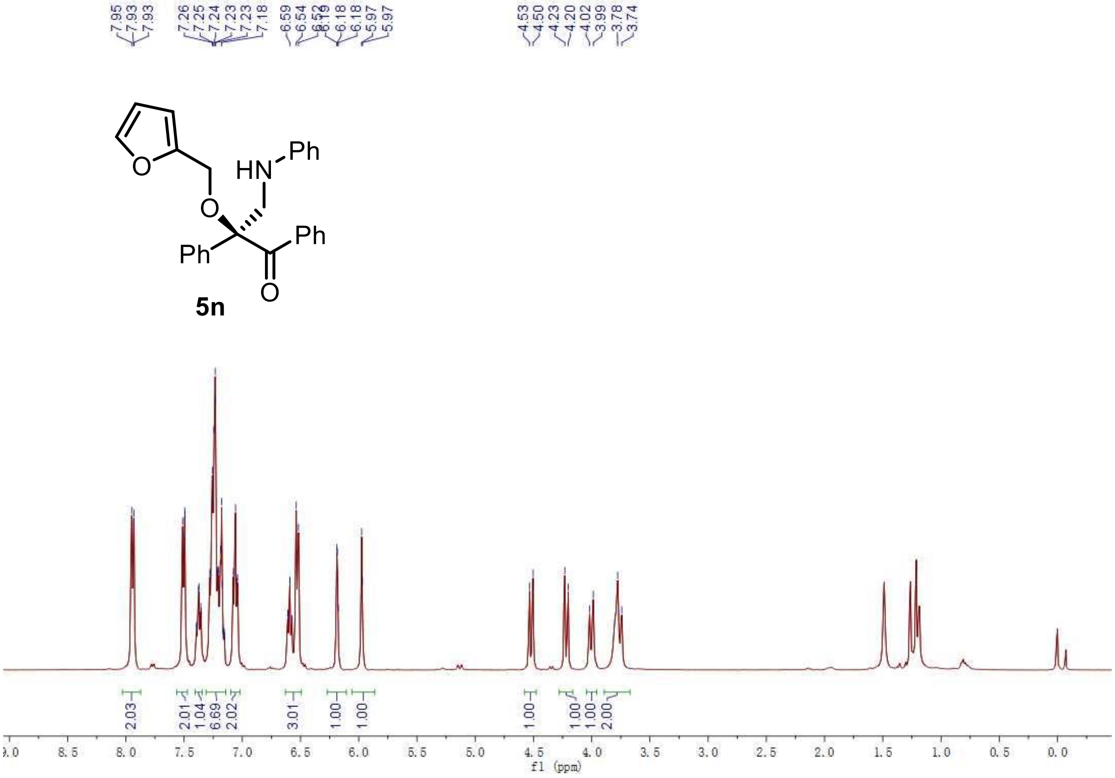

Supplementary Figure 34. ^{13}C NMR spectrum of (*S*)-**5n** (100 MHz, $CDCl_3$)

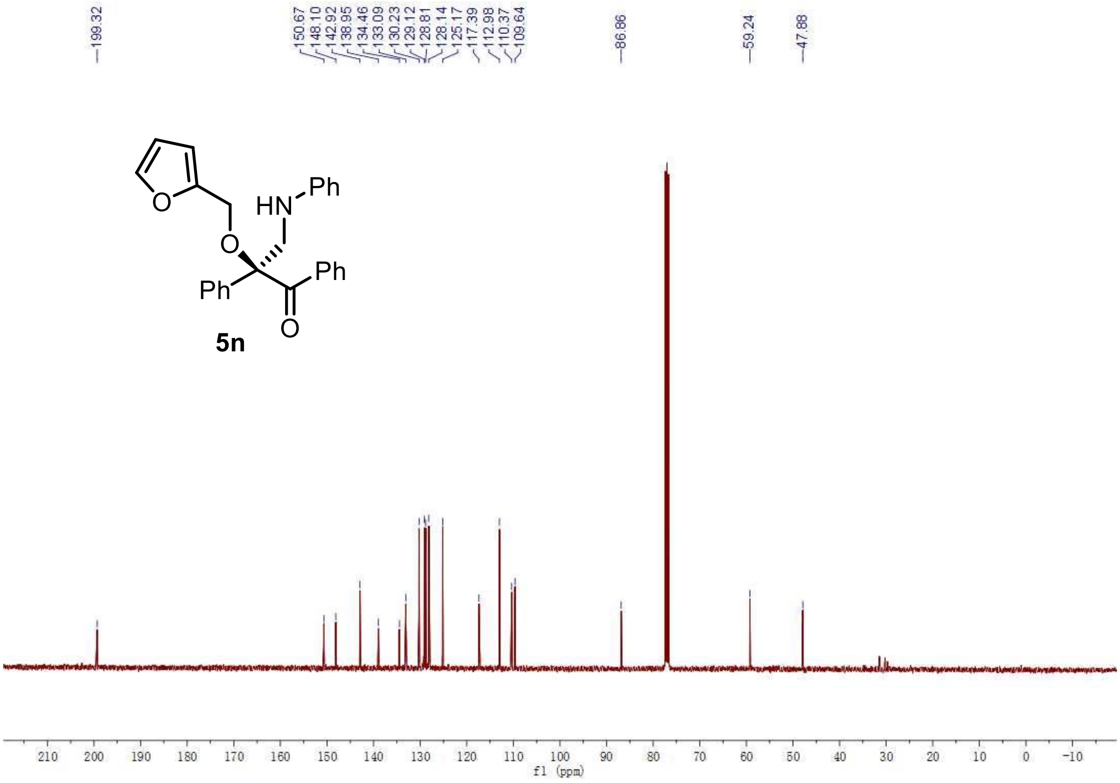

Supplementary Figure 35. ^{1}H NMR spectrum of (*S*)-**5o** (400 MHz, $CDCl_3$)

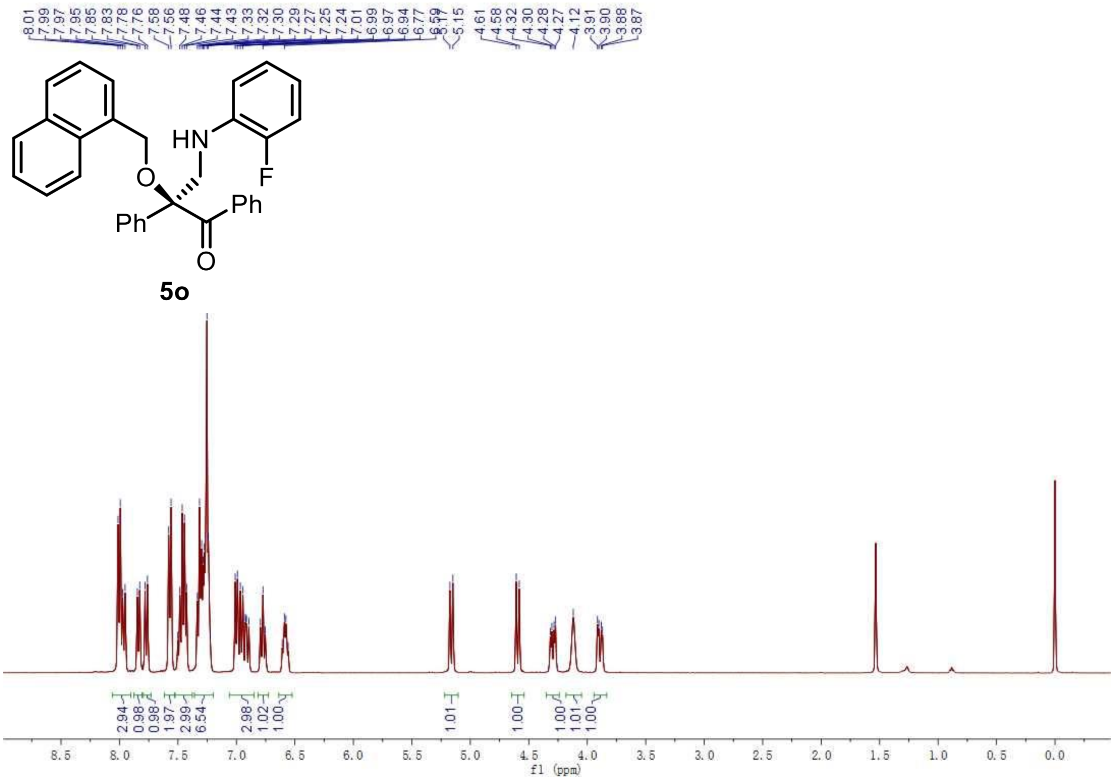

Supplementary Figure 36. ^{19}F NMR spectrum of (*S*)-**5o** (376 MHz, $CDCl_3$)

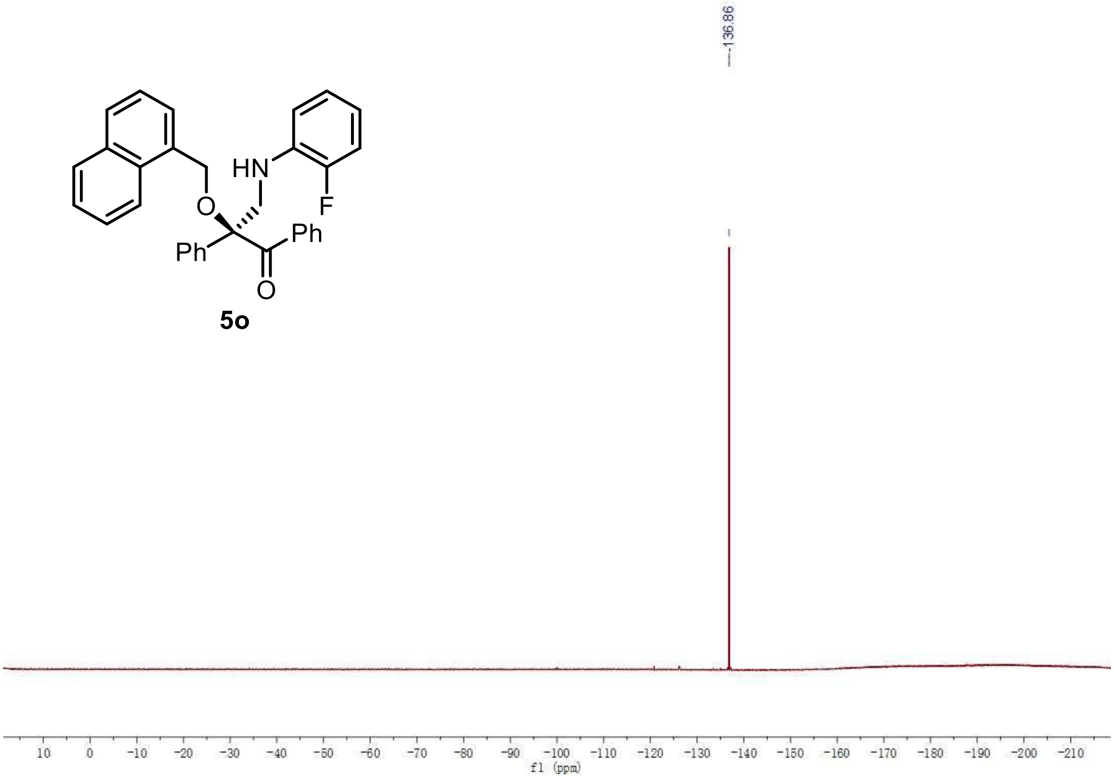

Supplementary Figure 37. ^{13}C NMR spectrum of (*S*)-**5o** (100 MHz, $CDCl_3$)

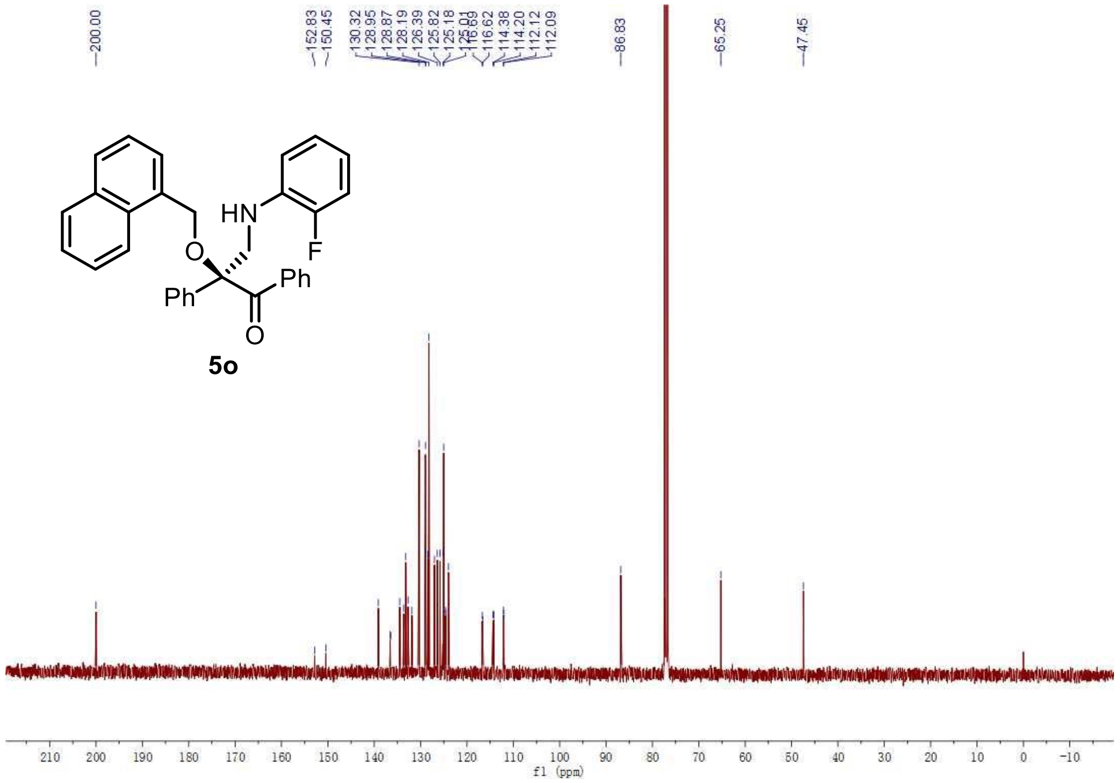

Supplementary Figure 38. ^{1}H NMR spectrum of (*S*)-**5p** (400 MHz, $CDCl_3$)

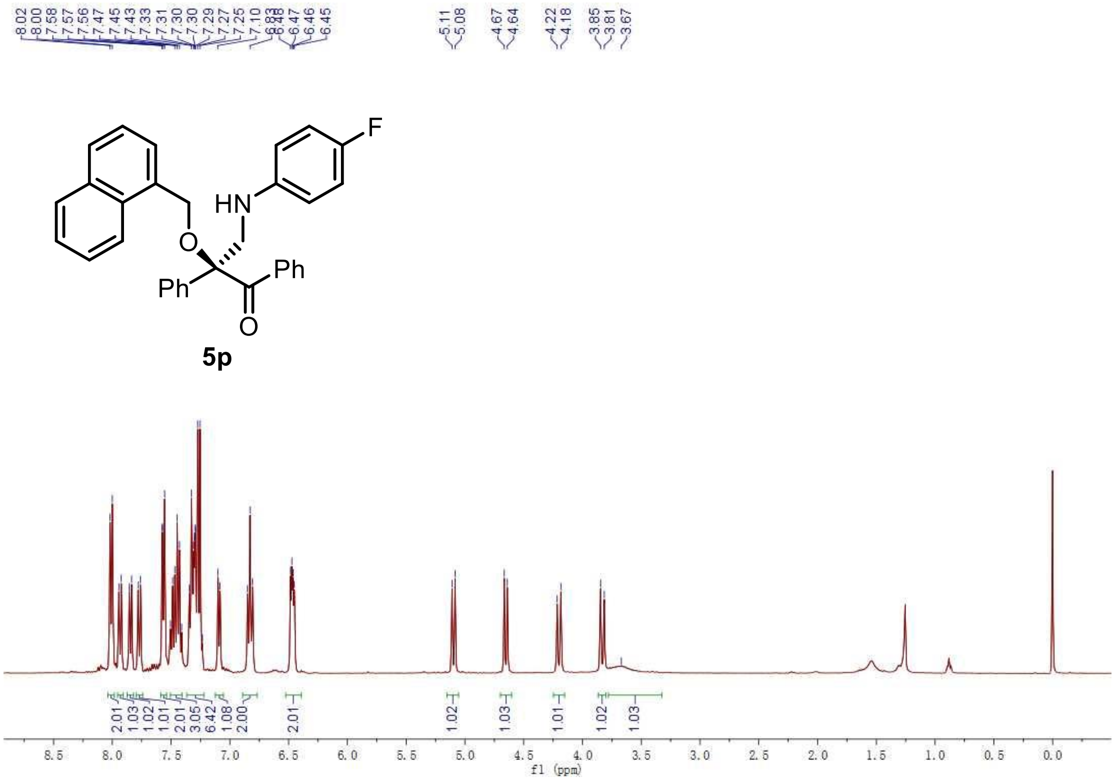

Supplementary Figure 39. ^{19}F NMR spectrum of (*S*)-**5p** (376 MHz, $CDCl_3$)

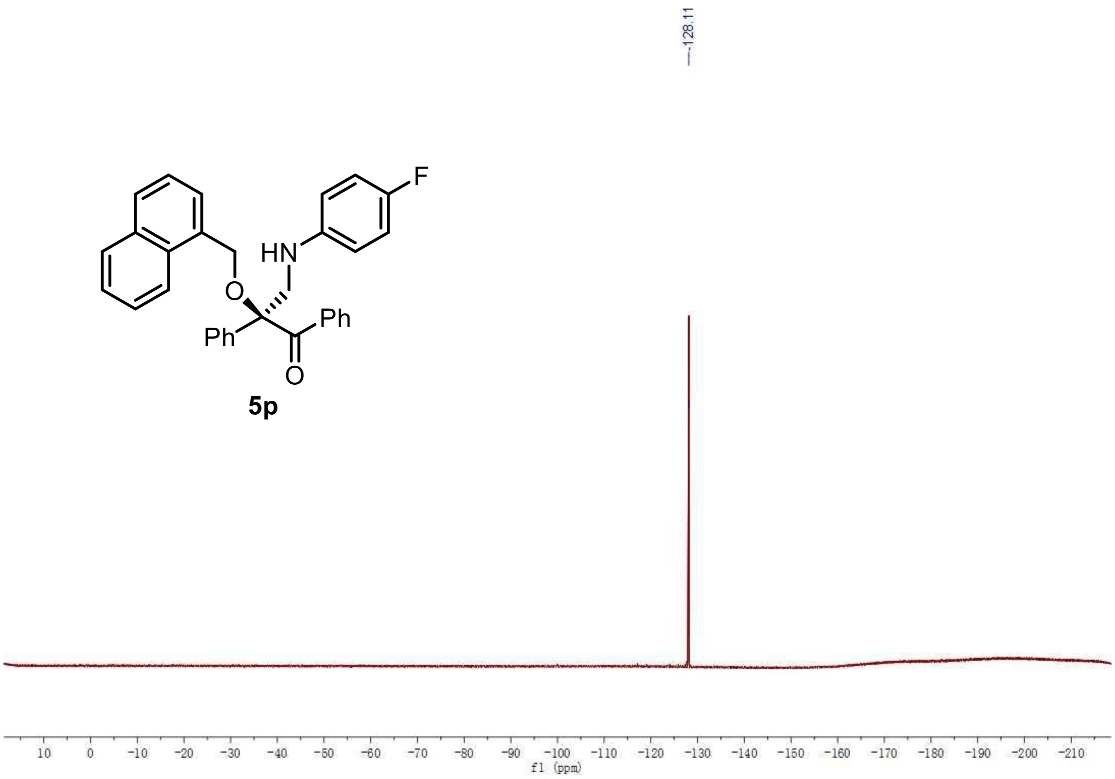

Supplementary Figure 40. ^{13}C NMR spectrum of (*S*)-**5p** (100 MHz, $CDCl_3$)

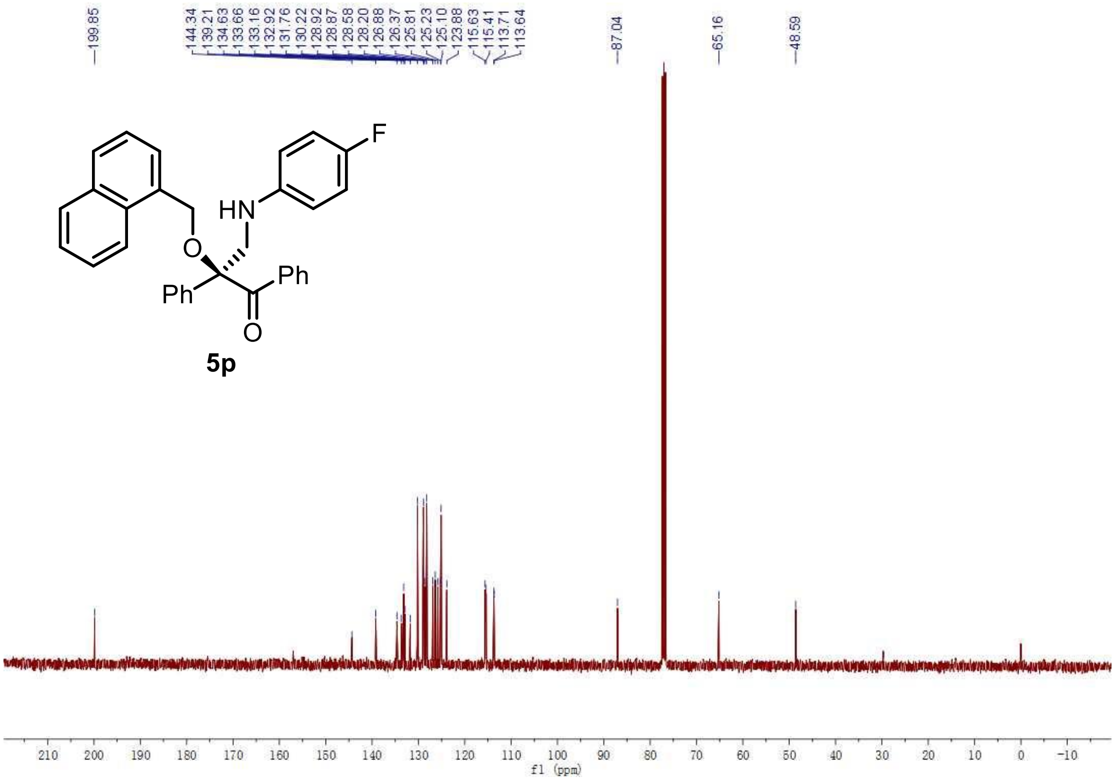

Supplementary Figure 41. ^{1}H NMR spectrum of (*S*)-**5q** (400 MHz, $CDCl_3$)

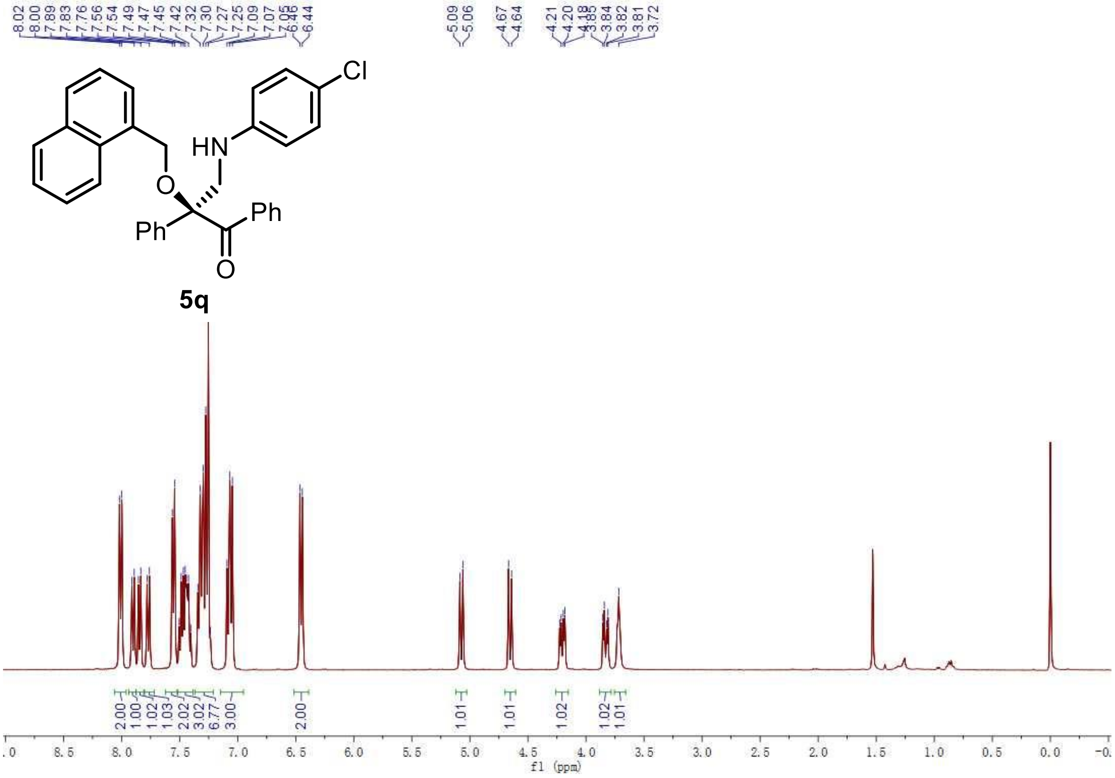

Supplementary Figure 42. ^{13}C NMR spectrum of (*S*)-**5q** (100 MHz, $CDCl_3$)

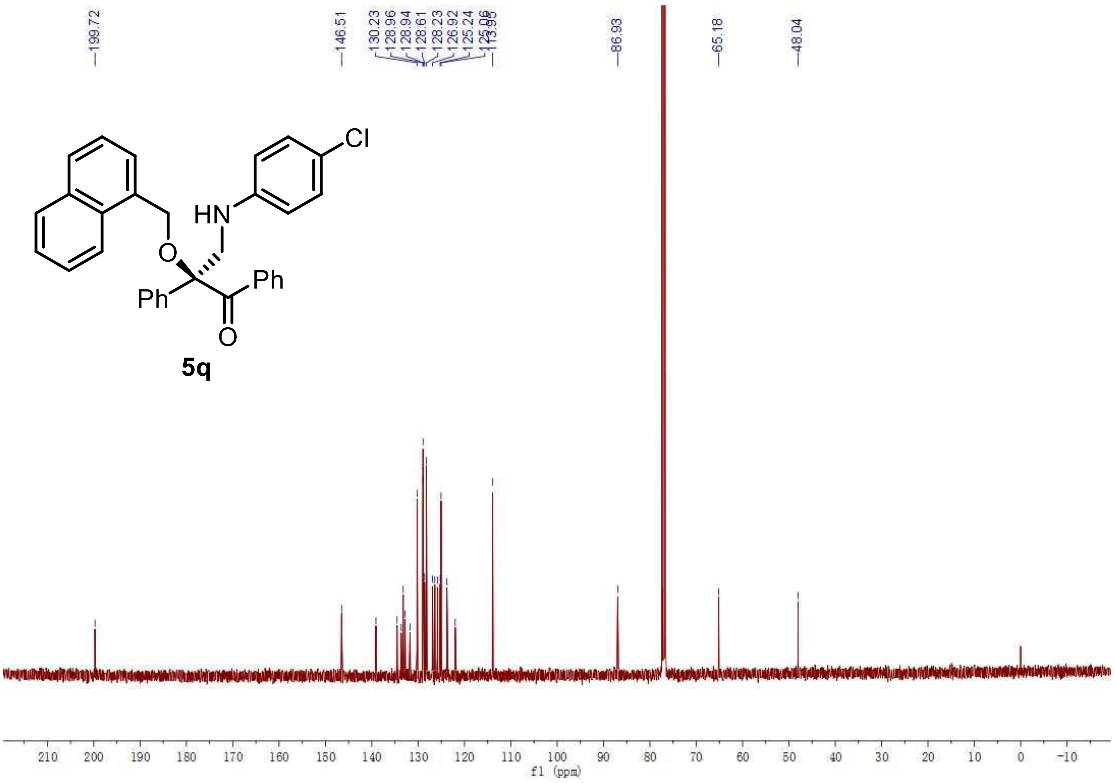

Supplementary Figure 43. ^{1}H NMR spectrum of (*S*)-**5r** (400 MHz, $CDCl_3$)

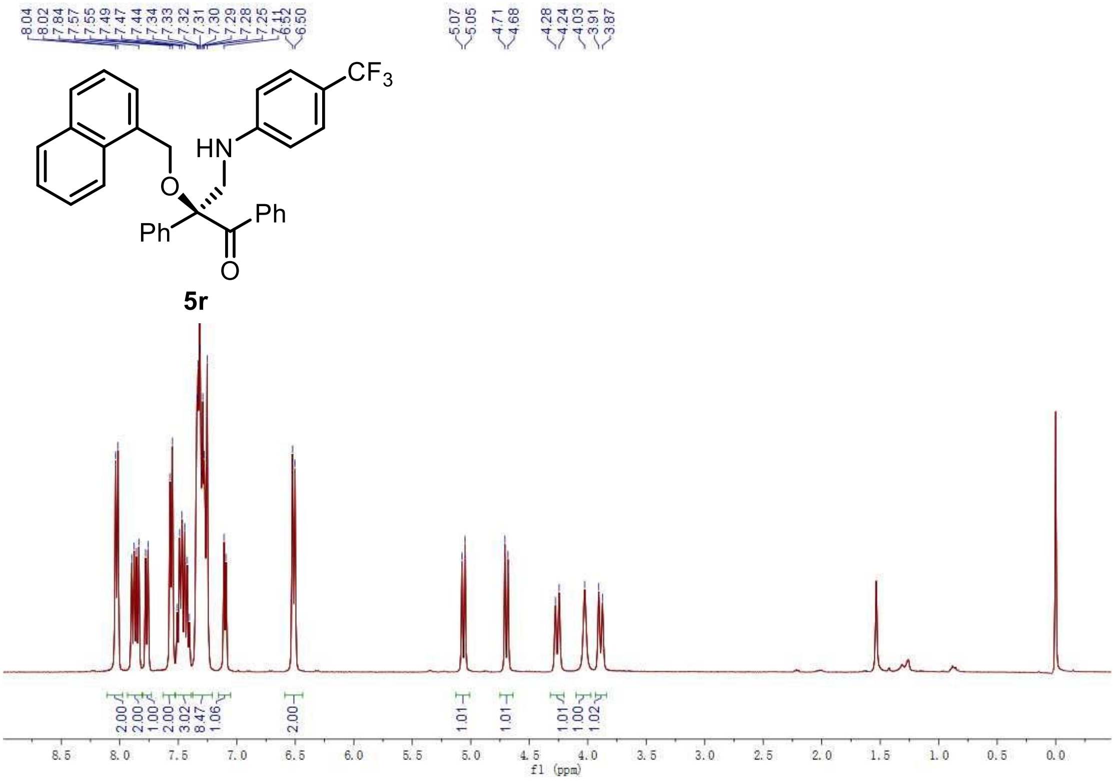

Supplementary Figure 44. ^{19}F NMR spectrum of (*S*)-**5r** (376 MHz, $CDCl_3$)

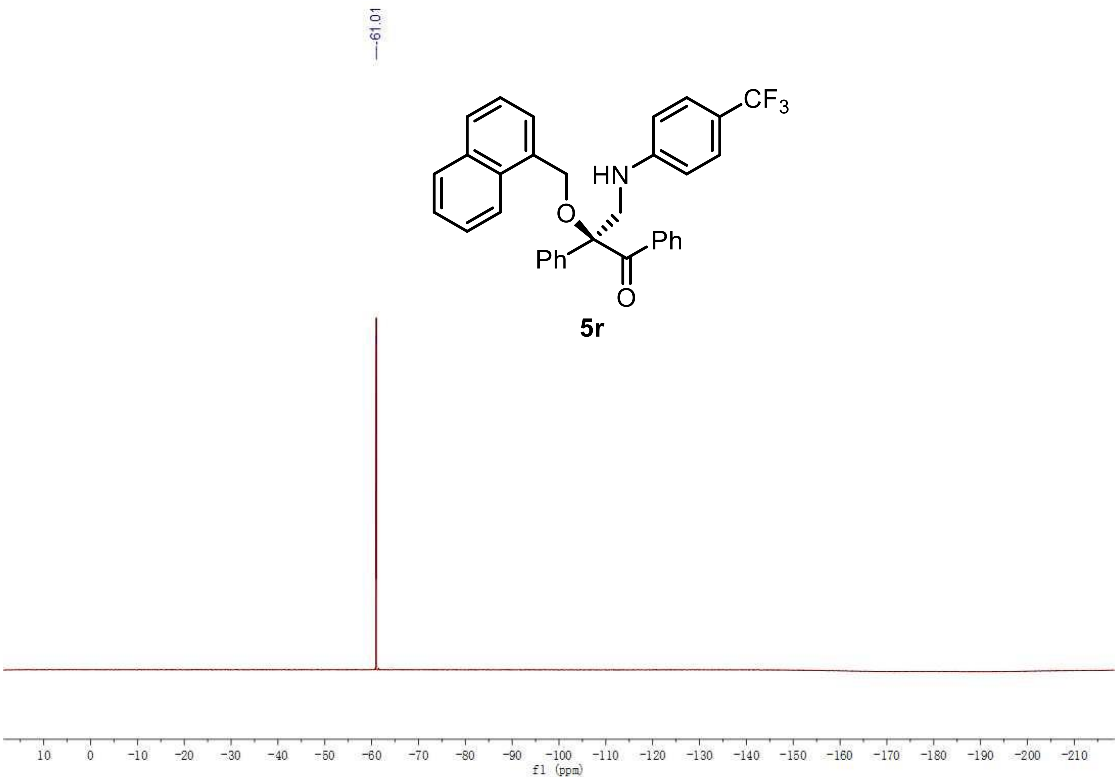

Supplementary Figure 45. ^{13}C NMR spectrum of (*S*)-**5r** (100 MHz, $CDCl_3$)

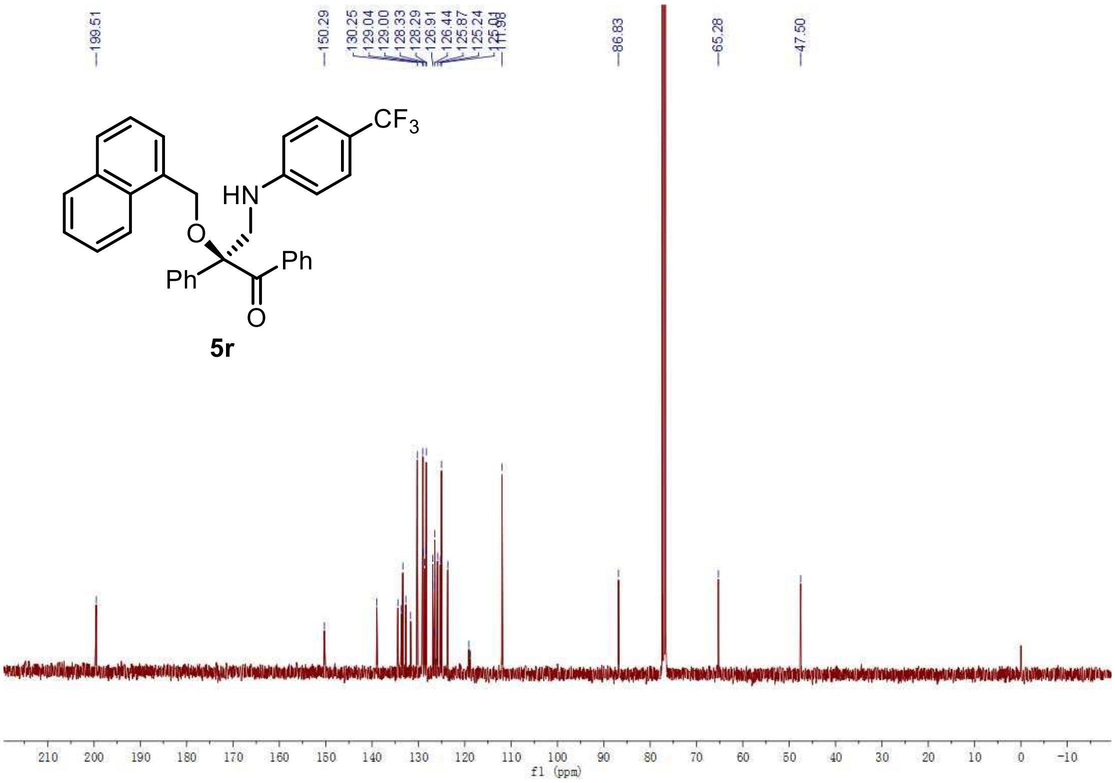

Supplementary Figure 46. ^{1}H NMR spectrum of (*S*)-**5s** 400 MHz, $CDCl_3$)

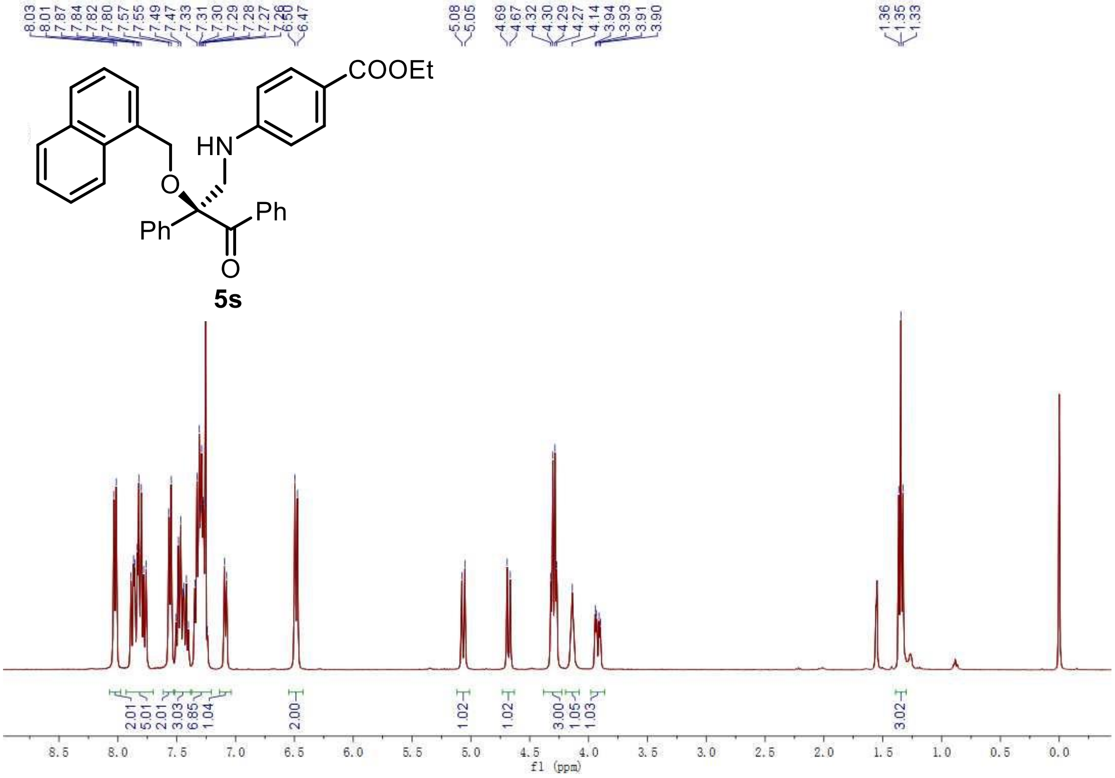

Supplementary Figure 47. ^{13}C NMR spectrum of (*S*)-**5s** (100 MHz, $CDCl_3$)

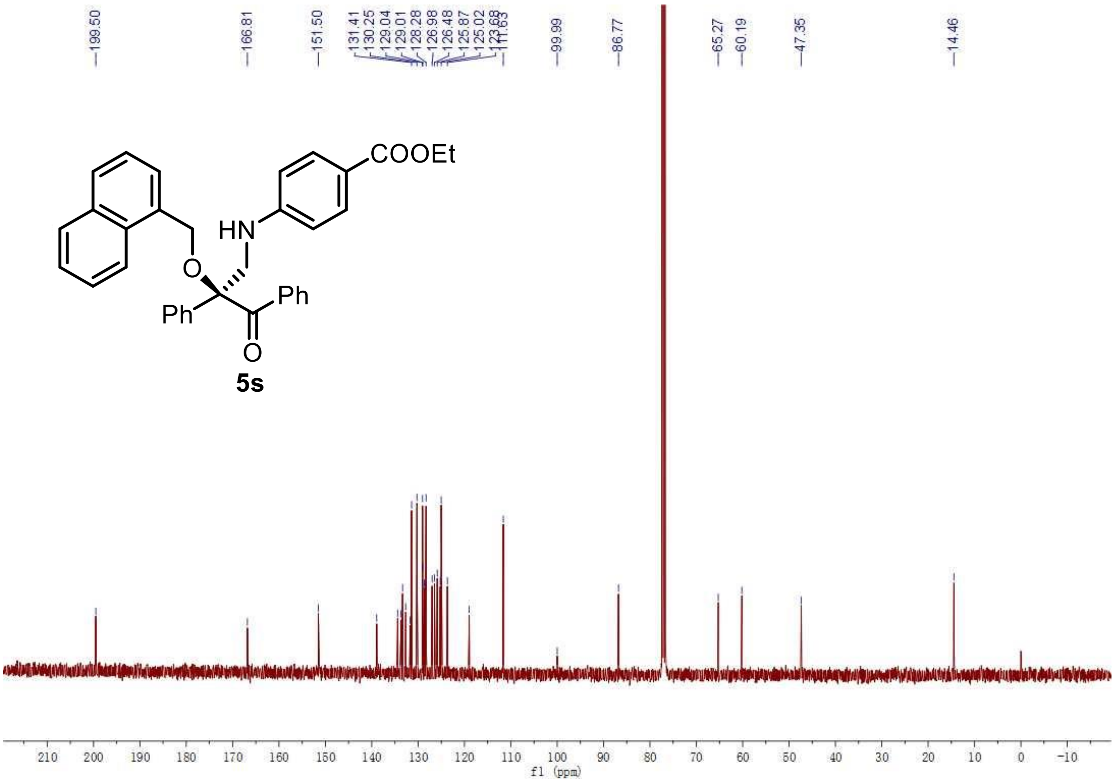

Supplementary Figure 48. ^{1}H NMR spectrum of (*S*)-**5t** (400 MHz, $CDCl_3$)

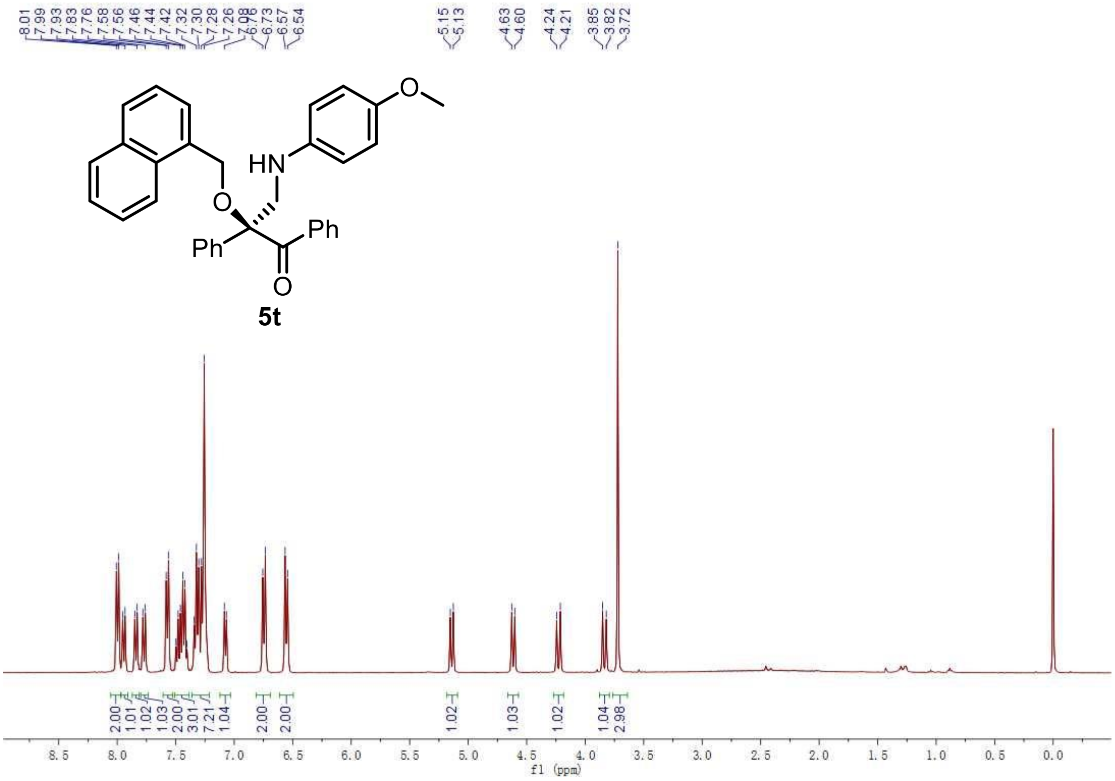

Supplementary Figure 49. ^{13}C NMR spectrum of (*S*)-**5t** (100 MHz, $CDCl_3$)

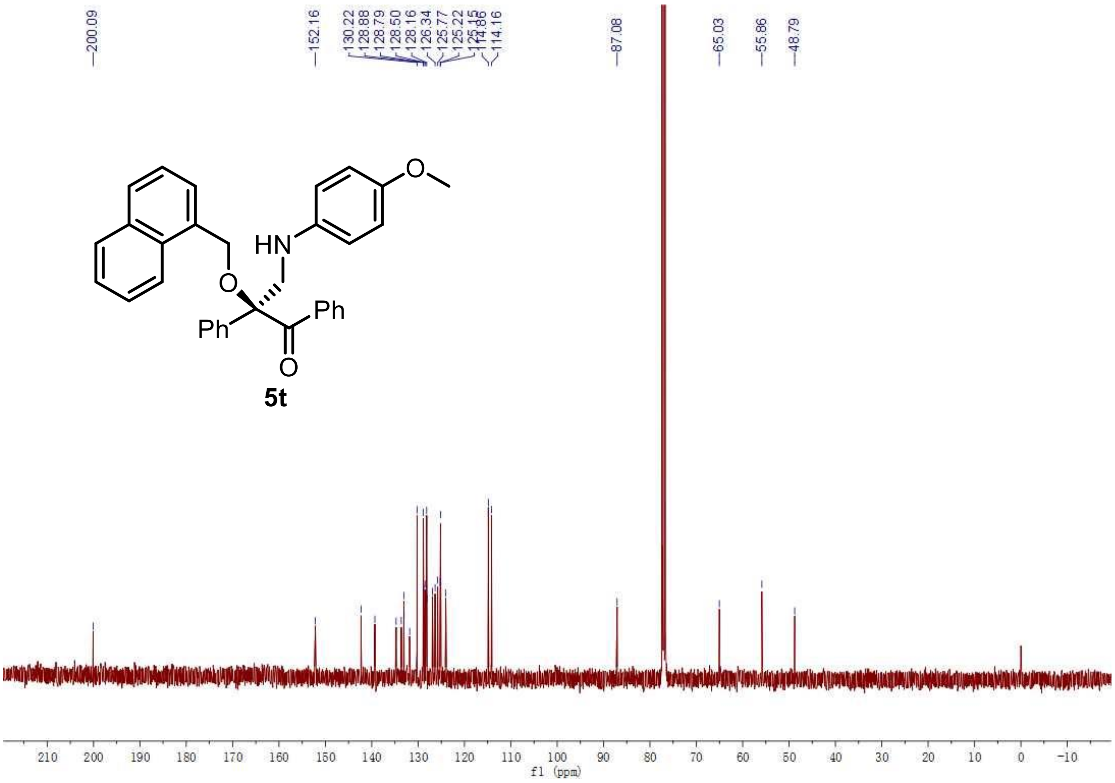

Supplementary Figure 50. ^{1}H NMR spectrum of (*S*)-**5u** (400 MHz, $CDCl_3$)

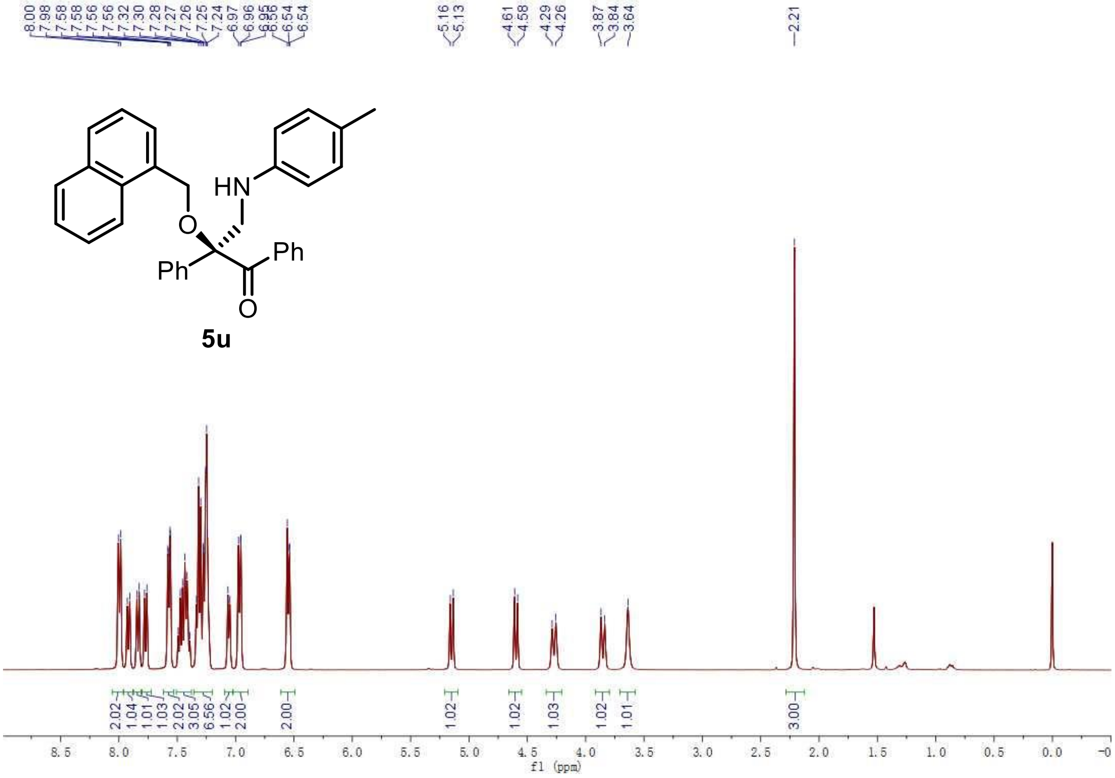

Supplementary Figure 51. ^{13}C NMR spectrum of (*S*)-**5u** (100 MHz, $CDCl_3$)

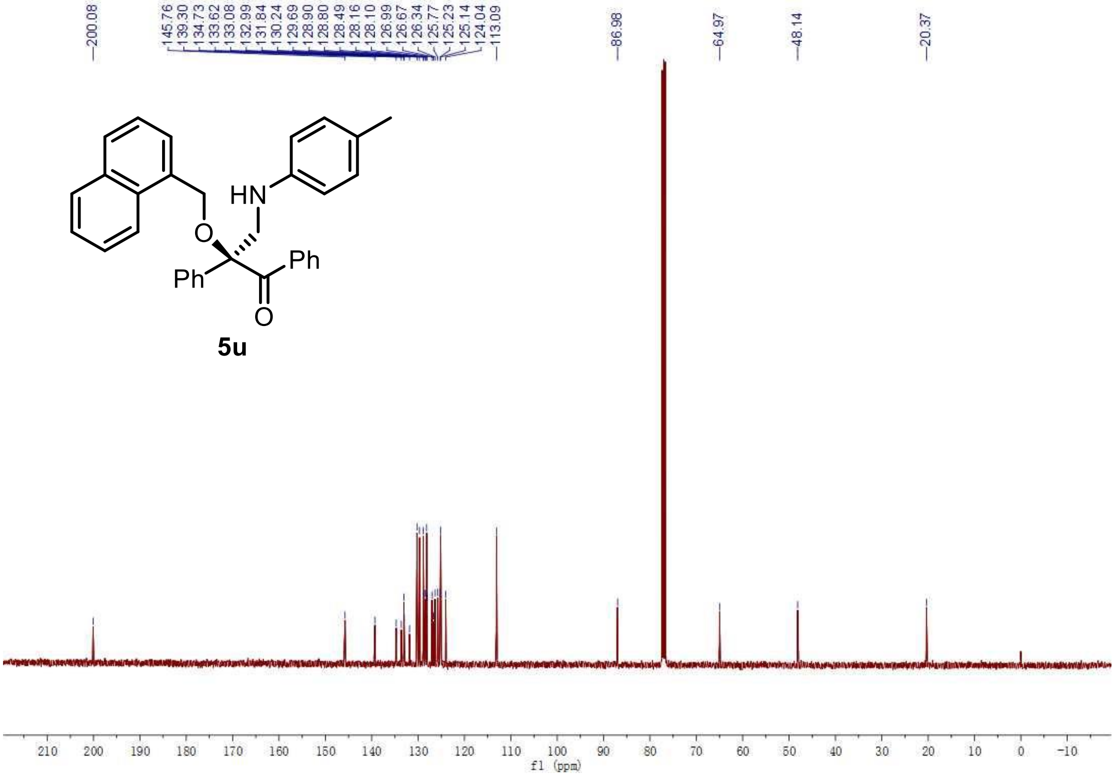

Supplementary Figure 52. ^{1}H NMR spectrum of (*S*)-**5v** (400 MHz, $CDCl_3$)

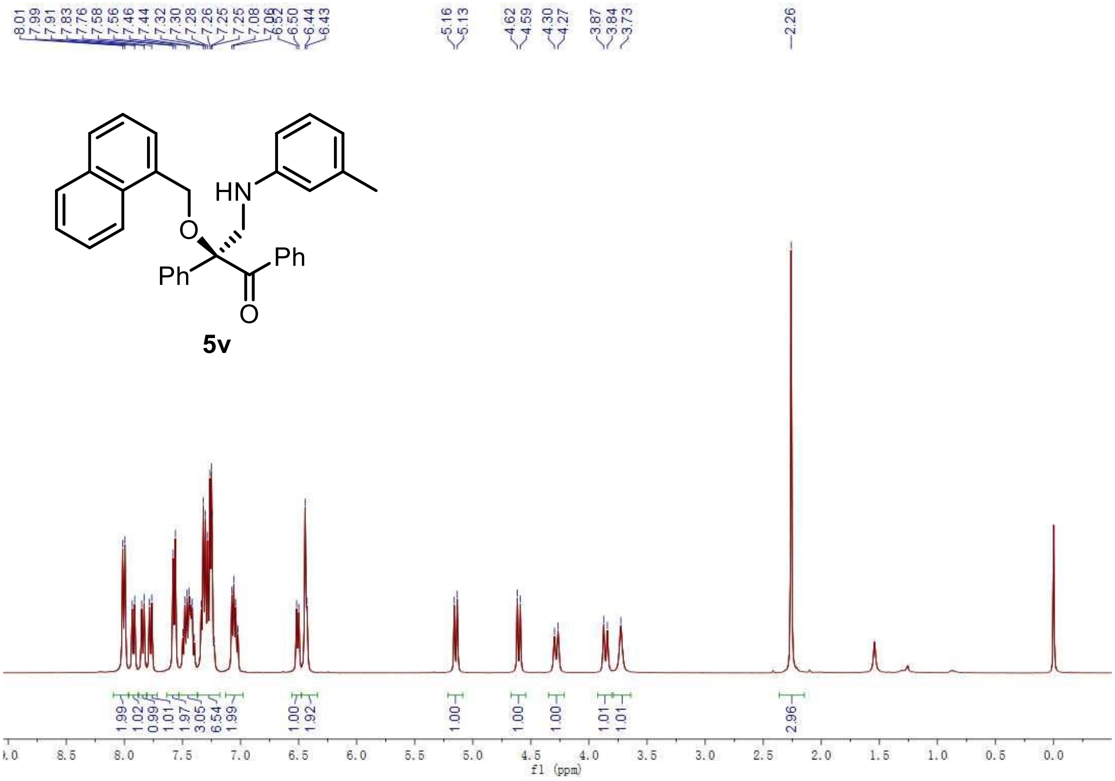

Supplementary Figure 53. ^{13}C NMR spectrum of (*S*)-**5v** (100 MHz, $CDCl_3$)

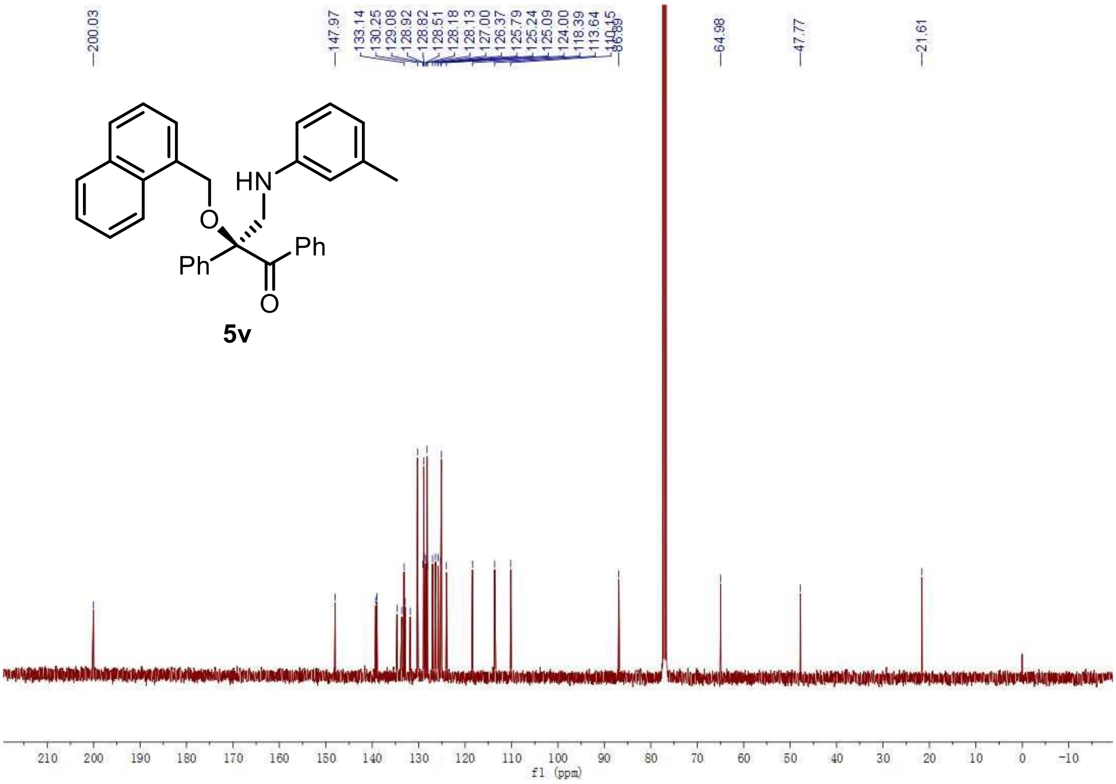

Supplementary Figure 54. ^{1}H NMR spectrum of (*S*)-**5w** (400 MHz, $CDCl_3$)

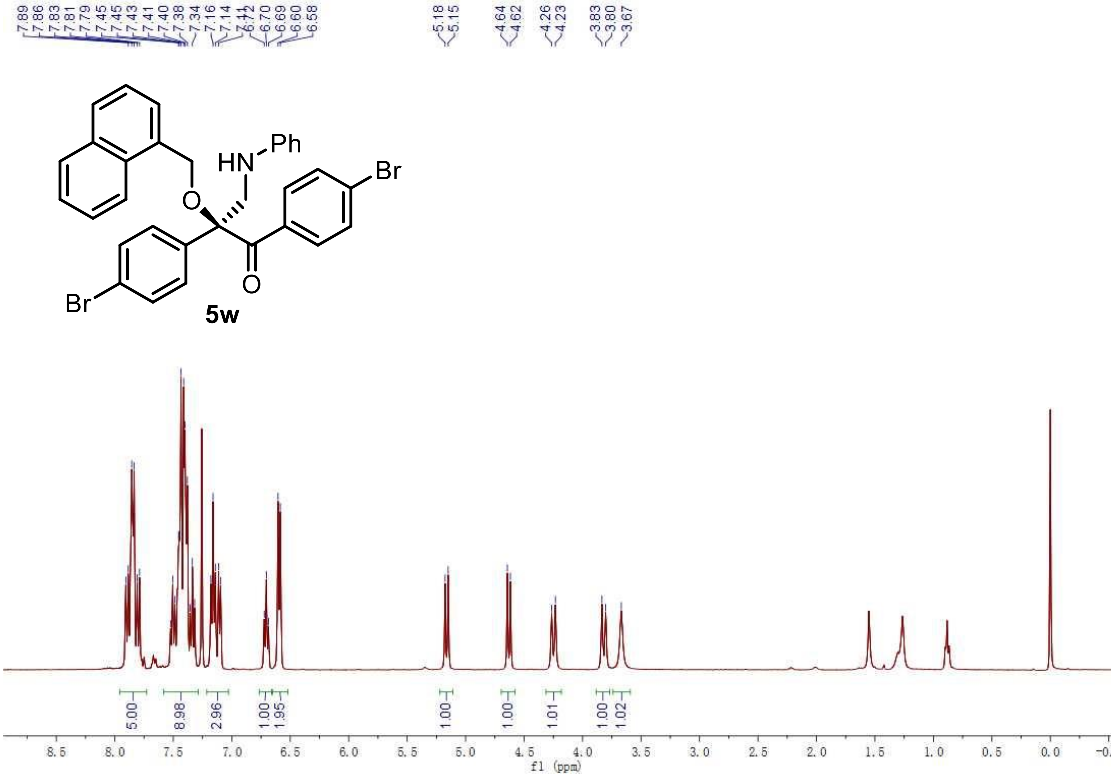

Supplementary Figure 55. ^{13}C NMR spectrum of (*S*)-**5w** (100 MHz, $CDCl_3$)

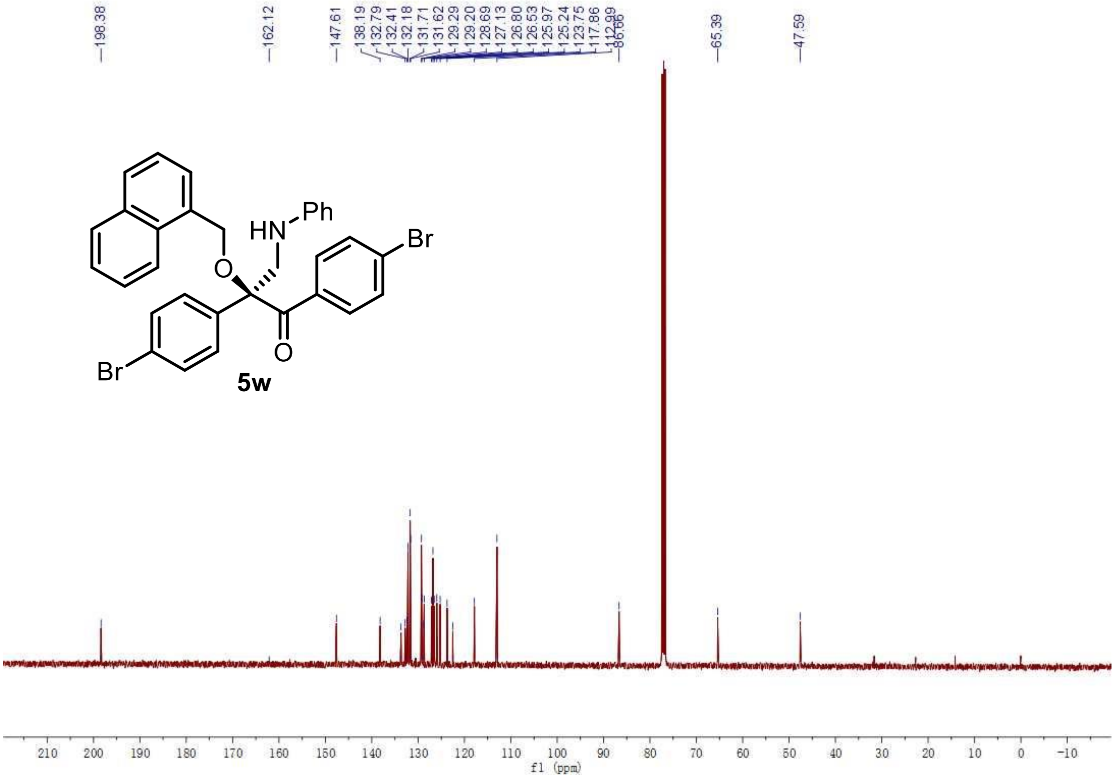

Supplementary Figure 56. ^{1}H NMR spectrum of (*S*)-**5x** (400 MHz, $CDCl_3$)

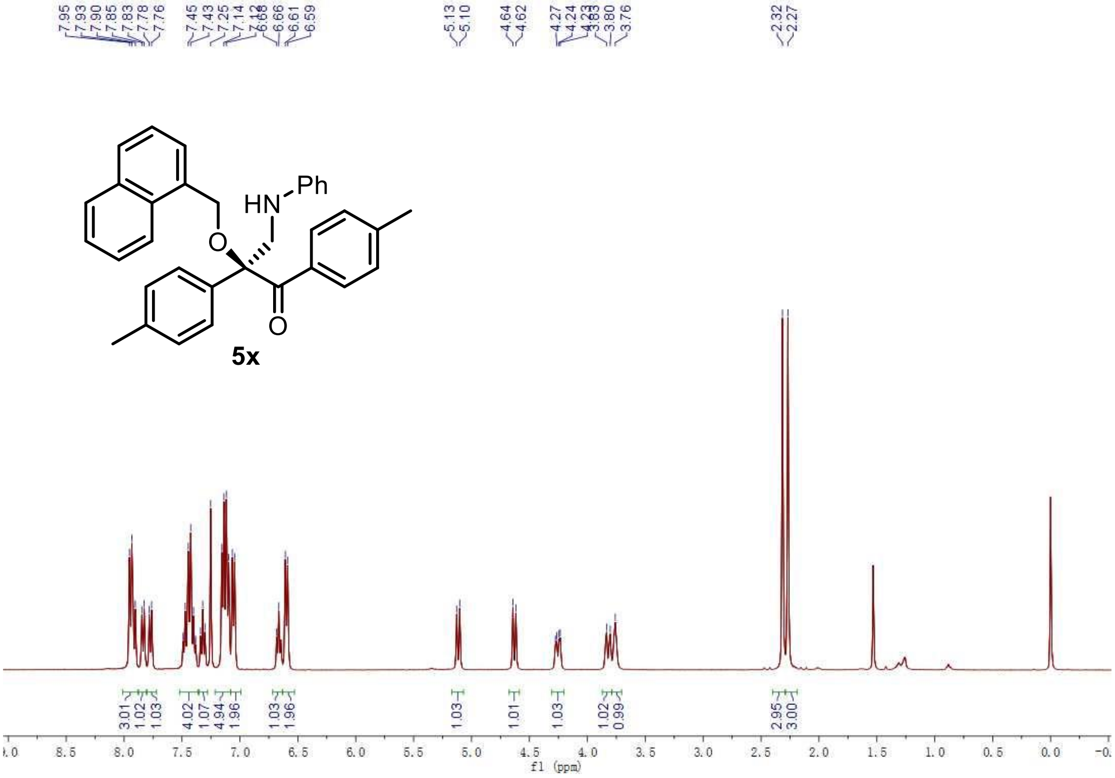

Supplementary Figure 57. ^{13}C NMR spectrum of (*S*)-**5x** (100 MHz, $CDCl_3$)

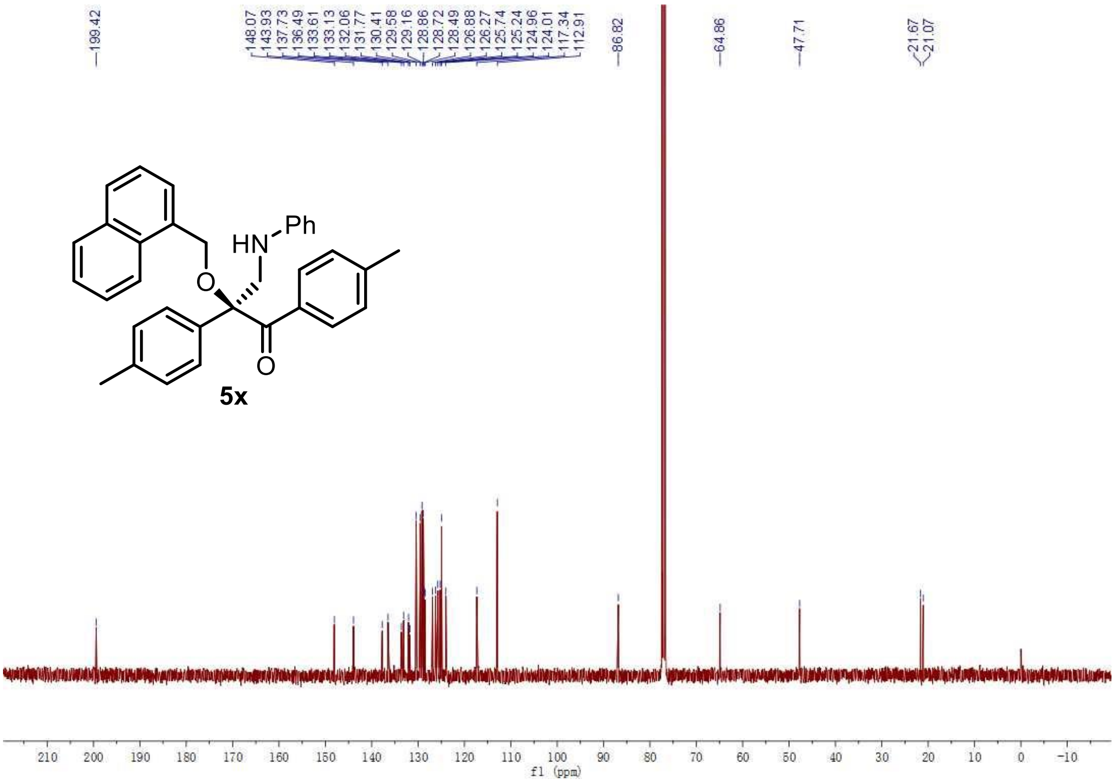

Supplementary Figure 58. ^{1}H NMR spectrum of (*S*)-**4a** (400 MHz, $CDCl_3$)

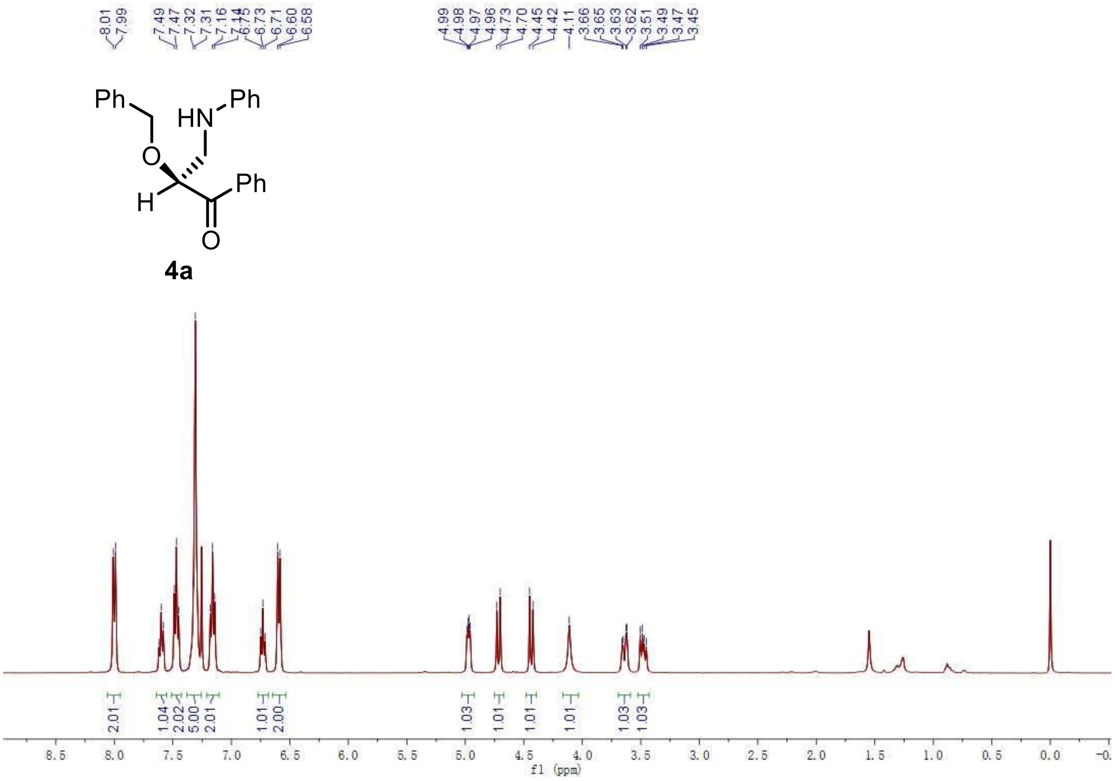

Supplementary Figure 59. ^{13}C NMR spectrum of (*S*)-**4a** (100 MHz, $CDCl_3$)

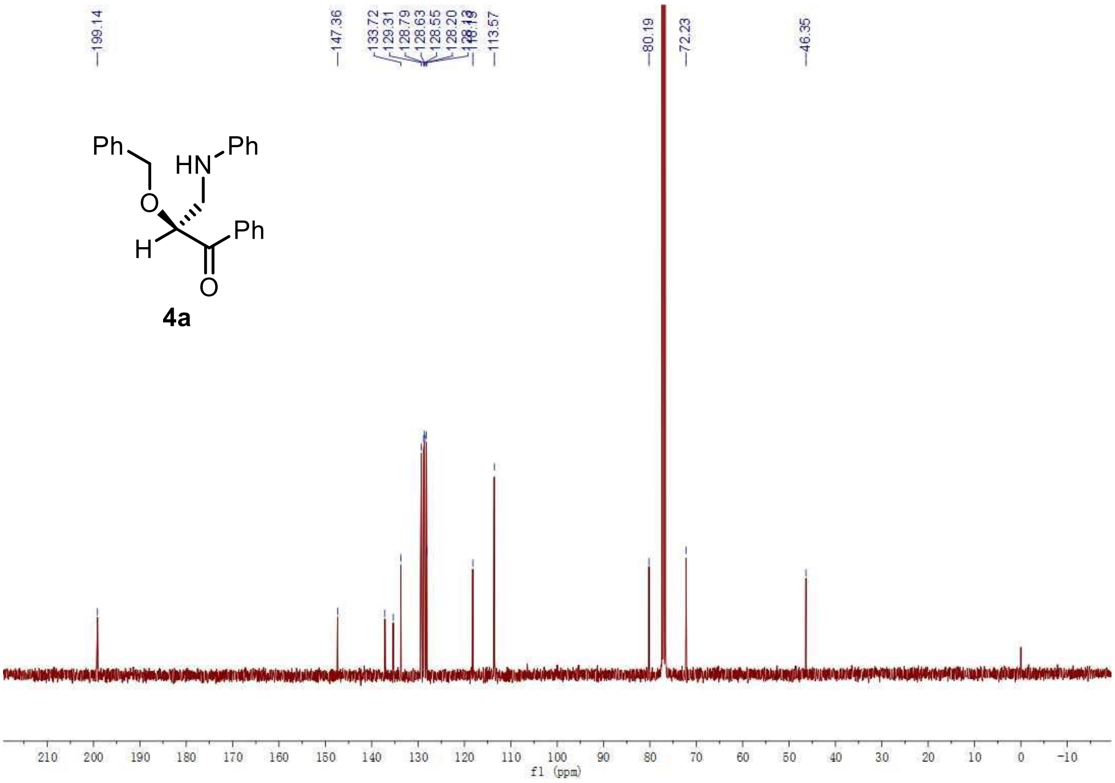

Supplementary Figure 60. ^{1}H NMR spectrum of (*S*)-**4b** (400 MHz, $CDCl_3$)

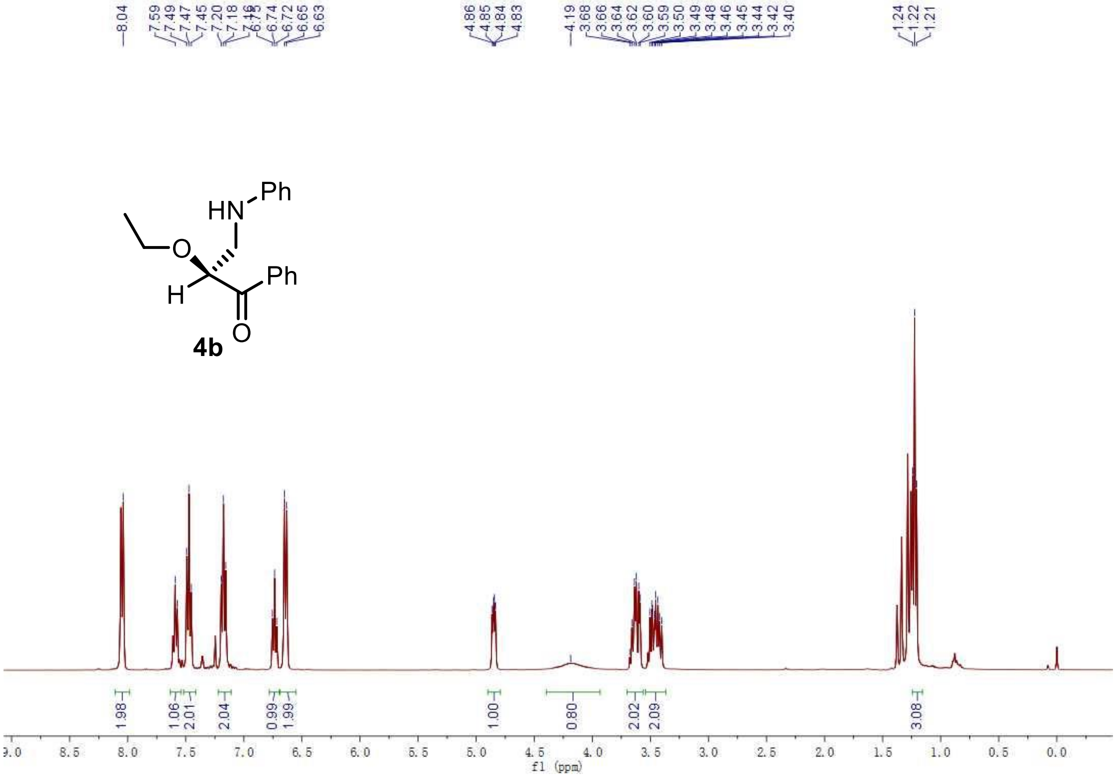

Supplementary Figure 61. ^{13}C NMR spectrum of (*S*)-**4a** (100 MHz, $CDCl_3$)

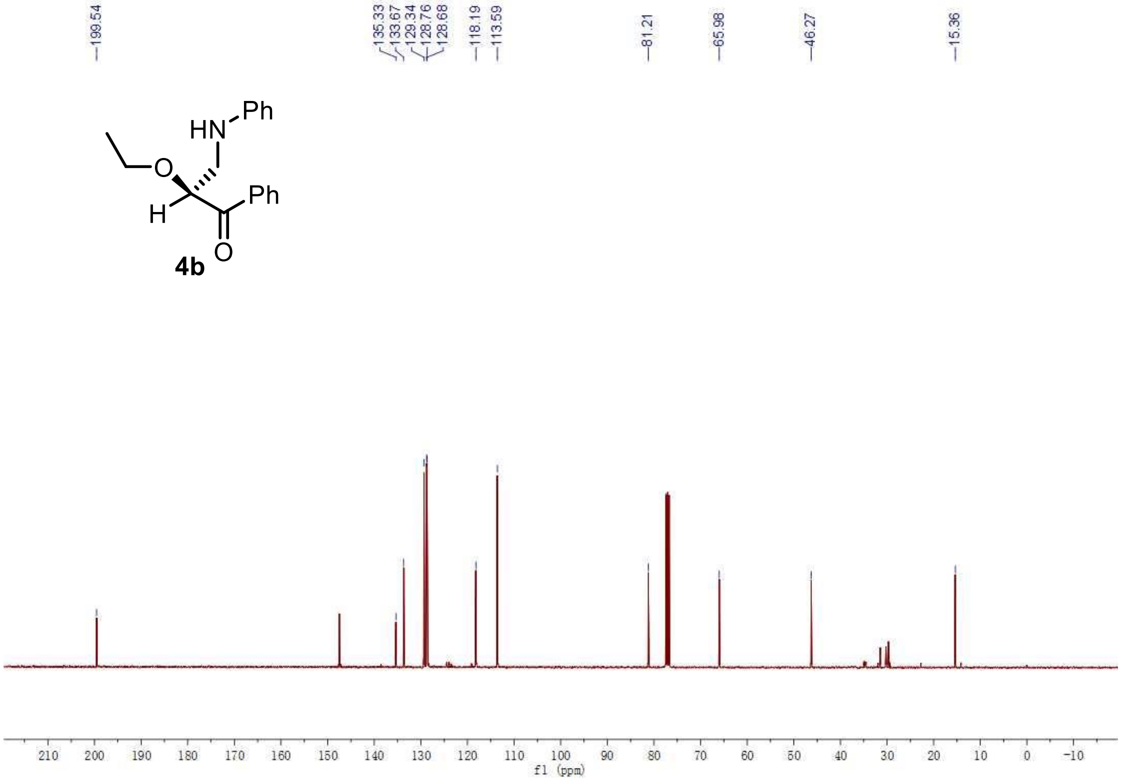

Supplementary Figure 62. ^{1}H NMR spectrum of (*S*)-**4c** (400 MHz, $CDCl_3$)

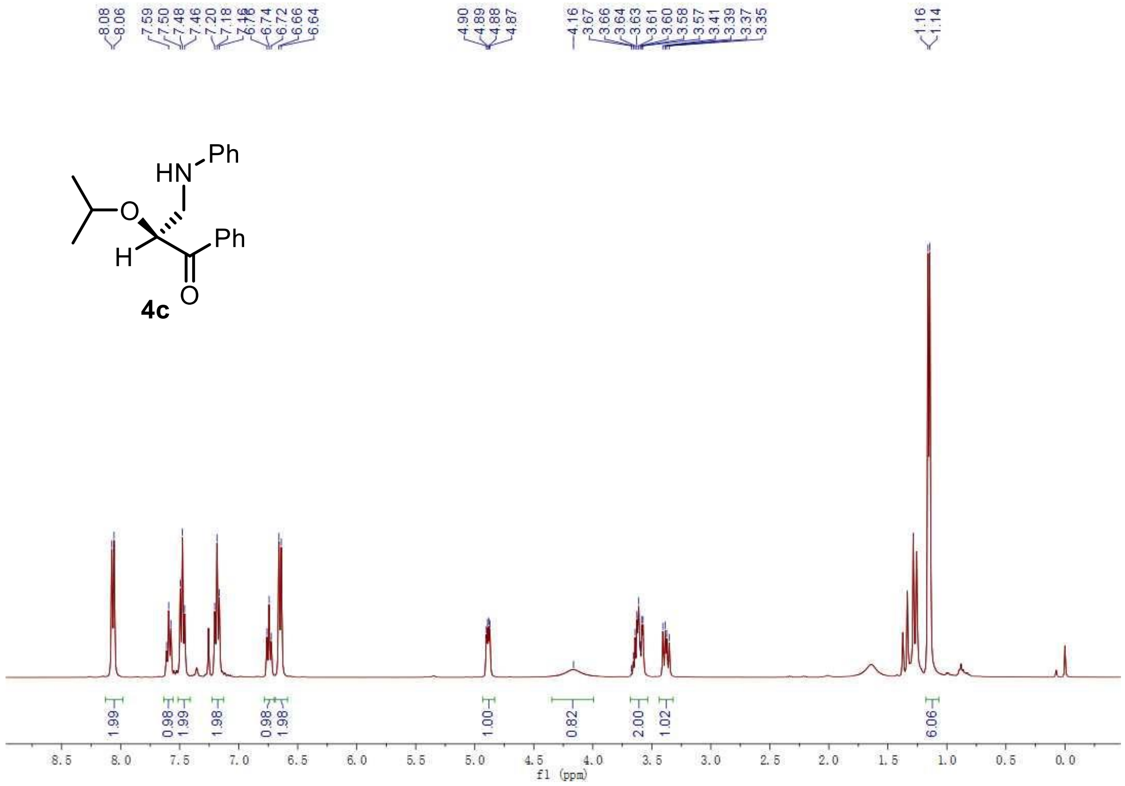

Supplementary Figure 63. ^{13}C NMR spectrum of (*S*)-**4c** (100 MHz, $CDCl_3$)

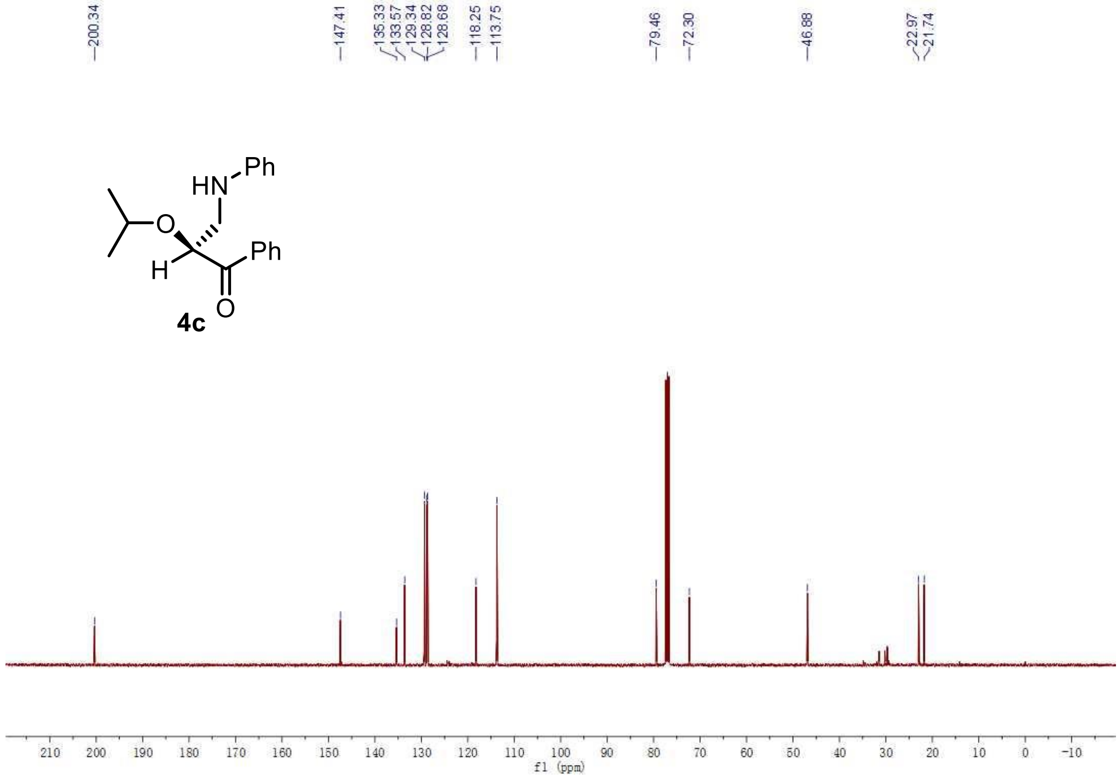

Supplementary Figure 64. ^{1}H NMR spectrum of (*S*)-**4d** (400 MHz, $CDCl_3$)

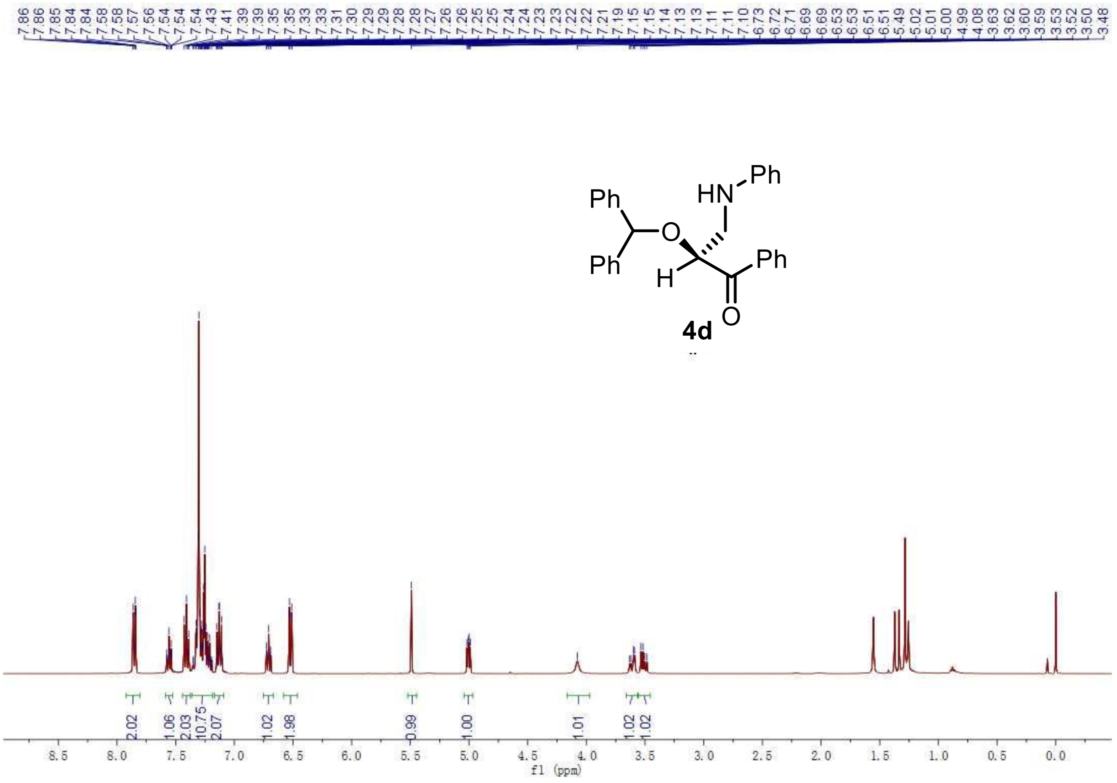

Supplementary Figure 65. ^{13}C NMR spectrum of (*S*)-**4d** (100 MHz, $CDCl_3$)

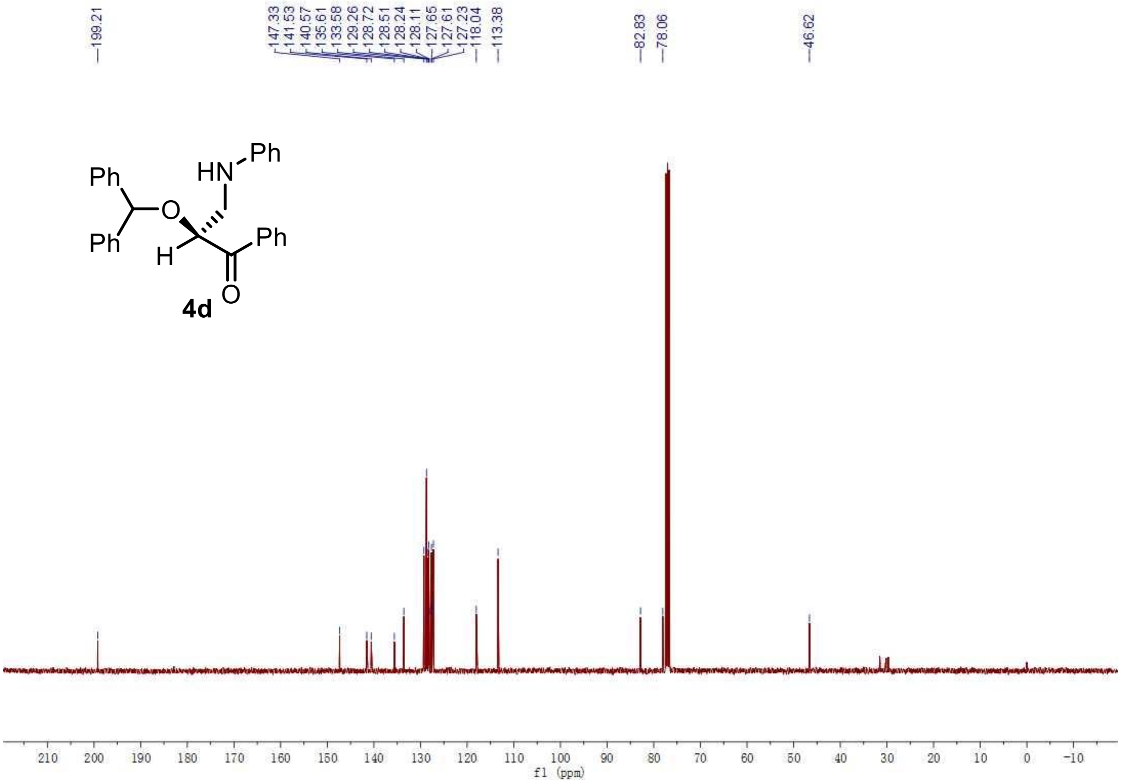

Supplementary Figure 66. ^{1}H NMR spectrum of (*S*)-**4e** (400 MHz, $CDCl_3$)

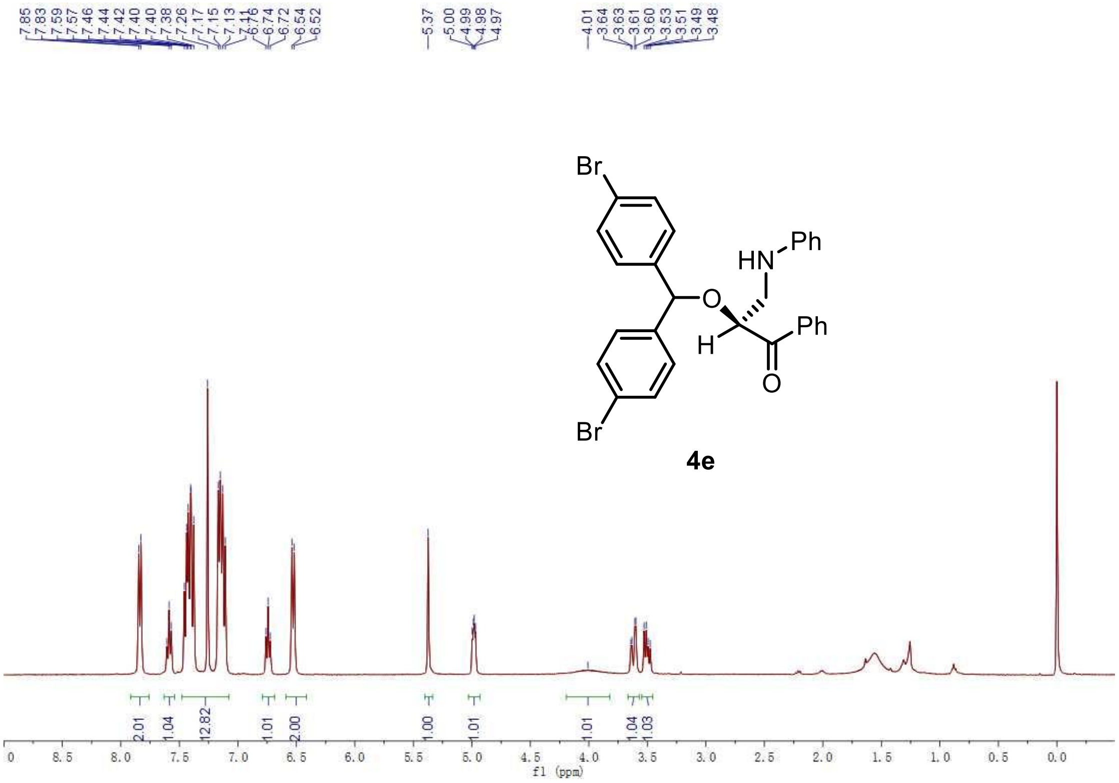

Supplementary Figure 67. ^{13}C NMR spectrum of (*S*)-**4e** (100 MHz, $CDCl_3$)

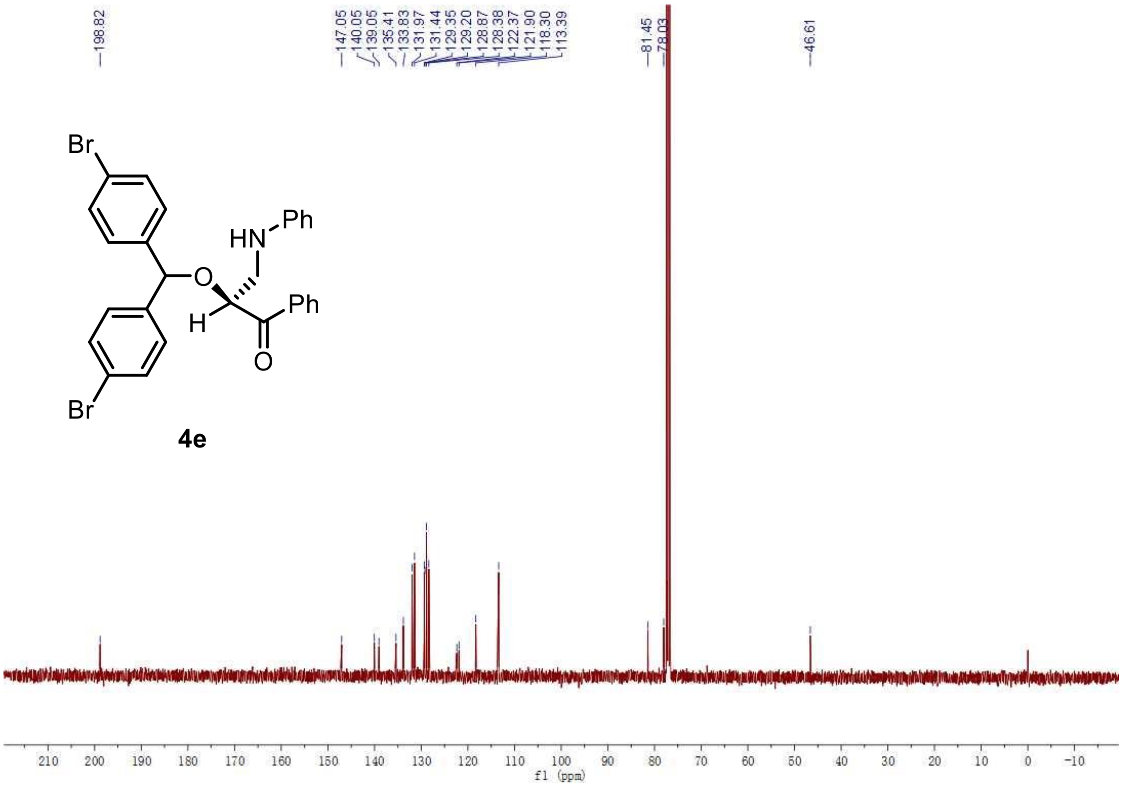

Supplementary Figure 68. ^{1}H NMR spectrum of (*S*)-**4f** (400 MHz, $CDCl_3$)

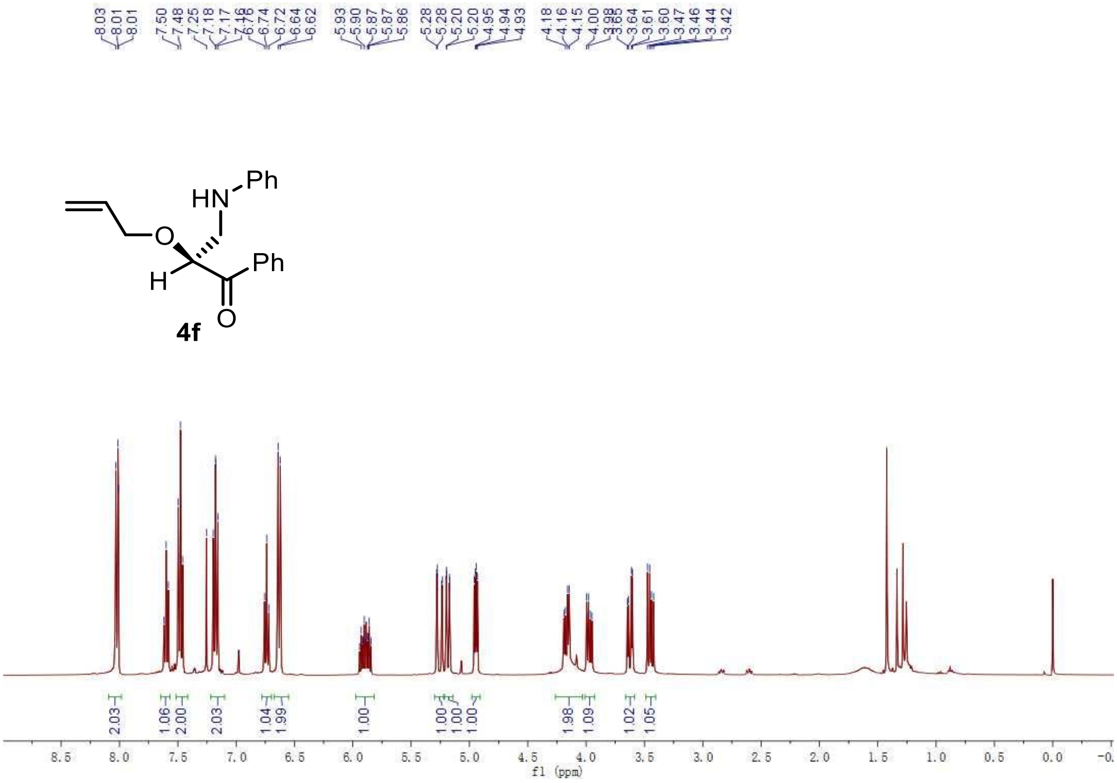

Supplementary Figure 69. ^{13}C NMR spectrum of (*S*)-**4f** (100 MHz, $CDCl_3$)

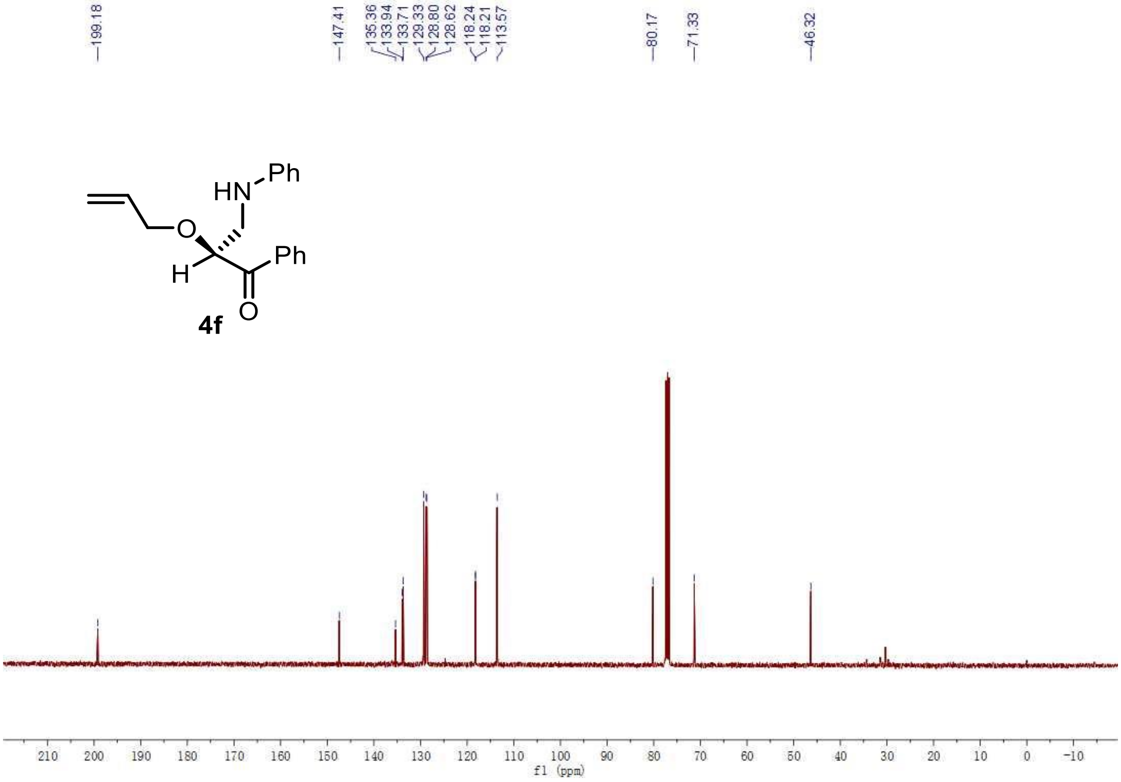

Supplementary Figure 70. ^{1}H NMR spectrum of (*S*)-**4g** (400 MHz, $CDCl_3$)

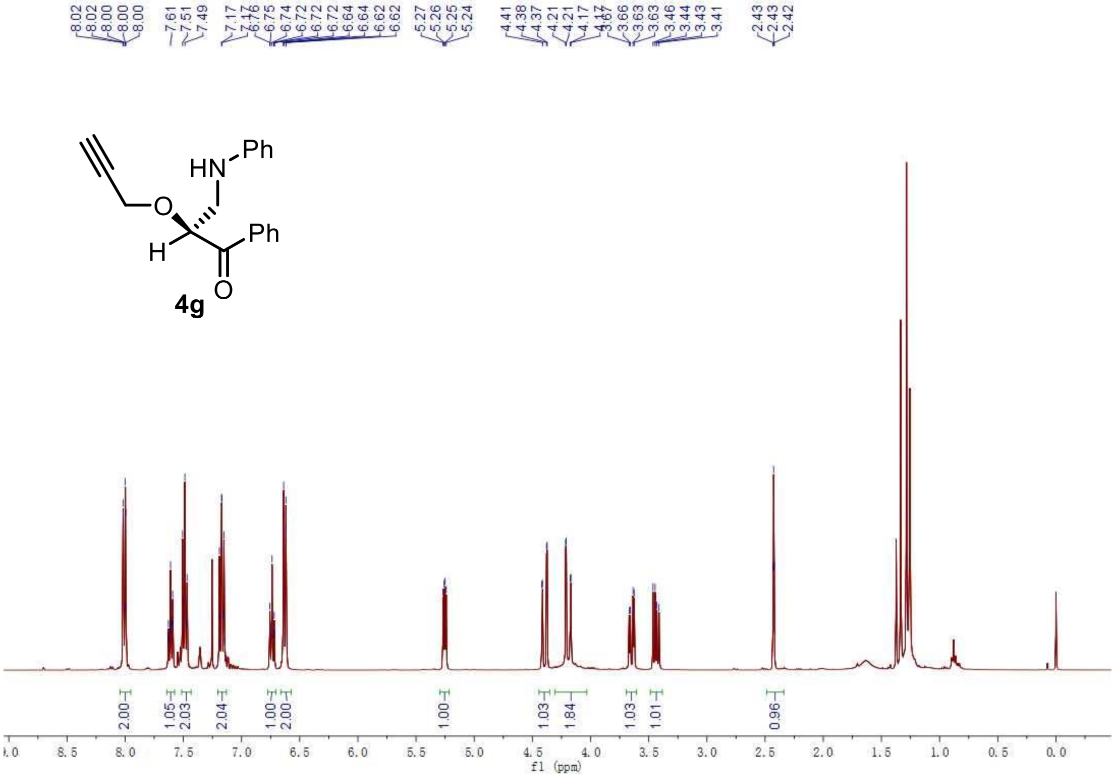

Supplementary Figure 71. ^{13}C NMR spectrum of (*S*)-**4g** (100 MHz, $CDCl_3$)

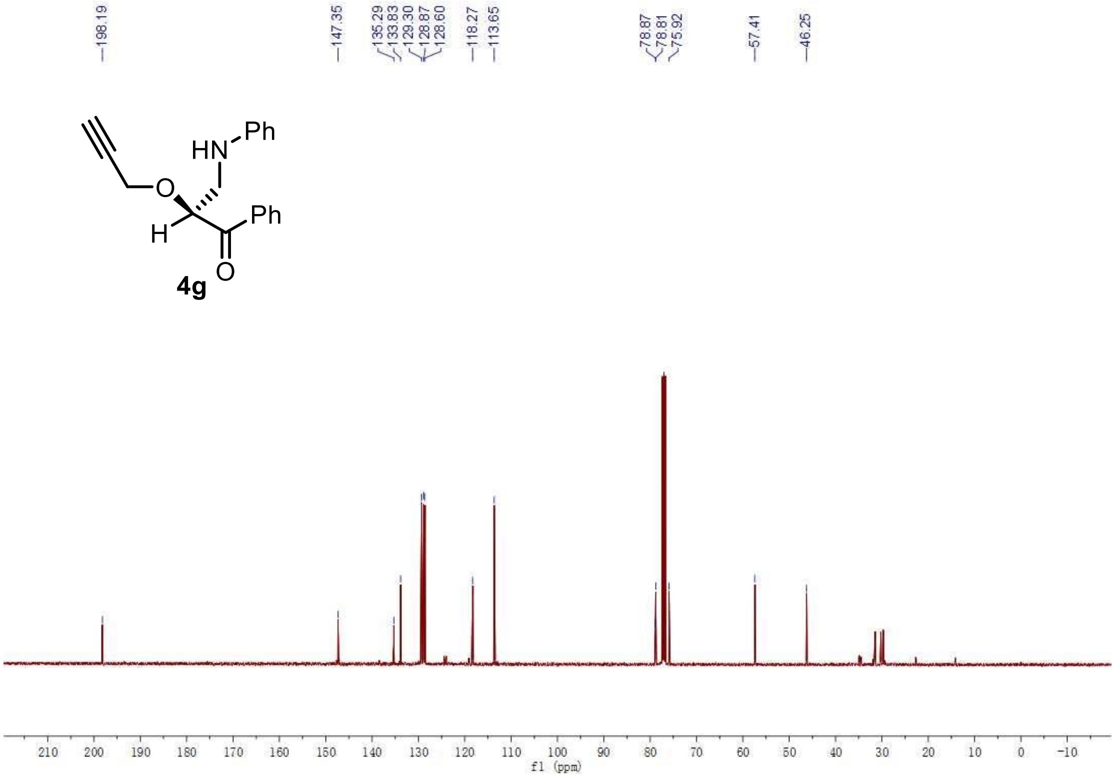

Supplementary Figure 72. ^{1}H NMR spectrum of (*S*)-**4h** (400 MHz, $CDCl_3$)

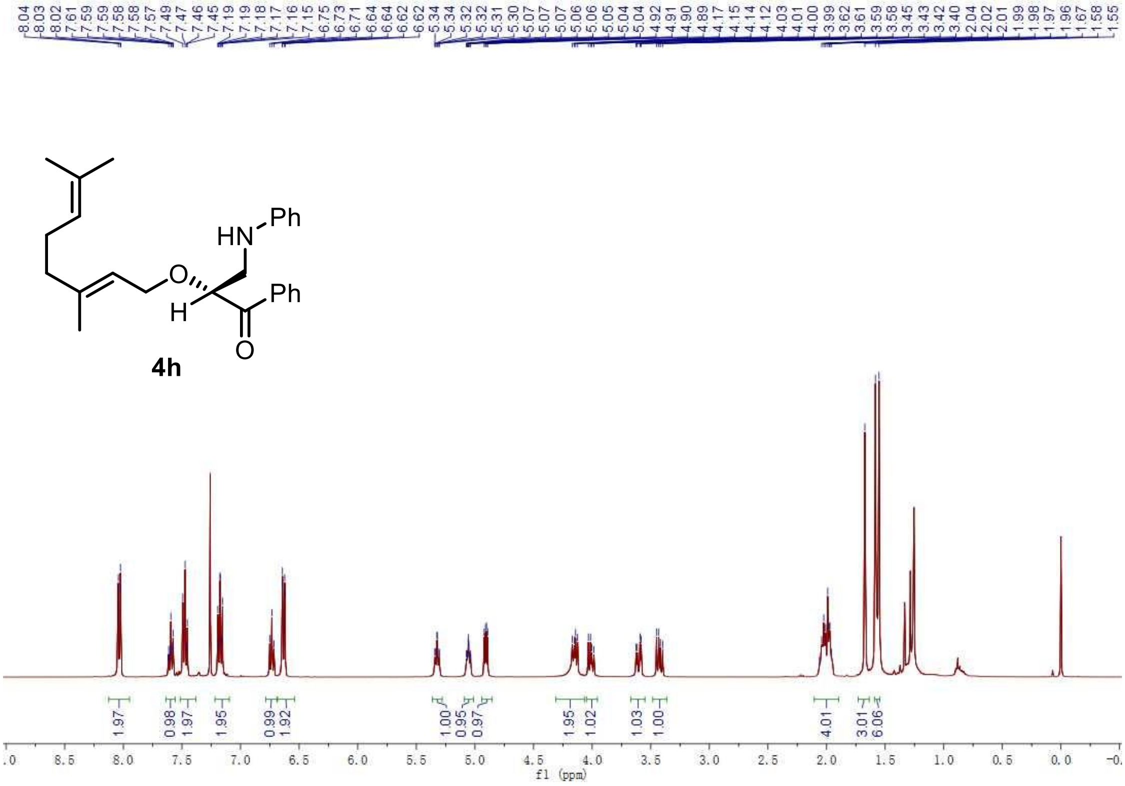

Supplementary Figure 73. ^{13}C NMR spectrum of (*S*)-**4h** (100 MHz, $CDCl_3$)

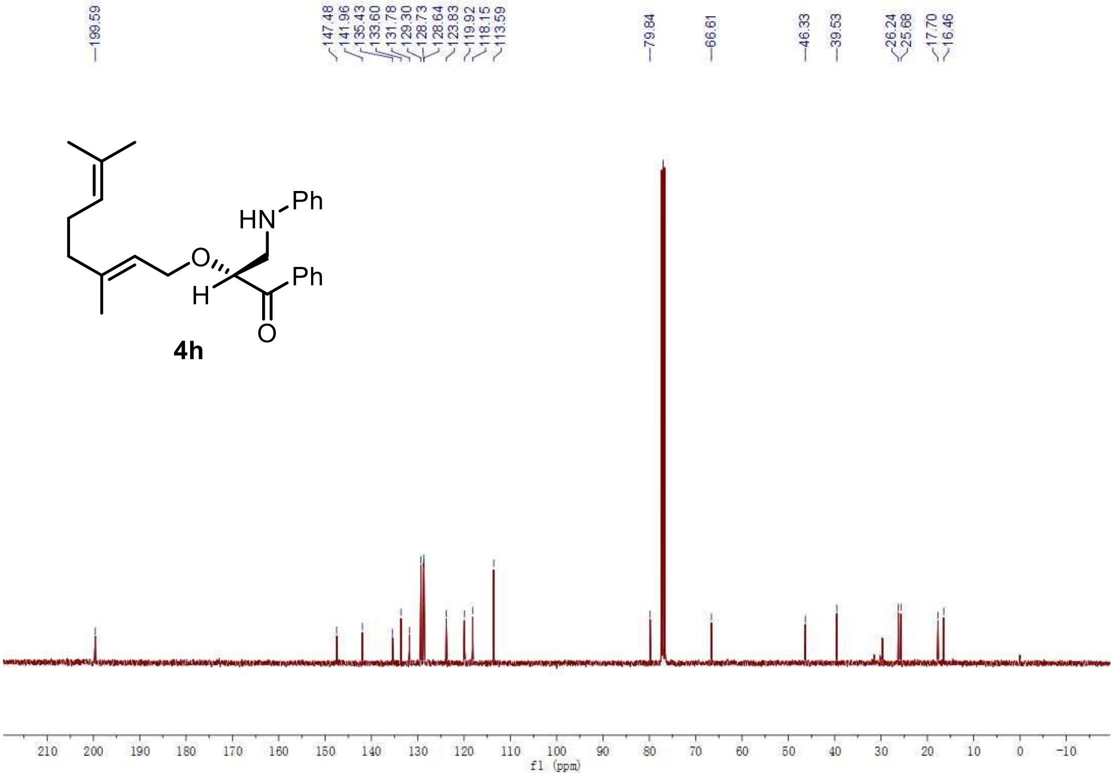

Supplementary Figure 74. ^{1}H NMR spectrum of (*S*)-**4i** (400 MHz, $CDCl_3$)

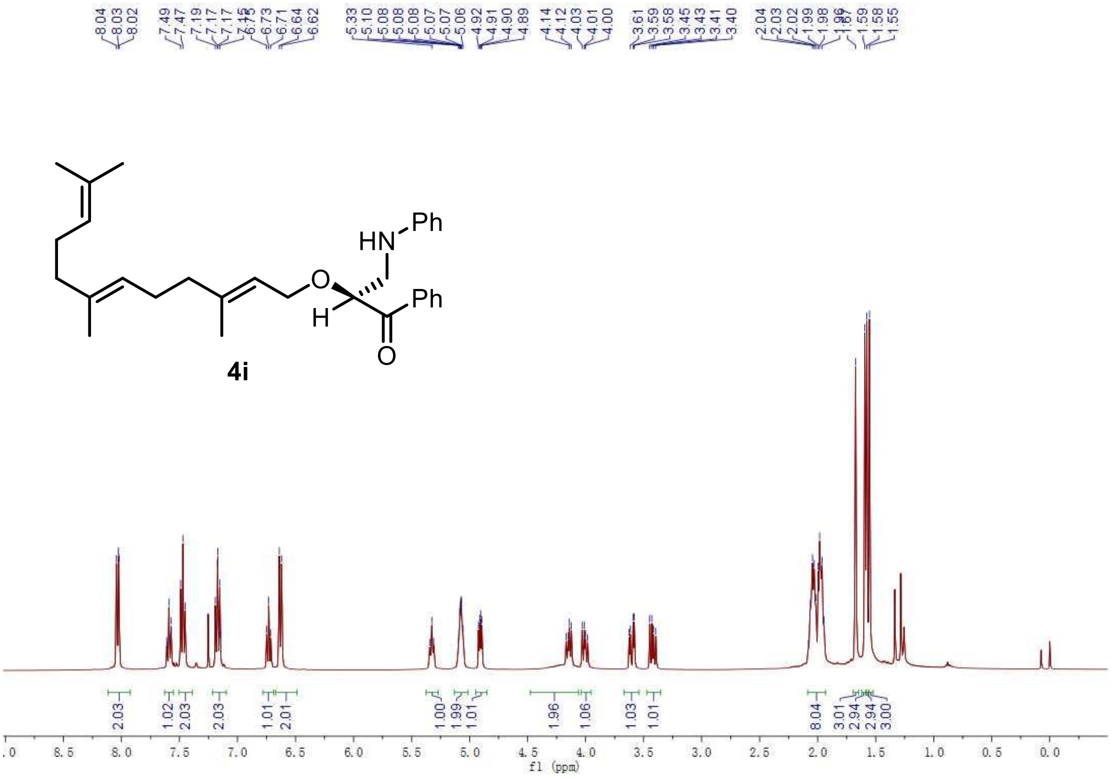

Supplementary Figure 75. ^{13}C NMR spectrum of (*S*)-**4i** (100 MHz, $CDCl_3$)

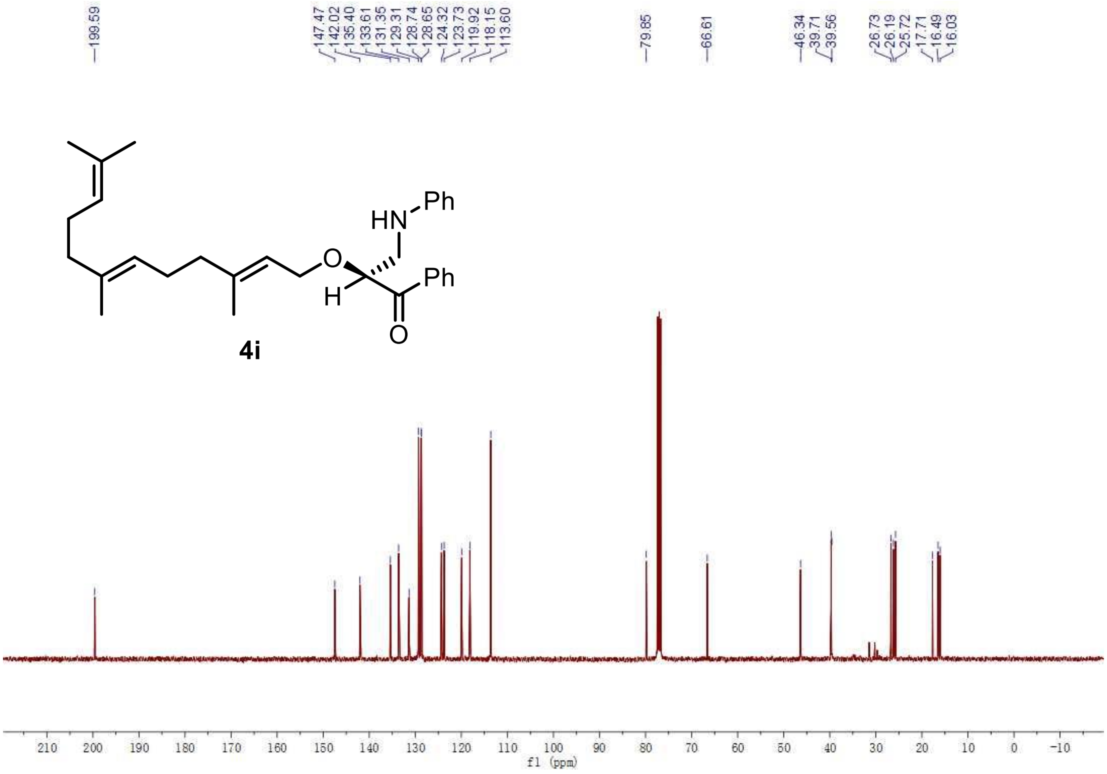

Supplementary Figure 76. ^{1}H NMR spectrum of (*S*)-**4j** (400 MHz, $CDCl_3$)

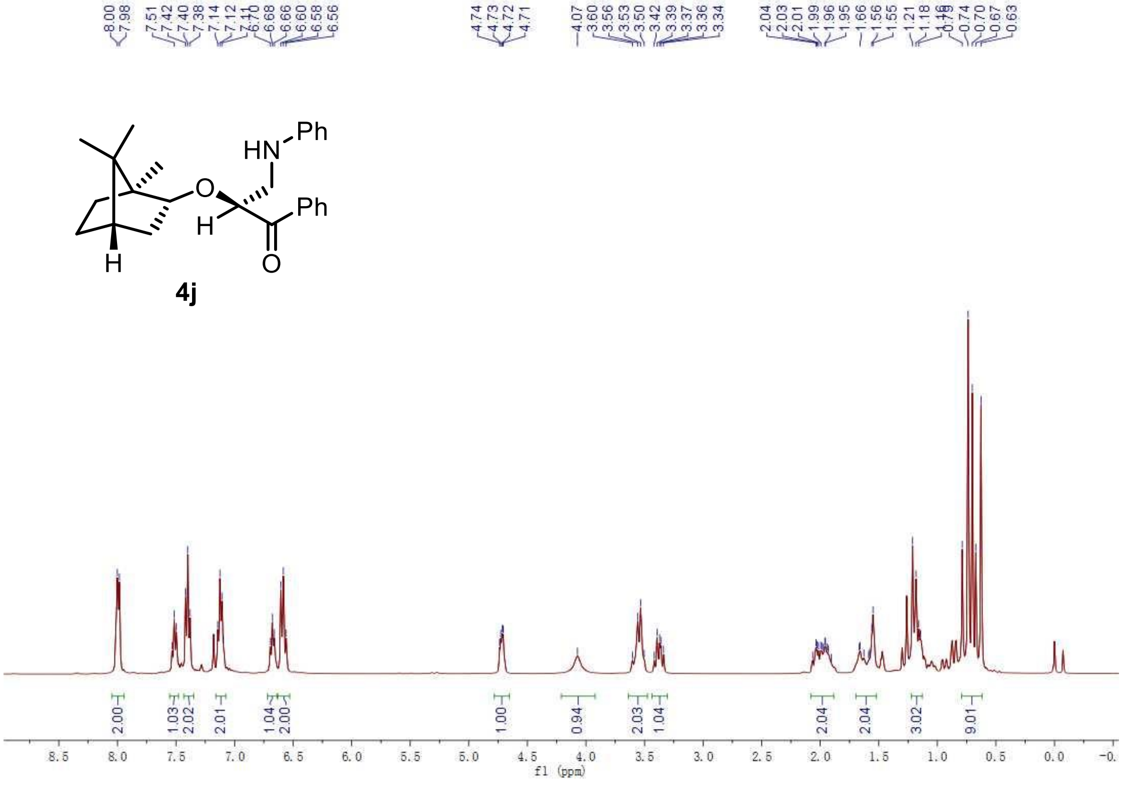

Supplementary Figure 77. ^{13}C NMR spectrum of (*S*)-**4j** (100 MHz, $CDCl_3$)

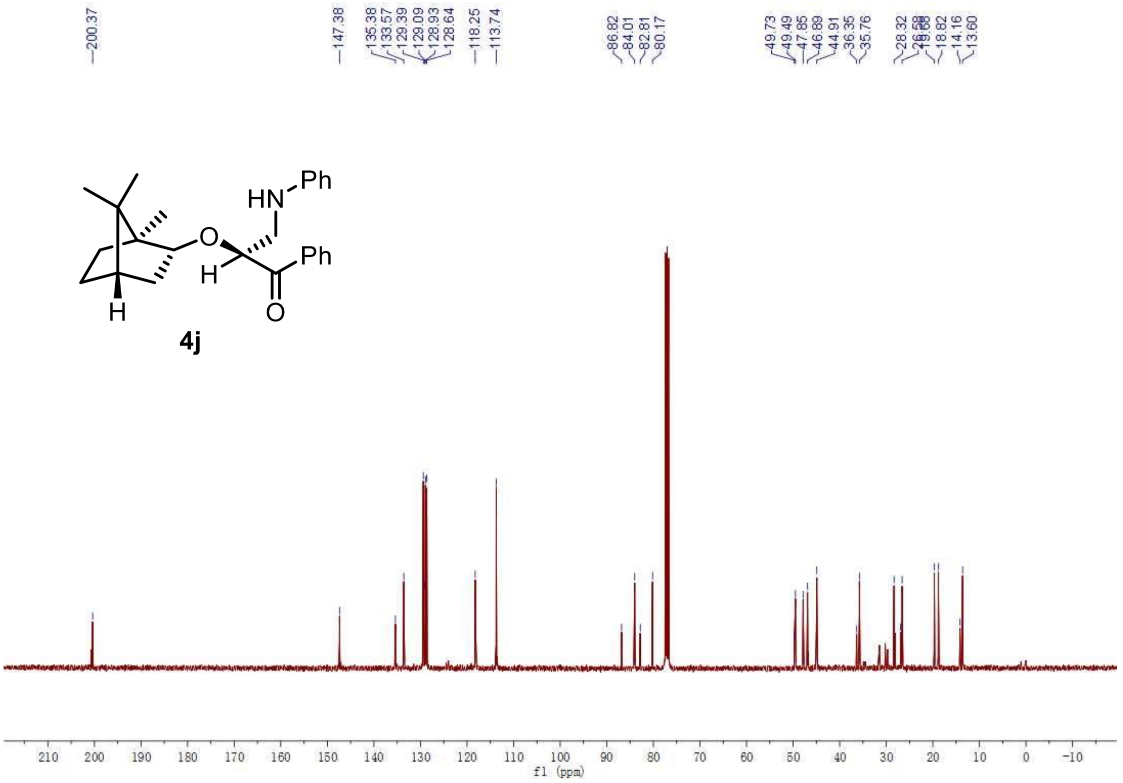

Supplementary Figure 78. ^{1}H NMR spectrum of (*S*)-**4k** (400 MHz, $CDCl_3$)

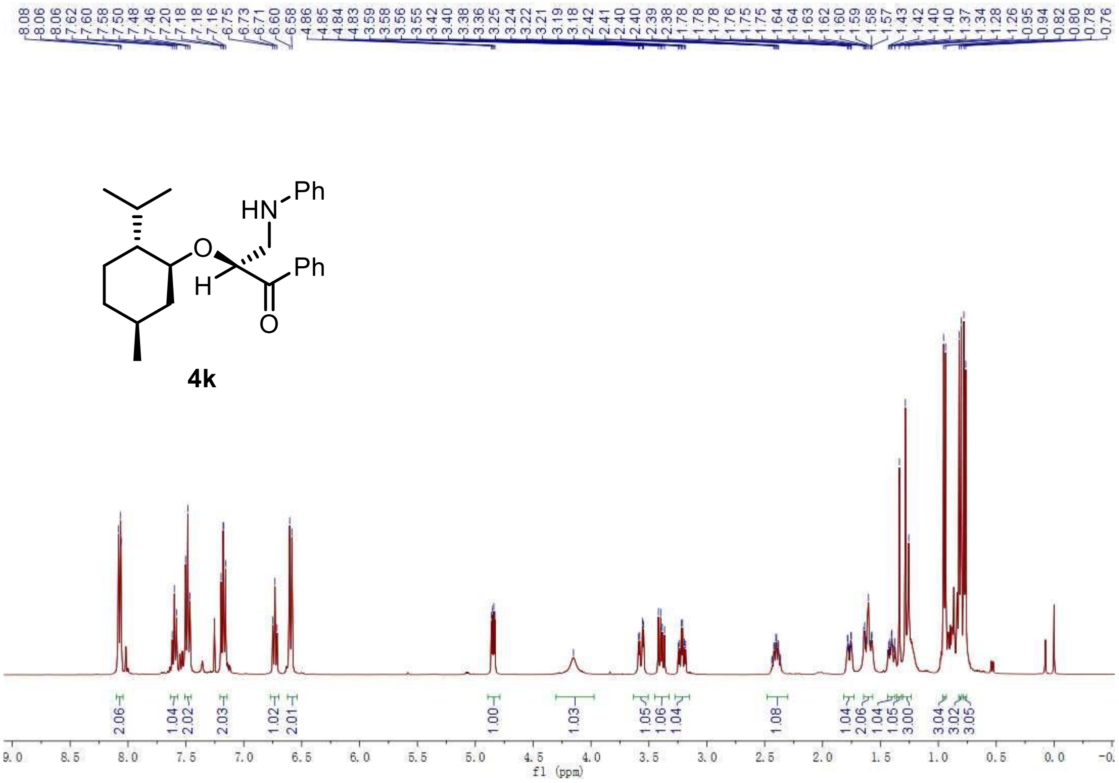

Supplementary Figure 79. ^{1}H NMR spectrum of (*S*)-**4k** 400 MHz, $CDCl_3$)

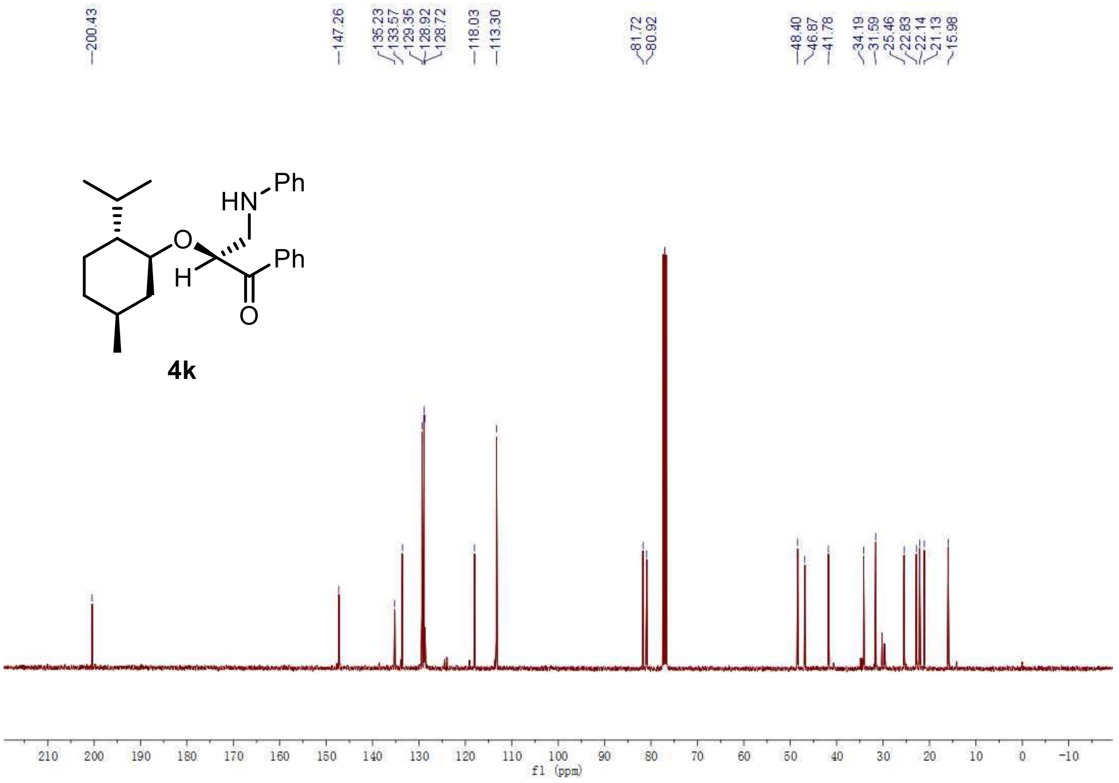

Supplementary Figure 80. ^{1}H NMR spectrum of (*S*)-**4l** 400 MHz, $CDCl_3$)

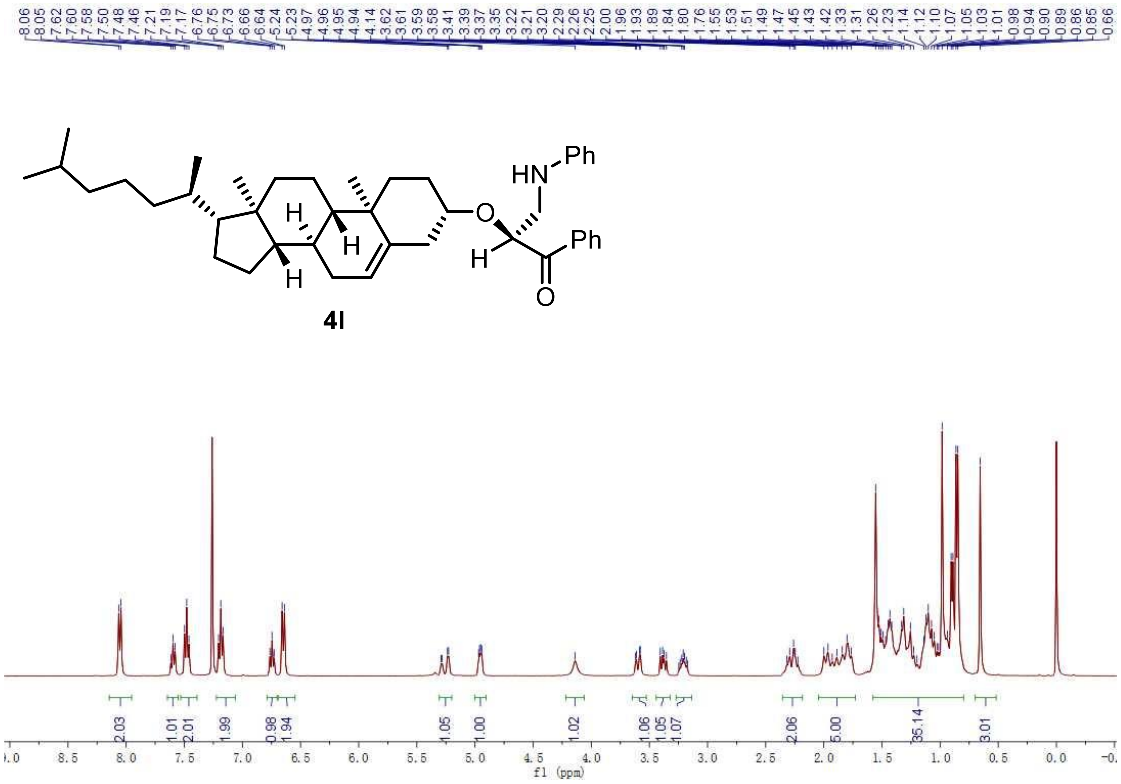

Supplementary Figure 81. ^{13}C NMR spectrum of (*S*)-**4l** (100 MHz, $CDCl_3$)

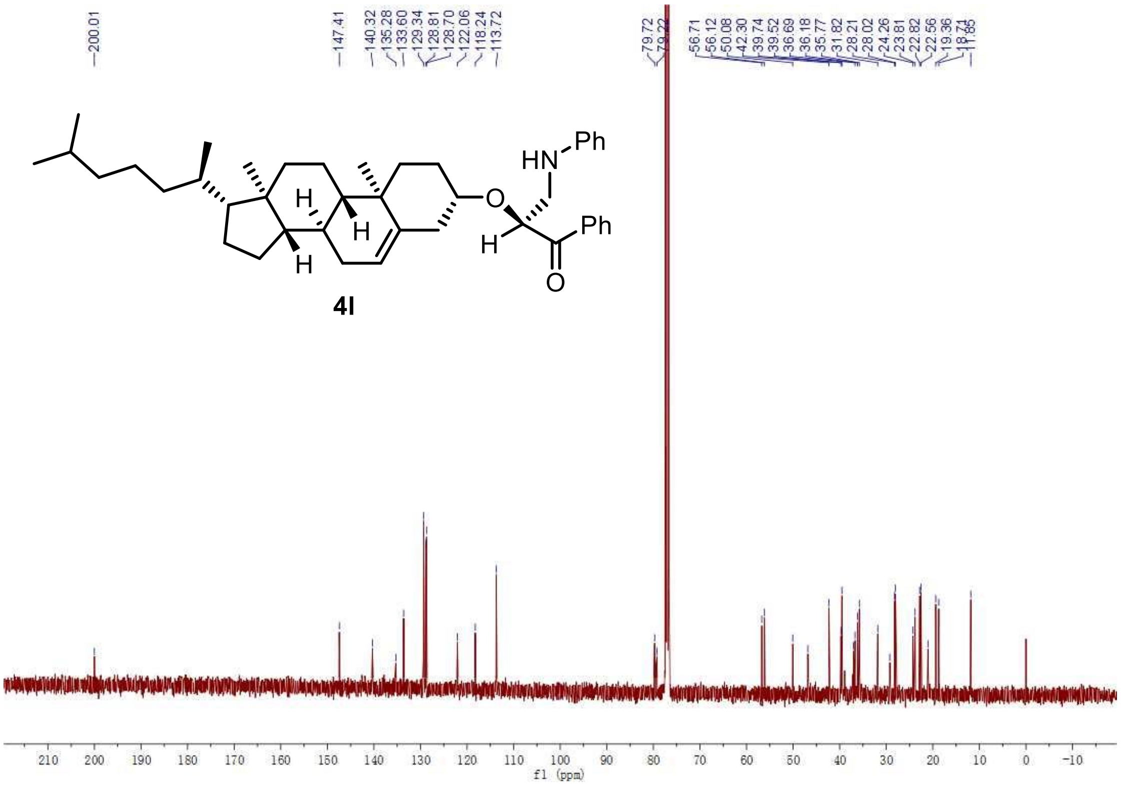

Supplementary Figure 82. ^{1}H NMR spectrum of (*S*)-**4m** (400 MHz, $CDCl_3$)

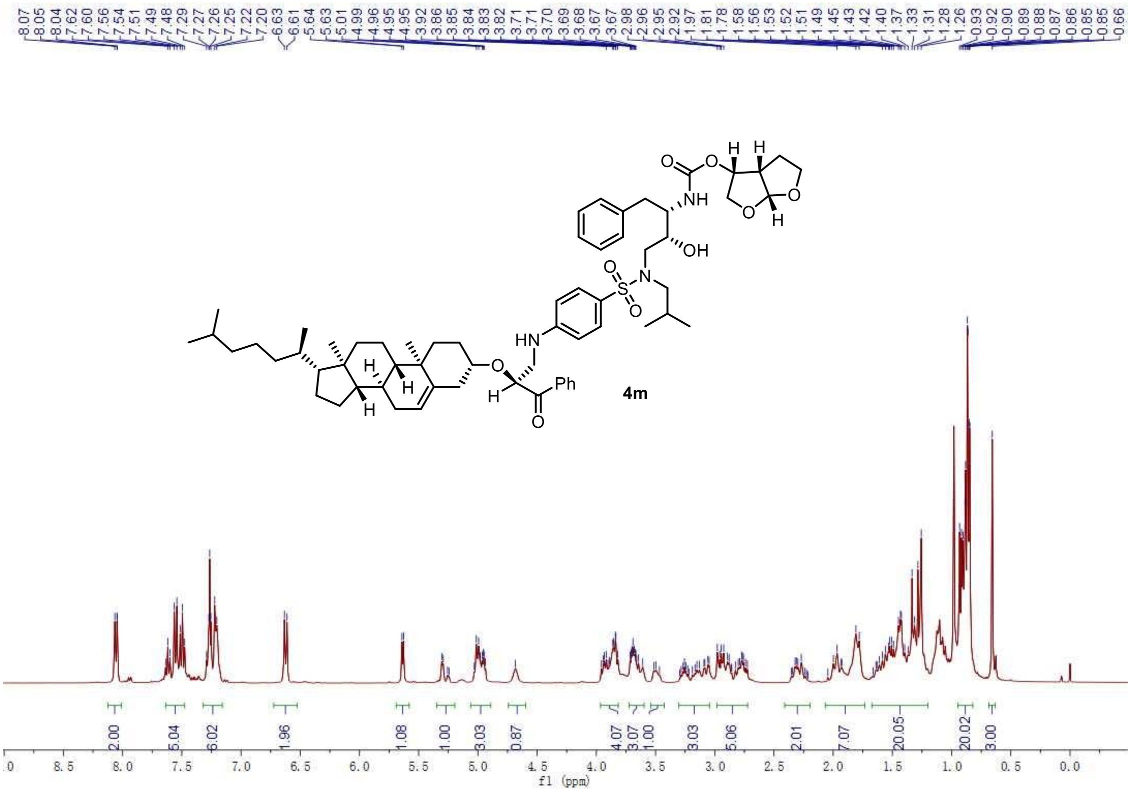

Supplementary Figure 83. ^{13}C NMR spectrum of (*S*)-**4m** (100 MHz, $CDCl_3$)

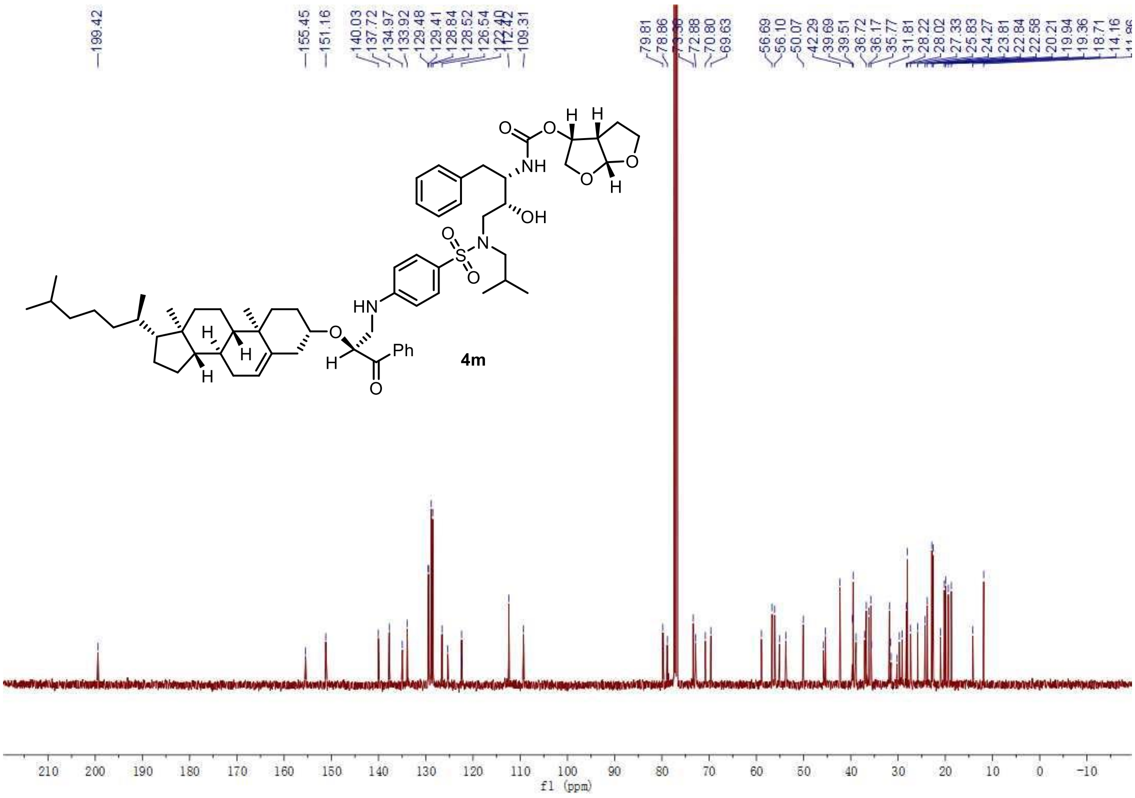

Supplementary Figure 84. ^{1}H NMR spectrum of (*S*)-**4n** 400 MHz, $CDCl_3$)

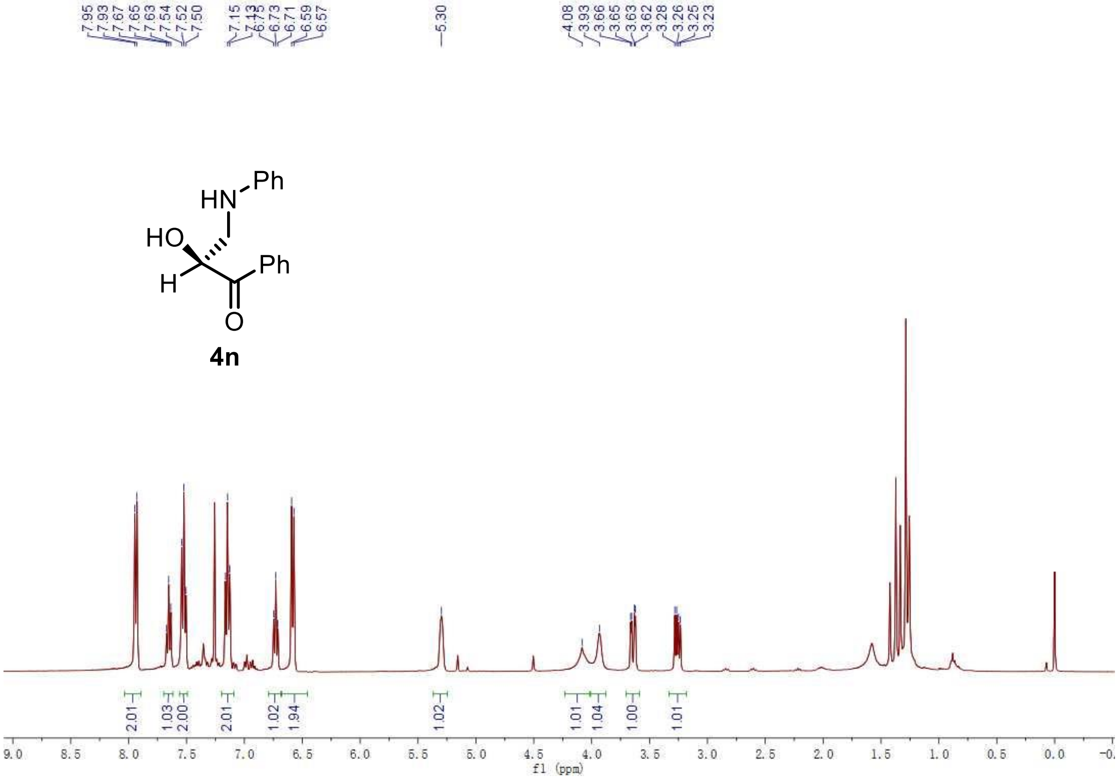

Supplementary Figure 85. ^{13}C NMR spectrum of (*S*)-**4n** (100 MHz, $CDCl_3$)

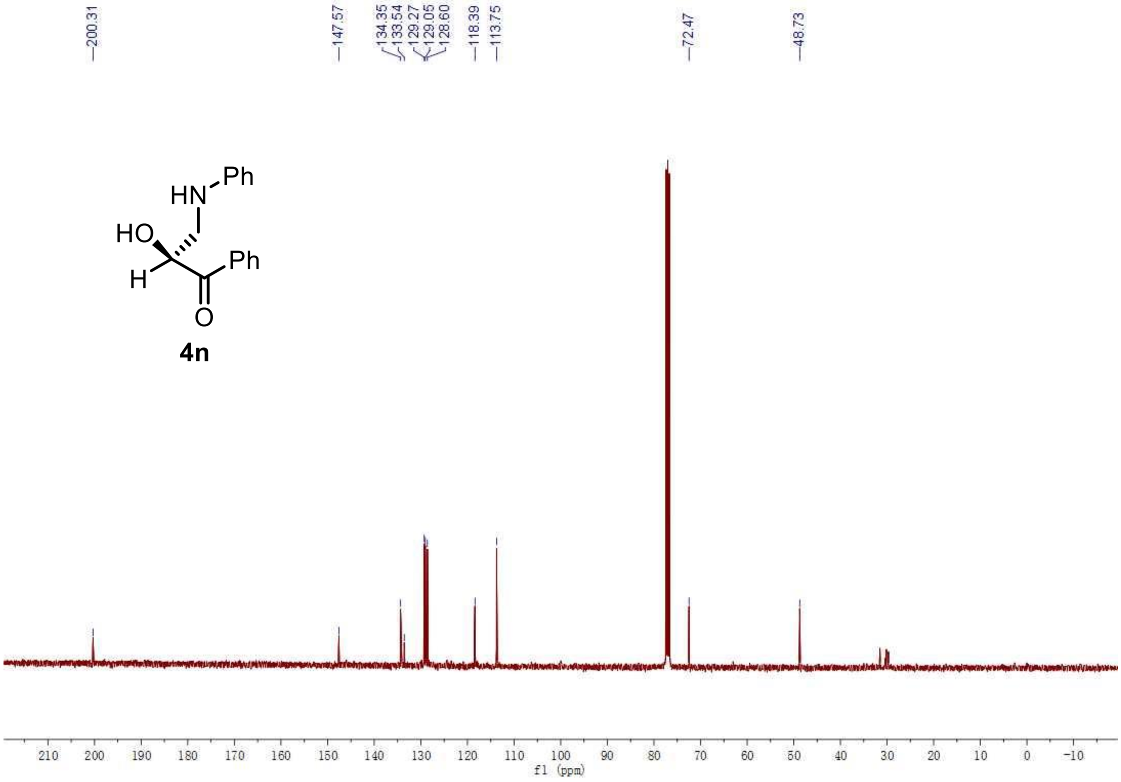

Supplementary Figure 86. ^{1}H NMR spectrum of **7** (400 MHz, $CDCl_3$)

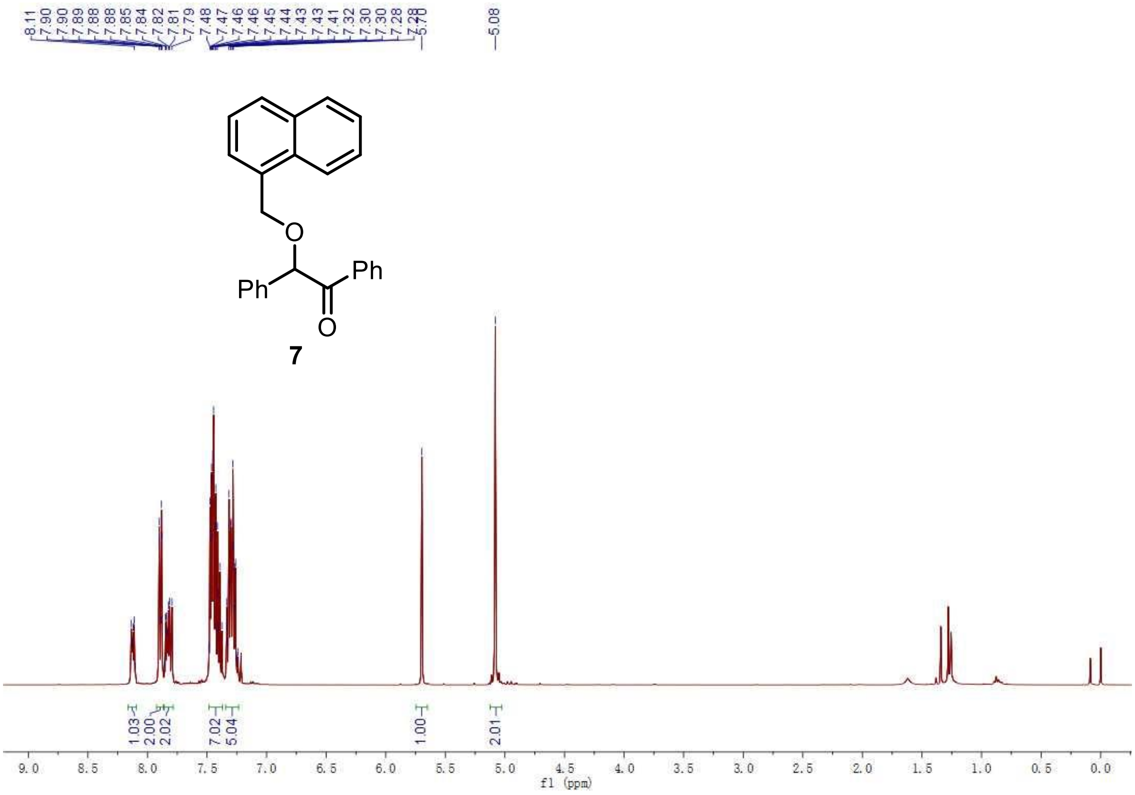

Supplementary Figure 87. ^{13}C NMR spectrum of **7** (100 MHz, $CDCl_3$)

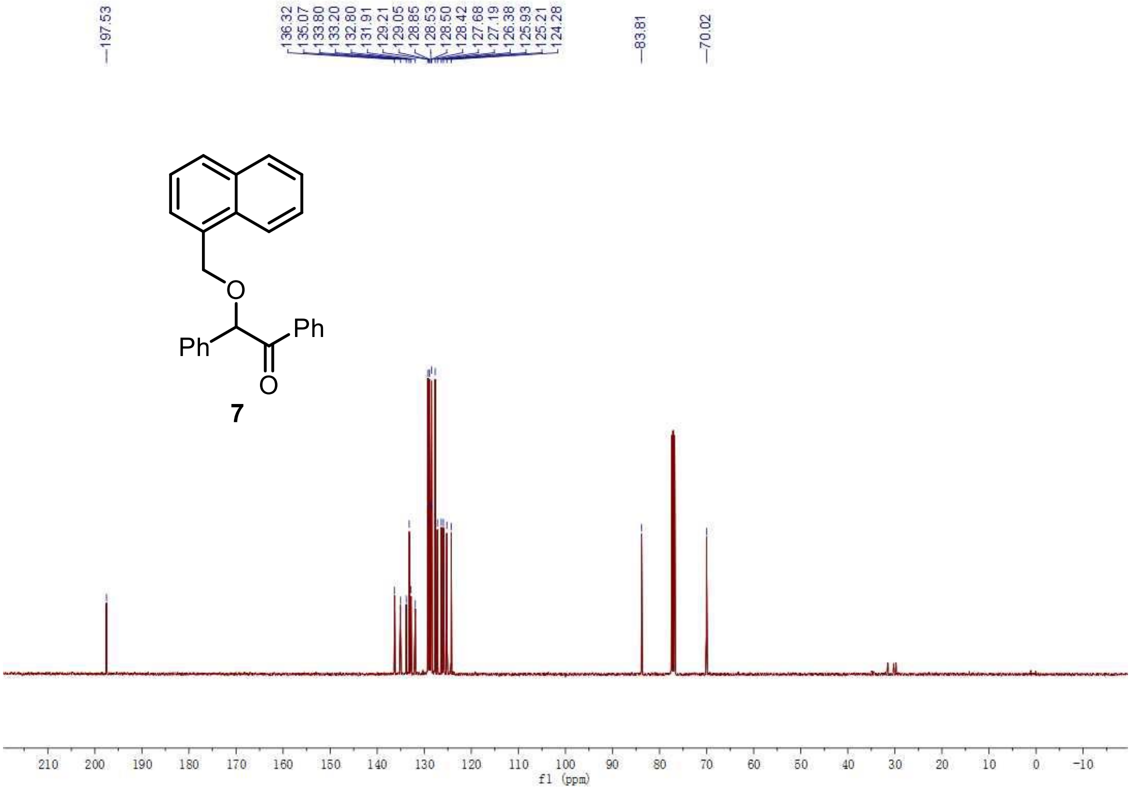

Supplementary Figure 88. ^{1}H NMR spectrum of **8** (400 MHz, $CDCl_3$)

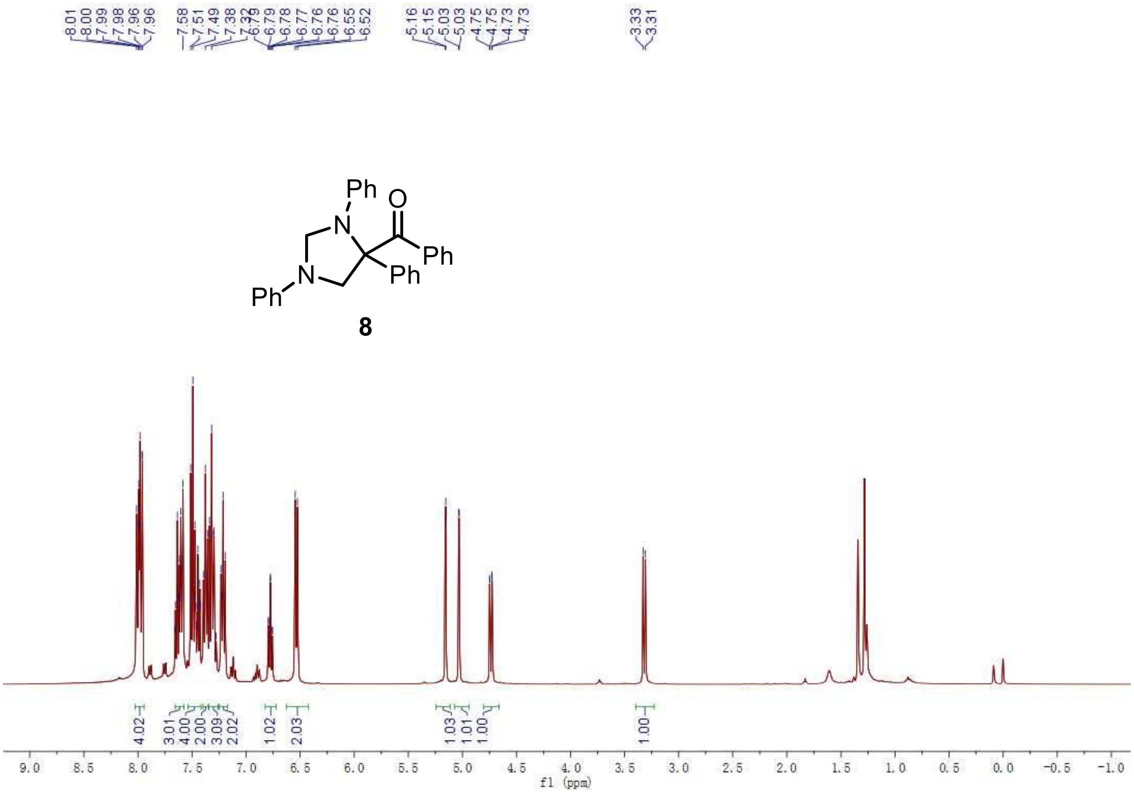

Supplementary Figure 89. ^{13}C NMR spectrum of **8** (100 MHz, $CDCl_3$)

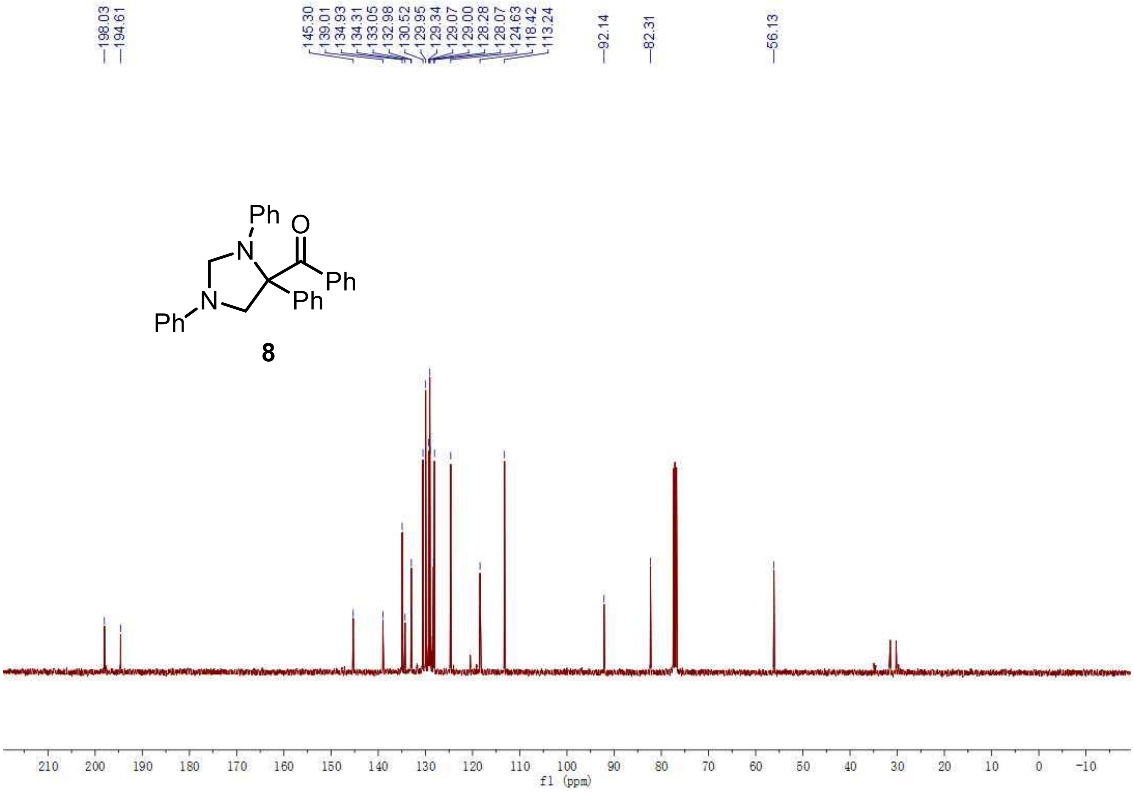

Supplementary Figure 90. ^{1}H NMR spectrum of **9** (400 MHz, $CDCl_3$)

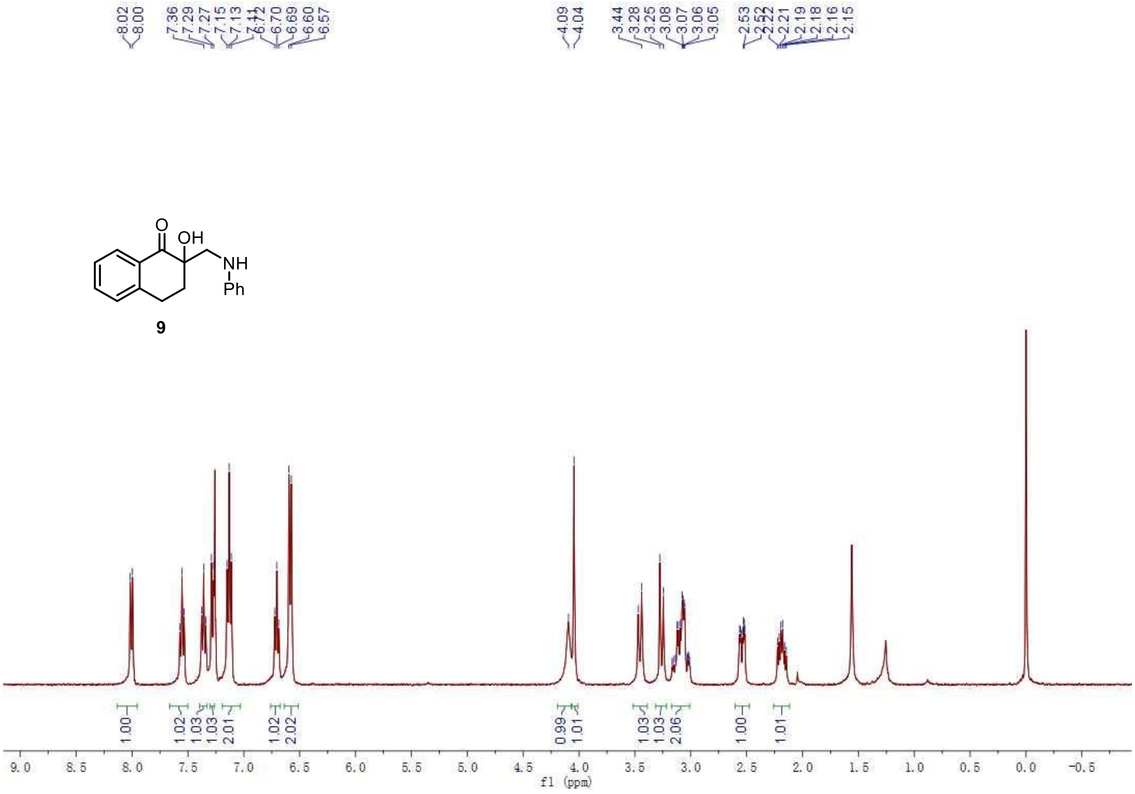

Supplementary Figure 91. ^{13}C NMR spectrum of **9** (100 MHz, $CDCl_3$)

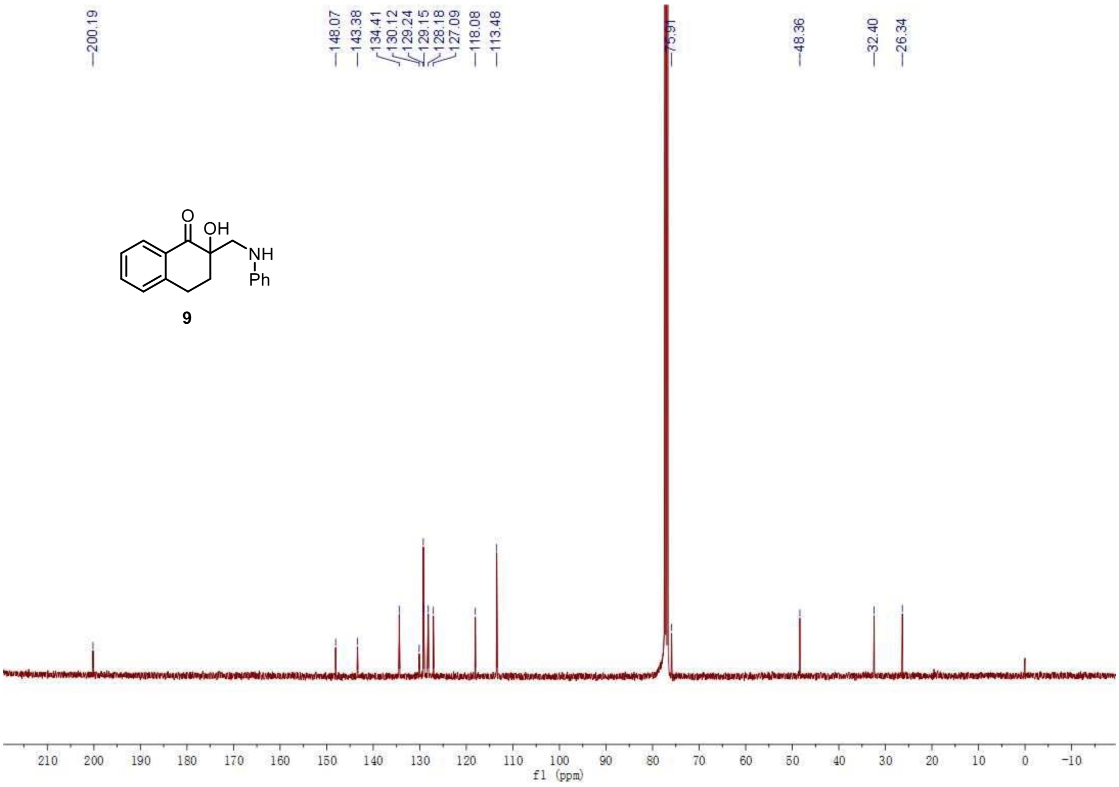

Supplementary Figure 92. HMBC Spectrum of Compound **9**

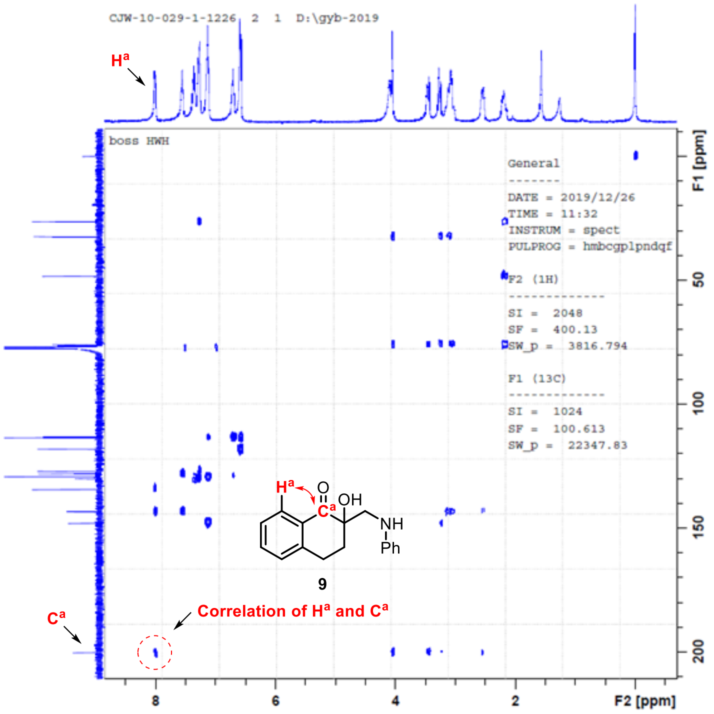

12. HPLC Spectra of Compounds

Condition: hexane/2-propanol = 80:1
Flow rate = 1.0 mL/min
λ = 254 nm
Chiral IC

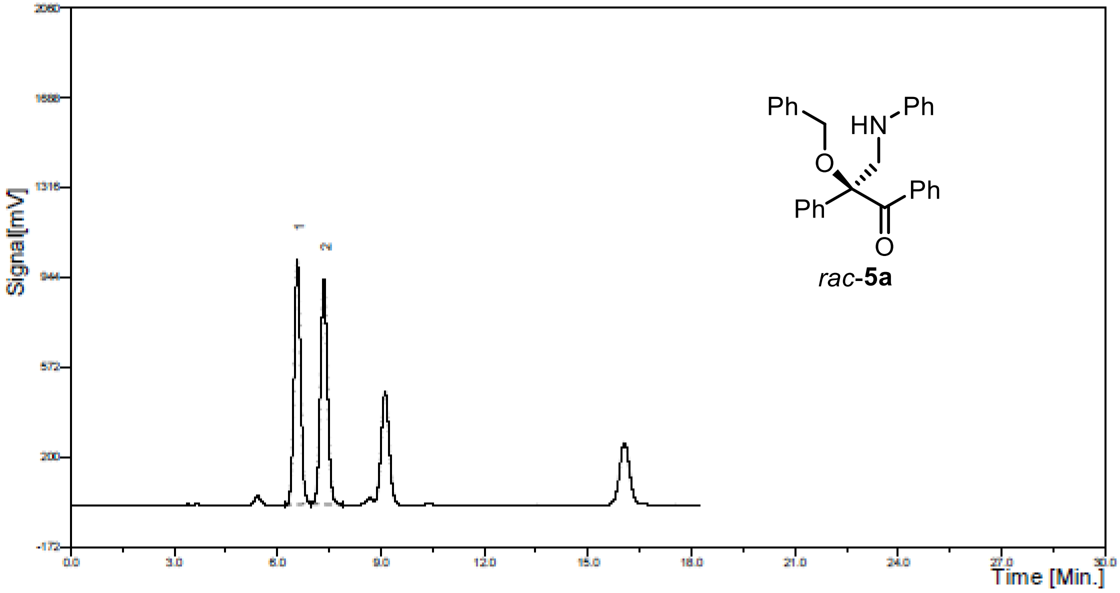

组分表

#	组分名	保留时间(min)	峰高(mV)	峰面积(mV.sec)	面积百分比(%)	浓 度	样品含量(%)
1	Unknown	6.57167	1013.80	13423.63	49.9934	0.0000	100.0000
2	Unknown	7.34583	931.90	13427.20	50.0066	0.0000	100.0000
合计			1945.70	26850.83	100		

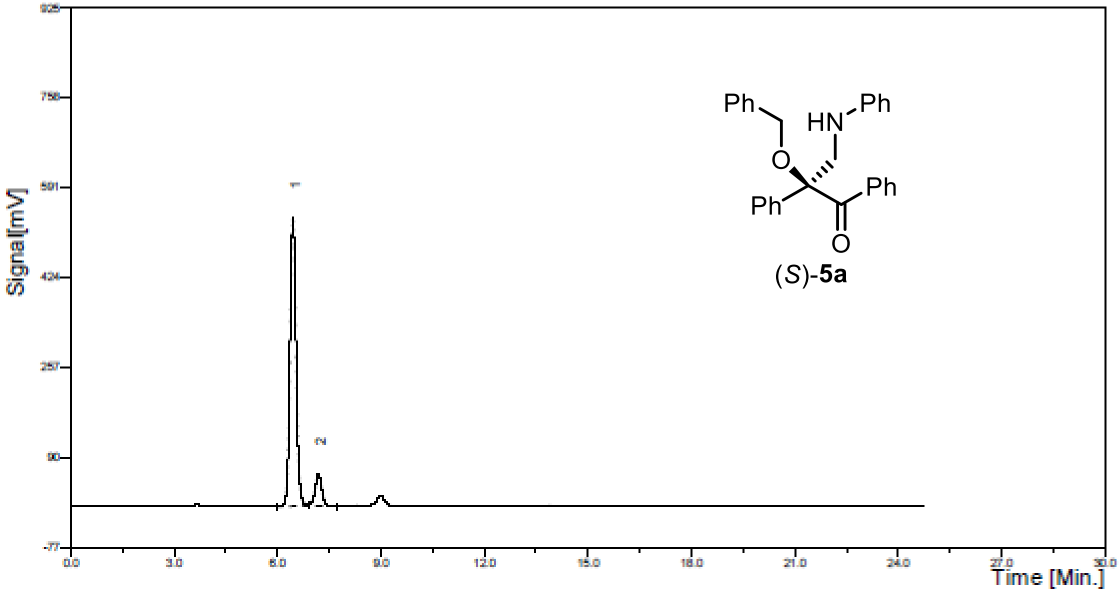

组分表

#	组分名	保留时间(min)	峰高(mV)	峰面积(mV.sec)	面积百分比(%)	浓 度	样品含量(%)
1	Unknown	6.44500	536.17	6550.27	89.0100	0.0000	100.0000
2	Unknown	7.18333	59.36	808.76	10.9900	0.0000	100.0000
合计			595.52	7359.03	100		

Condition: hexane/2-propanol = 20:1
Flow rate = 1.0 mL/min
λ = 254 nm
Chiral IA

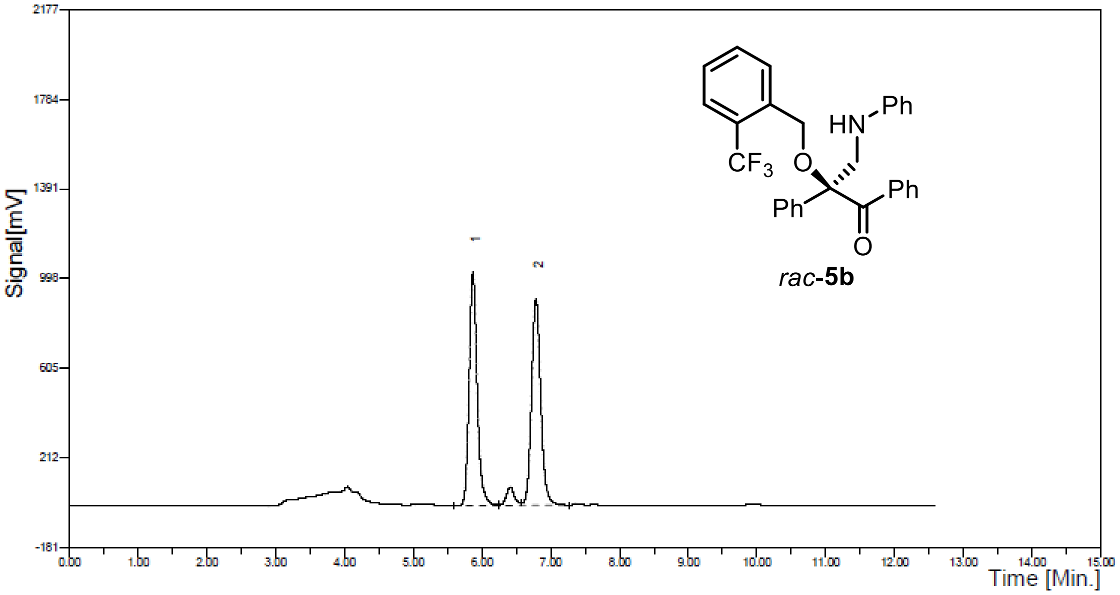

组分表

#	组分名	保留时间(min)	峰高(mV)	峰面积(mV. sec)	面积百分比(%)	浓 度	样品含量(%)
1	Unknown	5. 87167	1021. 04	8342. 46	49. 7101	0. 0000	0. 0000
2	Unknown	6. 78667	907. 15	8439. 78	50. 2899	0. 0000	0. 0000
合计			1928. 19	16782. 24	100		

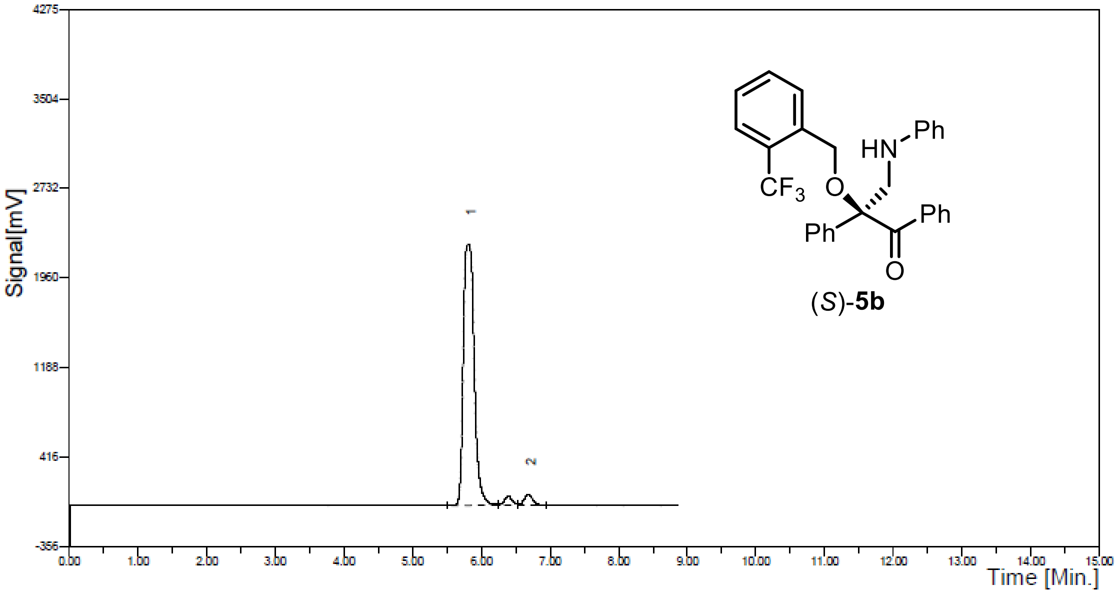

组分表

#	组分名	保留时间(min)	峰高(mV)	峰面积(mV. sec)	面积百分比(%)	浓 度	样品含量(%)
1	Unknown	5. 81167	2245. 72	24574. 24	96. 7834	0. 0000	0. 0000
2	Unknown	6. 68417	93. 81	816. 74	3. 2166	0. 0000	0. 0000
合计			2339. 52	25390. 98	100		

Condition: hexane/2-propanol = 40:1
Flow rate = 1.0 mL/min
λ = 254 nm
Chiral IA

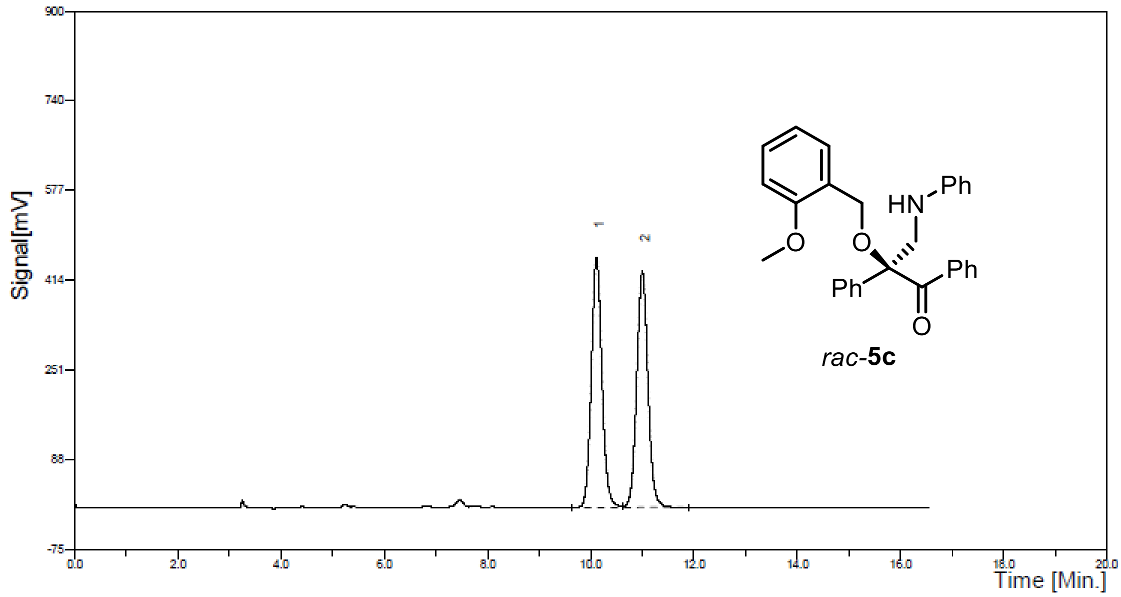

组分表

#	组分名	保留时间(min)	峰高(mV)	峰面积(mV.sec)	面积百分比(%)	浓 度	样品含量(%)
1	Unknown	10.11500	453.92	6509.83	50.1948	0.0000	100.0000
2	Unknown	11.00083	427.33	6459.30	49.8052	0.0000	100.0000
合计			881.24	12969.13	100		

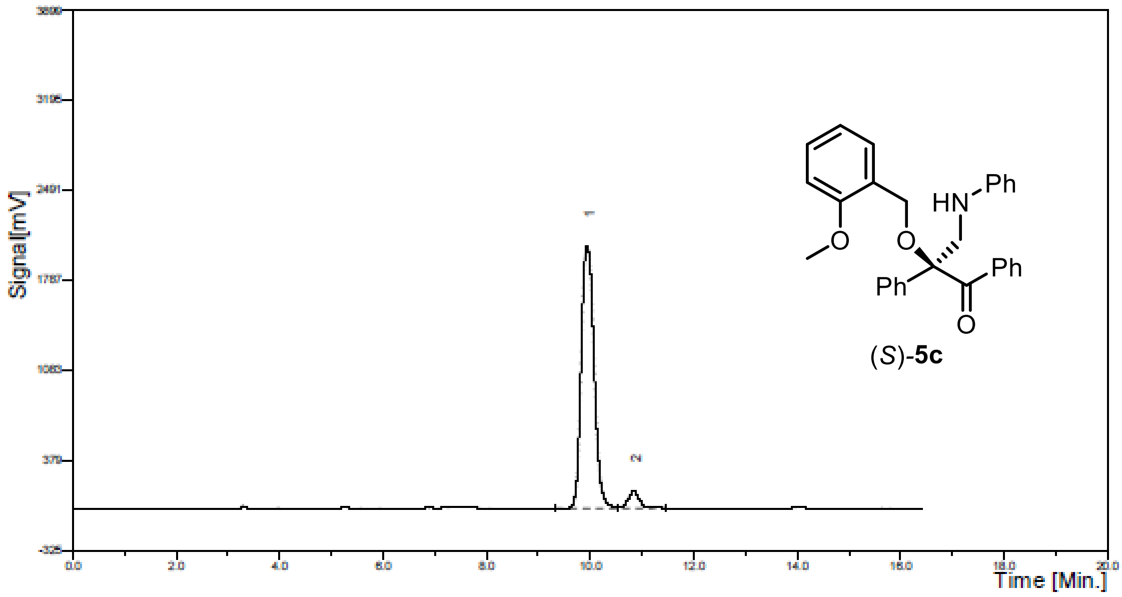

组分表

#	组分名	保留时间(min)	峰高(mV)	峰面积(mV.sec)	面积百分比(%)	浓 度	样品含量(%)
1	Unknown	9.93750	2049.10	35651.52	94.5576	0.0000	100.0000
2	Unknown	10.83667	131.10	2051.96	5.4424	0.0000	100.0000
合计			2180.20	37703.48	100		

Condition: hexane/2-propanol = 40:1
Flow rate = 1.0 mL/min
λ = 254 nm
Chiral IA

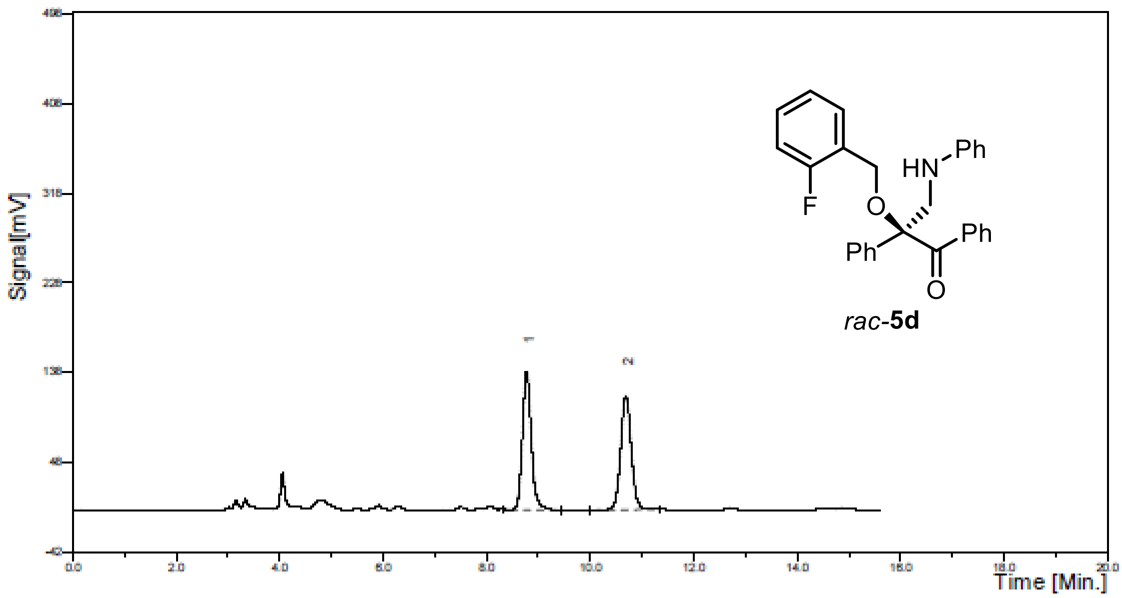

组分表

#	组分名	保留时间(min)	峰高(mV)	峰面积(mV. sec)	面积百分比(%)	浓 度	样品含量(%)
1	Unknown	8. 77750	138. 60	1666. 06	50. 4480	0. 0000	0. 0000
2	Unknown	10. 68750	114. 48	1636. 46	49. 5520	0. 0000	0. 0000
合计			253. 08	3302. 52	100		

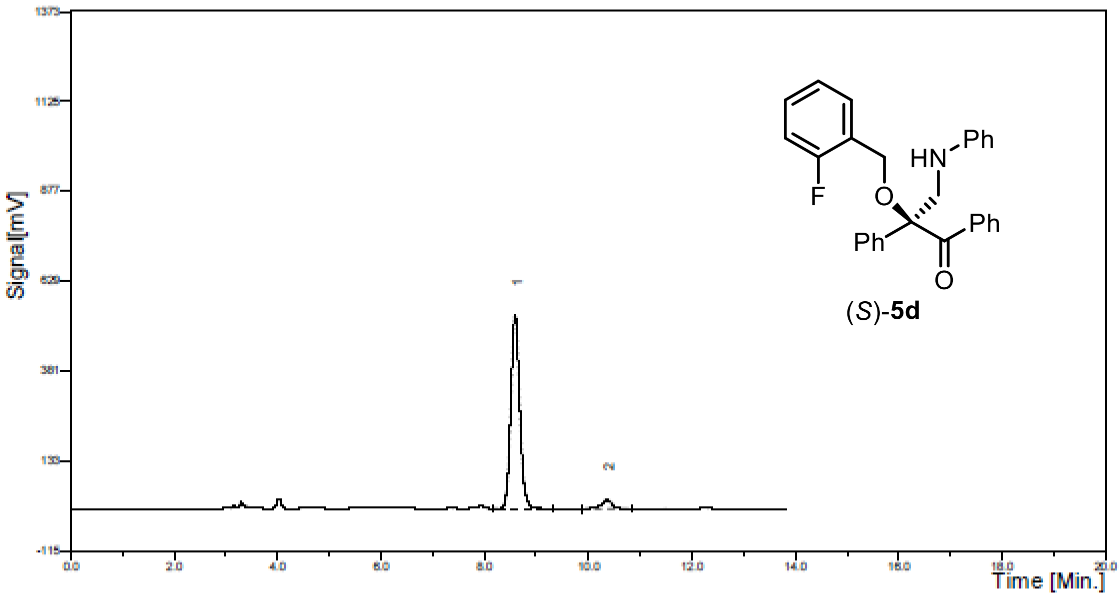

组分表

#	组分名	保留时间(min)	峰高(mV)	峰面积(mV. sec)	面积百分比(%)	浓 度	样品含量(%)
1	Unknown	8. 59583	532. 65	6453. 82	94. 1653	0. 0000	100. 0000
2	Unknown	10. 35583	25. 58	399. 90	5. 8347	0. 0000	100. 0000
合计			558. 23	6853. 72	100		

Condition: hexane/2-propanol = 40:1
Flow rate = 1.0 mL/min
λ = 254 nm
Chiral IA

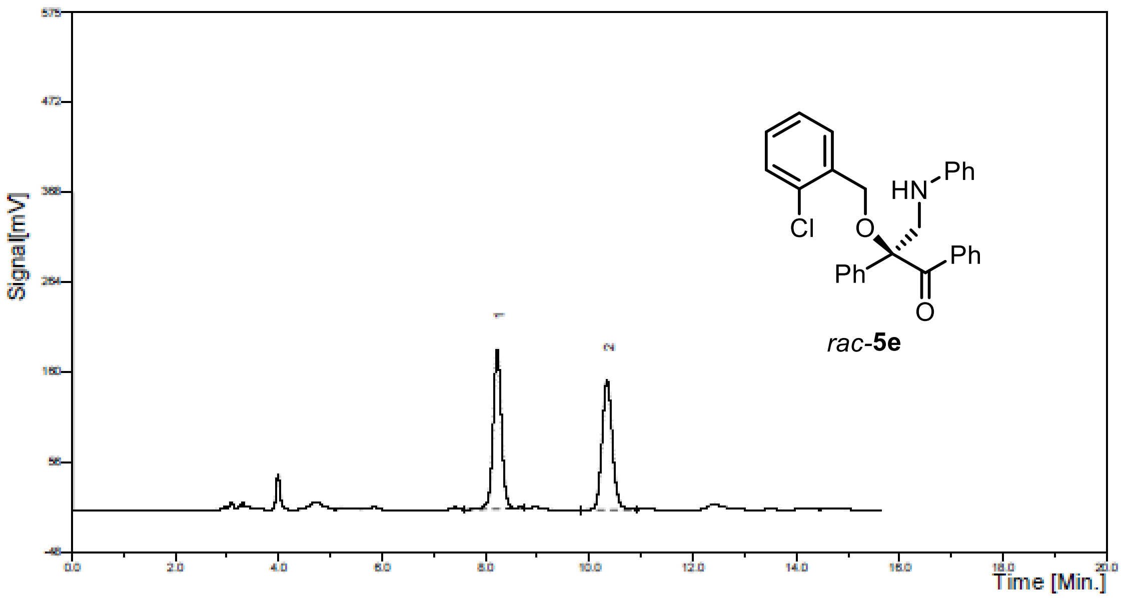

组分表

#	组分名	保留时间(min)	峰高(mV)	峰面积(mV.sec)	面积百分比(%)	浓 度	样品含量(%)
1	Unknown	8.22667	183.99	2099.70	50.1167	0.0000	100.0000
2	Unknown	10.34250	149.71	2089.92	49.8833	0.0000	100.0000
合计			333.70	4189.62	100		

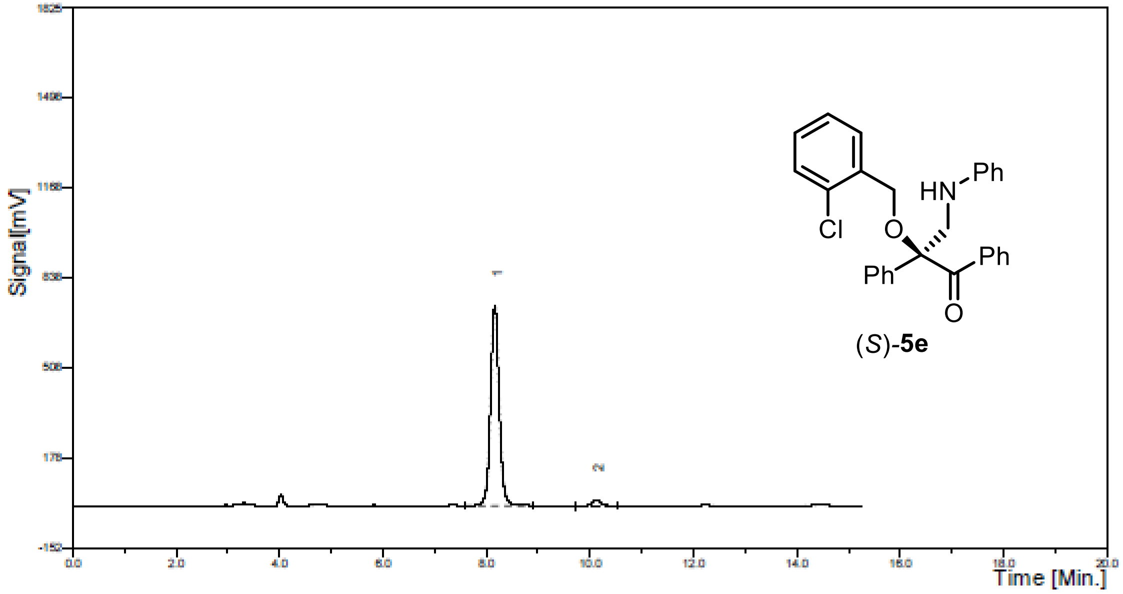

组分表

#	组分名	保留时间(min)	峰高(mV)	峰面积(mV.sec)	面积百分比(%)	浓 度	样品含量(%)
1	Unknown	8.16417	732.84	8610.61	96.6280	0.0000	0.0000
2	Unknown	10.12000	22.21	300.49	3.3720	0.0000	0.0000
合计			755.05	8911.10	100		

Condition: hexane/2-propanol = 40:1
Flow rate = 1.0 mL/min
λ = 254 nm
Chiral IA

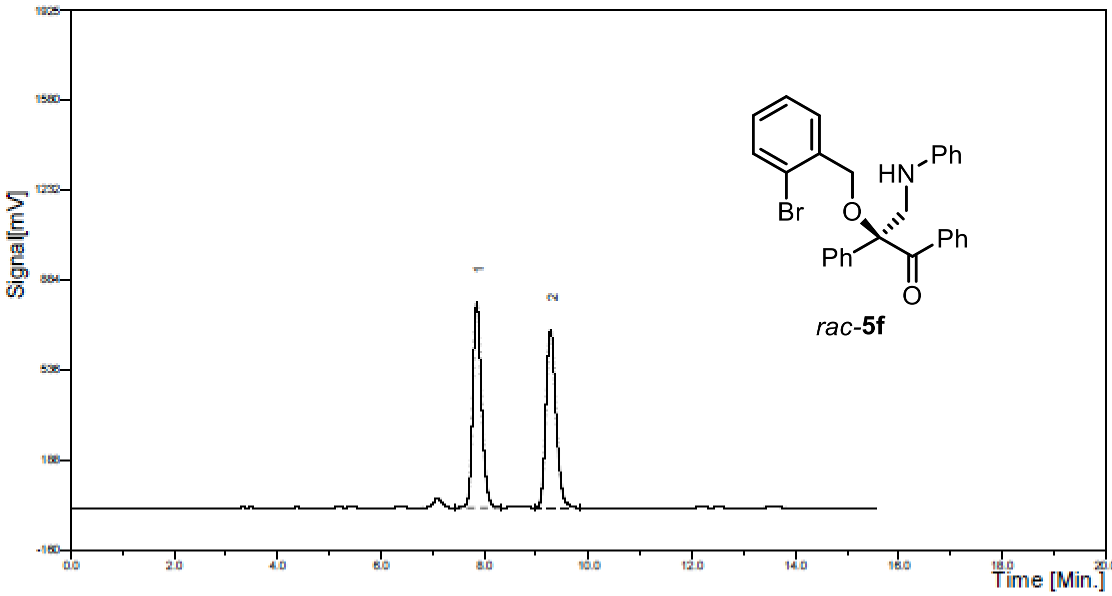

组分表

#	组分名	保留时间(min)	峰高(mV)	峰面积(mV. sec)	面积百分比(%)	浓 度	样品含量(%)
1	Unknown	7.85500	791.99	9447.14	50.2931	0.0000	100.0000
2	Unknown	9.28250	686.87	9337.02	49.7069	0.0000	100.0000
合计			1478.87	18784.16	100		

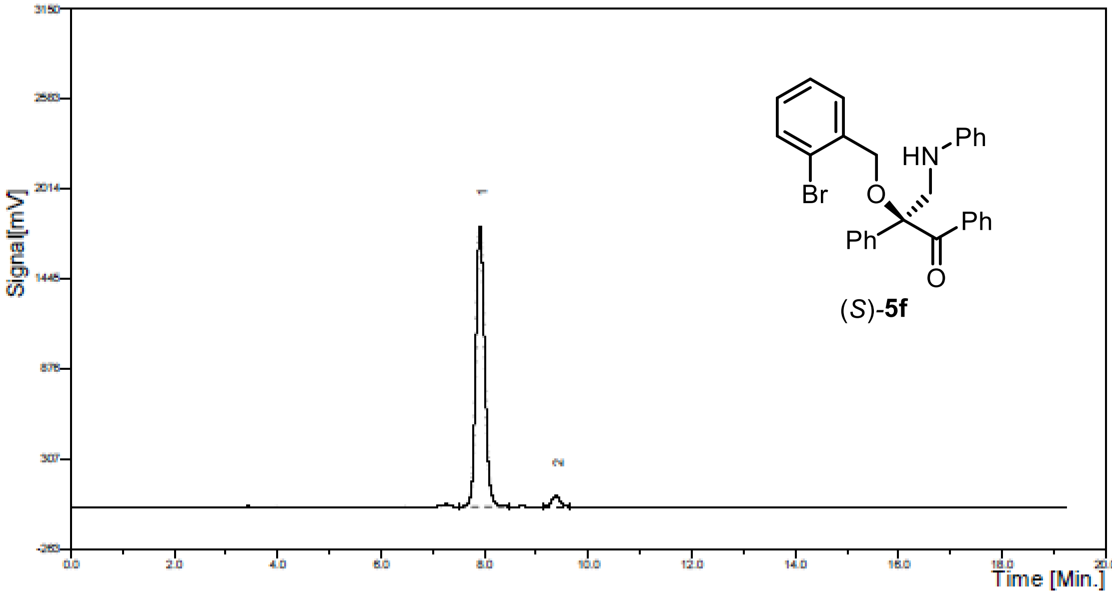

组分表

#	组分名	保留时间(min)	峰高(mV)	峰面积(mV. sec)	面积百分比(%)	浓 度	样品含量(%)
1	Unknown	7.91167	1773.85	21052.03	96.2293	0.0000	100.0000
2	Unknown	9.37833	70.58	824.92	3.7707	0.0000	100.0000
合计			1844.43	21876.96	100		

Condition: hexane/2-propanol = 40:1
Flow rate = 1.0 mL/min
λ = 254 nm
Chiral IA

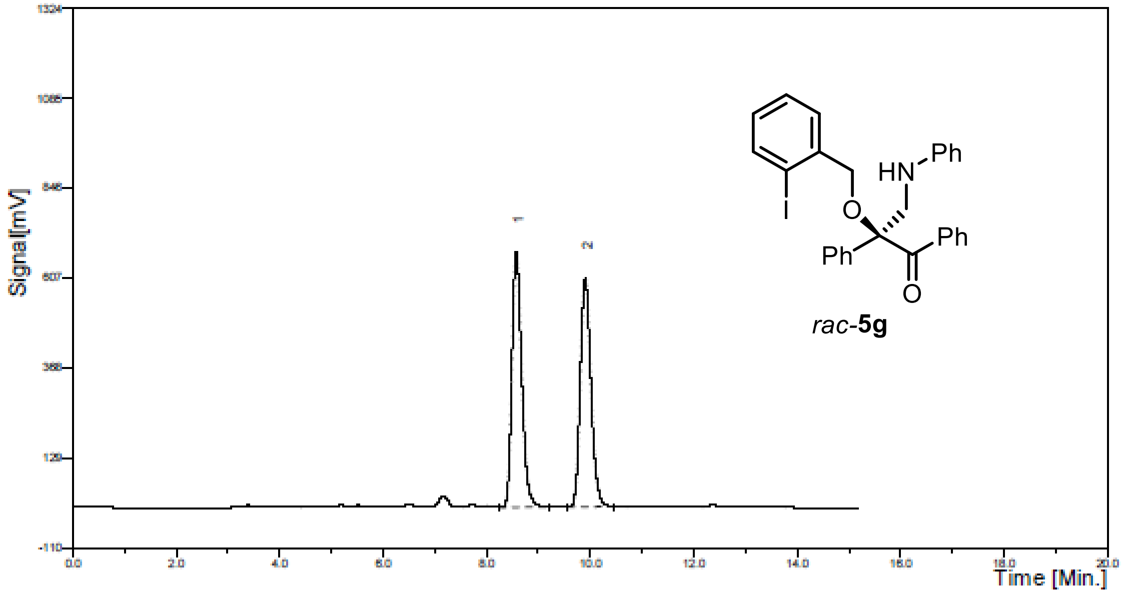

组分表

#	组分名	保留时间(min)	峰高(mV)	峰面积(mV. sec)	面积百分比(%)	浓 度	样品含量(%)
1	Unknown	8. 57583	676. 76	8960. 36	50. 6762	0. 0000	0. 0000
2	Unknown	9. 90083	609. 69	8721. 23	49. 3238	0. 0000	0. 0000
合计			1286. 45	17681. 59	100		

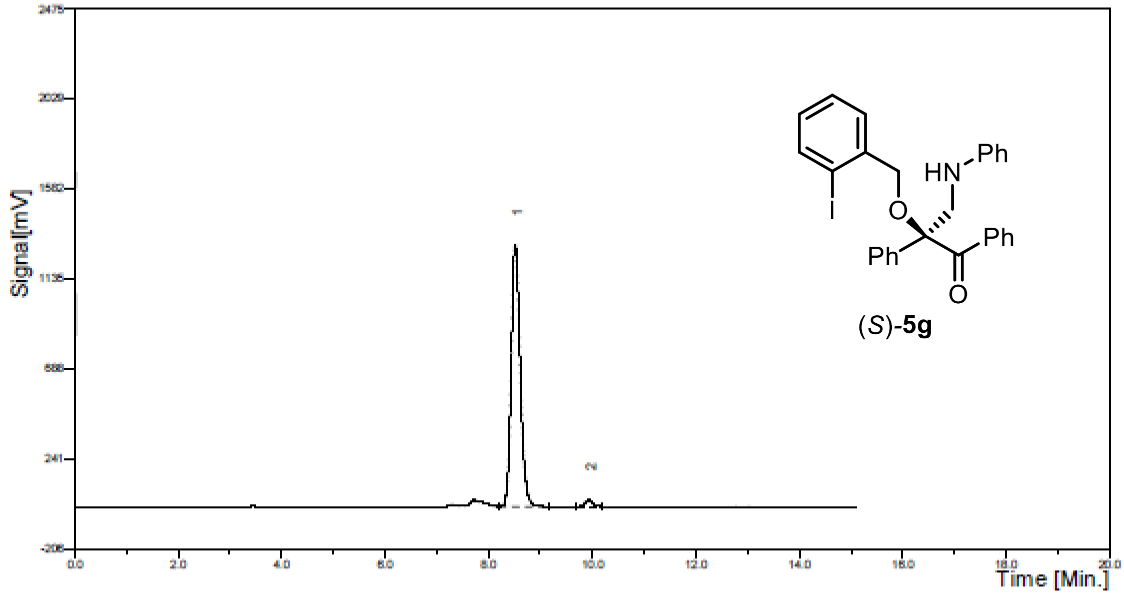

组分表

#	组分名	保留时间(min)	峰高(mV)	峰面积(mV. sec)	面积百分比(%)	浓 度	样品含量(%)
1	Unknown	8. 52250	1300. 30	15820. 64	97. 5183	0. 0000	100. 0000
2	Unknown	9. 92583	33. 02	402. 61	2. 4817	0. 0000	100. 0000
合计			1333. 32	16223. 25	100		

Condition: hexane/2-propanol = 80:1
Flow rate = 1.0 mL/min
λ = 254 nm
Chiral IA

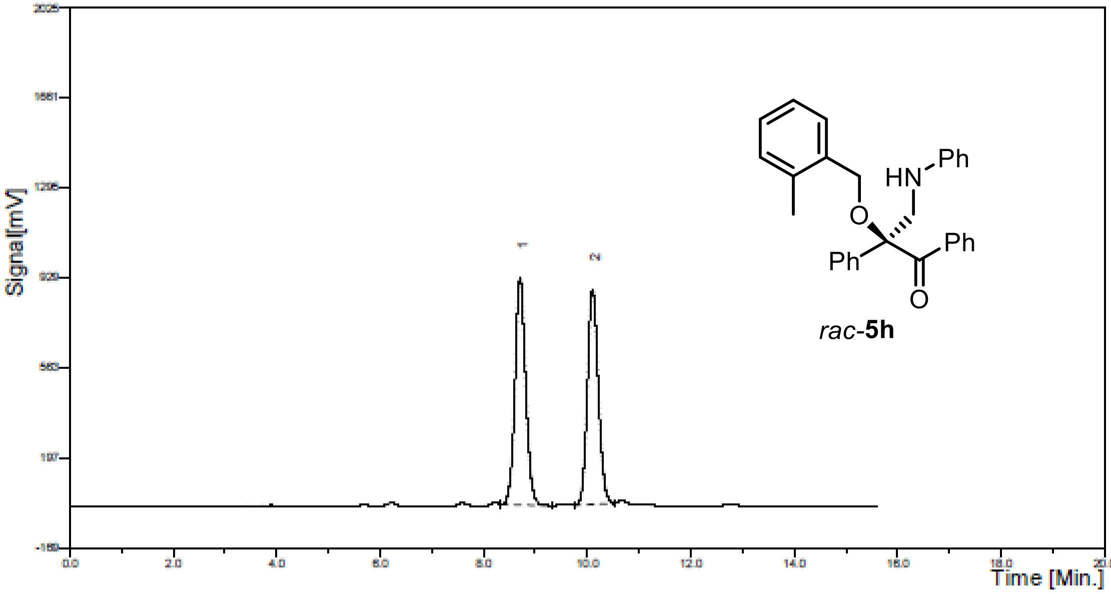

组分表

#	组分名	保留时间(min)	峰高(mV)	峰面积(mV. sec)	面积百分比(%)	浓 度	样品含量(%)
1	Unknown	8. 70750	921. 21	13322. 88	51. 1082	0. 0000	0. 0000
2	Unknown	10. 10167	870. 29	12745. 10	48. 8918	0. 0000	0. 0000
合计			1791. 50	26067. 98	100		

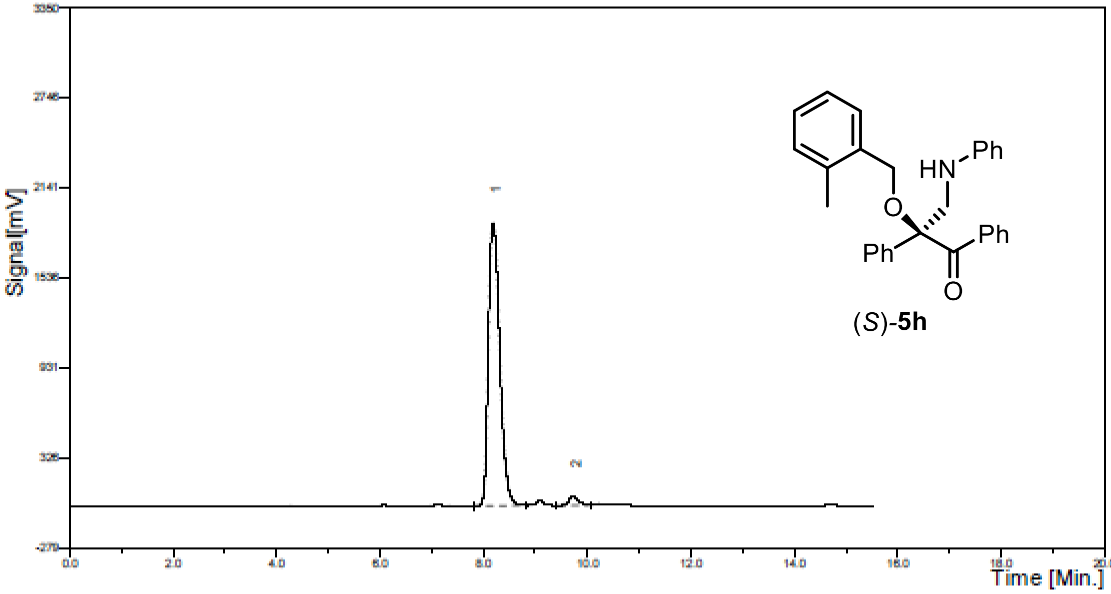

组分表

#	组分名	保留时间(min)	峰高(mV)	峰面积(mV. sec)	面积百分比(%)	浓 度	样品含量(%)
1	Unknown	8. 19083	1901. 37	29243. 70	97. 3661	0. 0000	0. 0000
2	Unknown	9. 72833	59. 39	791. 08	2. 6339	0. 0000	100. 0000
合计			1960. 76	30034. 79	100		

Condition: hexane/2-propanol = 40:1
Flow rate = 1.0 mL/min
λ = 254 nm
Chiral IA

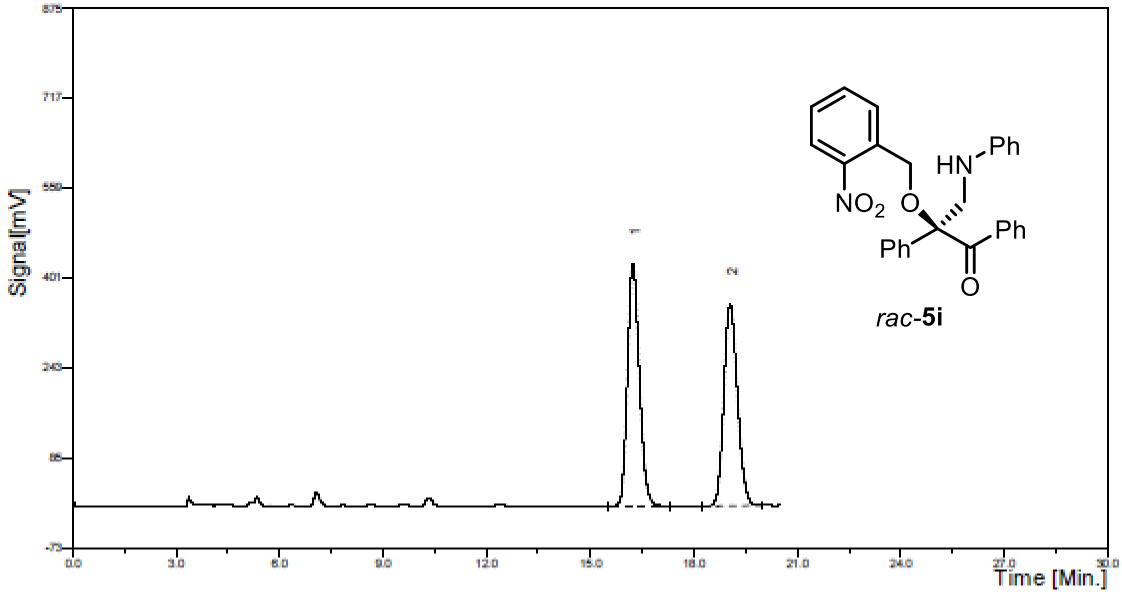

组分表

#	组分名	保留时间(min)	峰高(mV)	峰面积(mV. sec)	面积百分比(%)	浓 度	样品含量(%)
1	Unknown	16. 22833	424. 83	10286. 55	51. 2532	0. 0000	0. 0000
2	Unknown	19. 05167	355. 21	9783. 52	48. 7468	0. 0000	0. 0000
合计			780. 04	20070. 07	100		

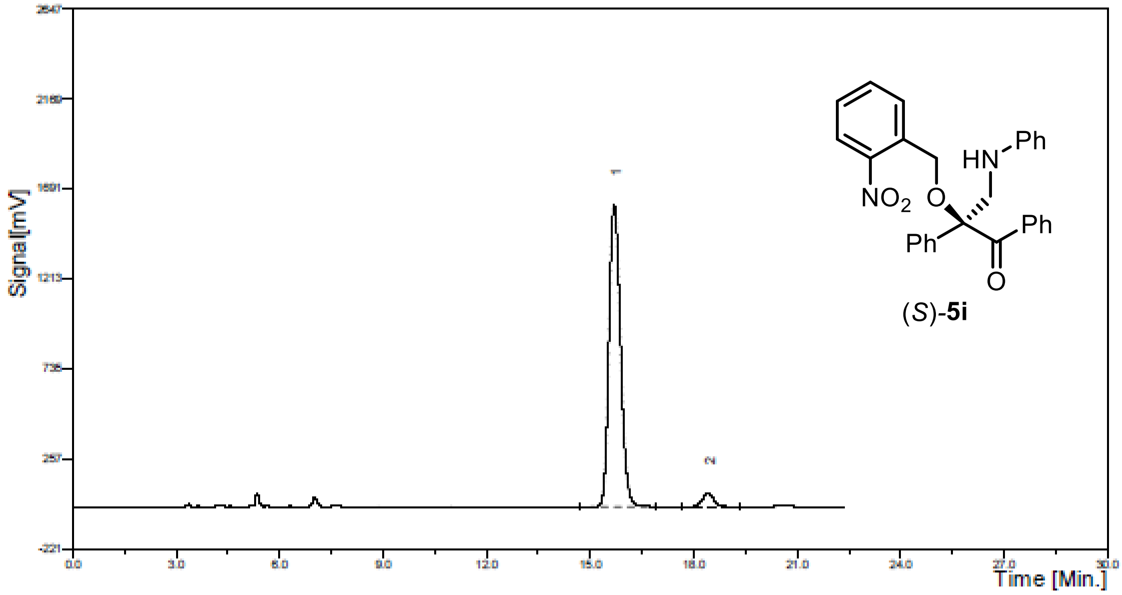

组分表

#	组分名	保留时间(min)	峰高(mV)	峰面积(mV. sec)	面积百分比(%)	浓 度	样品含量(%)
1	Unknown	15. 69167	1608. 51	37452. 92	95. 7125	0. 0000	100. 0000
2	Unknown	18. 40917	68. 70	1677. 72	4. 2875	0. 0000	100. 0000
合计			1677. 21	39130. 64	100		

Condition: hexane/2-propanol = 40:1
Flow rate = 1.0 mL/min
λ = 254 nm
Chiral IA

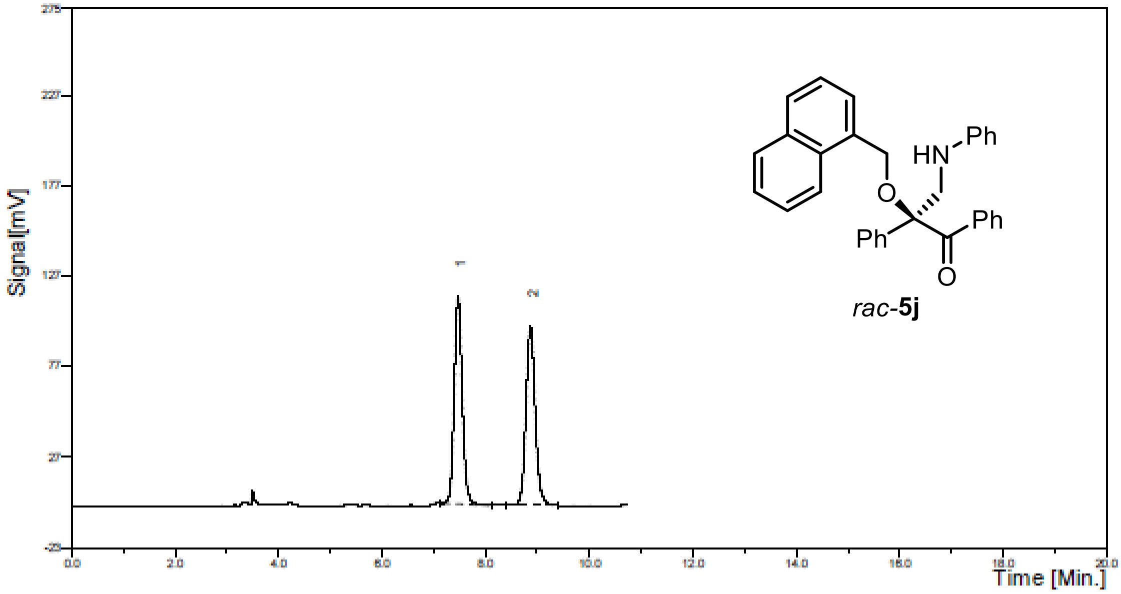

组分表

#	组分名	保留时间(min)	峰高(mV)	峰面积(mV. sec)	面积百分比(%)	浓 度	样品含量(%)
1	Unknown	7. 47417	114. 47	1231. 92	50. 0716	0. 0000	100. 0000
2	Unknown	8. 87667	98. 63	1228. 40	49. 9284	0. 0000	100. 0000
合计			213. 10	2460. 33	100		

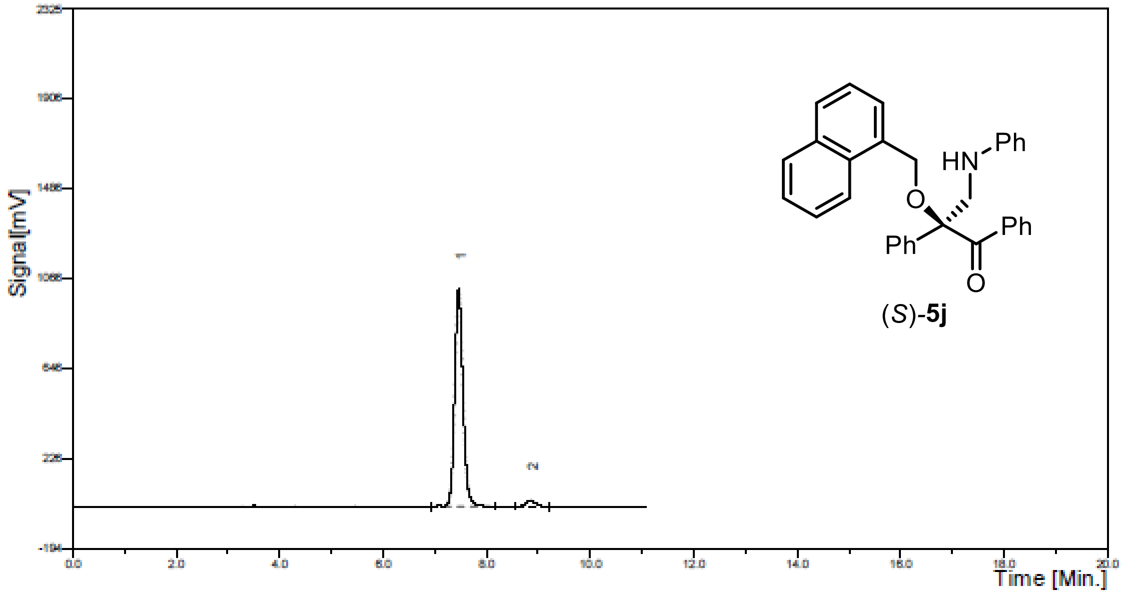

组分表

#	组分名	保留时间(min)	峰高(mV)	峰面积(mV. sec)	面积百分比(%)	浓 度	样品含量(%)
1	Unknown	7. 46167	1016. 44	11447. 51	96. 7975	0. 0000	100. 0000
2	Unknown	8. 85833	30. 01	378. 73	3. 2025	0. 0000	0. 0000
合计			1046. 46	11826. 24	100		

Condition: hexane/2-propanol = 40:1
Flow rate = 1.0 mL/min
λ = 254 nm
Chiral IA

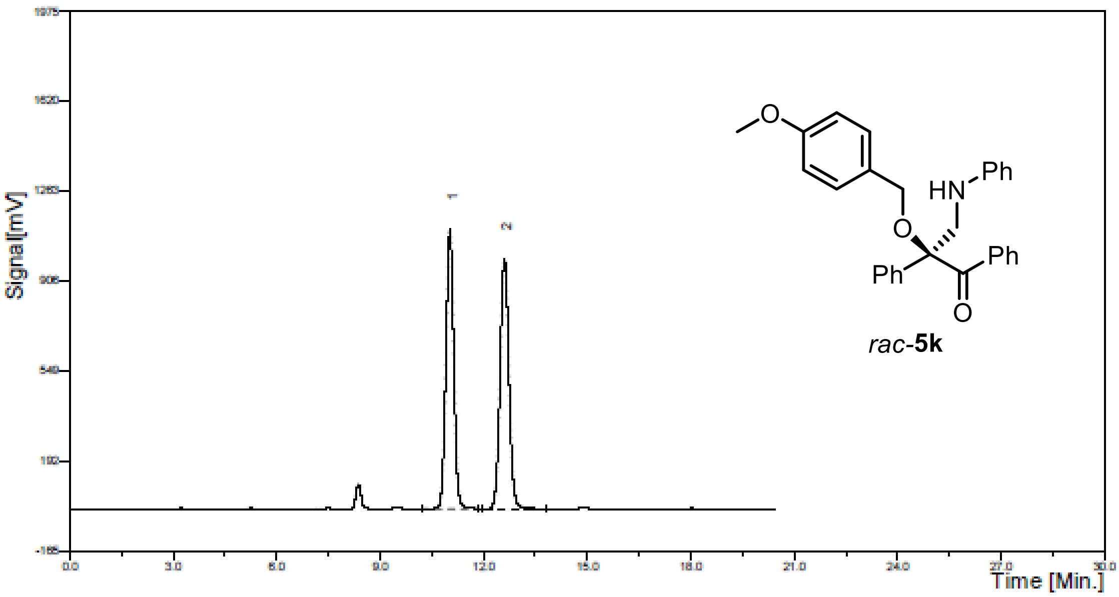

组分表

#	组分名	保留时间(min)	峰高(mV)	峰面积(mV. sec)	面积百分比(%)	浓 度	样品含量(%)
1	Unknown	11. 02583	1106. 08	17559. 48	49. 6812	0. 0000	100. 0000
2	Unknown	12. 61000	988. 66	17784. 87	50. 3188	0. 0000	100. 0000
合计			2094. 74	35344. 35	100		

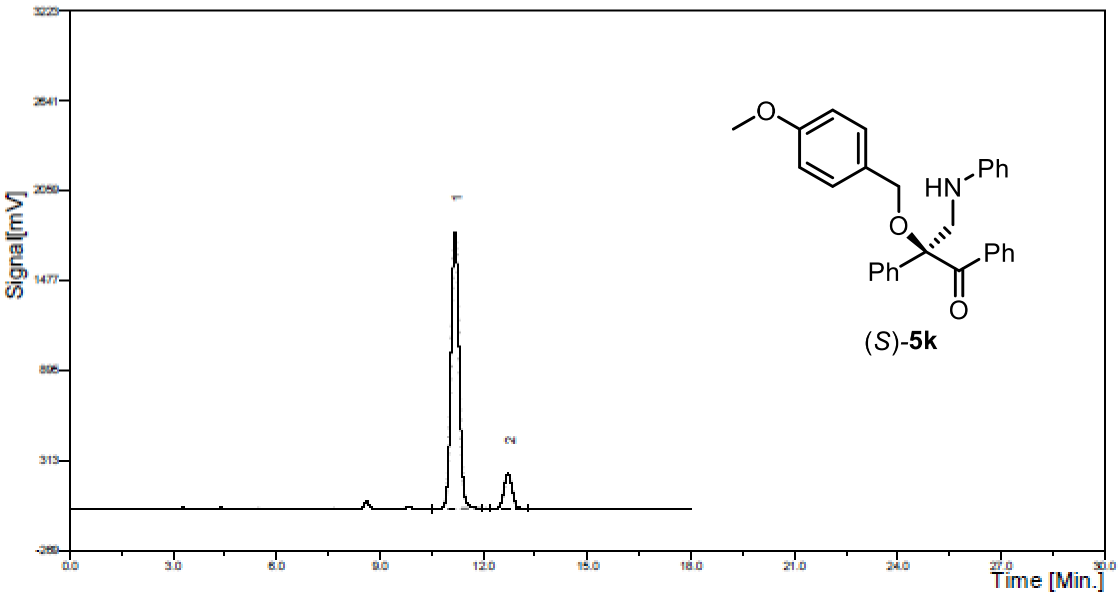

组分表

#	组分名	保留时间(min)	峰高(mV)	峰面积(mV. sec)	面积百分比(%)	浓 度	样品含量(%)
1	Unknown	11. 18333	1790. 92	29610. 92	88. 6308	0. 0000	100. 0000
2	Unknown	12. 72000	226. 94	3798. 38	11. 3692	0. 0000	100. 0000
合计			2017. 86	33409. 30	100		

Condition: hexane/2-propanol = 40:1
Flow rate = 1.0 mL/min
λ = 254 nm
Chiral IA

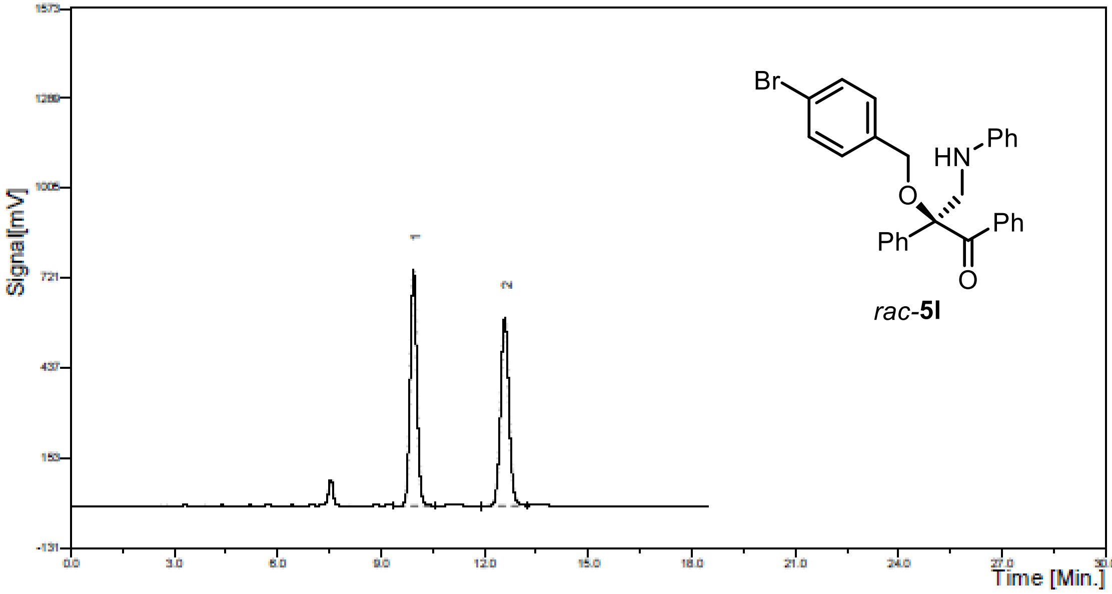

组分表

#	组分名	保留时间(min)	峰高(mV)	峰面积(mV.sec)	面积百分比(%)	浓 度	样品含量(%)
1	Unknown	9.94250	747.17	10143.11	50.1488	0.0000	100.0000
2	Unknown	12.58833	592.78	10082.91	49.8512	0.0000	100.0000
合计			1339.95	20226.02	100		

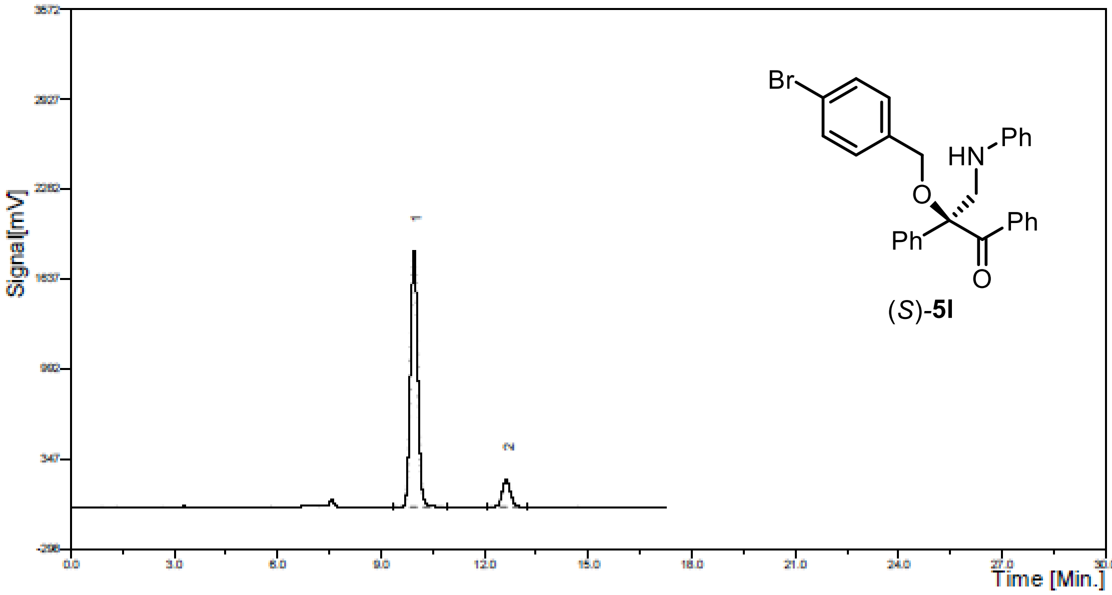

组分表

#	组分名	保留时间(min)	峰高(mV)	峰面积(mV.sec)	面积百分比(%)	浓 度	样品含量(%)
1	Unknown	9.95583	1836.44	27551.86	89.3812	0.0000	100.0000
2	Unknown	12.63833	194.09	3273.26	10.6188	0.0000	100.0000
合计			2030.53	30825.11	100		

Condition: hexane/2-propanol = 40:1
Flow rate = 1.0 mL/min
λ = 254 nm
Chiral IA

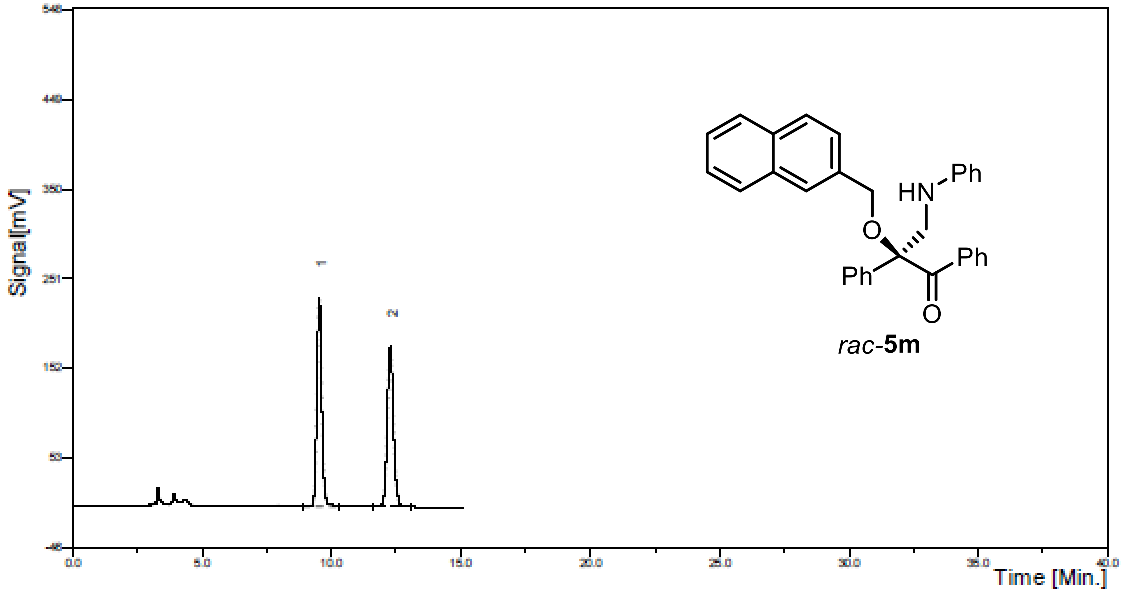

组分表

#	组分名	保留时间(min)	峰高(mV)	峰面积(mV. sec)	面积百分比(%)	浓 度	样品含量(%)
1	Unknown	9. 53417	231. 42	3070. 95	50. 8733	0. 0000	100. 0000
2	Unknown	12. 28250	176. 88	2965. 51	49. 1267	0. 0000	100. 0000
合计			408. 29	6036. 46	100		

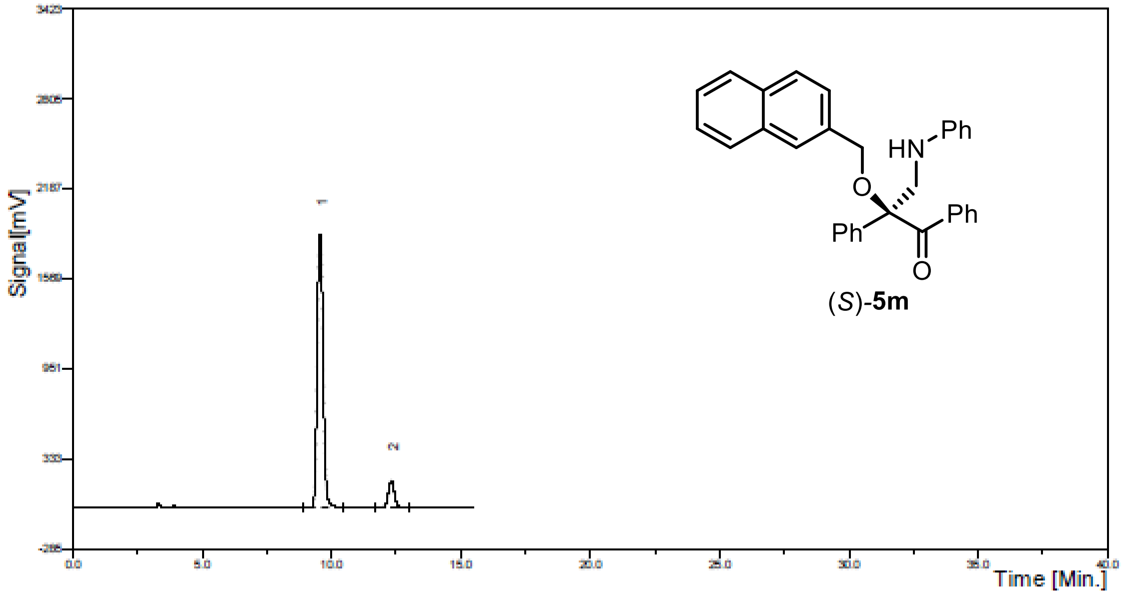

组分表

#	组分名	保留时间(min)	峰高(mV)	峰面积(mV. sec)	面积百分比(%)	浓 度	样品含量(%)
1	Unknown	9. 56000	1867. 67	29845. 92	90. 8319	0. 0000	100. 0000
2	Unknown	12. 31833	182. 52	3012. 51	9. 1681	0. 0000	100. 0000
合计			2050. 19	32858. 42	100		

Condition: hexane/2-propanol = 40:1
Flow rate = 1.0 mL/min
λ = 254 nm
Chiral IA

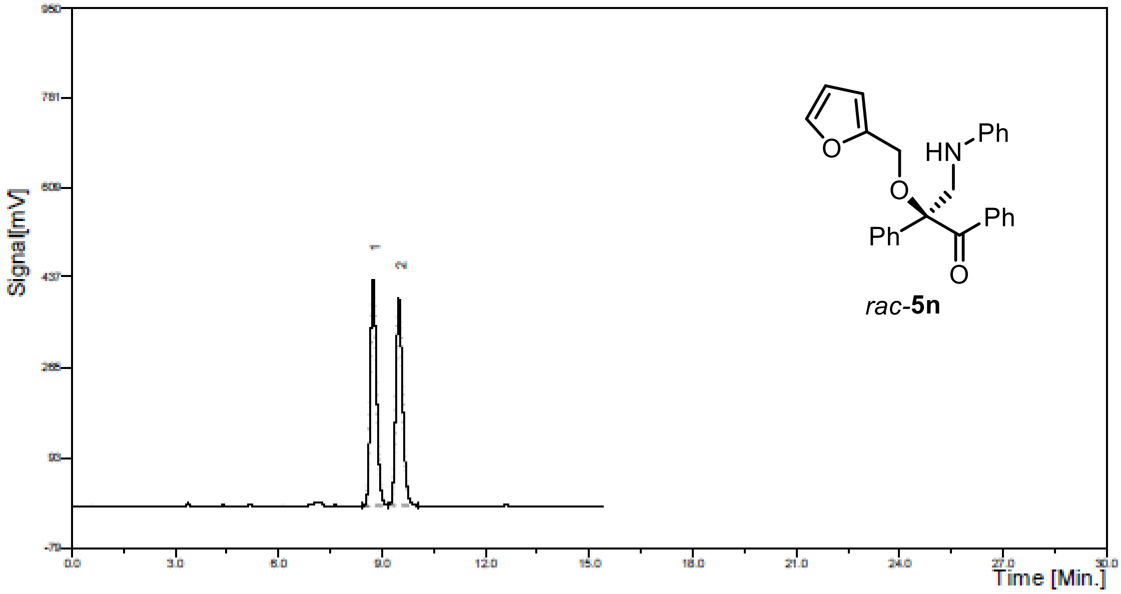

组分表

#	组分名	保留时间(min)	峰高(mV)	峰面积(mV.sec)	面积百分比(%)	浓 度	样品含量(%)
1	Unknown	8.74000	431.29	5443.10	50.3515	0.0000	100.0000
2	Unknown	9.48917	393.37	5367.10	49.6485	0.0000	100.0000
合计			824.66	10810.20	100		

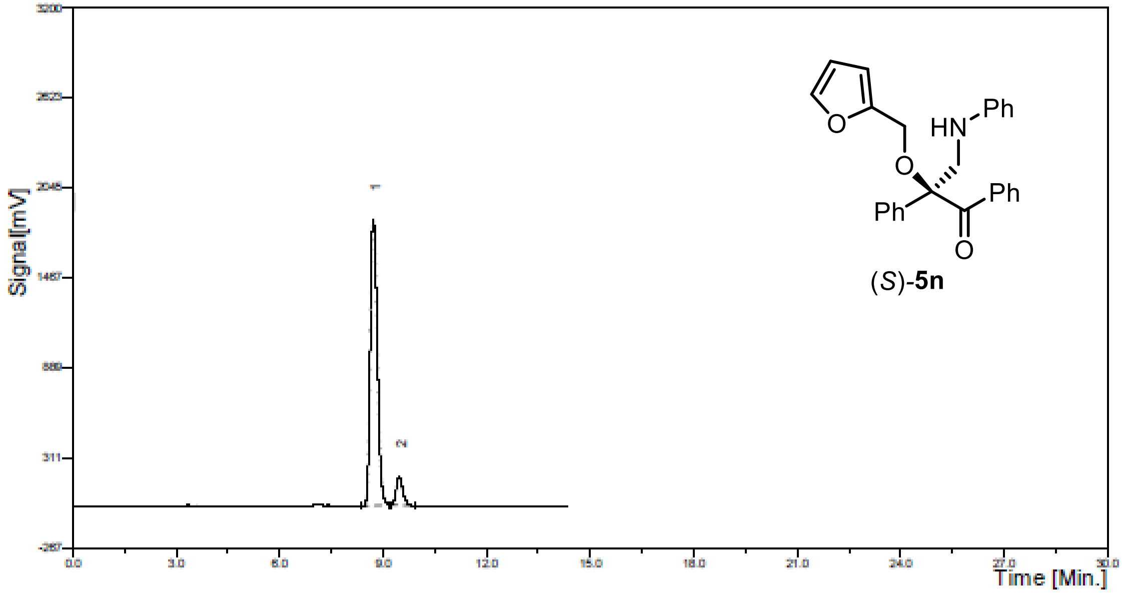

组分表

#	组分名	保留时间(min)	峰高(mV)	峰面积(mV.sec)	面积百分比(%)	浓 度	样品含量(%)
1	Unknown	8.71750	1831.91	26278.65	91.4960	0.0000	100.0000
2	Unknown	9.46917	179.47	2442.43	8.5040	0.0000	100.0000
合计			2011.38	28721.08	100		

Condition: hexane/2-propanol = 80:1
Flow rate = 1.0 mL/min
λ = 254 nm
Chiral IA

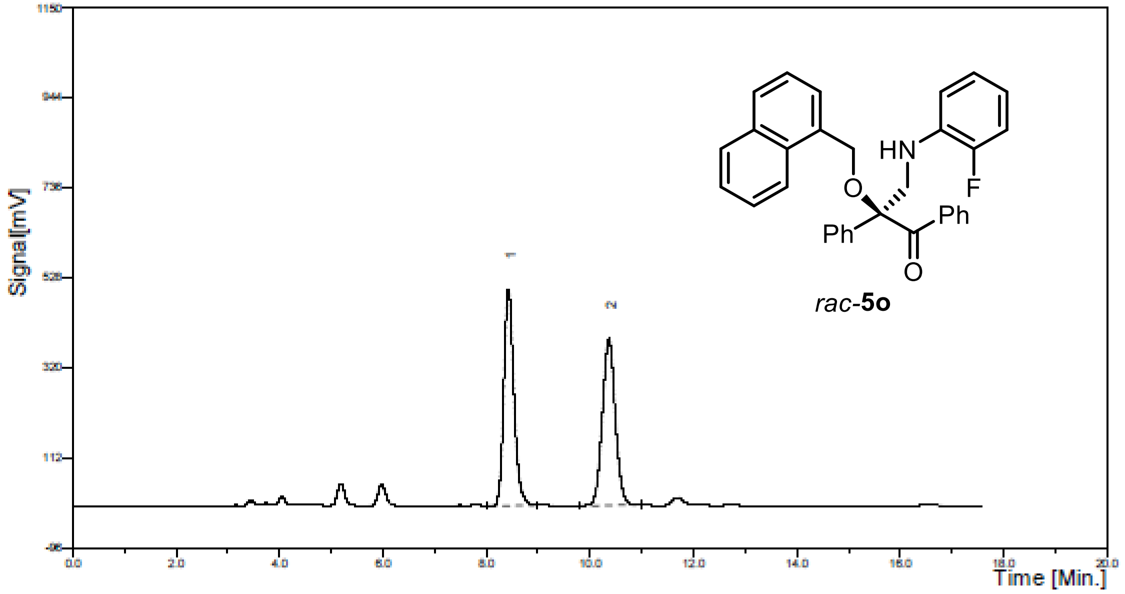

组分表

#	组分名	保留时间(min)	峰高(mV)	峰面积(mV. sec)	面积百分比(%)	浓 度	样品含量(%)
1	Unknown	8. 43000	499. 02	6779. 06	50. 3010	0. 0000	100. 0000
2	Unknown	10. 36250	383. 88	6697. 94	49. 6990	0. 0000	100. 0000
合计			882. 91	13477. 00	100		

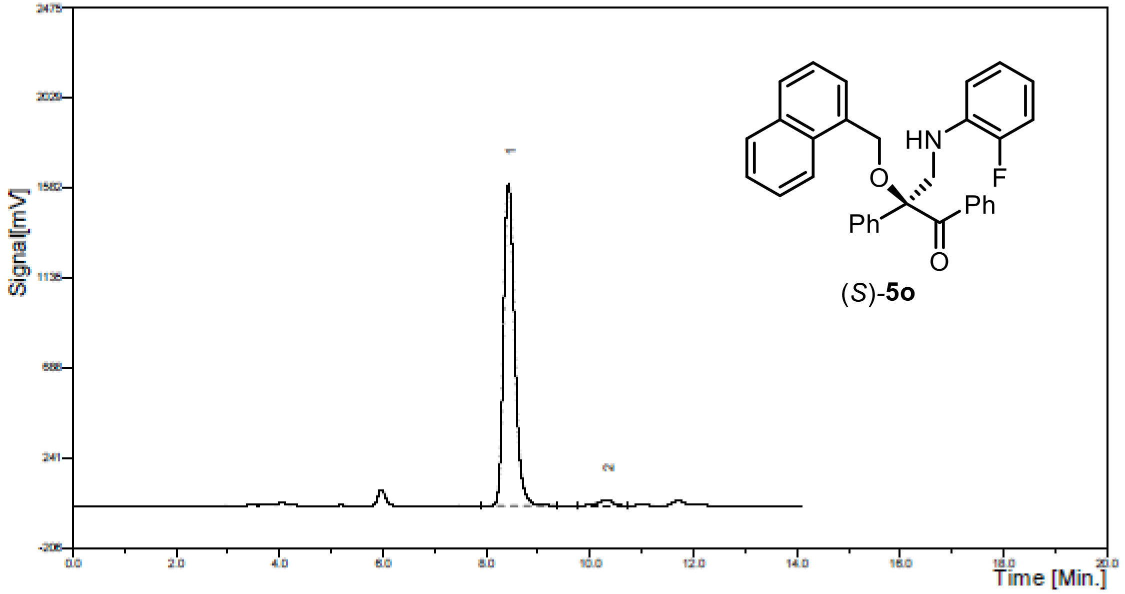

组分表

#	组分名	保留时间(min)	峰高(mV)	峰面积(mV. sec)	面积百分比(%)	浓 度	样品含量(%)
1	Unknown	8. 43167	1599. 97	24081. 09	97. 3945	0. 0000	0. 0000
2	Unknown	10. 31667	28. 97	644. 21	2. 6055	0. 0000	100. 0000
合计			1628. 94	24725. 30	100		

Condition: hexane/2-propanol = 80:1
Flow rate = 1.0 mL/min
λ = 254 nm
Chiral IA

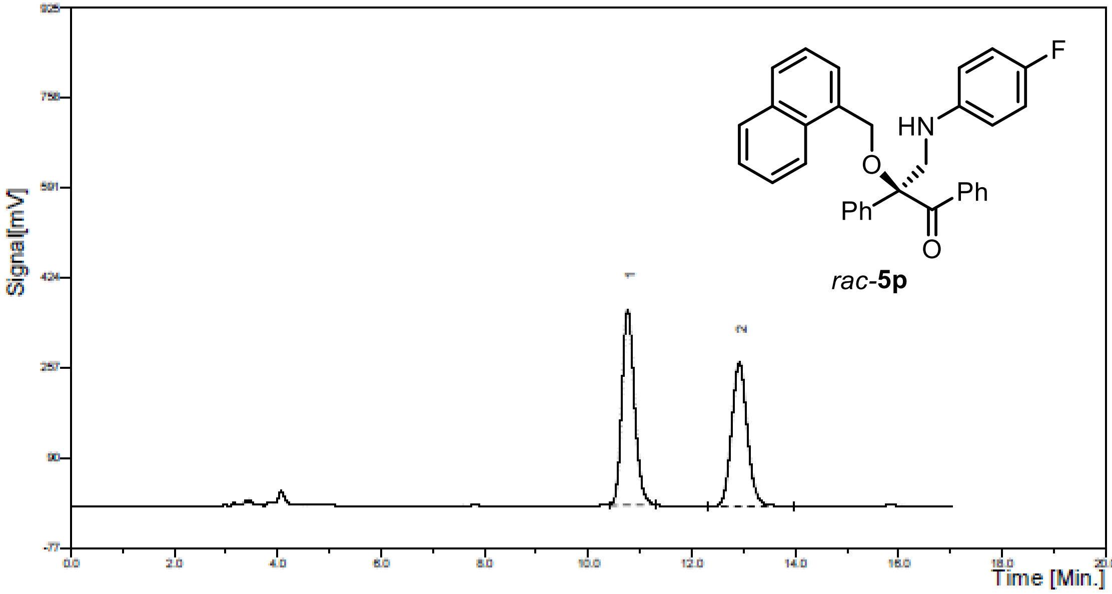

组分表

#	组分名	保留时间(min)	峰高(mV)	峰面积(mV. sec)	面积百分比(%)	浓 度	样品含量(%)
1	Unknown	10. 76000	362. 70	6138. 41	52. 1189	0. 0000	0. 0000
2	Unknown	12. 92083	266. 50	5639. 29	47. 8811	0. 0000	100. 0000
合计			629. 19	11777. 70	100		

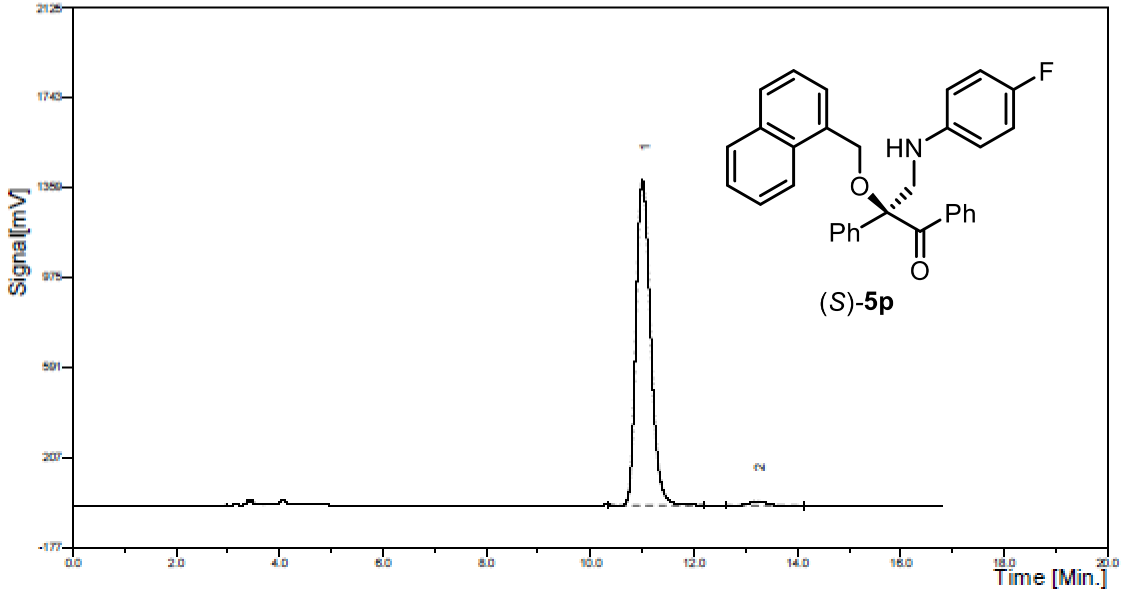

组分表

#	组分名	保留时间(min)	峰高(mV)	峰面积(mV. sec)	面积百分比(%)	浓 度	样品含量(%)
1	Unknown	11. 00500	1384. 42	27079. 66	98. 3485	0. 0000	100. 0000
2	Unknown	13. 22083	19. 54	454. 74	1. 6515	0. 0000	100. 0000
合计			1403. 96	27534. 40	100		

Condition: hexane/2-propanol = 40:1
Flow rate = 1.0 mL/min
λ = 254 nm
Chiral IA

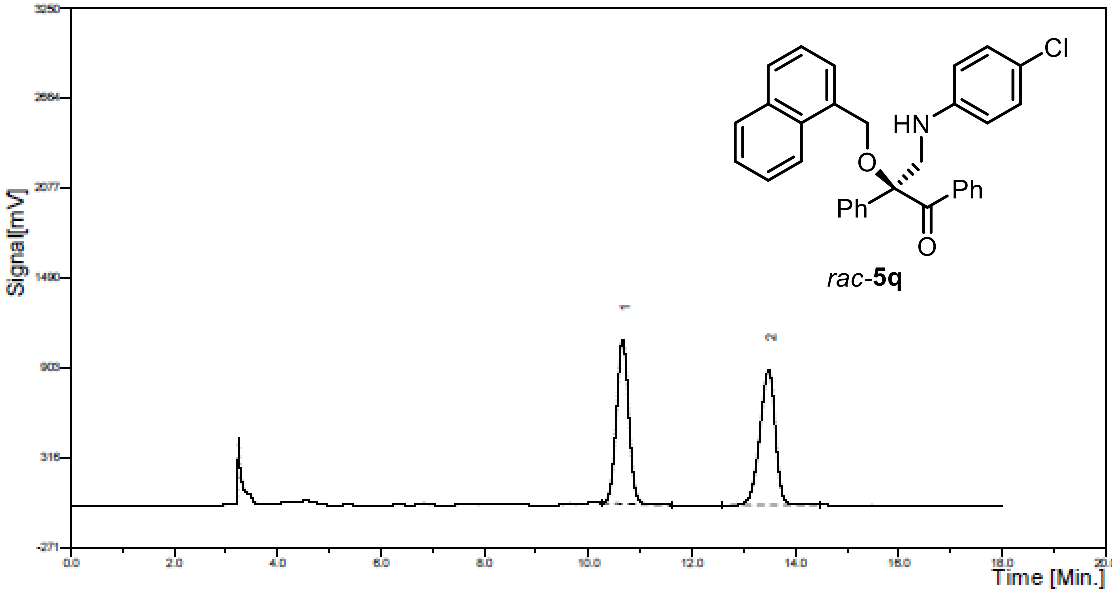

组分表

#	组分名	保留时间(min)	峰高(mV)	峰面积(mV.sec)	面积百分比(%)	浓 度	样品含量(%)
1	Unknown	10.65917	1068.30	17802.05	49.1948	0.0000	0.0000
2	Unknown	13.48333	881.38	18384.81	50.8052	0.0000	0.0000
合计			1949.68	36186.86	100		

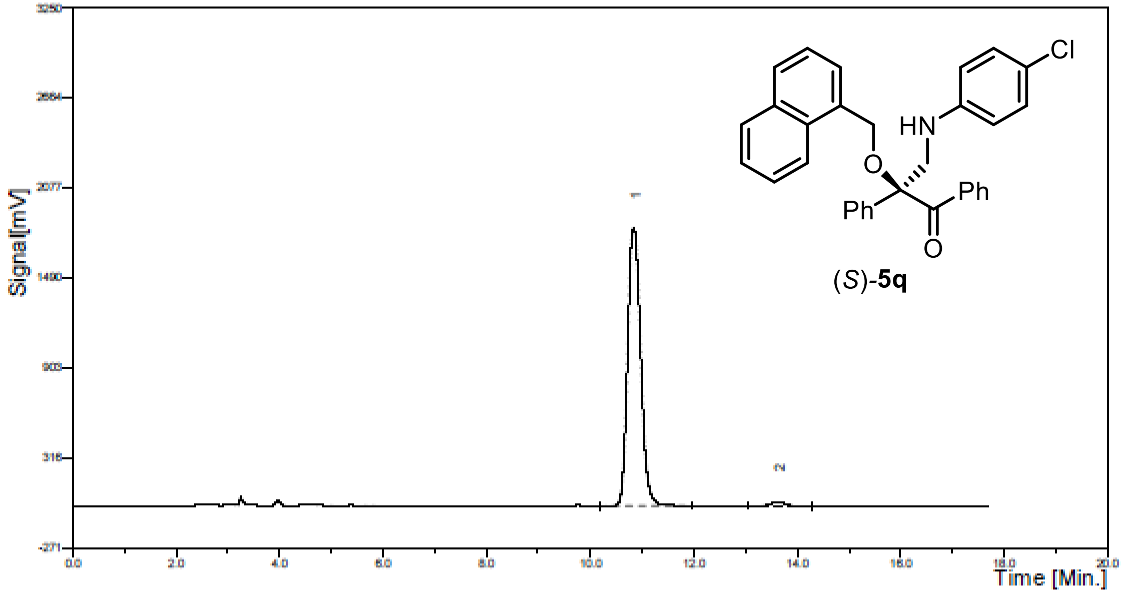

组分表

#	组分名	保留时间(min)	峰高(mV)	峰面积(mV.sec)	面积百分比(%)	浓 度	样品含量(%)
1	Unknown	10.83417	1812.59	31992.89	98.3179	0.0000	100.0000
2	Unknown	13.61417	28.95	547.38	1.6821	0.0000	100.0000
合计			1841.54	32540.27	100		

Condition: hexane/2-propanol = 40:1
Flow rate = 1.0 mL/min
λ = 254 nm
Chiral IA

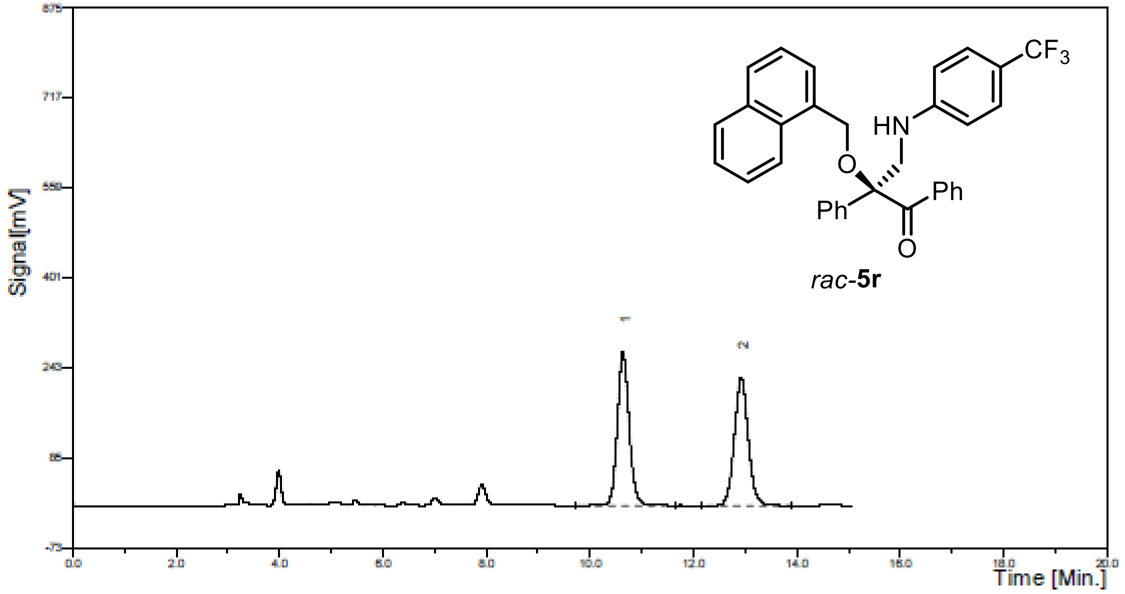

组分表

#	组分名	保留时间(min)	峰高(mV)	峰面积(mV. sec)	面积百分比(%)	浓 度	样品含量(%)
1	Unknown	10. 63750	269. 43	4319. 58	50. 6156	0. 0000	100. 0000
2	Unknown	12. 92500	223. 53	4214. 51	49. 3844	0. 0000	100. 0000
合计			492. 96	8534. 09	100		

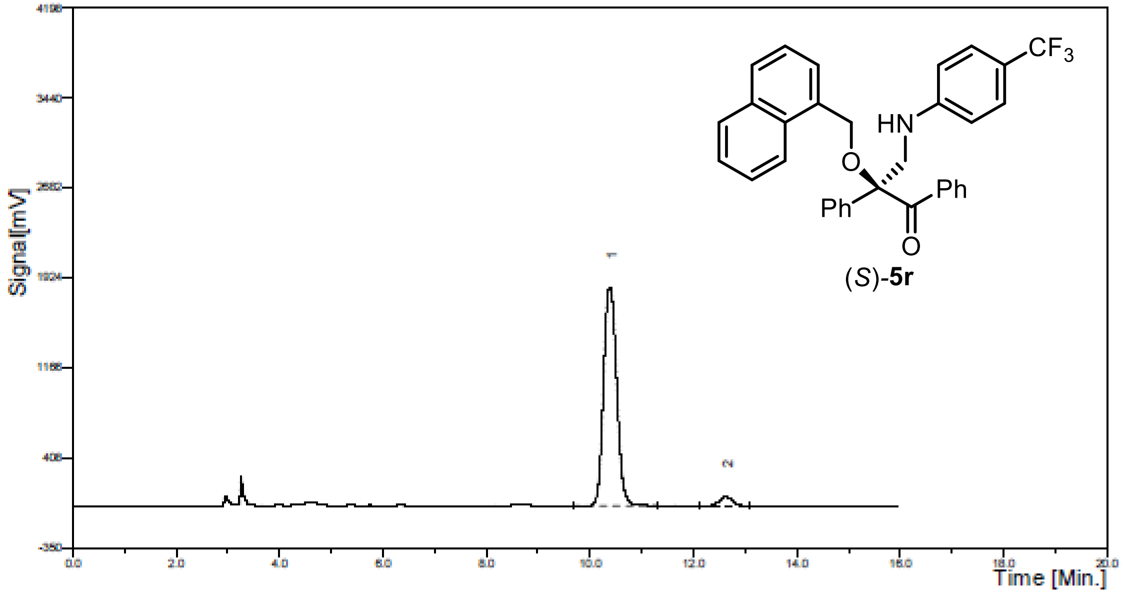

组分表

#	组分名	保留时间(min)	峰高(mV)	峰面积(mV. sec)	面积百分比(%)	浓 度	样品含量(%)
1	Unknown	10. 38083	1837. 47	32651. 27	95. 9604	0. 0000	100. 0000
2	Unknown	12. 62500	76. 45	1374. 51	4. 0396	0. 0000	100. 0000
合计			1913. 92	34025. 78	100		

Condition: hexane/2-propanol = 10:1
Flow rate = 1.0 mL/min
λ = 254 nm
Chiral IA

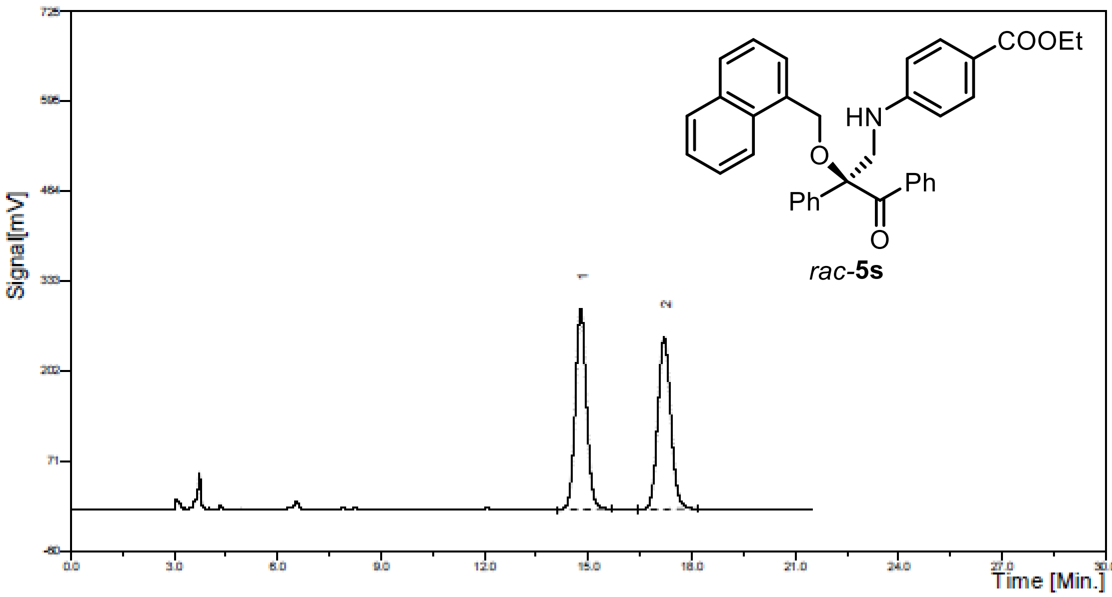

组分表

#	组分名	保留时间(min)	峰高(mV)	峰面积(mV.sec)	面积百分比(%)	浓 度	样品含量(%)
1	Unknown	14.77917	290.63	6597.88	50.0604	0.0000	100.0000
2	Unknown	17.19750	249.85	6581.96	49.9396	0.0000	100.0000
合计			540.48	13179.84	100		

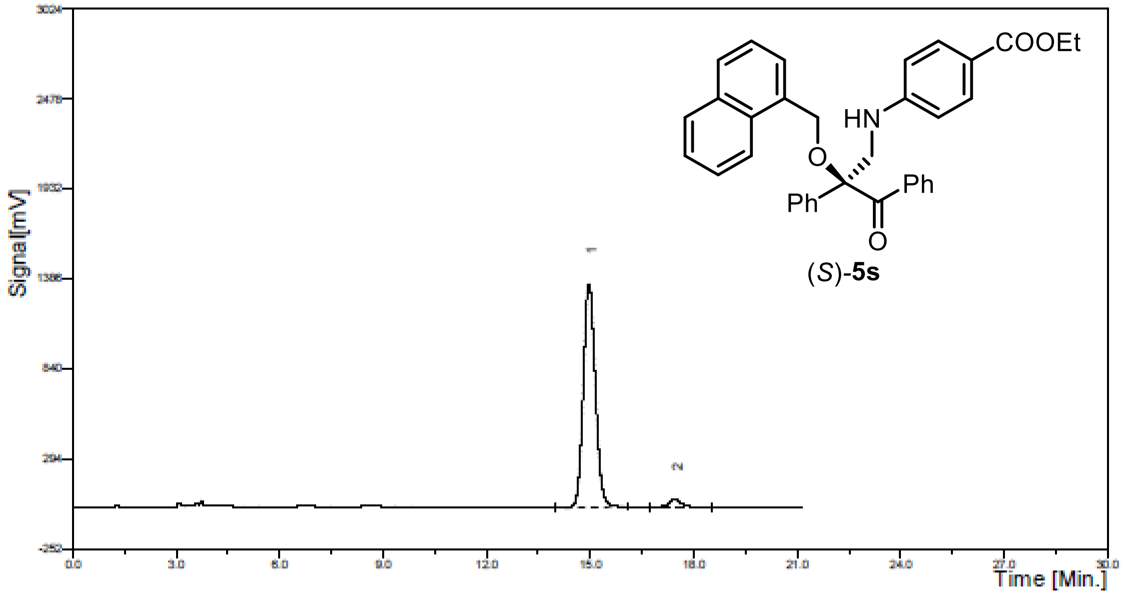

组分表

#	组分名	保留时间(min)	峰高(mV)	峰面积(mV.sec)	面积百分比(%)	浓 度	样品含量(%)
1	Unknown	14.96083	1354.84	33027.68	96.4534	0.0000	100.0000
2	Unknown	17.45417	45.06	1214.42	3.5466	0.0000	100.0000
合计			1399.90	34242.11	100		

Condition: hexane/2-propanol = 40:1
Flow rate = 1.0 mL/min
λ = 254 nm
Chiral IA

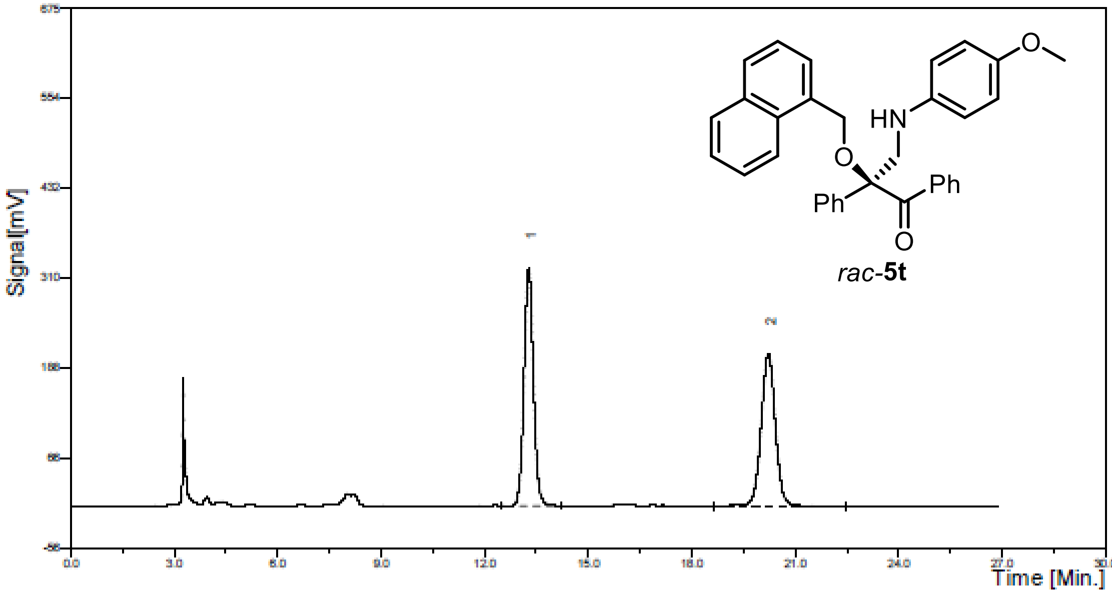

组分表

#	组分名	保留时间(min)	峰高(mV)	峰面积(mV. sec)	面积百分比(%)	浓 度	样品含量(%)
1	Unknown	13. 28333	320. 98	6125. 09	50. 5747	0. 0000	100. 0000
2	Unknown	20. 22167	206. 13	5985. 88	49. 4253	0. 0000	0. 0000
合计			527. 11	12110. 97	100		

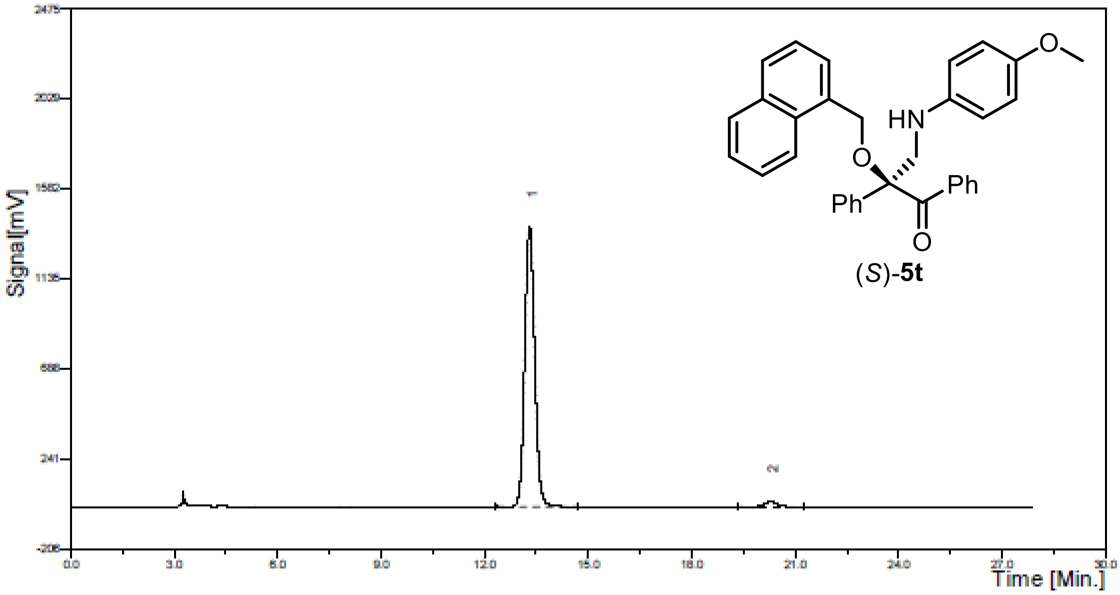

组分表

#	组分名	保留时间(min)	峰高(mV)	峰面积(mV. sec)	面积百分比(%)	浓 度	样品含量(%)
1	Unknown	13. 30917	1385. 68	27736. 75	97. 4439	0. 0000	100. 0000
2	Unknown	20. 29750	27. 03	727. 58	2. 5561	0. 0000	100. 0000
合计			1412. 71	28464. 34	100		

Condition: hexane/2-propanol = 40:1
Flow rate = 1.0 mL/min
λ = 254 nm
Chiral IA

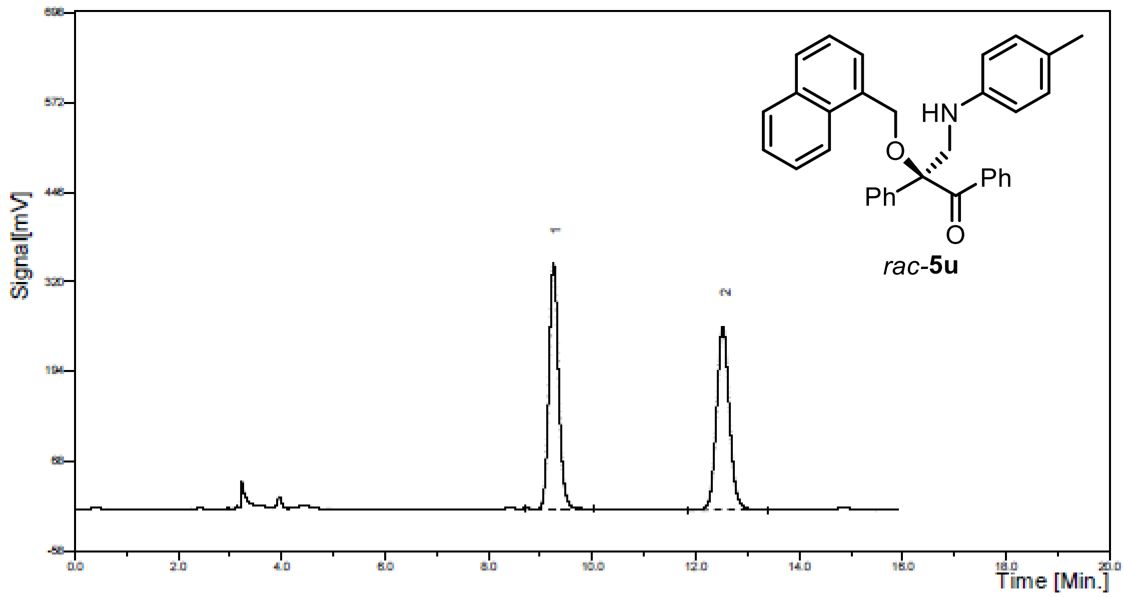

组分表

#	组分名	保留时间(min)	峰高(mV)	峰面积(mV. sec)	面积百分比(%)	浓 度	样品含量(%)
1	Unknown	9. 26000	344. 59	4461. 93	50. 1141	0. 0000	100. 0000
2	Unknown	12. 52500	257. 35	4441. 62	49. 8859	0. 0000	100. 0000
合计			601. 94	8903. 55	100		

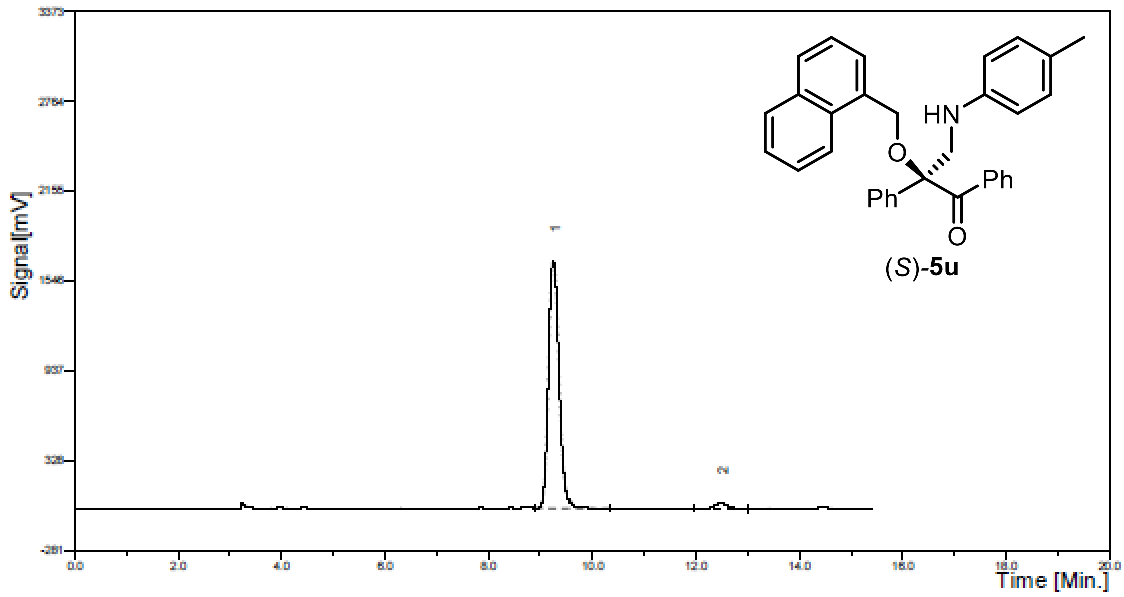

组分表

#	组分名	保留时间(min)	峰高(mV)	峰面积(mV. sec)	面积百分比(%)	浓 度	样品含量(%)
1	Unknown	9. 26000	1681. 15	24134. 73	97. 3922	0. 0000	100. 0000
2	Unknown	12. 48417	38. 86	646. 24	2. 6078	0. 0000	100. 0000
合计			1720. 01	24780. 97	100		

Condition: hexane/2-propanol = 80:1
Flow rate = 1.0 mL/min
λ = 254 nm
Chiral IA

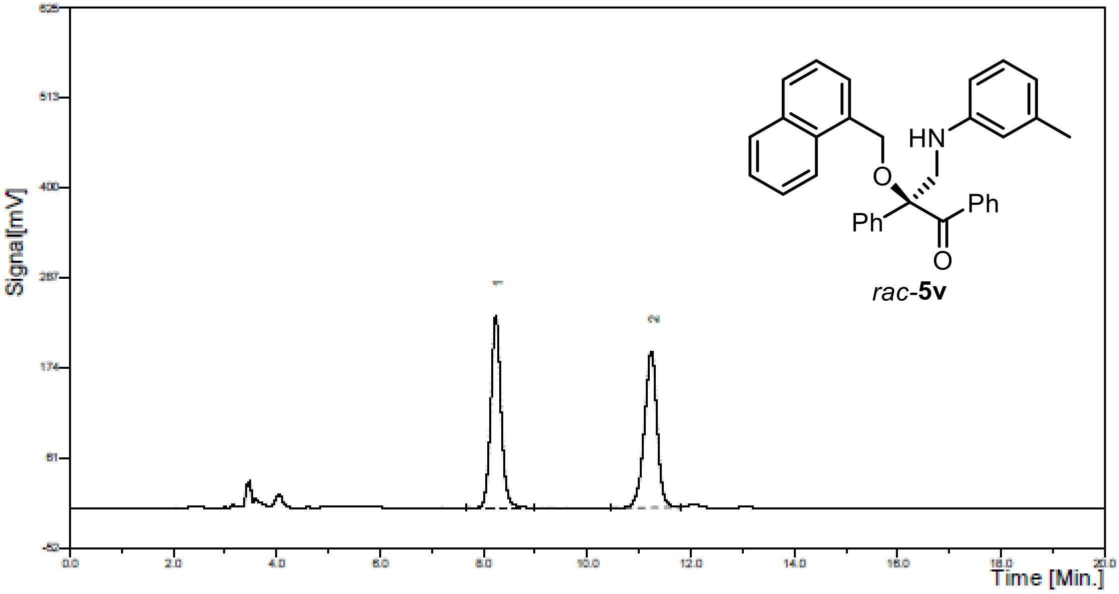

组分表

#	组分名	保留时间(min)	峰高(mV)	峰面积(mV. sec)	面积百分比(%)	浓 度	样品含量(%)
1	Unknown	8. 23917	239. 94	3344. 86	49. 6766	0. 0000	100. 0000
2	Unknown	11. 23750	195. 64	3388. 41	50. 3234	0. 0000	100. 0000
合计			435. 58	6733. 27	100		

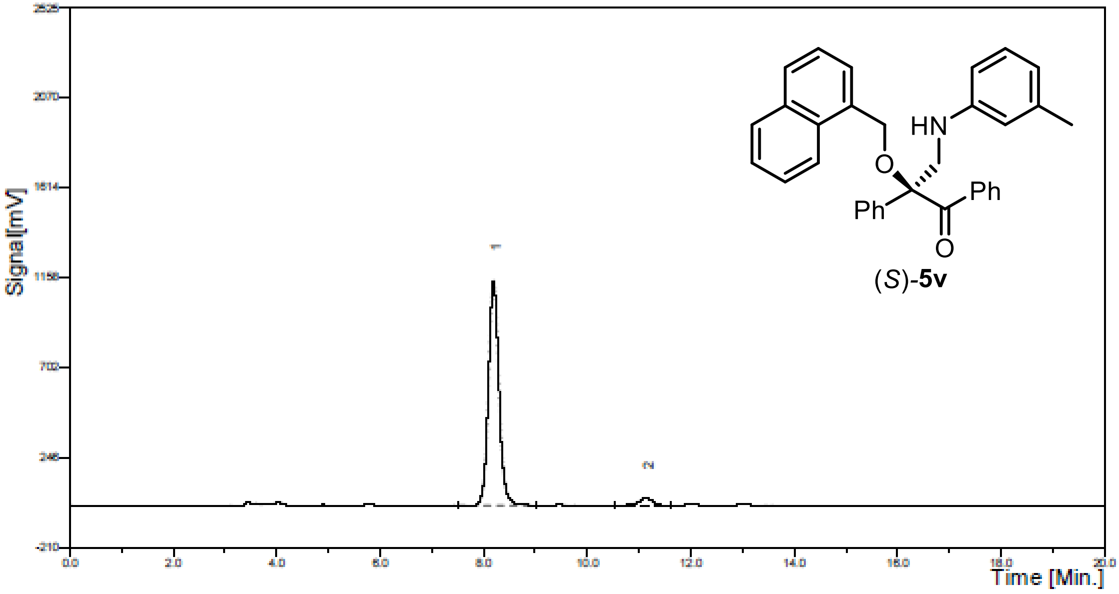

组分表

#	组分名	保留时间(min)	峰高(mV)	峰面积(mV. sec)	面积百分比(%)	浓 度	样品含量(%)
1	Unknown	8. 19500	1138. 50	16210. 18	95. 7947	0. 0000	100. 0000
2	Unknown	11. 13833	38. 91	711. 61	4. 2053	0. 0000	100. 0000
合计			1177. 41	16921. 79	100		

Condition: hexane/2-propanol = 40:1
Flow rate = 1.0 mL/min
λ = 254 nm
Chiral IA

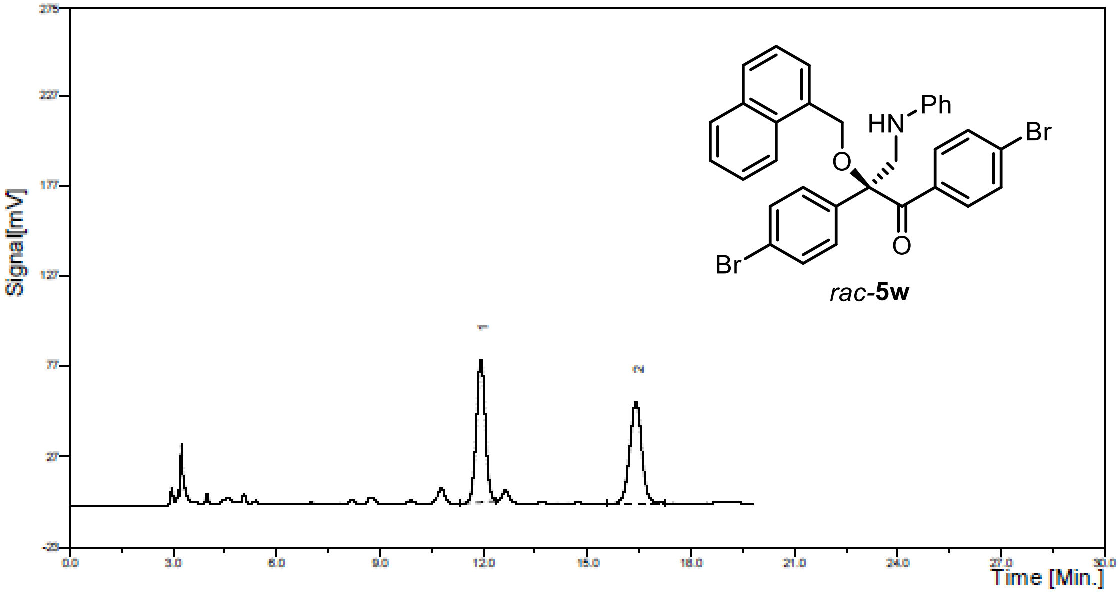

组分表

#	组分名	保留时间(min)	峰高(mV)	峰面积(mV.sec)	面积百分比(%)	浓 度	样品含量(%)
1	Unknown	11.93250	78.97	1430.24	51.4680	0.0000	100.0000
2	Unknown	16.40750	55.48	1348.65	48.5320	0.0000	100.0000
合计			134.46	2778.88	100		

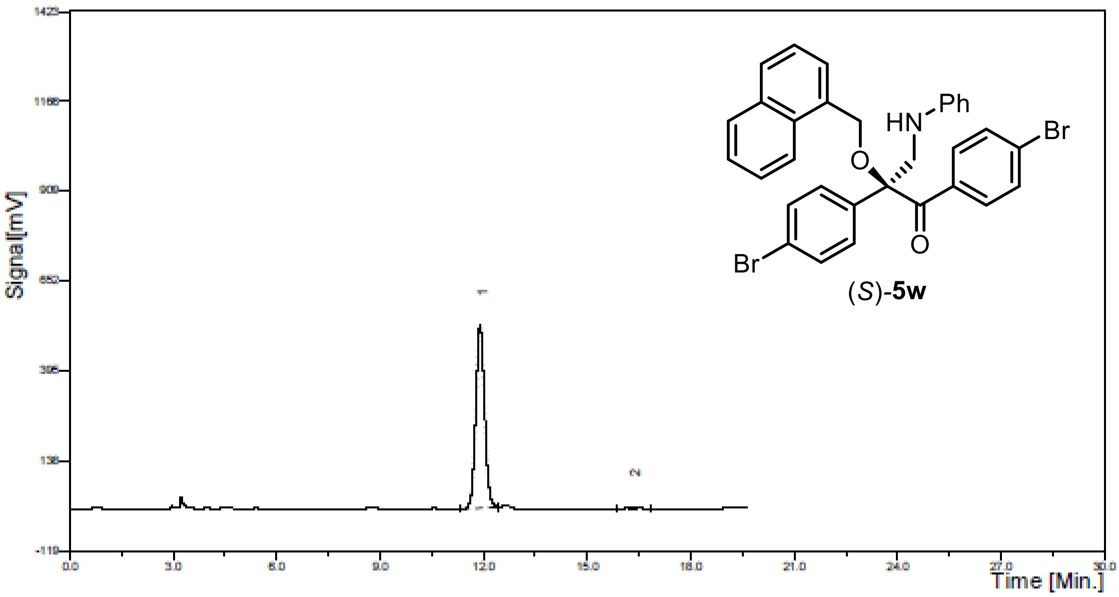

组分表

#	组分名	保留时间(min)	峰高(mV)	峰面积(mV.sec)	面积百分比(%)	浓 度	样品含量(%)
1	Unknown	11.90250	521.36	9303.48	98.3491	0.0000	100.0000
2	Unknown	16.32833	6.71	156.17	1.6509	0.0000	100.0000
合计			528.07	9459.64	100		

Condition: hexane/2-propanol = 40:1
Flow rate = 1.0 mL/min
λ = 254 nm
Chiral IA

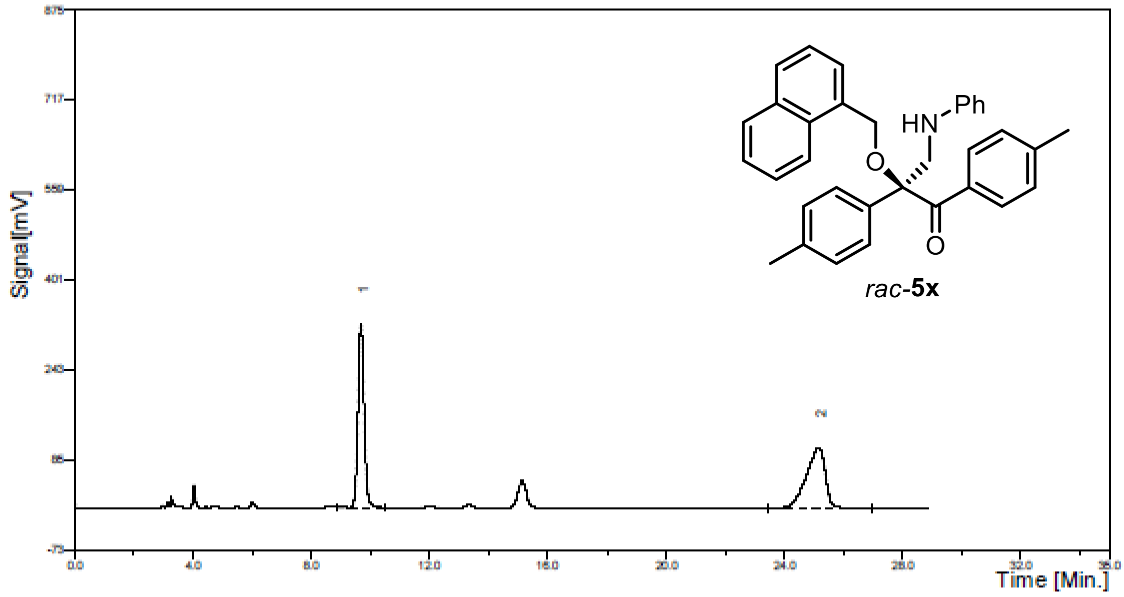

组分表

#	组分名	保留时间(min)	峰高(mV)	峰面积(mV.sec)	面积百分比(%)	浓 度	样品含量(%)
1	Unknown	9.68833	323.57	4665.24	49.5129	0.0000	100.0000
2	Unknown	25.16750	106.74	4757.03	50.4871	0.0000	100.0000
合计			430.31	9422.26	100		

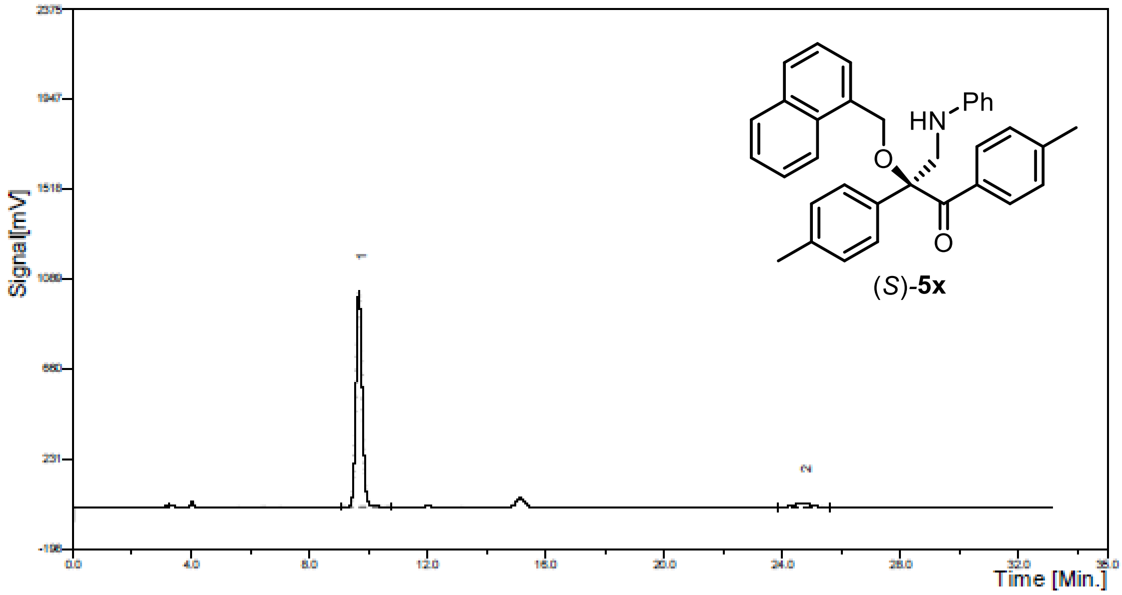

组分表

#	组分名	保留时间(min)	峰高(mV)	峰面积(mV.sec)	面积百分比(%)	浓 度	样品含量(%)
1	Unknown	9.68250	1033.96	15176.34	94.8015	0.0000	0.0000
2	Unknown	24.72500	22.23	832.21	5.1985	0.0000	100.0000
合计			1056.19	16008.55	100		

Condition: hexane/2-propanol = 3:1
Flow rate = 1.0 mL/min
λ = 254 nm
Chiral IC

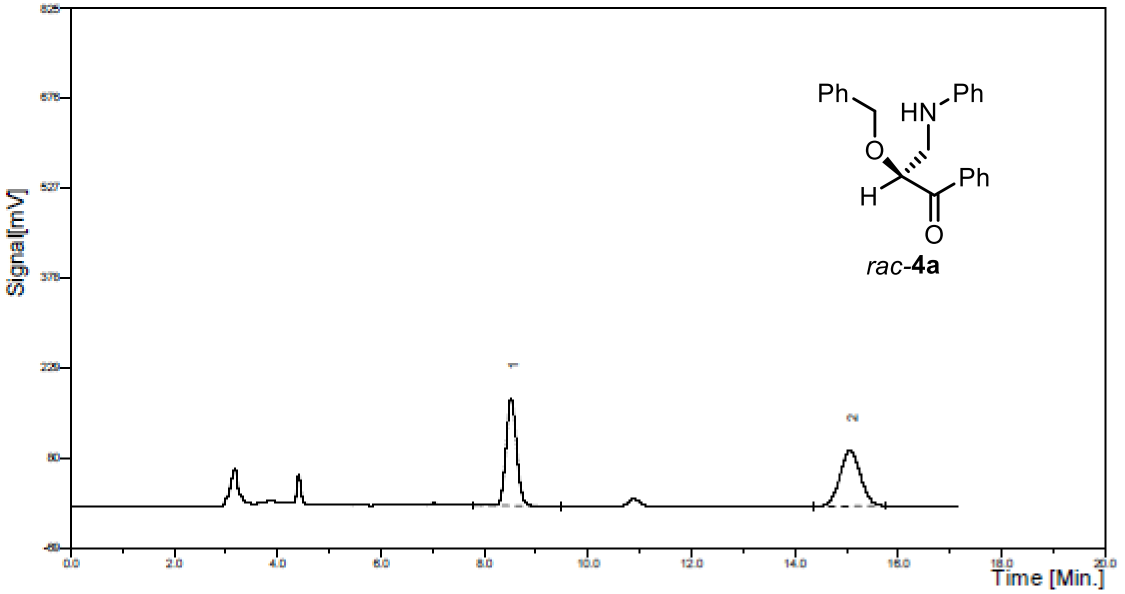

组分表

#	组分名	保留时间(min)	峰高(mV)	峰面积(mV.sec)	面积百分比(%)	浓 度	样品含量(%)
1	Unknown	8.52083	178.00	2533.75	49.8381	0.0000	100.0000
2	Unknown	15.07000	92.26	2550.20	50.1619	0.0000	0.0000
合计			270.25	5083.95	100		

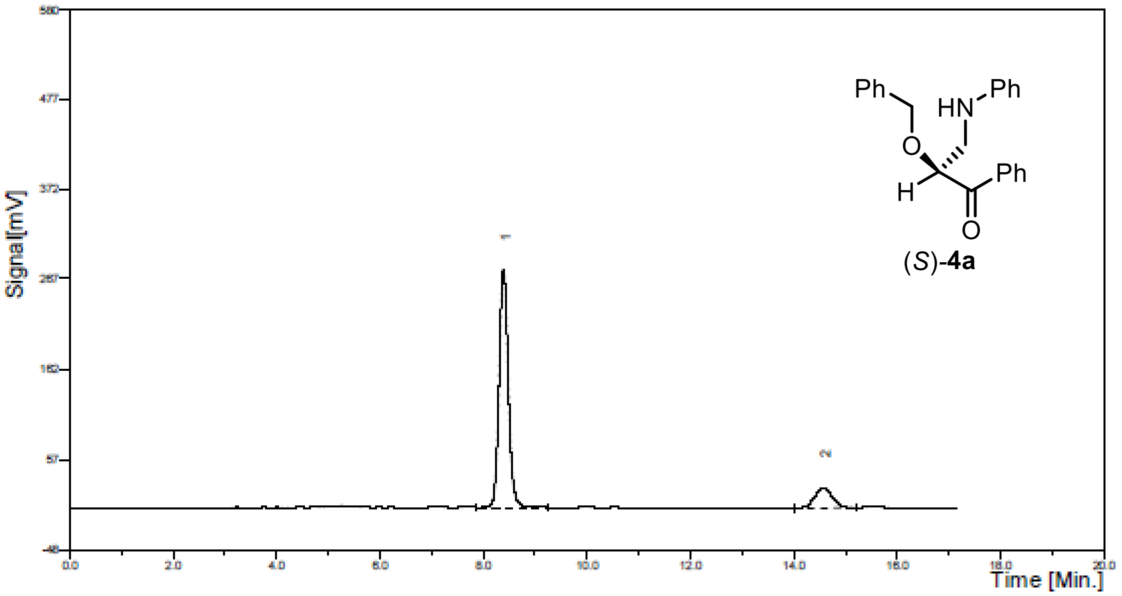

组分表

#	组分名	保留时间(min)	峰高(mV)	峰面积(mV.sec)	面积百分比(%)	浓 度	样品含量(%)
1	Unknown	8.39250	276.84	3426.64	85.9551	0.0000	100.0000
2	Unknown	14.57083	23.82	559.90	14.0449	0.0000	0.0000
合计			300.66	3986.55	100		

Condition: hexane/2-propanol = 10:1
Flow rate = 1.0 mL/min
λ = 254 nm
Chiral IC

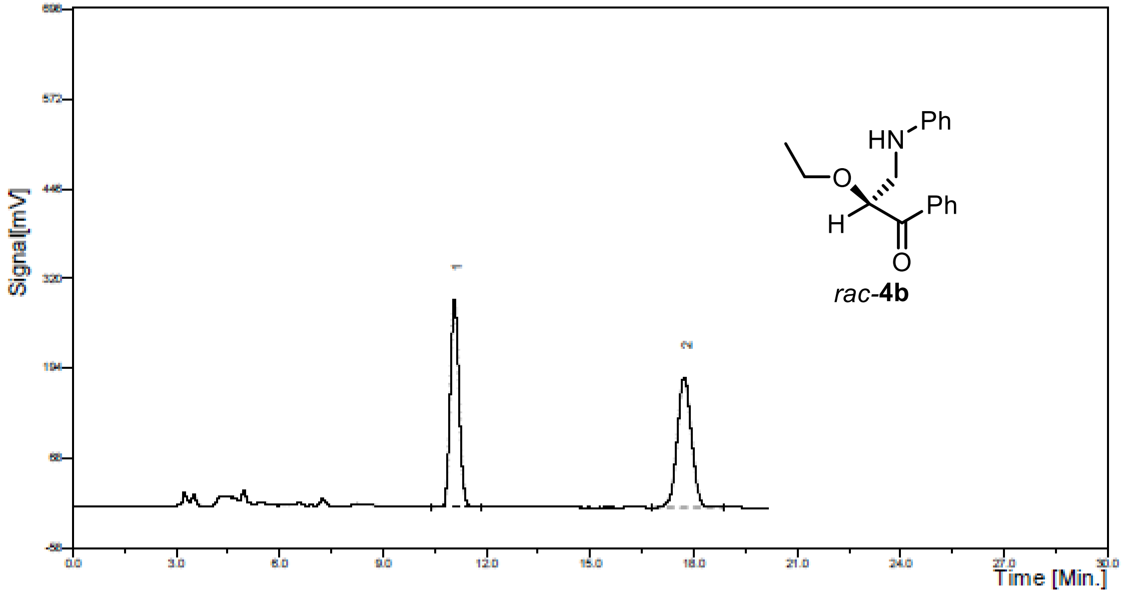

组分表

#	组分名	保留时间(min)	峰高(mV)	峰面积(mV.sec)	面积百分比(%)	浓 度	样品含量(%)
1	Unknown	11.07000	290.12	5141.36	49.4236	0.0000	100.0000
2	Unknown	17.72250	181.69	5261.28	50.5764	0.0000	100.0000
合计			471.81	10402.63	100		

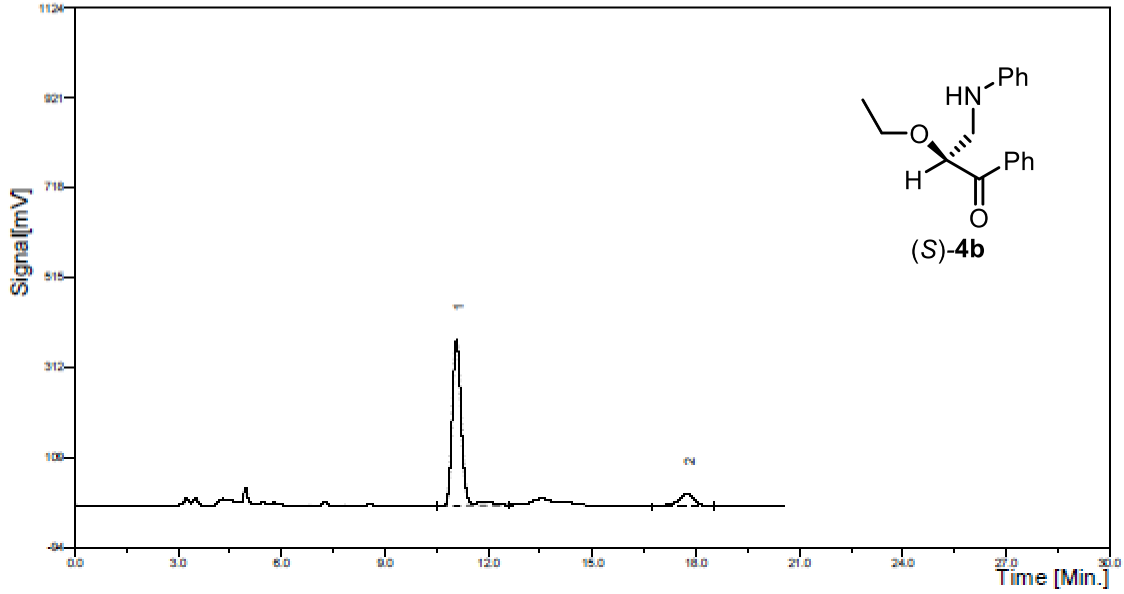

组分表

#	组分名	保留时间(min)	峰高(mV)	峰面积(mV.sec)	面积百分比(%)	浓 度	样品含量(%)
1	Unknown	11.07500	373.66	7003.83	89.1802	0.0000	100.0000
2	Unknown	17.74833	26.88	849.74	10.8198	0.0000	100.0000
合计			400.54	7853.57	100		

Condition: hexane/2-propanol = 10:1
Flow rate = 1.0 mL/min
λ = 254 nm
Chiral IC

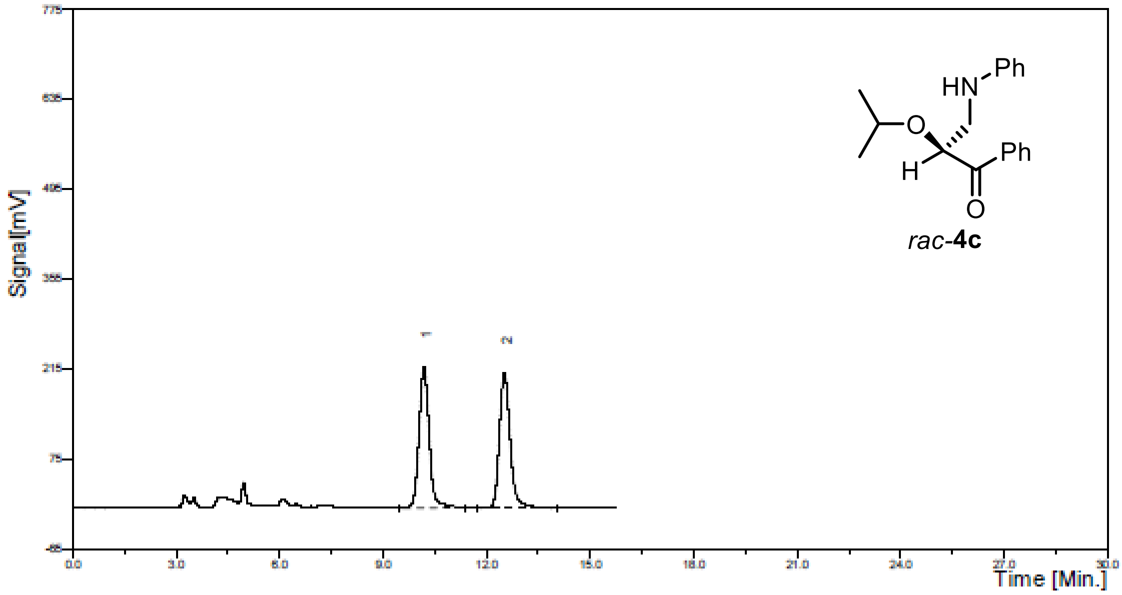

组分表

#	组分名	保留时间(min)	峰高(mV)	峰面积(mV. sec)	面积百分比(%)	浓 度	样品含量(%)
1	Unknown	10. 18667	217. 87	4463. 88	49. 5413	0. 0000	100. 0000
2	Unknown	12. 52000	208. 01	4546. 53	50. 4587	0. 0000	100. 0000
合计			425. 88	9010. 41	100		

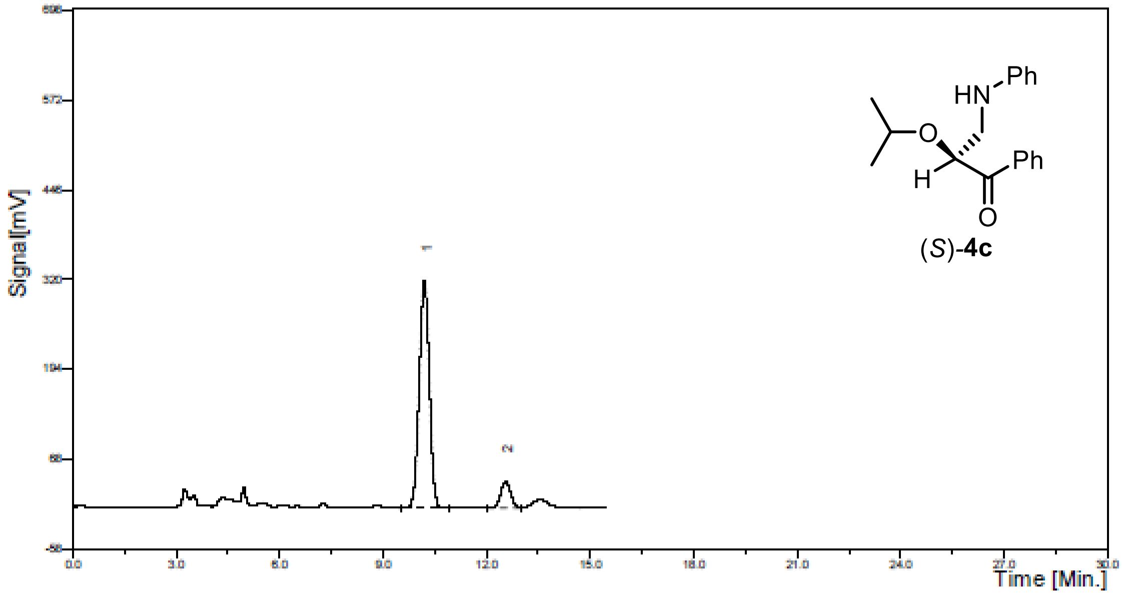

组分表

#	组分名	保留时间(min)	峰高(mV)	峰面积(mV. sec)	面积百分比(%)	浓 度	样品含量(%)
1	Unknown	10. 19833	317. 91	6517. 09	89. 7805	0. 0000	100. 0000
2	Unknown	12. 55417	37. 33	741. 82	10. 2195	0. 0000	100. 0000
合计			355. 24	7258. 92	100		

Condition: hexane/2-propanol = 20:1
Flow rate = 1.0 mL/min
λ = 254 nm
Chiral IA

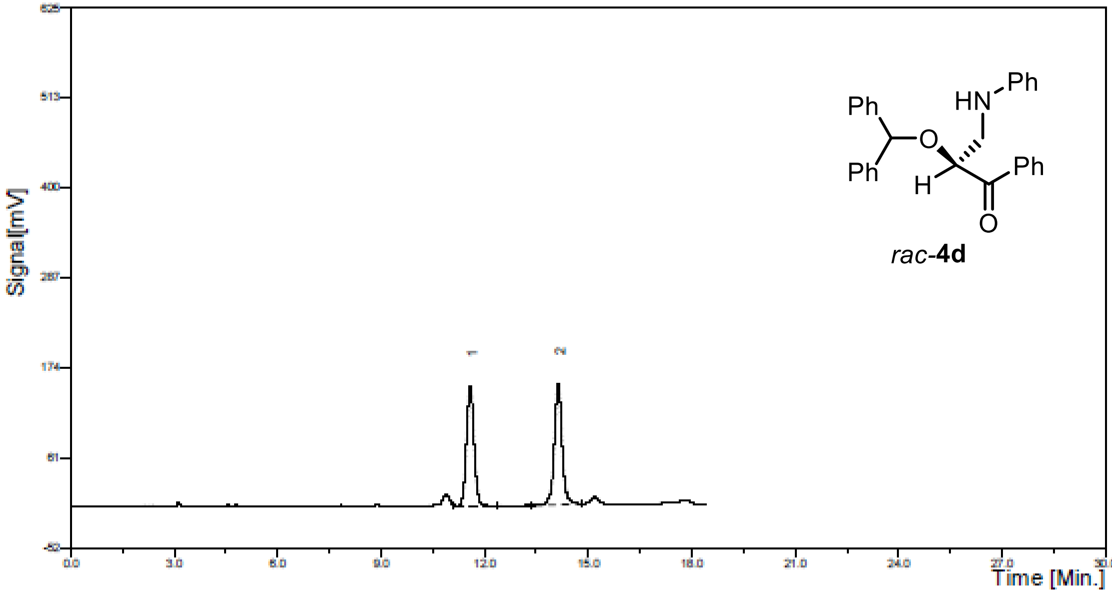

组分表

#	组分名	保留时间(min)	峰高(mV)	峰面积(mV.sec)	面积百分比(%)	浓 度	样品含量(%)
1	Unknown	11.58583	148.65	2257.50	48.7761	0.0000	100.0000
2	Unknown	14.14000	150.79	2370.79	51.2239	0.0000	100.0000
合计			299.44	4628.29	100		

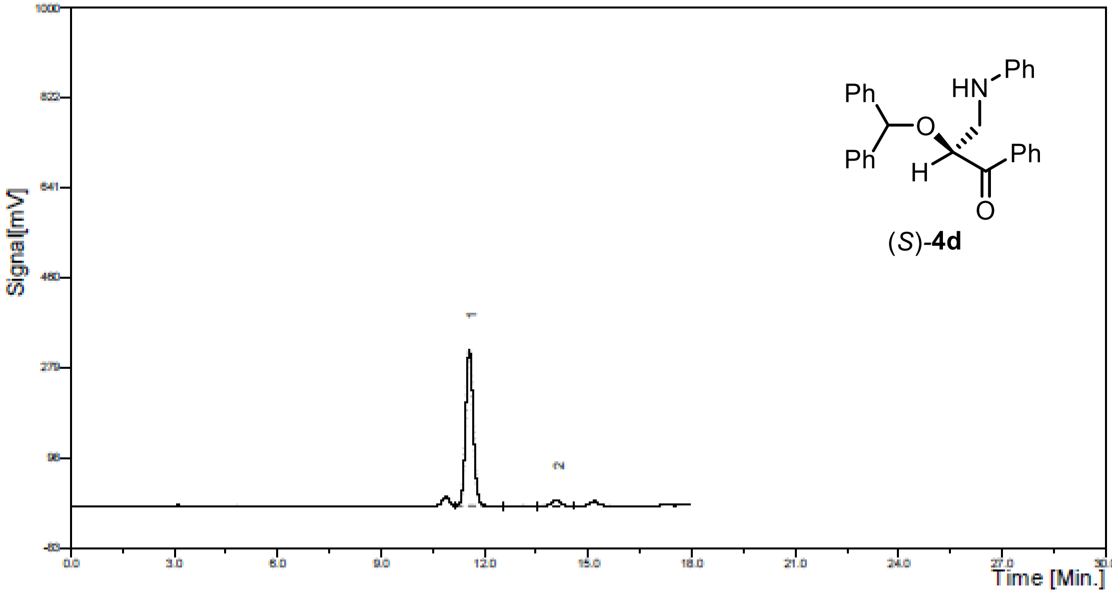

组分表

#	组分名	保留时间(min)	峰高(mV)	峰面积(mV.sec)	面积百分比(%)	浓 度	样品含量(%)
1	Unknown	11.56167	314.53	4825.49	95.6088	0.0000	100.0000
2	Unknown	14.09917	13.19	221.63	4.3912	0.0000	100.0000
合计			327.72	5047.12	100		

Condition: hexane/2-propanol = 5:1
Flow rate = 1.0 mL/min
λ = 254 nm
Chiral IC

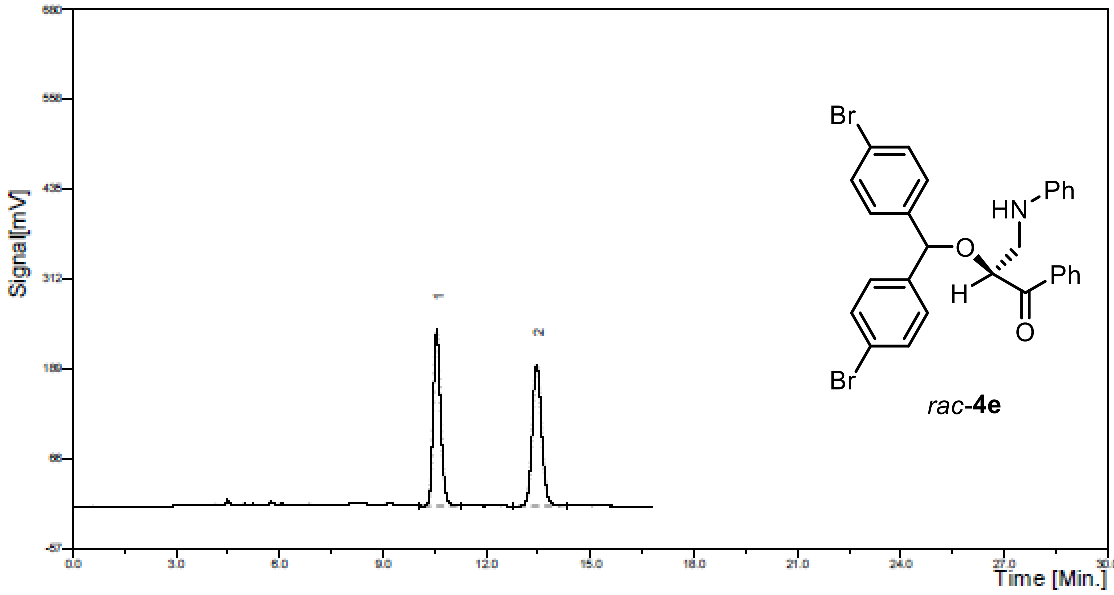

组分表

#	组分名	保留时间(min)	峰高(mV)	峰面积(mV. sec)	面积百分比(%)	浓 度	样品含量(%)
1	Unknown	10. 56083	241. 57	3616. 72	49. 5879	0. 0000	100. 0000
2	Unknown	13. 46417	193. 07	3676. 83	50. 4121	0. 0000	100. 0000
合计			434. 63	7293. 56	100		

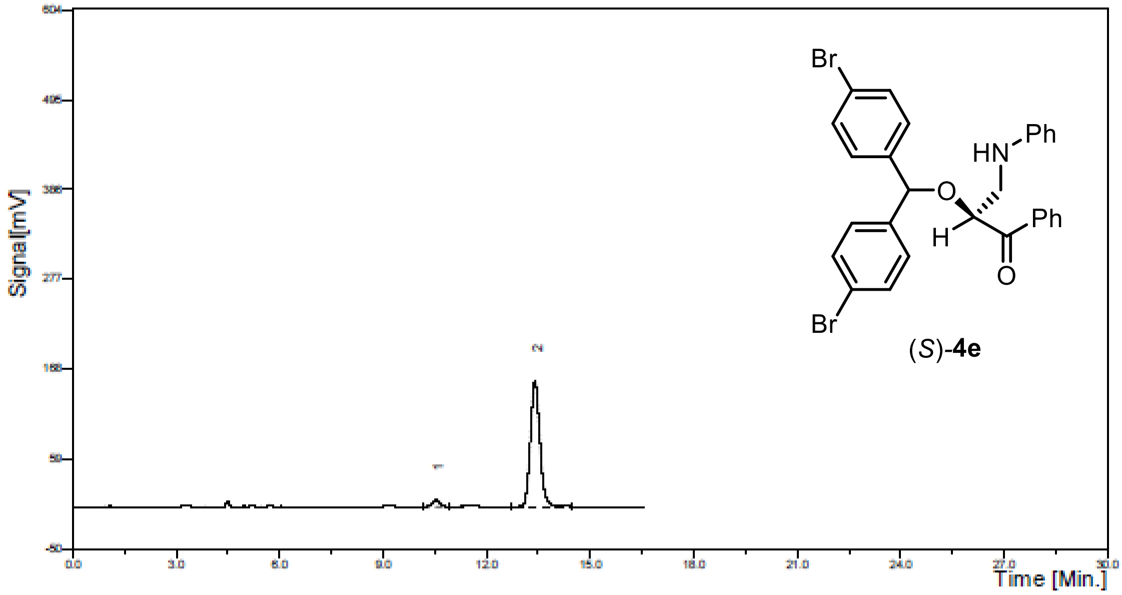

组分表

#	组分名	保留时间(min)	峰高(mV)	峰面积(mV. sec)	面积百分比(%)	浓 度	样品含量(%)
1	Unknown	10. 52417	7. 94	117. 08	3. 8933	0. 0000	100. 0000
2	Unknown	13. 40500	151. 87	2890. 05	96. 1067	0. 0000	100. 0000
合计			159. 81	3007. 13	100		

Condition: hexane/2-propanol = 10:1
Flow rate = 1.0 mL/min
λ = 254 nm
Chiral IA

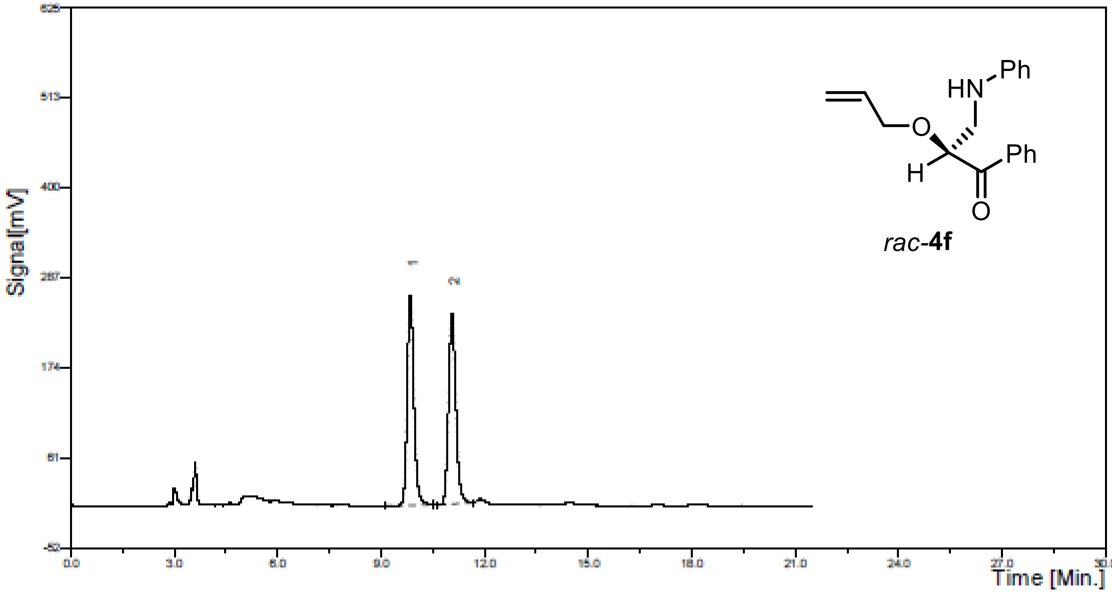

组分表

#	组分名	保留时间(min)	峰高(mV)	峰面积(mV. sec)	面积百分比(%)	浓 度	样品含量(%)
1	Unknown	9. 84750	261. 69	3538. 82	49. 8756	0. 0000	100. 0000
2	Unknown	11. 05583	238. 44	3556. 47	50. 1244	0. 0000	100. 0000
合计			500. 13	7095. 29	100		

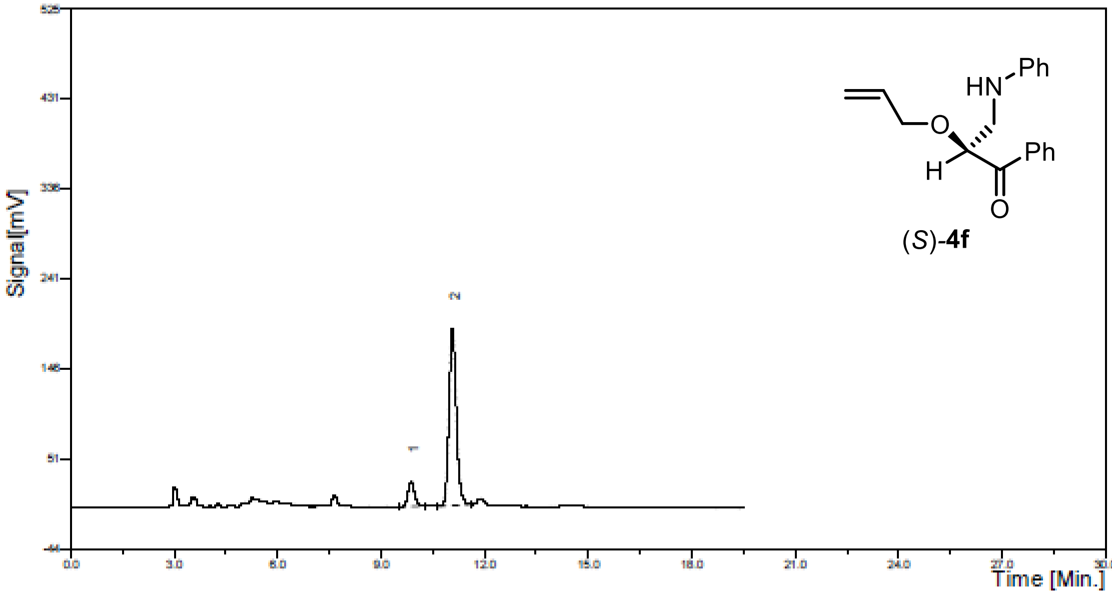

组分表

#	组分名	保留时间(min)	峰高(mV)	峰面积(mV. sec)	面积百分比(%)	浓 度	样品含量(%)
1	Unknown	9. 87333	26. 54	345. 66	11. 1647	0. 0000	100. 0000
2	Unknown	11. 07000	185. 50	2750. 33	88. 8353	0. 0000	100. 0000
合计			212. 04	3095. 99	100		

Condition: hexane/2-propanol = 10:1
Flow rate = 1.0 mL/min
λ = 254 nm
Chiral IA

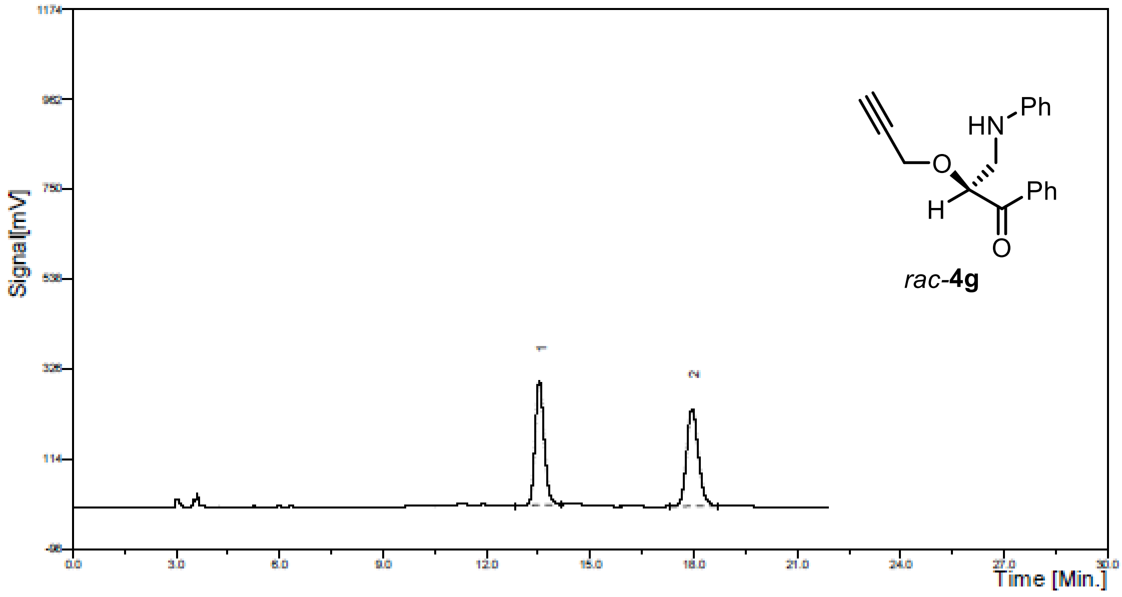

组分表

#	组分名	保留时间(min)	峰高(mV)	峰面积(mV.sec)	面积百分比(%)	浓 度	样品含量(%)
1	Unknown	13.53417	293.17	5360.07	49.3424	0.0000	0.0000
2	Unknown	17.94833	229.65	5502.93	50.6576	0.0000	100.0000
合计			522.82	10863.00	100		

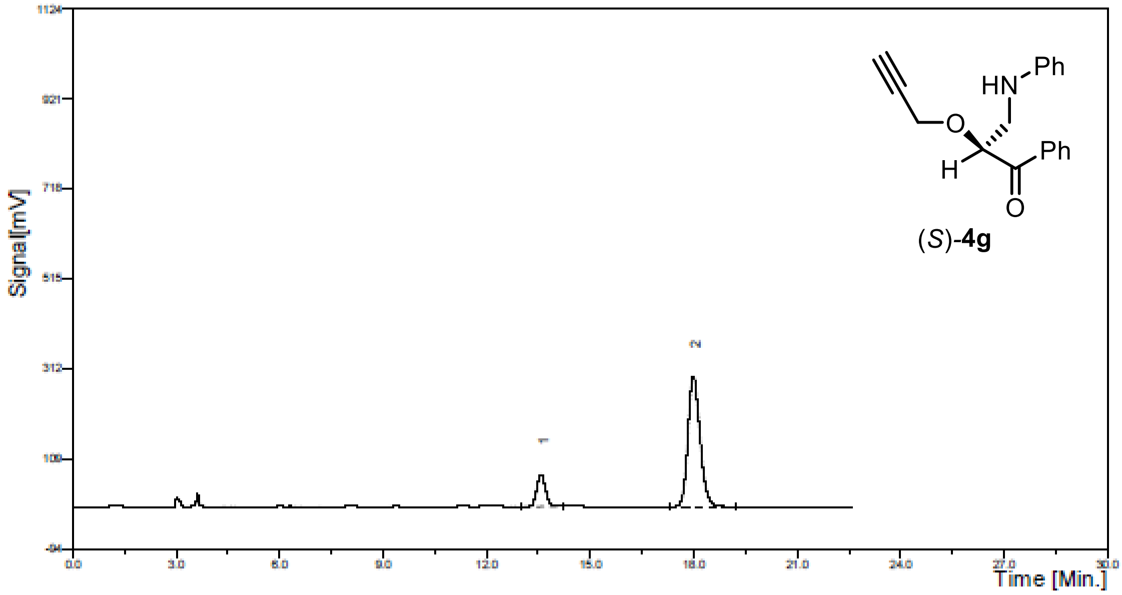

组分表

#	组分名	保留时间(min)	峰高(mV)	峰面积(mV.sec)	面积百分比(%)	浓 度	样品含量(%)
1	Unknown	13.58417	72.81	1295.80	15.1413	0.0000	100.0000
2	Unknown	17.99083	293.34	7262.27	84.8587	0.0000	100.0000
合计			366.15	8558.06	100		

Condition: hexane/2-propanol = 10:1
Flow rate = 1.0 mL/min
λ = 254 nm
Chiral IA

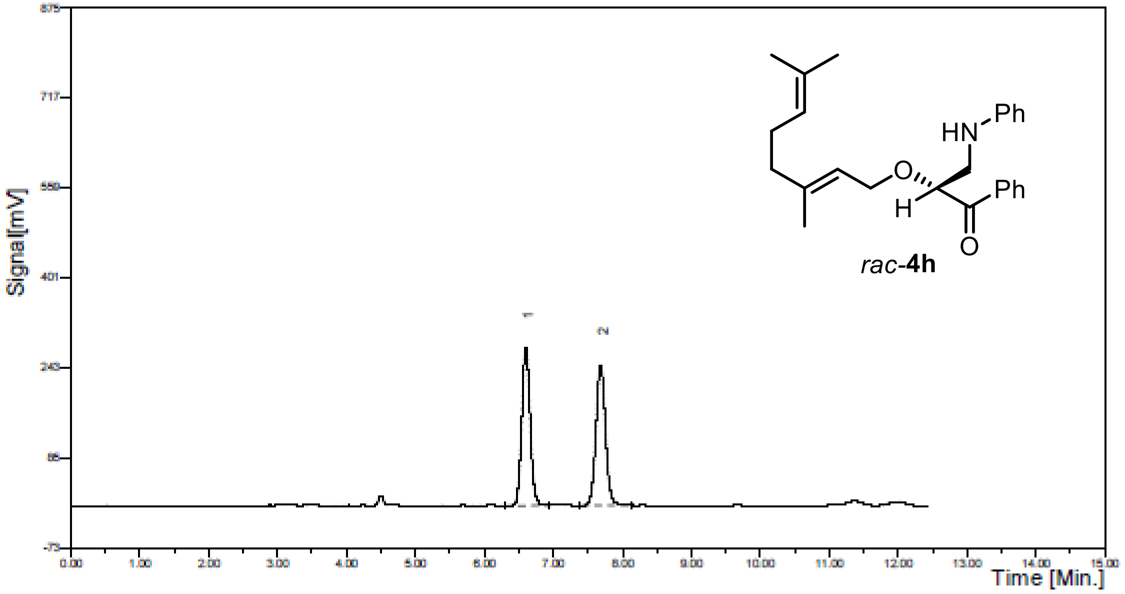

组分表

#	组分名	保留时间(min)	峰高(mV)	峰面积(mV.sec)	面积百分比(%)	浓 度	样品含量(%)
1	Unknown	6.60500	276.14	2349.43	49.7349	0.0000	100.0000
2	Unknown	7.68250	245.89	2374.49	50.2651	0.0000	100.0000
合计			522.04	4723.92	100		

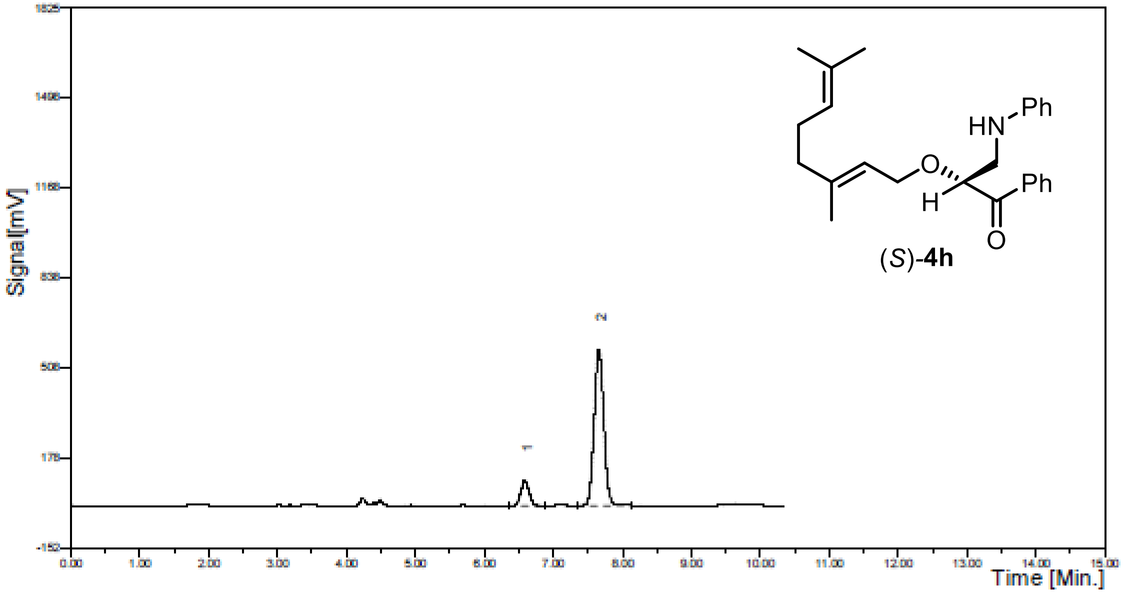

组分表

#	组分名	保留时间(min)	峰高(mV)	峰面积(mV.sec)	面积百分比(%)	浓 度	样品含量(%)
1	Unknown	6.59000	93.94	791.79	12.4316	0.0000	100.0000
2	Unknown	7.65667	571.71	5577.41	87.5684	0.0000	100.0000
合计			665.65	6369.20	100		

Condition: hexane/2-propanol = 20:1
Flow rate = 1.0 mL/min
λ = 254 nm
Chiral IA

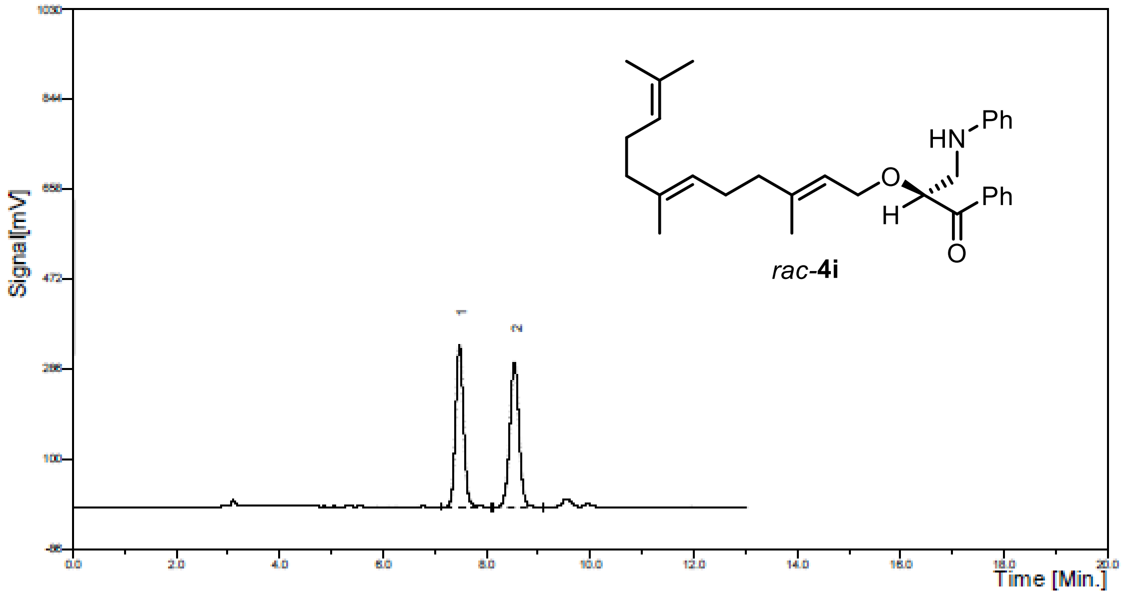

组分表

#	组分名	保留时间(min)	峰高(mV)	峰面积(mV.sec)	面积百分比(%)	浓 度	样品含量(%)
1	Unknown	7.47750	335.94	3627.45	49.0885	0.0000	0.0000
2	Unknown	8.54083	299.82	3762.16	50.9115	0.0000	100.0000
合计			635.76	7389.61	100		

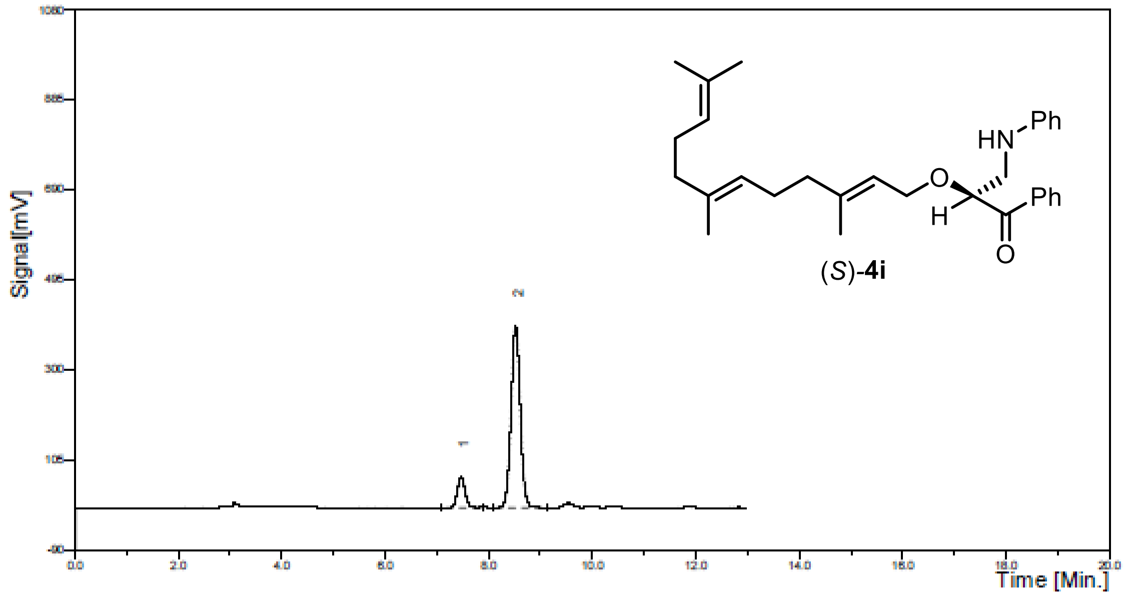

组分表

#	组分名	保留时间(min)	峰高(mV)	峰面积(mV.sec)	面积百分比(%)	浓 度	样品含量(%)
1	Unknown	7.47167	66.50	683.15	12.2564	0.0000	100.0000
2	Unknown	8.52583	393.63	4890.67	87.7436	0.0000	100.0000
合计			460.13	5573.81	100		

Condition: hexane/2-propanol = 3:1
Flow rate = 1.0 mL/min
λ = 254 nm
Chiral IC

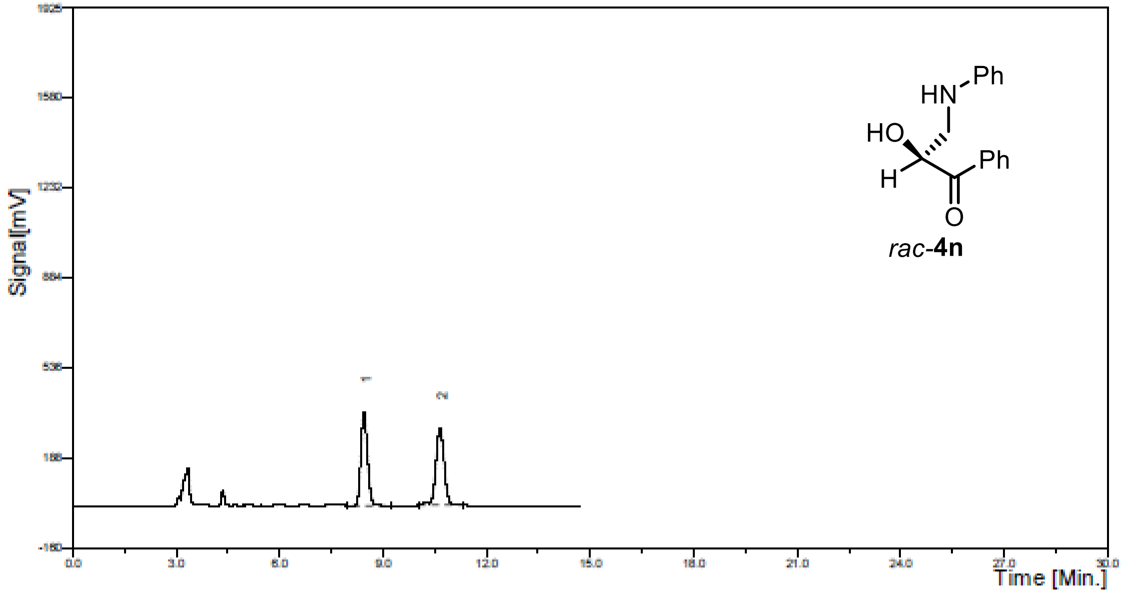

组分表

#	组分名	保留时间(min)	峰高(mV)	峰面积(mV.sec)	面积百分比(%)	浓 度	样品含量(%)
1	Unknown	8.44583	363.50	4872.72	49.4729	0.0000	0.0000
2	Unknown	10.65000	294.39	4976.55	50.5271	0.0000	100.0000
合计			657.89	9849.27	100		

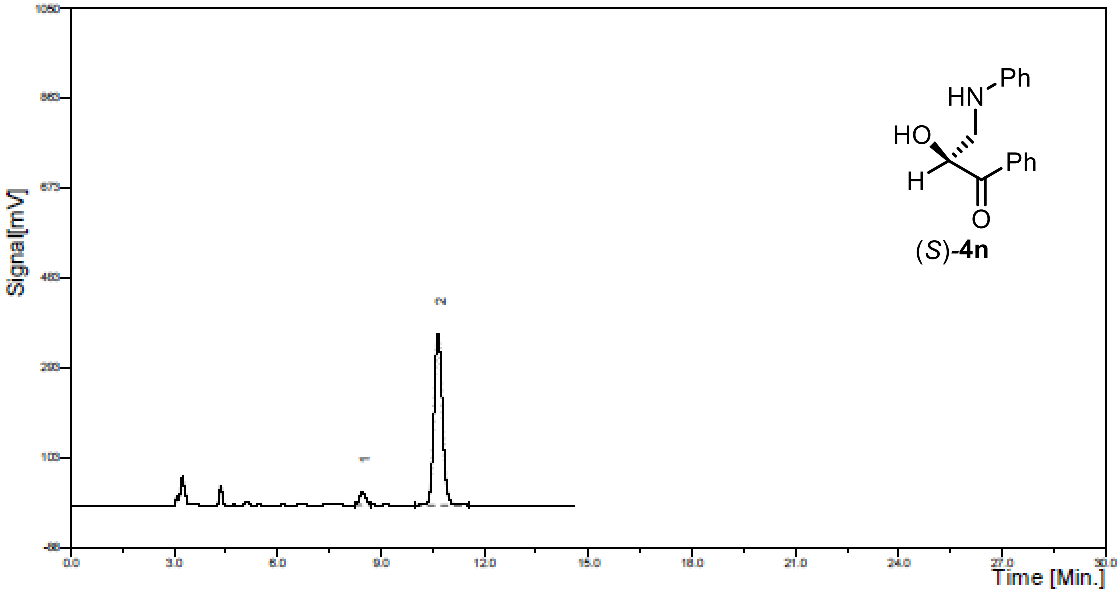

组分表

#	组分名	保留时间(min)	峰高(mV)	峰面积(mV.sec)	面积百分比(%)	浓 度	样品含量(%)
1	Unknown	8.46583	26.98	335.58	5.1538	0.0000	100.0000
2	Unknown	10.65917	362.16	6175.69	94.8462	0.0000	0.0000
合计			389.14	6511.26	100		

Condition: hexane/2-propanol = 10:1
Flow rate = 1.0 mL/min
λ = 254 nm
Chiral IC

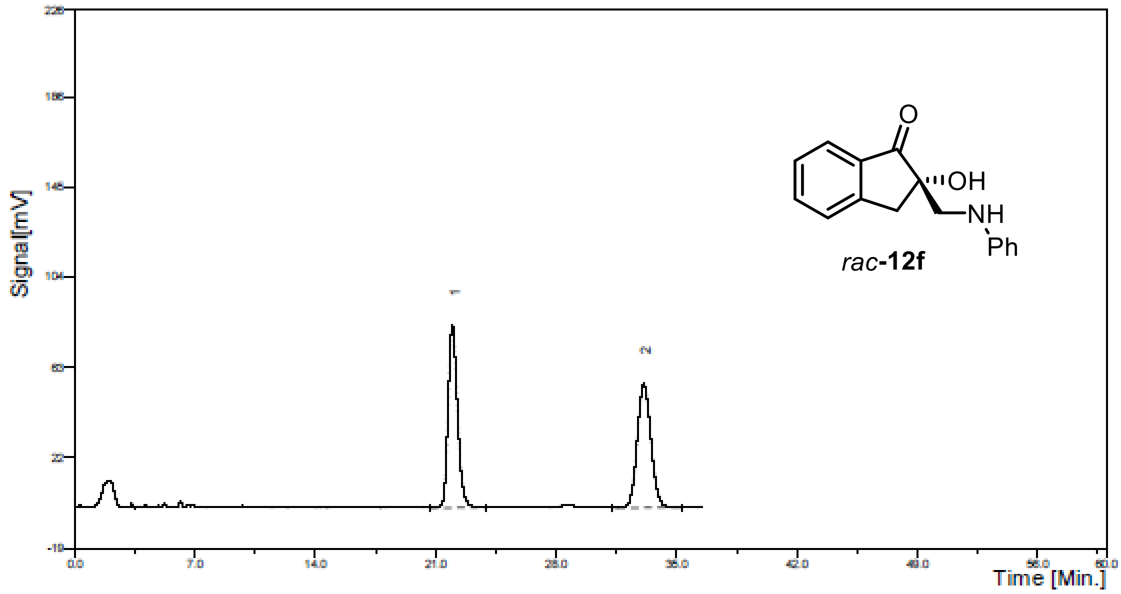

组分表

#	组分名	保留时间(min)	峰高(mV)	峰面积(mV.sec)	面积百分比(%)	浓 度	样品含量(%)
1	Unknown	21.97250	82.82	3148.65	50.5554	0.0000	100.0000
2	Unknown	33.08167	56.17	3079.47	49.4446	0.0000	100.0000
合计			139.00	6228.11	100		

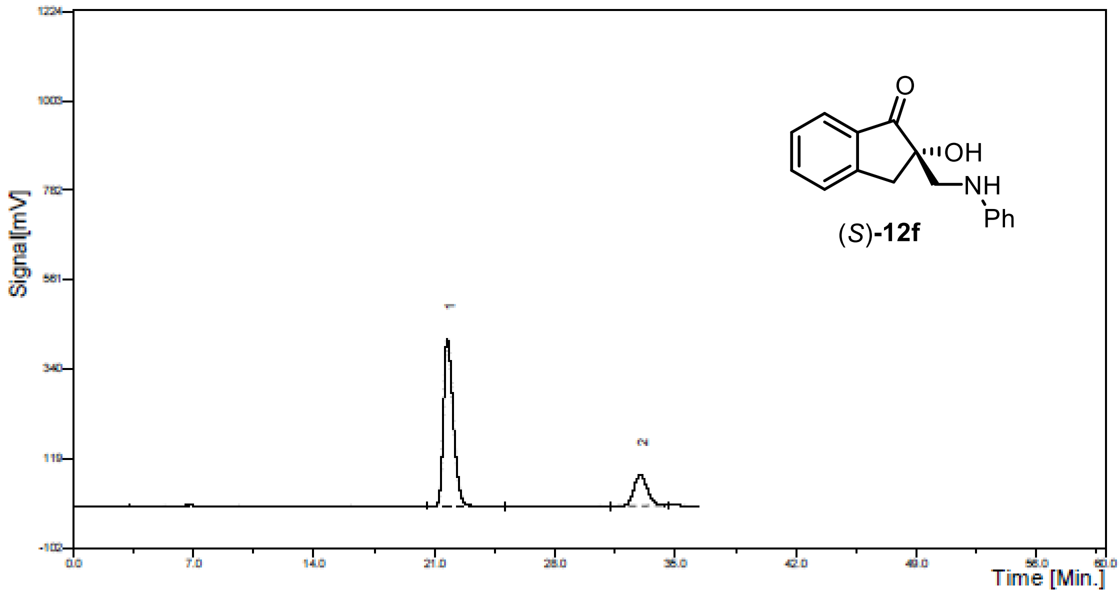

组分表

#	组分名	保留时间(min)	峰高(mV)	峰面积(mV.sec)	面积百分比(%)	浓 度	样品含量(%)
1	Unknown	21.74583	414.02	16434.24	80.0320	0.0000	100.0000
2	Unknown	32.97500	75.96	4100.34	19.9680	0.0000	0.0000
合计			489.98	20534.58	100		

13. Supplementary references

1. For preparation of **2a**: Hansen, J. H., Parr, Br. T., Pelphrey, P., Jin, Q., Autschbach, J., Davies, H. M. L. Rhodium(II)-Catalyzed Cross-Coupling of Diazo Compounds. *Angew. Chem. Int. Ed.* **50**, 2544-2548 (2011).
2. For preparation of **2b-2d**: Nicolaou, K. C., Mathison, C. J. N., Montagnon, T. *o*-Iodoxybenzoic Acid (IBX) as a Viable Reagent in the Manipulation of Nitrogen- and Sulfur-Containing Substrates: Scope, Generality, and Mechanism of IBX-Mediated Amine Oxidations and Dithiane Deprotections. *J. Am. Chem. Soc.* **126**, 5192-5201 (2004).
3. For preparation of **2e** and **2e`**: (a) Yang, K., Zhang, J., Li, Y., Cheng, B., Zhao, L., Zhai, H. Facile Synthesis of 2-Arylphenols via Palladium-Catalyzed Cross-Coupling of Aryl Iodides with 6-Diazo-2-cyclohexenones. *Org. Lett.* **15**, 808-811, (2013); (b) Jiang, Y., Khong, V. Z. Y., Lourdusamy, E., Park, C.-M. Synthesis of 2-aminofurans and 2-unsubstituted furansviacarbenoid-mediated [3+2] cycloaddition. *Chem. Commun.* **48**, 3133-3135, (2012).
4. Zhu, C., Xu, G., Sun, J. Gold-Catalyzed Formal [4+1]/[4+3] Cycloadditions of Diazo Esters with Triazines. *Angew. Chem. Int. Ed.* **55**, 11867-11871 (2016).
5. For preparation of **6a-6h**: Hu, W., Zhou, J., Xu, X., Liu, J., Gong, L. (*R*)-3,3'-Bis(9-Phenanthryl)-1,1'-Binaphthalene-2,2'-Diyl Hydrogen Phosphate. *Org. Synth.* **88**, 406-417 (2011).
6. For preparation of **6i**: Storer, R. I., Carrera, D. E., Ni, Y., MacMillan, D. W. C. Enantioselective Organocatalytic Reductive Amination. *J. Am. Chem. Soc.* **128**, 84-86 (2006).
7. For preparation of **6j**: Zhu, S. S., Cefalo, D. R., La, D. S., Jamieson, J. Y., Davis, W. M., Hoveyda, A. H., Schrock, R. R. Chiral Mo-Binol Complexes: Activity, Synthesis, and Structure. Efficient Enantioselective Six-Membered Ring Synthesis through Catalytic Metathesis. *J. Am. Chem. Soc.* **121**, 8251-8259 (1999).
8. For preparation of **6j**: Klussmann, M., Ratjen, L., Hoffmann, S., Wakchaure, V., Goddard, R., List, B. Synthesis of TRIP and Analysis of Phosphate Salt Impurities. *Synlett.* **2010**, 2189-2192 (2010).
